# Supplementary figures and images for: Unveiling a missing component of the atypical type IV secretion system required for natural transformation of Helicobacter pylori
Source: PLoS Pathog. 2026 Jul 14;22(7):e1014140. doi: 10.1371/journal.ppat.1014140 (PMC13395361; doi:10.1371/journal.ppat.1014140)

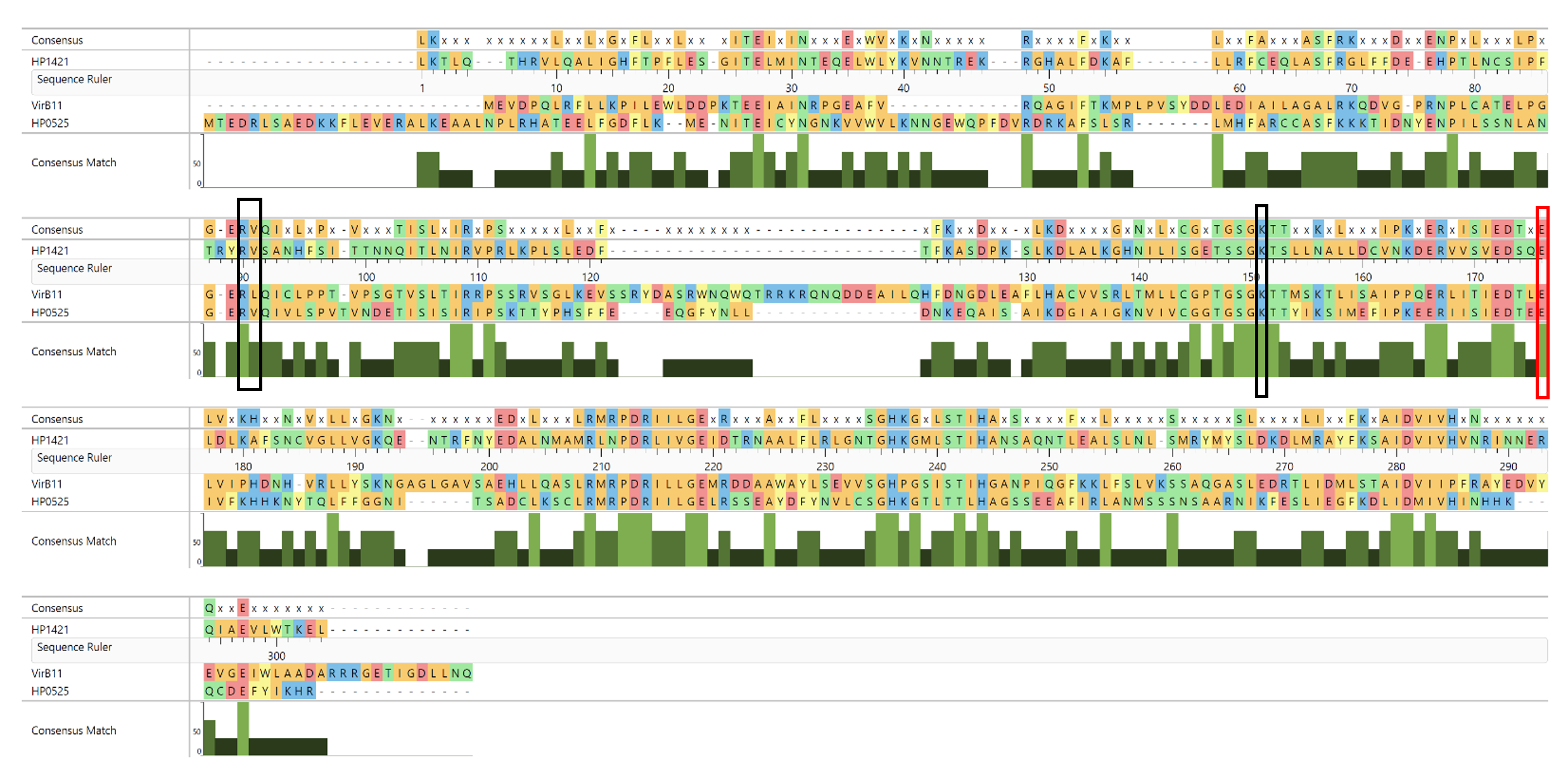

Supplement: S1 Fig — The following sequences were used in the alignment: VirB11 from Ti plasmid pTiBo542 Agrobacterium tumefaciens, HP0525 and HP1421 both from H. pylori. Conserved residues also found in other ATPases are shown in black boxes, residues involved in ATP binding and hydrolysis are boxed in red. Amino acid numbering at the sequence ruler refers to HP1421. (TIF) [file ppat.1014140.s001.tif]

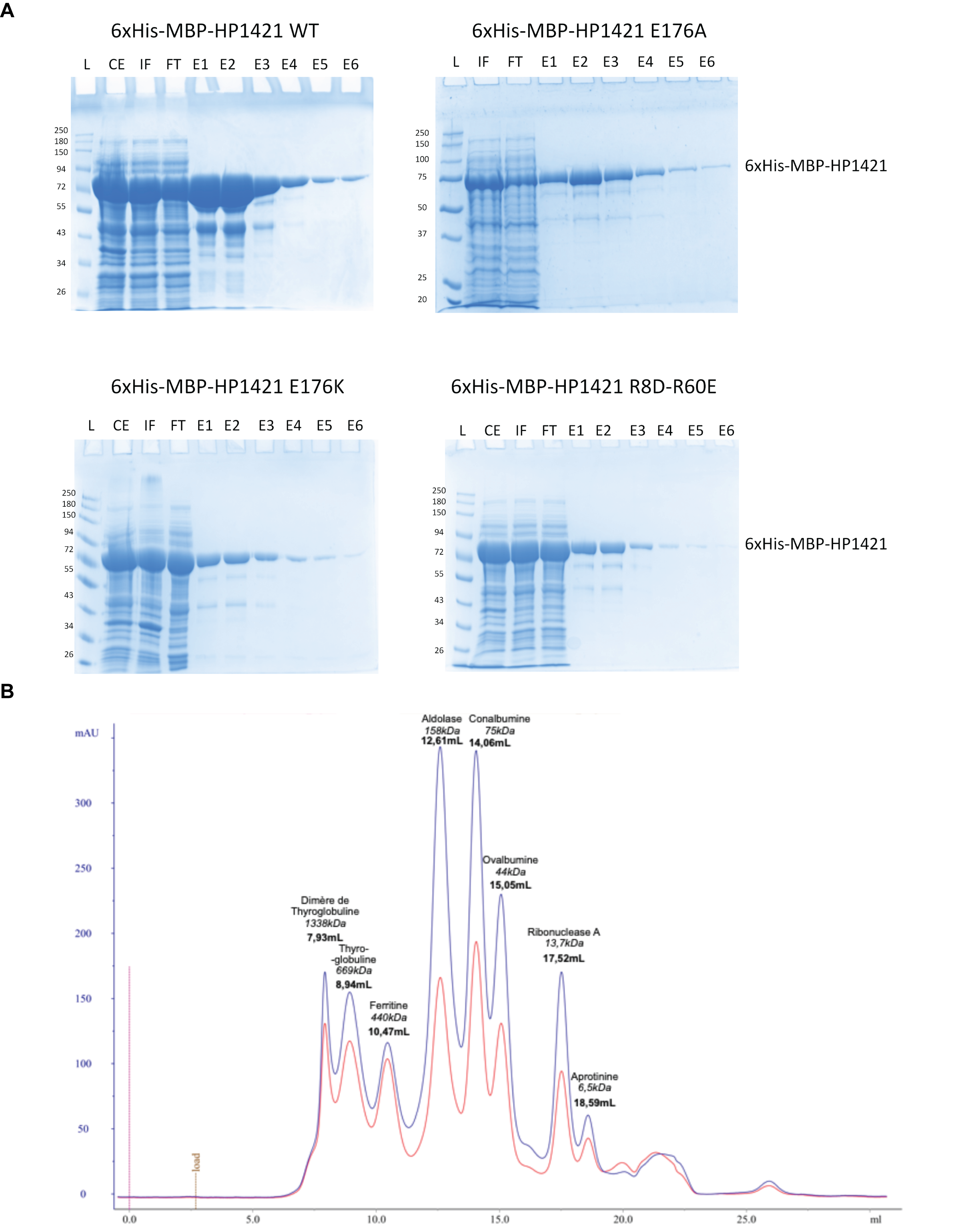

Supplement: S2 Fig — (A) HP1421 purification. 12.5% acrylamide gels stained by Coomassie blue. L: ladder; CE: crude extract; IF: input fraction; FT: flowthrough; E1-6: elution fractions. (B) Chromatogram for size exclusion chromatography (SEC) of protein standards using a Superdex 200 column. (TIF) [file ppat.1014140.s002.tif]

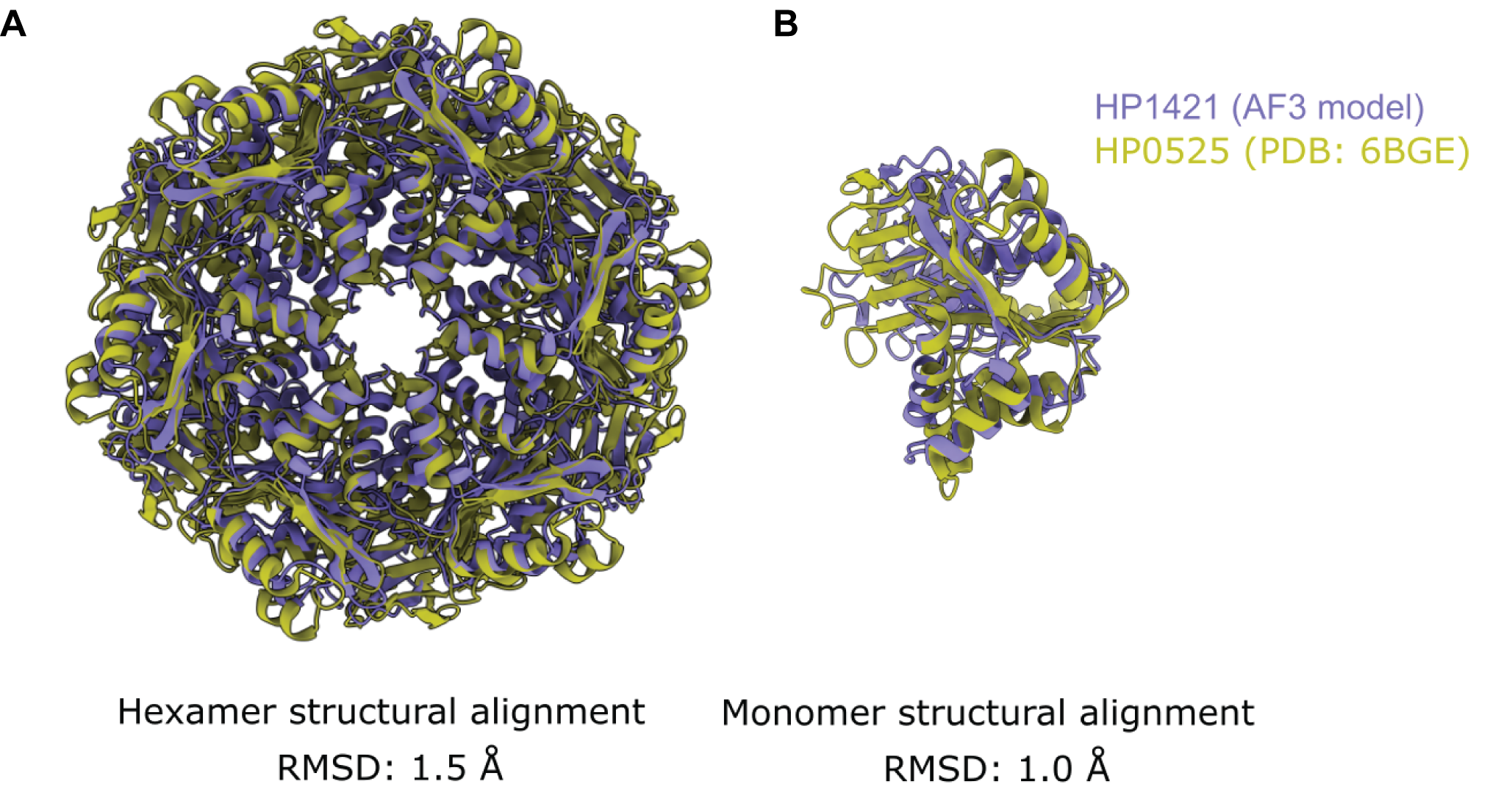

Supplement: S3 Fig — Model from AlphaFold3 using 6 copies of HP1421. HP0525 structure from Protein Data Bank (6GBE) (A) hexamer structural alignment (RMSD: 1.5 Å). (B) monomer structural alignment (RMSD: 1.0 Å). (TIF) [file ppat.1014140.s003.tif]

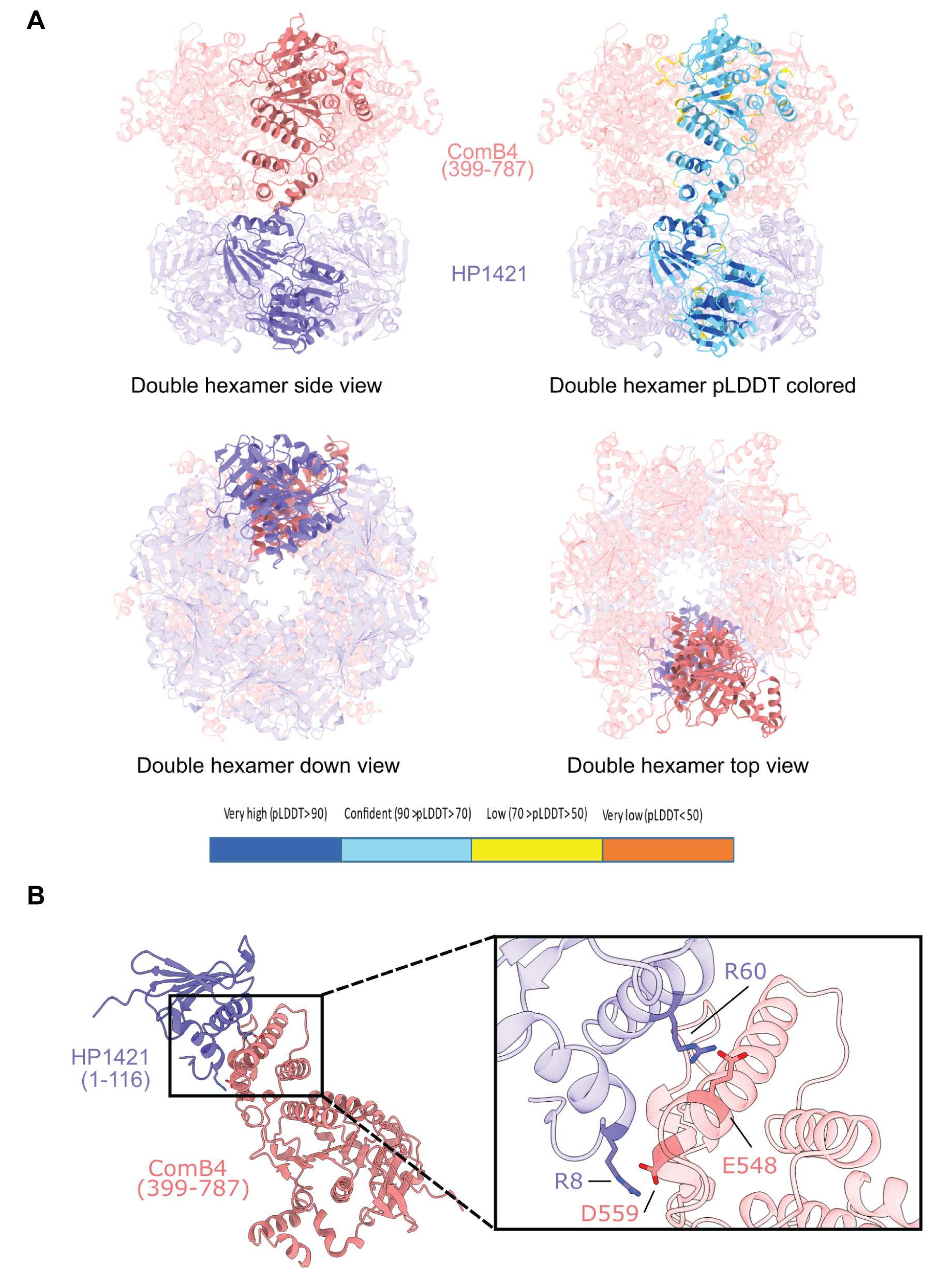

Supplement: S4 Fig — (A) AlphaFold3 model of HP1421 and ComB4 double hexamer in different views. HP1421 (blue) and ComB4 C-terminal domain (salmon) double hexamer prediction (pTM = 0.82, ipTM = 0.81) with one copy of each highlighted to ease visualisation. Panels represent double hexamer model. Upper left: side view; upper right: side view with a pLDDT colored HP1421/ComB4 complex; lower left: top view; lower right: down view. (B) AlphaFold 2 model of HP1421 and ComB4. HP1421 N-ter (1–116) and ComB4 C-ter (399–787) AF2 model (pTM = 0.89, ipTM = 0.88). Zoom: HP1421 and ComB4 predicted interface and main evolutionary conserved residues (R8 and R60 on HP1421, E548 and D559 on ComB4). (TIF) [file ppat.1014140.s004.tif]

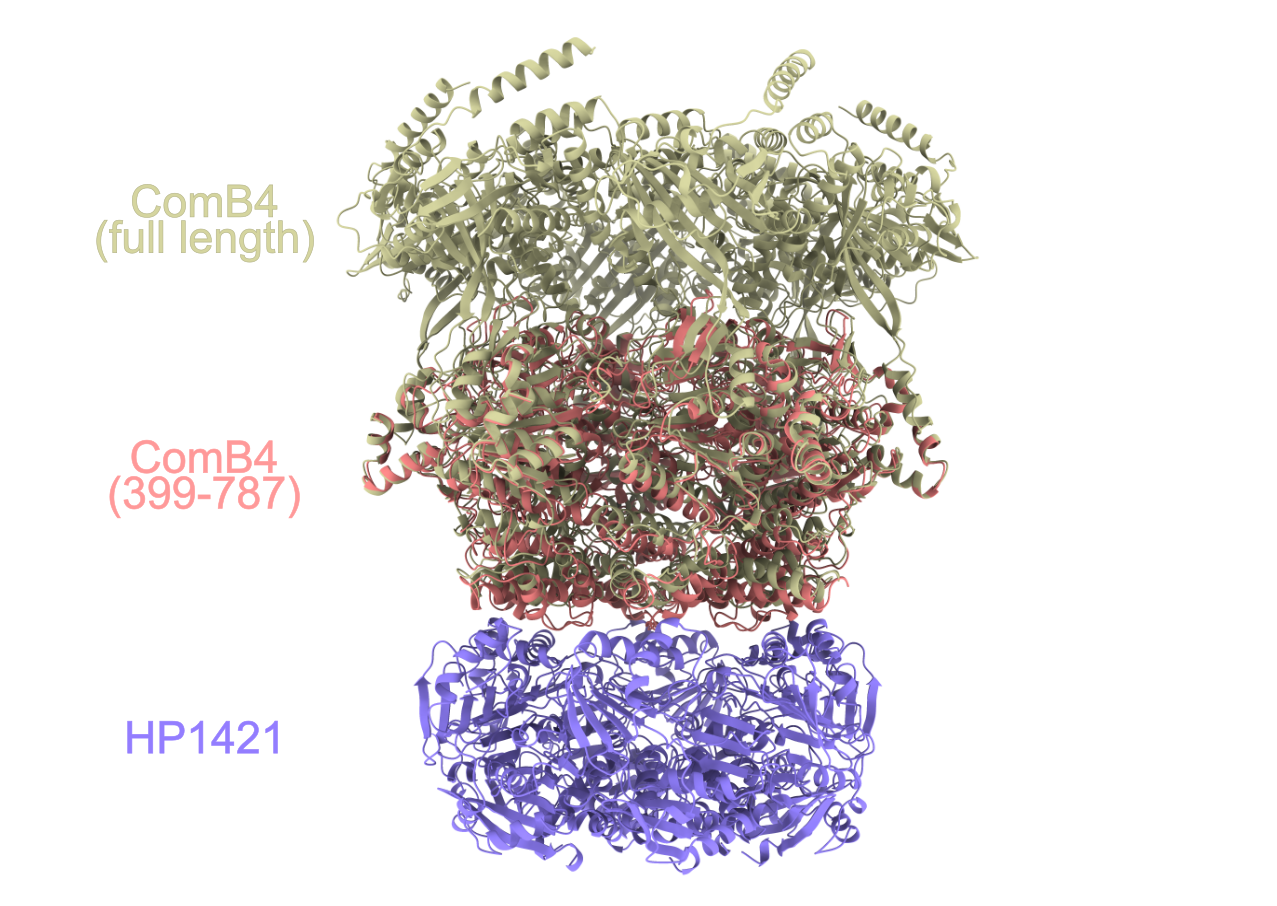

Supplement: S5 Fig — AlphaFold3 models of HP1421 (full length) and ComB4 (399–787) double hexamer and full length ComB4 hexamer. (TIFF) [file ppat.1014140.s005.tiff]

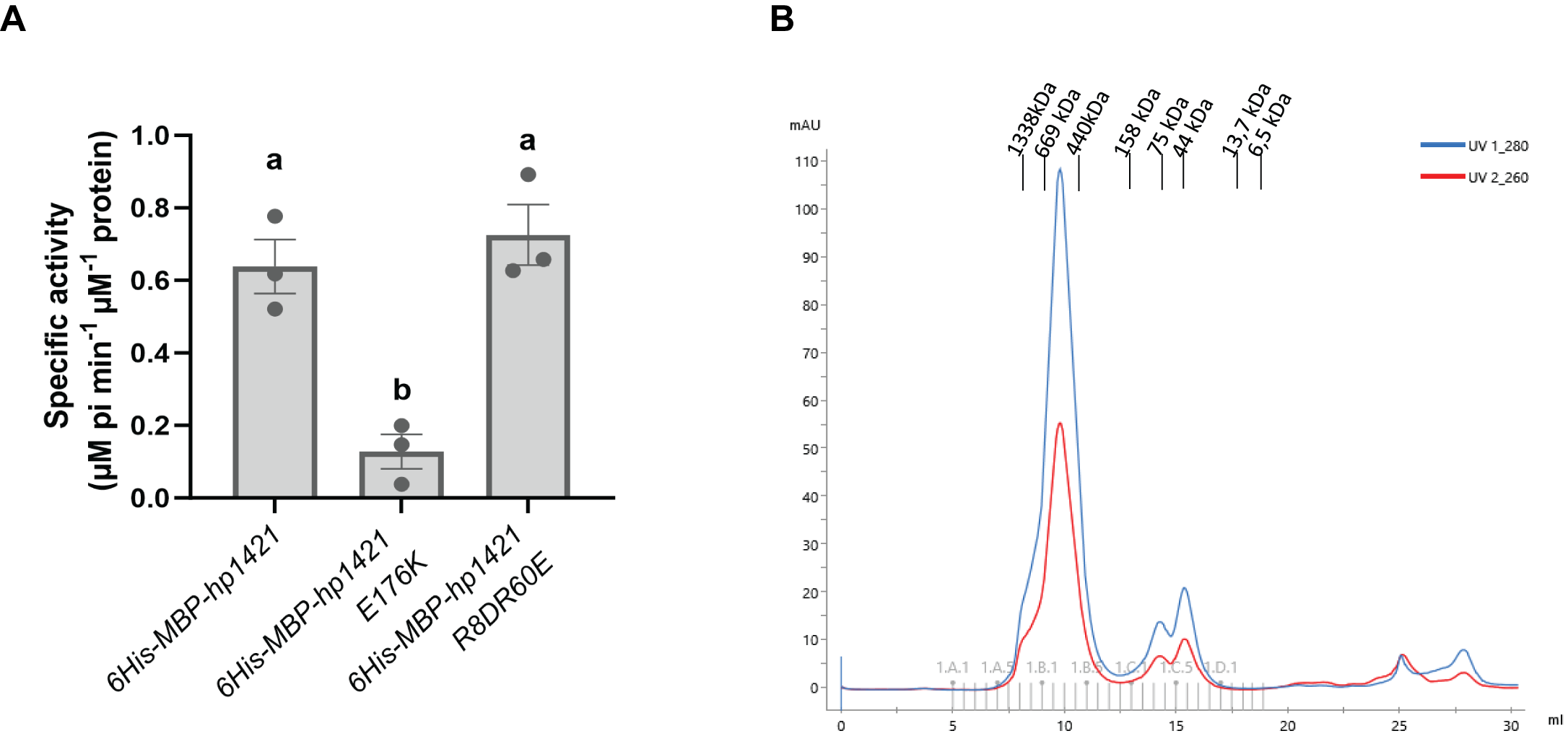

Supplement: S6 Fig — (A) Specific ATPase activity of purified HP1421 variants was measured by following pi release due to ATP hydrolysis, data represent Mean + SEM (n = 3). Different lowercase letters indicate significant differences (p < 0.05) between treatments (Tukey’s test). (B) Chromatogram for size exclusion chromatography (SEC) of 6xHis-MBP-HP1421 R8D-R60E using a Superdex 200 column. (TIF) [file ppat.1014140.s006.tif]

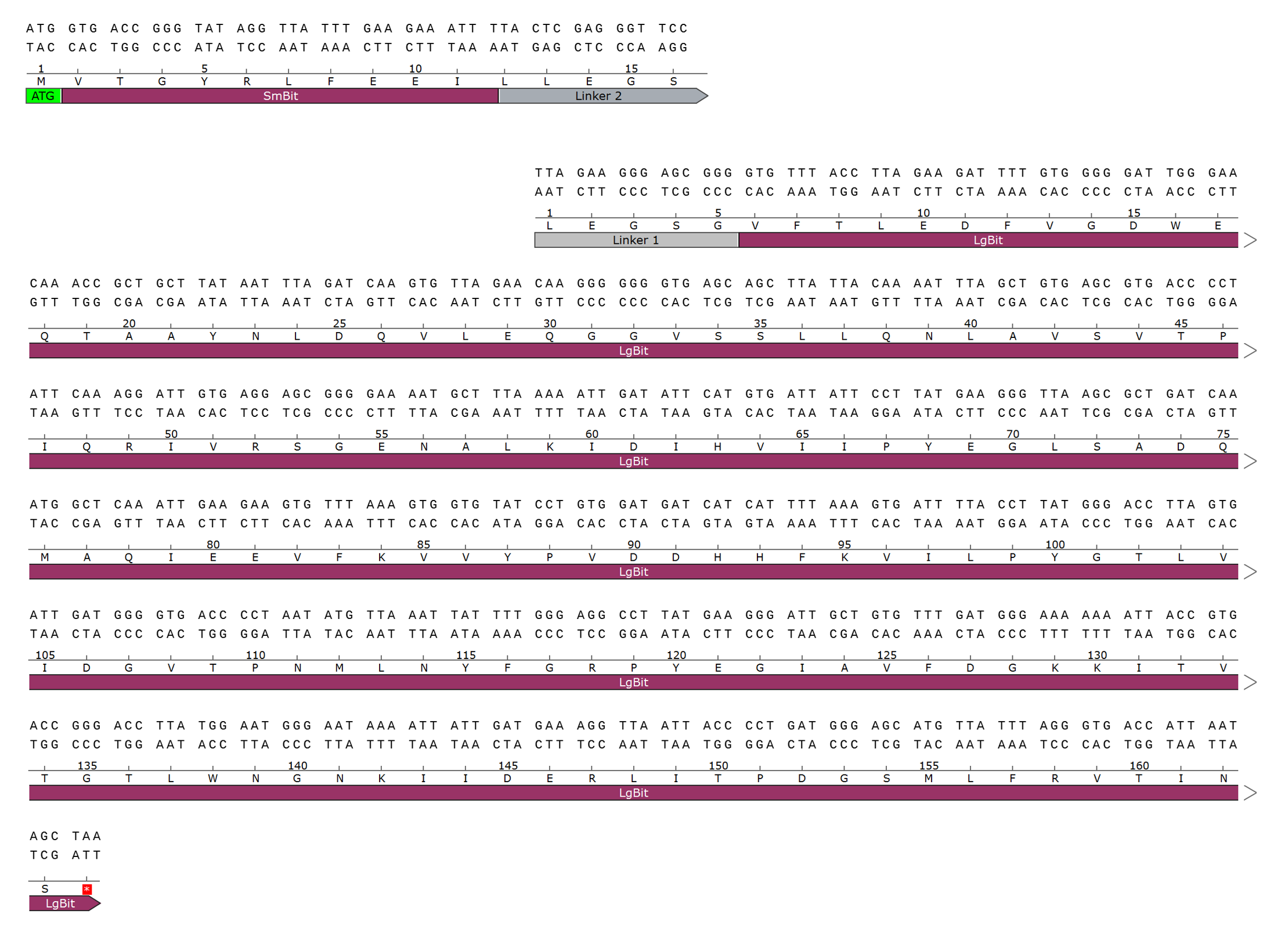

Supplement: S7 Fig — Codon optimized sequences of SmBit for N terminal tagging and LgBit for C-terminal tagging of Nanoluciferase for H. pylori expression. (TIF) [file ppat.1014140.s007.tif]

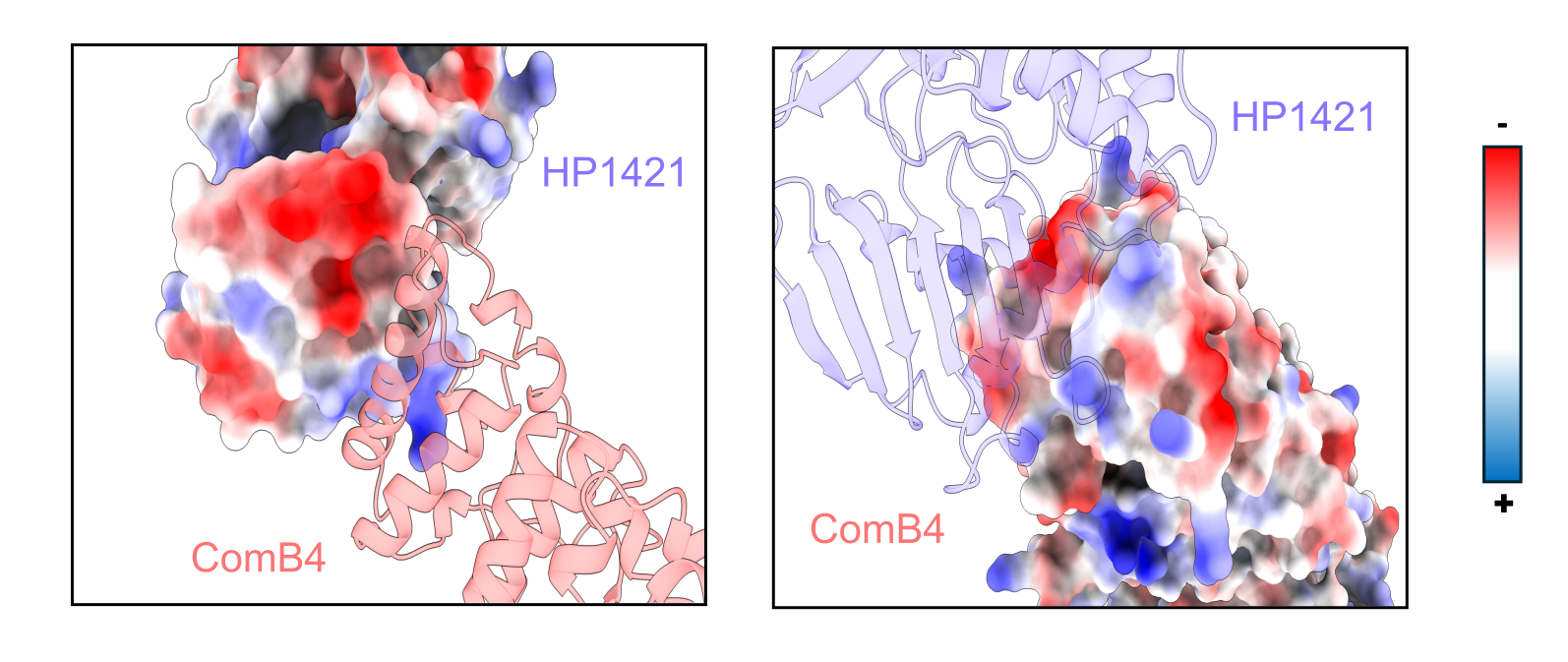

Supplement: S8 Fig — AlphaFold3 model of HP1421 and ComB4 zoom on each protein interface area with electrostatic potential shown. (TIFF) [file ppat.1014140.s008.tiff]
