# Supplementary material for: Unveiling a missing component of the atypical type IV secretion system required for natural transformation of Helicobacter pylori
Source: PLoS Pathog. 2026 Jul 14;22(7):e1014140. doi: 10.1371/journal.ppat.1014140 (PMC13395361; doi:10.1371/journal.ppat.1014140)
Supplement: S1 Table — (PDF) [file ppat.1014140.s009.pdf]

S1 Table. *H. pylori* strains

Strain	Genotype	Source
1	26695	(1)
134	26695 *strep*^{R}	(1)
770	26695 *comB2::Km*	(3)
1188, 1189	26695 *hp1421::Kan*	This work
1283, 1284	26695 *hp1421::Kan pUreA-FLAG-hp1421-Cm*	This work
1303, 1304	26695 *hp1421::Kan pUreA-FLAG-hp1421 E176A-Cm*	This work
1376, 1377	26695 *hp1421::Kan pUreA-FLAG-hp1421 E176K-Cm*	This work
1378,1399	26695 *hp1421::Kan pUreA-FLAG-hp1421 E176K-Cm* c*omB4::Apra*	This work
1397	26695 c*omB4::Apra*	This work
1494, 1495	26695 *hp1421::Kan rdxA-pcomH-FLAG-hp1421-Cm*	This work
1630, 1631	26695 *hp1421::Kan* c*omB4::Apra pUreA-comB4 E548R/D559R-AT-pcomH-FLAG-hp1421-CmR*	This work
1632, 1633	26695 *hp1421::Kan* c*omB4::Apra pUreA-comB4-AT-pcomH-FLAG-hp1421-CmR*	This work
1634, 1635	26695 *hp1421::Kan* c*omB4::Apra pUreA-comB4-AT-pcomH-FLAG-hp1421 R8D/R60E-CmR*	This work
1642	26695 *pUreA-comB4-SmBit-pcomH-LgBit-hp1421-CmR*	This work
1643	26695 *pUreA-comB4-SmBit-pcomH-LgBit-hp1421R8D/R60E-CmR*	This work
1665	26695 *pUreA-comB4 E548R/D559R-SmBit-pcomH-LgBit-hp1421-CmR*	This work

1. Marsin S, Mathieu A, Kortulewski T, Guérois R, Radicella JP. Unveiling novel RecO distant orthologues involved in homologous recombination. PLoS Genet. 2008;4(8).

2.Mathieu A, O'Rourke EJ, Radicella JP*. Helicobacter pylori* genes involved in avoidance of mutations induced by 8-oxoguanine. J bacteriol. 2006; 188(21), 7464–7469.

3. Corbinais C, Mathieu A, Kortulewski T, Radicella JP, Marsin S. Following transforming DNA in Helicobacter pylori from uptake to expression. Mol Microbiol. 2016;101(6):1039– 53
