# Supplementary material for: Unveiling a missing component of the atypical type IV secretion system required for natural transformation of Helicobacter pylori
Source: PLoS Pathog. 2026 Jul 14;22(7):e1014140. doi: 10.1371/journal.ppat.1014140 (PMC13395361; doi:10.1371/journal.ppat.1014140)
Supplement: S2 Table — (PDF) [file ppat.1014140.s010.pdf]

S2 Table. *E. coli* strains

Strain	Description	Source
DH5-α	For plasmid construction and maintenance	New England Biolabs
BL21 (DE3)	For protein expression and purification	New England Biolabs
BTH101	For bacterial two-hybrid tests	Euromedex
