# Supplementary material for: Unveiling a missing component of the atypical type IV secretion system required for natural transformation of Helicobacter pylori
Source: PLoS Pathog. 2026 Jul 14;22(7):e1014140. doi: 10.1371/journal.ppat.1014140 (PMC13395361; doi:10.1371/journal.ppat.1014140)
Supplement: S3 Table — (PDF) [file ppat.1014140.s011.pdf]

S3 Table. Plasmids used in this study

Plasmid	Genotype	Source
p1361	pBAD33 pEYY166 Dronpa-MTSBs	Lab. collection
p1524, p1980	pJET 1.2 *hp1421::Kan*	This work
p1561	pKT25 T25-Zip	Euromedex
p1562	pUT18C *T18-Zip*	Euromedex
p1665	pUT18C *T18-hp1421-Amp*	This work
p1765	pRSF Duet *6xHis-MBP-TEV-hp1421-Kan*	Laurent Terradot
p1770	pKT25 *T25-hp1421-Kan*	This work
p1781	pJET 1.2 *pUreA-FLAG-hp1421-Cm*	This work
p1787	pJET 1.2 *pUreA-FLAG-hp1421 E176A-Cm*	This work
p1792	pRSF Duet *6xHis-MBP-TEV-hp1421 E176A-Kan*	This work
p1811	pRSF Duet *6xHis-MBP-TEV-hp1421 E176K-Kan*	This work
p1813	pJET 1.2 *pUreA-FLAG-hp1421 E176K-Cm*	This work
p1820	pJET 1.2 c*omB4::Apra*	This work
p1854	pJET 1.2 *rdxA-pcomH-FLAG-hp1421-Cm*	This work
p1916	pRSF Duet *6xHis-MBP-TEV-hp1421 R8D/R60E-Kan*	This work
p1946	pJET 1.2 *pUreA-comB4-AT-pcomH-FLAG-hp1421-CmR*	This work
p1947	pJET 1.2 *pUreA-comB4-AT-pcomH-FLAG-hp1421 R8D/R60E-CmR*	This work
p1948	pJET 1.2 *pUreA-comB4 E548R/D559R-AT-pcomH-FLAG-hp1421-CmR*	This work
p1961	pJET 1.2 *pUreA-comB4-SmBit-pcomH-LgBit-hp1421R8D/R60E-CmR*	This work
p1964	pJET 1.2 *pUreA-comB4 E548R/D559R-SmBit-pcomH-LgBit-hp1421-CmR*	This work
p1968	pJET 1.2 *pUreA-comB4-SmBit-pcomH-LgBit-hp1421-CmR*	This work
p1983	pUT18C *T18-hp1421 R8D/R60E-Amp*	This work
p1984	pKT25 T25-hp1421 *R8D/R60E-Kan*	This work
p1985	pUT18 - negative control for BTH	Euromedex
p1986	pKT25 - negative control for BTH	Euromedex
