# Supplementary material for: Unveiling a missing component of the atypical type IV secretion system required for natural transformation of Helicobacter pylori
Source: PLoS Pathog. 2026 Jul 14;22(7):e1014140. doi: 10.1371/journal.ppat.1014140 (PMC13395361; doi:10.1371/journal.ppat.1014140)
Supplement: S4 Table — (PDF) [file ppat.1014140.s012.pdf]

S4 Table. Oligonucleotides used in this study

Name	Sequence (5'-3')	Description
Oc61	AGACAGCCAAAAATTGGATTTAAAAGC	*Construction p1811 and p1813*
Op31	ATGGAATTGAATCAACCACCACTC	Fw HP0247 (DNA uptake, Recipient)
Op32	TTAACGGCGTTTGGGTTTTTTAG	Rv HP0247 (DNA uptake, Recipient)
Oc139	GAATGTTAGAAAAGCTTTTAAGGGTACCCGGGTGACTAAC	*Construction p1820*
Oc140	CTAGCTCCTCAAAAGTTTCTGGATCCCCGTGTCATTATTC	*Construction p1820*
Oc141	GAATAATGACACGGGGATCCAGAAACTTTTGAGGAGCTAG	*Construction p1820*
Oc142	GTTAGTCACCCGGGTACCCTTAAAAGCTTTTCTAACATTC	*Construction p1820*
Oc266	GAATAAGCATGGATTACAAGGATGACGACGATAAGTTGAAAACTTTAC	*Construction p1854*
Oc267	CCTTGTAATCCATGCTTATTCCTTTTTATTTTAGATTTTTGGATTATAGC	*Construction p1854*
Oc306	ATGACGTTTTACAAGCCCTAATTGGCCATTTTAC	*SDM R8D hp1421 (p1983 and 1984)*
Oc307	CTTGTAAAACGTCATGGGTTTGTAAAGTTTTC	*SDM R8D hp1421 (p1983 and 1984)*
Oc308	TTTGCTGGAGTTTTGCGAGCAATTGGCTAG	*SDM R60E hp1421(p1983 and 1984)*
Oc309	AACTCCAGCAAAAACGCCTTATCAAAAAGTG	*SDM R60E hp1421 (p1983 and 1984)*
Oc345	CTTTATTTTCAGGGCGCCATGGGAAAAACTTTACAAACCCAT	*Construction p1916*
Oc346	CCGAGCTCGAATTCGGATCCTTAAAGCTCTTTAGTCCATAAG	*Construction p1916*
Oc347	CTTATGGACTAAAGAGCTTTAAGGATCCGAATTCGAGCTCGG	*Construction p1916*
Oc348	ATGGGTTTGTAAAGTTTTTCCCATGGCGCCCTGAAAATAAAG	*Construction p1916*
Oc429	TAGCTCGAGGGTTCCGGACACTTCTTTTCAAATCCCACAAC	*Construction p1946, p1947 and p1948*
Oc430	GGGGTGAGTTTCATGGATCCAGCGGATCCTTAC	*Construction p1946, p1947 and p1948*
Oc431	TAAGGATCCGCTGGATCCATGAAACTCACCCC	*Construction p1946, p1947 and p1948*
Oc432	GTGTCCGGAACCCTCGAGCTATTCGGTCAGGCGG	*Construction p1946, p1947 and p1948*
Oc454	GCTCCTCAAAAGTTTCTCG	*Construction p1961, p1964 and p1968*
Oc455	GCGAGAAACTTTTGAGGAGCCTCGAGGGTTCCGGA	*Construction p1961, p1964 and p1968*
Oc456	GGGATTTGAAAAGAAGTGTTATAAAATTTCTTCAAATAACCTATACCC	*Construction p1961, p1964 and p1968*

Oc482	TACTGGATGAATTGTTTTAGATGCCCCTAAAATCCTTAAAAAAC	*Construction p1980*
Oc483	GATATTCTCATTTTAGCCATAAGCCCTATCCTTTTATCATC	*Construction p1980*
Op99	CACTTCTTTTCAAATCCCACAACC	*Construction p1961, p1964 and p1968*
Op284	GGACACCCGTTCGCGATTG	Fw p1361 (DNA uptake, Donor)
Op285	CGTCAGGATGGCCTTCTGC	Rv p1361 (DNA uptake, Donor)
Op376	GATAAAAGGATAGGGCTTTTGAAAACTTTACAAACCCAATGGCTAAAATGAGAATATC	*Construction p1524*
Op377	GGATTTTAGGGGCATTTAAAGCTCTTTAGTCCATACTAAAACAATTCATCCAG	*Construction p1524*
Op378	CTGGATGAATTGTTTTAGTATGGACTAAAGAGCTTTAAATGCCCCTAAAATCC	*Construction p1524*
Op379	GATATTCTCATTTTAGCCATTGGGTTTGTAAAGTTTTCAAAAGCCCTATCCTTTTATC	*Construction p1524*
Op722	GGTCGACTCTAGAGGATCCCCGGGTACCTAAGTTGAAAACTTTACAAACCCATAGAG	*Construction p1770*
Op723	CGTTGTAAAACGACGGCCGAATTCTTAGTTAAAGCTCTTTAGTCCATAAGACTTCAGC	*Construction p1770*
Op724	CTCTATGGGTTTGTAAAGTTTTCAACTTAGGTACCCGGGGATCCTCTAGAGTCGACC	*Construction p1770*
Op725	GCTGAAGTCTTATGGACTAAAGAGCTTTAACTAAGAATTCGGCCGTCGTTTTACAACG	*Construction p1770*
Op726	GGGTACCGAGCTCGAATTCATCGATATTGAAAACTTTACAAACCCATAGAG	*Construction p1665*
Op727	ATTGTACTGAGAGTGCACCATATTACTTAGTTAAAGCTCTTTAGTCCATAAGACTTCAGC	*Construction p1665*
Op728	CTCTATGGGTTTGTAAAGTTTTCAATATCGATGAATTCGAGCTCGGTACCC	*Construction p1665*
Op729	GCTGAAGTCTTATGGACTAAAGAGCTTTAACTAAGTAATATGGTGCACTCTCAGTACAAT	*Construction p1665*
Op940	AGACAGCCAAGCATTGGATTTAAAAGC	*Construction p1787 and 1792*
Op941	TCAACGCTCACCACCCTT	*Construction p1787, p1792, p1811 and p1813*
Op1007	GAATAGGAGAATAAGGAATTCATGGATTACAAGGATGACGACGATAAGTTGAAAACTTTAC	*Construction p1781*
Op1008	GATCTAGAGTCGCGGCCGCTTTAAAGCTCTTTAGT	*Construction p1781*
Op1009	GTAAAGTTTTCAACTTATCGTCGTCATCCTTGTAATCCATGAATTCCTTATTCTCCTATTC	*Construction p1781*
Op1010	ACTAAAGAGCTTTAAAGCGGCCGCGACTCTAGATC	*Construction p1781*
SeqAM15	CTAAAACAATTCATCCAGTAAAATATA	Rv KanR
SeqAM16	ATGGCTAAAATGAGAATATCACCGGA	*Fw KanR*
