# Supplementary material for: Unveiling a missing component of the atypical type IV secretion system required for natural transformation of Helicobacter pylori
Source: PLoS Pathog. 2026 Jul 14;22(7):e1014140. doi: 10.1371/journal.ppat.1014140 (PMC13395361; doi:10.1371/journal.ppat.1014140)
Supplement: S5 Table — (PDF) [file ppat.1014140.s013.pdf]

genome_gembase_name size #replicons #CDS (excl.pseudo) #pseudogenes #rna taxID species_taxid organism_name taxonomy bioproject biosample asm_name assembly(GCF*)
HEAC001.0523.00001 1557588 2 1400 114 45 382638 212 Helicobacter acinonychis str. Sheeba ['Bacteria', 'Campylobacterota', 'Epsilonproteobacteria', 'Campylobacterales', 'Helicobacteraceae', 'Helicobacter'] PRJNA224116 SAMEA3138199 ASM930v1 GCF_000009305.1
HEAI001.0523.00001 1609065 4 1483 123 46 1578720 1578720 Helicobacter ailurogastricus strain type ['Bacteria', 'Proteobacteria', 'Epsilonproteobacteria', 'Campylobacterales', 'Helicobacteraceae', 'Helicobacter'] PRJNA224116 SAMD00519148 ASM2599811v1 GCF_025998115.1
HEAP001.0523.00001 2067954 2 1959 43 51 135569 135569 Helicobacter apodemus strain SCJK1 ['Bacteria', 'Proteobacteria', 'Epsilonproteobacteria', 'Campylobacterales', 'Helicobacteraceae', 'Helicobacter'] PRJNA224116 SAMN07204495 ASM309757v1 GCF_003097575.1
HEBI001.0523.00001 2620399 1 2278 42 47 37372 37372 Helicobacter bilis strain AAQJH ['Bacteria', 'Proteobacteria', 'Epsilonproteobacteria', 'Campylobacterales', 'Helicobacteraceae', 'Helicobacter'] PRJNA224116 SAMN03485893 ASM199998v1 GCF_001999985.1
HECE001.0523.00001 1960111 2 1714 50 48 182217 138563 Helicobacter cetorum MIT 00-7128 ['Bacteria', 'Proteobacteria', 'Epsilonproteobacteria', 'Campylobacterales', 'Helicobacteraceae', 'Helicobacter'] PRJNA224116 SAMN02603199 ASM25925v1 GCF_000259255.1
HECE001.0523.00002 1847790 2 1653 81 46 1163745 138563 Helicobacter cetorum MIT 99-5656 ['Bacteria', 'Campylobacterota', 'Epsilonproteobacteria', 'Campylobacterales', 'Helicobacteraceae', 'Helicobacter'] PRJNA224116 SAMN02603198 ASM25927v1 GCF_000259275.1
HECH001.0523.00001 1420261 1 1302 14 49 45498 45498 Helicobacter cholecystus strain NCTC13205 ['Bacteria', 'Proteobacteria', 'Epsilonproteobacteria', 'Campylobacterales', 'Helicobacteraceae', 'Helicobacter'] PRJNA224116 SAMEA104224780 57728_B01 GCF_900638485.1
HECI001.0523.00001 2154041 1 2213 36 48 213 213 Helicobacter cinaedi strain P01D0000 ['Bacteria', 'Proteobacteria', 'Epsilonproteobacteria', 'Campylobacterales', 'Helicobacteraceae', 'Helicobacter'] PRJNA224116 SAMD00106671 ASM321372v2 GCF_003213725.2
HECI001.0523.00002 2221266 1 2241 34 48 213 213 Helicobacter cinaedi strain P03D0629 ['Bacteria', 'Proteobacteria', 'Epsilonproteobacteria', 'Campylobacterales', 'Helicobacteraceae', 'Helicobacter'] PRJNA224116 SAMD00106672 ASM321382v2 GCF_003213825.2
HECI001.0523.00003 2165979 1 2231 34 48 213 213 Helicobacter cinaedi strain P06D0798 ['Bacteria', 'Proteobacteria', 'Epsilonproteobacteria', 'Campylobacterales', 'Helicobacteraceae', 'Helicobacter'] PRJNA224116 SAMD00106675 ASM321415v2 GCF_003214155.2
HECI001.0523.00004 2199263 1 2240 34 48 213 213 Helicobacter cinaedi strain MRY08-1234 ['Bacteria', 'Proteobacteria', 'Epsilonproteobacteria', 'Campylobacterales', 'Helicobacteraceae', 'Helicobacter'] PRJNA224116 SAMD00047012 ASM357419v1 GCF_003574195.1
HECI001.0523.00005 2256207 2 2292 29 48 213 213 Helicobacter cinaedi strain D7095 ['Bacteria', 'Proteobacteria', 'Epsilonproteobacteria', 'Campylobacterales', 'Helicobacteraceae', 'Helicobacter'] PRJNA224116 SAMN15354345 ASM1493109v1 GCF_014931095.1
HECI001.0523.00006 2218389 1 2235 33 48 213 213 Helicobacter cinaedi strain 2017D-0197 ['Bacteria', 'Proteobacteria', 'Epsilonproteobacteria', 'Campylobacterales', 'Helicobacteraceae', 'Helicobacter'] PRJNA224116 SAMN15354330 ASM1493115v1 GCF_014931155.1
HECI001.0523.00007 2194683 8 2217 22 48 213 213 Helicobacter cinaedi strain T36 ['Bacteria', 'Pseudomonadota', 'Epsilonproteobacteria', 'Campylobacterales', 'Helicobacteraceae', 'Helicobacter'] PRJNA224116 SAMD00406394 ASM2165557v1 GCF_021655575.1
HECI001.0523.00008 1771717 1 1712 10 48 213 213 Helicobacter cinaedi strain 94105 ['Bacteria', 'Pseudomonadota', 'Epsilonproteobacteria', 'Campylobacterales', 'Helicobacteraceae', 'Helicobacter'] PRJNA224116 SAMD00406774 ASM2165559v1 GCF_021655595.1
HECI001.0523.00009 2240130 1 2304 40 48 213 213 Helicobacter cinaedi isolate MGYG-HGUT-01432 ['Bacteria', 'Proteobacteria', 'Epsilonproteobacteria', 'Campylobacterales', 'Helicobacteraceae', 'Helicobacter'] PRJNA224116 SAMEA5850935 UHGG_MGYG-HGUT-01432 GCF_902381705.1
HECI001.0523.00010 2240130 1 2331 38 48 537971 213 Helicobacter cinaedi CCUG 18818 = ATCC BAA-847 ['Bacteria', 'Proteobacteria', 'Epsilonproteobacteria', 'Campylobacterales', 'Helicobacteraceae', 'Helicobacter'] PRJNA224116 SAMD00061010 ASM34997v1 GCF_000349975.1
HECI001.0523.00011 2101402 2 2117 42 48 1172562 213 Helicobacter cinaedi PAGU611 ['Bacteria', 'Proteobacteria', 'Epsilonproteobacteria', 'Campylobacterales', 'Helicobacteraceae', 'Helicobacter'] PRJNA224116 SAMD00061064 ASM28463v1 GCF_000284635.1
HEEN001.0523.00001 1594790 1 1458 22 49 222136 222136 Helicobacter enhydrae strain MIT 01-6242 ['Bacteria', 'Proteobacteria', 'Epsilonproteobacteria', 'Campylobacterales', 'Helicobacteraceae', 'Helicobacter'] PRJNA224116 SAMN05379701 ASM169333v1 GCF_001693335.1
HEFE001.0523.00001 1672681 1 1641 19 45 936155 214 Helicobacter felis ATCC 49179 ['Bacteria', 'Proteobacteria', 'Epsilonproteobacteria', 'Campylobacterales', 'Helicobacteraceae', 'Helicobacter'] PRJNA224116 SAMEA2272759 ASM20059v1 GCF_000200595.1
HEHE001.0523.00001 1660951 3 1509 193 46 35817 35817 Helicobacter heilmannii strain type ['Bacteria', 'Proteobacteria', 'Epsilonproteobacteria', 'Campylobacterales', 'Helicobacteraceae', 'Helicobacter'] PRJNA224116 SAMD00519147 ASM2599805v1 GCF_025998055.1
HEHE002.0523.00001 1799146 1 1799 8 43 235279 32025 Helicobacter hepaticus ATCC 51449 ['Bacteria', 'Proteobacteria', 'Epsilonproteobacteria', 'Campylobacterales', 'Helicobacteraceae', 'Helicobacter'] PRJNA224116 SAMN02604097 ASM790v1 GCF_000007905.1
HEHI002.0523.00001 1829936 1 1696 33 48 1591088 1591088 Helicobacter himalayensis strain YS1 ['Bacteria', 'Pseudomonadota', 'Epsilonproteobacteria', 'Campylobacterales', 'Helicobacteraceae', 'Helicobacter'] PRJNA224116 SAMN04578135 ASM160209v1 GCF_001602095.1
HEMU001.0523.00001 1576607 1 1395 19 47 217 217 Helicobacter mustelae strain NCTC12198 ['Bacteria', 'Proteobacteria', 'Epsilonproteobacteria', 'Campylobacterales', 'Helicobacteraceae', 'Helicobacter'] PRJNA224116 SAMEA44549668 51342_F02 GCF_900476215.1
HEMU001.0523.00002 1578097 1 1380 24 47 679897 217 Helicobacter mustelae 12198 ['Bacteria', 'Proteobacteria', 'Epsilonproteobacteria', 'Campylobacterales', 'Helicobacteraceae', 'Helicobacter'] PRJNA224116 SAMEA2272735 ASM9198v1 GCF_000091985.1
HEPU001.0523.00001 1733410 1 1652 12 48 35818 35818 Helicobacter pullorum strain NCTC13154 ['Bacteria', 'Proteobacteria', 'Epsilonproteobacteria', 'Campylobacterales', 'Helicobacteraceae', 'Helicobacter'] PRJNA224116 SAMEA104210757 57428_B01 GCF_900638355.1
HEPY001.0523.00001 1604233 1 1430 62 45 210 210 Helicobacter pylori strain BM013A ['Bacteria', 'Proteobacteria', 'Epsilonproteobacteria', 'Campylobacterales', 'Helicobacteraceae', 'Helicobacter'] PRJNA224116 SAMN02736809 ASM68566v1 GCF_000685665.1
HEPY001.0523.00002 1659060 1 1459 100 45 210 210 Helicobacter pylori strain BM012B ['Bacteria', 'Proteobacteria', 'Epsilonproteobacteria', 'Campylobacterales', 'Helicobacteraceae', 'Helicobacter'] PRJNA224116 SAMN02736818 ASM68570v1 GCF_000685705.1
HEPY001.0523.00003 1604212 1 1427 63 45 210 210 Helicobacter pylori strain BM013B ['Bacteria', 'Proteobacteria', 'Epsilonproteobacteria', 'Campylobacterales', 'Helicobacteraceae', 'Helicobacter'] PRJNA224116 SAMN02736816 ASM68574v1 GCF_000685745.1
HEPY001.0523.00004 1586473 1 1394 87 44 210 210 Helicobacter pylori strain Hp238 ['Bacteria', 'Proteobacteria', 'Epsilonproteobacteria', 'Campylobacterales', 'Helicobacteraceae', 'Helicobacter'] PRJNA224116 SAMN03154060 ASM81702v1 GCF_000817025.1
HEPY001.0523.00005 1667303 1 1465 87 45 210 210 Helicobacter pylori strain 26695-1MET ['Bacteria', 'Proteobacteria', 'Epsilonproteobacteria', 'Campylobacterales', 'Helicobacteraceae', 'Helicobacter'] PRJNA224116 SAMN03268365 ASM82702v1 GCF_000827025.1
HEPY001.0523.00006 1631276 2 1425 83 45 210 210 Helicobacter pylori strain 7C ['Bacteria', 'Pseudomonadota', 'Epsilonproteobacteria', 'Campylobacterales', 'Helicobacteraceae', 'Helicobacter'] PRJNA224116 SAMN03144736 ASM143351v1 GCF_001433515.1
HEPY001.0523.00007 1617826 1 1438 106 42 210 210 Helicobacter pylori strain L7 ['Bacteria', 'Proteobacteria', 'Epsilonproteobacteria', 'Campylobacterales', 'Helicobacteraceae', 'Helicobacter'] PRJNA224116 SAMN02731779 ASM165337v1 GCF_001653375.1
HEPY001.0523.00008 1614411 1 1431 92 42 210 210 Helicobacter pylori strain DU15 ['Bacteria', 'Proteobacteria', 'Epsilonproteobacteria', 'Campylobacterales', 'Helicobacteraceae', 'Helicobacter'] PRJNA224116 SAMN02731778 ASM165339v1 GCF_001653395.1
HEPY001.0523.00009 1659899 1 1455 87 42 210 210 Helicobacter pylori strain CC33C ['Bacteria', 'Proteobacteria', 'Epsilonproteobacteria', 'Campylobacterales', 'Helicobacteraceae', 'Helicobacter'] PRJNA224116 SAMN02731783 ASM165341v1 GCF_001653415.1
HEPY001.0523.00010 1510564 1 1366 64 42 210 210 Helicobacter pylori strain ausabrJ05 ['Bacteria', 'Proteobacteria', 'Epsilonproteobacteria', 'Campylobacterales', 'Helicobacteraceae', 'Helicobacter'] PRJNA224116 SAMN02731780 ASM165343v1 GCF_001653435.1
HEPY001.0523.00011 1570310 1 1393 84 42 210 210 Helicobacter pylori strain K26A1 ['Bacteria', 'Proteobacteria', 'Epsilonproteobacteria', 'Campylobacterales', 'Helicobacteraceae', 'Helicobacter'] PRJNA224116 SAMN02731784 ASM165345v1 GCF_001653455.1
HEPY001.0523.00012 1531450 1 1366 87 42 210 210 Helicobacter pylori strain PNG84A ['Bacteria', 'Proteobacteria', 'Epsilonproteobacteria', 'Campylobacterales', 'Helicobacteraceae', 'Helicobacter'] PRJNA224116 SAMN02731781 ASM165347v1 GCF_001653475.1
HEPY001.0523.00013 1584869 2 1393 90 46 210 210 Helicobacter pylori strain G272 ['Bacteria', 'Proteobacteria', 'Epsilonproteobacteria', 'Campylobacterales', 'Helicobacteraceae', 'Helicobacter'] PRJNA224116 SAMN07338981 ASM222257v1 GCF_002222575.1
HEPY001.0523.00014 1670779 1 1469 90 45 210 210 Helicobacter pylori strain dRdM1 ['Bacteria', 'Proteobacteria', 'Epsilonproteobacteria', 'Campylobacterales', 'Helicobacteraceae', 'Helicobacter'] PRJNA224116 SAMN08388634 ASM290649v1 GCF_002906495.1
HEPY001.0523.00015 1666471 1 1464 88 45 210 210 Helicobacter pylori strain 26695-dR ['Bacteria', 'Proteobacteria', 'Epsilonproteobacteria', 'Campylobacterales', 'Helicobacteraceae', 'Helicobacter'] PRJNA224116 SAMN08388633 ASM290653v1 GCF_002906535.1
HEPY001.0523.00016 1666735 1 1466 87 45 210 210 Helicobacter pylori strain 26695-dRdM2 ['Bacteria', 'Proteobacteria', 'Epsilonproteobacteria', 'Campylobacterales', 'Helicobacteraceae', 'Helicobacter'] PRJNA224116 SAMN08388635 ASM290655v1 GCF_002906555.1
HEPY001.0523.00017 1674002 1 1472 77 45 210 210 Helicobacter pylori strain B128_1 ['Bacteria', 'Proteobacteria', 'Epsilonproteobacteria', 'Campylobacterales', 'Helicobacteraceae', 'Helicobacter'] PRJNA224116 SAMN06216064 ASM295197v1 GCF_002951975.1
HEPY001.0523.00018 1674015 1 1476 74 45 210 210 Helicobacter pylori strain 7.13_D3a ['Bacteria', 'Proteobacteria', 'Epsilonproteobacteria', 'Campylobacterales', 'Helicobacteraceae', 'Helicobacter'] PRJNA224116 SAMN06216085 ASM295245v1 GCF_002952455.1
HEPY001.0523.00019 1674013 1 1475 74 45 210 210 Helicobacter pylori strain 7.13_D3b ['Bacteria', 'Proteobacteria', 'Epsilonproteobacteria', 'Campylobacterales', 'Helicobacteraceae', 'Helicobacter'] PRJNA224116 SAMN06216086 ASM295247v1 GCF_002952475.1
HEPY001.0523.00020 1674013 1 1474 75 45 210 210 Helicobacter pylori isolate 7.13_D3c ['Bacteria', 'Proteobacteria', 'Epsilonproteobacteria', 'Campylobacterales', 'Helicobacteraceae', 'Helicobacter'] PRJNA224116 SAMN06216087 ASM295249v1 GCF_002952495.1
HEPY001.0523.00021 1674018 1 1480 74 45 210 210 Helicobacter pylori strain 7.13_R3c ['Bacteria', 'Proteobacteria', 'Epsilonproteobacteria', 'Campylobacterales', 'Helicobacteraceae', 'Helicobacter'] PRJNA224116 SAMN06216078 ASM295251v1 GCF_002952515.1
HEPY001.0523.00022 1674019 1 1481 74 45 210 210 Helicobacter pylori strain 7.13_R3b ['Bacteria', 'Proteobacteria', 'Epsilonproteobacteria', 'Campylobacterales', 'Helicobacteraceae', 'Helicobacter'] PRJNA224116 SAMN06216077 ASM295253v1 GCF_002952535.1
HEPY001.0523.00023 1674009 1 1474 74 45 210 210 Helicobacter pylori strain 7.13_R2c ['Bacteria', 'Proteobacteria', 'Epsilonproteobacteria', 'Campylobacterales', 'Helicobacteraceae', 'Helicobacter'] PRJNA224116 SAMN06216075 ASM295255v1 GCF_002952555.1
HEPY001.0523.00024 1673951 1 1473 76 45 210 210 Helicobacter pylori strain 7.13_R2a ['Bacteria', 'Proteobacteria', 'Epsilonproteobacteria', 'Campylobacterales', 'Helicobacteraceae', 'Helicobacter'] PRJNA224116 SAMN06216073 ASM295257v1 GCF_002952575.1
HEPY001.0523.00025 1674009 1 1475 75 45 210 210 Helicobacter pylori strain 7.13_R1c ['Bacteria', 'Proteobacteria', 'Epsilonproteobacteria', 'Campylobacterales', 'Helicobacteraceae', 'Helicobacter'] PRJNA224116 SAMN06216072 ASM295259v1 GCF_002952595.1
HEPY001.0523.00026 1674020 1 1480 74 45 210 210 Helicobacter pylori strain 7.13_R3a ['Bacteria', 'Proteobacteria', 'Epsilonproteobacteria', 'Campylobacterales', 'Helicobacteraceae', 'Helicobacter'] PRJNA224116 SAMN06216076 ASM295261v1 GCF_002952615.1
HEPY001.0523.00027 1674026 1 1481 74 45 210 210 Helicobacter pylori strain 7.13_R2b ['Bacteria', 'Proteobacteria', 'Epsilonproteobacteria', 'Campylobacterales', 'Helicobacteraceae', 'Helicobacter'] PRJNA224116 SAMN06216074 ASM295263v1 GCF_002952635.1
HEPY001.0523.00028 1674010 1 1476 75 45 210 210 Helicobacter pylori strain 7.13_R1b ['Bacteria', 'Proteobacteria', 'Epsilonproteobacteria', 'Campylobacterales', 'Helicobacteraceae', 'Helicobacter'] PRJNA224116 SAMN06216071 ASM295265v1 GCF_002952655.1
HEPY001.0523.00029 1674018 1 1482 74 45 210 210 Helicobacter pylori strain 7.13_R1a ['Bacteria', 'Proteobacteria', 'Epsilonproteobacteria', 'Campylobacterales', 'Helicobacteraceae', 'Helicobacter'] PRJNA224116 SAMN06216070 ASM295267v1 GCF_002952675.1
HEPY001.0523.00030 1668735 1 1470 88 45 210 210 Helicobacter pylori strain dRdM2addM2 ['Bacteria', 'Proteobacteria', 'Epsilonproteobacteria', 'Campylobacterales', 'Helicobacteraceae', 'Helicobacter'] PRJNA224116 SAMN08388637 ASM295355v1 GCF_002953555.1
HEPY001.0523.00031 1645738 1 1448 60 45 210 210 Helicobacter pylori strain FDAARGOS_300 ['Bacteria', 'Proteobacteria', 'Epsilonproteobacteria', 'Campylobacterales', 'Helicobacteraceae', 'Helicobacter'] PRJNA224116 SAMN06173313 ASM298370v1 GCF_002983705.1
HEPY001.0523.00032 1680898 1 1500 75 45 210 210 Helicobacter pylori strain FDAARGOS_298 ['Bacteria', 'Proteobacteria', 'Epsilonproteobacteria', 'Campylobacterales', 'Helicobacteraceae', 'Helicobacter'] PRJNA224116 SAMN06173311 ASM305066v1 GCF_003050665.1
HEPY001.0523.00033 1678260 1 1483 69 45 210 210 Helicobacter pylori strain HP14039 ['Bacteria', 'Campylobacterota', 'Epsilonproteobacteria', 'Campylobacterales', 'Helicobacteraceae', 'Helicobacter'] PRJNA224116 SAMN06329538 ASM363685v2 GCF_003636855.2
HEPY001.0523.00034 1563701 1 1391 64 45 210 210 Helicobacter pylori strain Hpbs1 ['Bacteria', 'Campylobacterota', 'Epsilonproteobacteria', 'Campylobacterales', 'Helicobacteraceae', 'Helicobacter'] PRJNA224116 SAMN10461767 ASM385547v1 GCF_003855475.1
HEPY001.0523.00035 1658454 3 1453 81 45 210 210 Helicobacter pylori strain HP42K ['Bacteria', 'Campylobacterota', 'Epsilonproteobacteria', 'Campylobacterales', 'Helicobacteraceae', 'Helicobacter'] PRJNA224116 SAMN10181238 ASM393908v1 GCF_003939085.1
HEPY001.0523.00036 1637442 1 1428 89 45 210 210 Helicobacter pylori strain H-137 ['Bacteria', 'Campylobacterota', 'Epsilonproteobacteria', 'Campylobacterales', 'Helicobacteraceae', 'Helicobacter'] PRJNA224116 SAMN08195713 ASM395739v1 GCF_003957395.1
HEPY001.0523.00037 1514674 1 1354 71 45 210 210 Helicobacter pylori strain Hpbs3 ['Bacteria', 'Campylobacterota', 'Epsilonproteobacteria', 'Campylobacterales', 'Helicobacteraceae', 'Helicobacter'] PRJNA224116 SAMN10663175 ASM408807v1 GCF_004088075.1
HEPY001.0523.00038 1535248 1 1386 67 45 210 210 Helicobacter pylori strain Hpbs2 ['Bacteria', 'Campylobacterota', 'Epsilonproteobacteria', 'Campylobacterales', 'Helicobacteraceae', 'Helicobacter'] PRJNA224116 SAMN10663081 ASM408815v1 GCF_004088155.1
HEPY001.0523.00039 1680829 1 1487 93 45 210 210 Helicobacter pylori strain ATCC 43504 ['Bacteria', 'Campylobacterota', 'Epsilonproteobacteria', 'Campylobacterales', 'Helicobacteraceae', 'Helicobacter'] PRJNA224116 SAMD00051588 HPATCC43504_01 GCF_004295525.1
HEPY001.0523.00040 1609151 2 1416 75 45 210 210 Helicobacter pylori strain PMSS1 ['Bacteria', 'Campylobacterota', 'Epsilonproteobacteria', 'Campylobacterales', 'Helicobacteraceae', 'Helicobacter'] PRJNA224116 SAMD00051589 HP_PMSS1_01 GCF_004295545.1
HEPY001.0523.00041 1636444 1 1430 75 45 210 210 Helicobacter pylori strain 479-A2-EK4 ['Bacteria', 'Campylobacterota', 'Epsilonproteobacteria', 'Campylobacterales', 'Helicobacteraceae', 'Helicobacter'] PRJNA224116 SAMN10053852 ASM633726v1 GCF_006337265.1
HEPY001.0523.00042 1649543 1 1460 94 45 210 210 Helicobacter pylori strain 476-A2-EK2 ['Bacteria', 'Campylobacterota', 'Epsilonproteobacteria', 'Campylobacterales', 'Helicobacteraceae', 'Helicobacter'] PRJNA224116 SAMN10053800 ASM633728v1 GCF_006337285.1
HEPY001.0523.00043 1580965 1 1417 79 45 210 210 Helicobacter pylori strain 381-F-EK9 ['Bacteria', 'Campylobacterota', 'Epsilonproteobacteria', 'Campylobacterales', 'Helicobacteraceae', 'Helicobacter'] PRJNA224116 SAMN10053787 ASM633730v1 GCF_006337305.1
HEPY001.0523.00044 1588985 1 1418 85 45 210 210 Helicobacter pylori strain 381-C-EK1 ['Bacteria', 'Campylobacterota', 'Epsilonproteobacteria', 'Campylobacterales', 'Helicobacteraceae', 'Helicobacter'] PRJNA224116 SAMN10053769 ASM633732v1 GCF_006337325.1
HEPY001.0523.00045 1611628 1 1420 76 45 210 210 Helicobacter pylori strain 169-C-EK8 ['Bacteria', 'Campylobacterota', 'Epsilonproteobacteria', 'Campylobacterales', 'Helicobacteraceae', 'Helicobacter'] PRJNA224116 SAMN10053716 ASM633734v1 GCF_006337345.1
HEPY001.0523.00046 1540135 1 1374 52 45 210 210 Helicobacter pylori strain 25-A-EK9 ['Bacteria', 'Campylobacterota', 'Epsilonproteobacteria', 'Campylobacterales', 'Helicobacteraceae', 'Helicobacter'] PRJNA224116 SAMN10053600 ASM633736v1 GCF_006337365.1
HEPY001.0523.00047 1583343 1 1397 64 45 210 210 Helicobacter pylori strain 21-F-EK1 ['Bacteria', 'Campylobacterota', 'Epsilonproteobacteria', 'Campylobacterales', 'Helicobacteraceae', 'Helicobacter'] PRJNA224116 SAMN10053522 ASM633738v1 GCF_006337385.1
HEPY001.0523.00048 1709363 1 1519 74 45 210 210 Helicobacter pylori strain 19-C-EK1 ['Bacteria', 'Campylobacterota', 'Epsilonproteobacteria', 'Campylobacterales', 'Helicobacteraceae', 'Helicobacter'] PRJNA224116 SAMN10053452 ASM633740v1 GCF_006337405.1
HEPY001.0523.00050 1579431 1 1418 76 45 210 210 Helicobacter pylori strain 381-A-EK4 ['Bacteria', 'Campylobacterota', 'Epsilonproteobacteria', 'Campylobacterales', 'Helicobacteraceae', 'Helicobacter'] PRJNA224116 SAMN10053762 ASM633744v1 GCF_006337445.1
HEPY001.0523.00051 1609437 1 1412 103 45 210 210 Helicobacter pylori strain 280-A-EK1 ['Bacteria', 'Campylobacterota', 'Epsilonproteobacteria', 'Campylobacterales', 'Helicobacteraceae', 'Helicobacter'] PRJNA224116 SAMN10053739 ASM633746v1 GCF_006337465.1
HEPY001.0523.00052 1690720 1 1472 92 45 210 210 Helicobacter pylori strain 173-A-EK1 ['Bacteria', 'Campylobacterota', 'Epsilonproteobacteria', 'Campylobacterales', 'Helicobacteraceae', 'Helicobacter'] PRJNA224116 SAMN10053719 ASM633748v1 GCF_006337485.1
HEPY001.0523.00053 1610187 1 1424 76 45 210 210 Helicobacter pylori strain 169-A-EK5 ['Bacteria', 'Campylobacterota', 'Epsilonproteobacteria', 'Campylobacterales', 'Helicobacteraceae', 'Helicobacter'] PRJNA224116 SAMN10053703 ASM633762v1 GCF_006337625.1
HEPY001.0523.00055 1659500 1 1463 68 45 210 210 Helicobacter pylori strain 24-A-EK1 ['Bacteria', 'Campylobacterota', 'Epsilonproteobacteria', 'Campylobacterales', 'Helicobacteraceae', 'Helicobacter'] PRJNA224116 SAMN10053562 ASM633787v1 GCF_006337875.1
HEPY001.0523.00056 1679162 1 1491 68 45 210 210 Helicobacter pylori strain 23-A-EK1 ['Bacteria', 'Campylobacterota', 'Epsilonproteobacteria', 'Campylobacterales', 'Helicobacteraceae', 'Helicobacter'] PRJNA224116 SAMN10053532 ASM633804v1 GCF_006338045.1
HEPY001.0523.00057 1582133 1 1404 61 45 210 210 Helicobacter pylori strain 21-A-EK1 ['Bacteria', 'Campylobacterota', 'Epsilonproteobacteria', 'Campylobacterales', 'Helicobacteraceae', 'Helicobacter'] PRJNA224116 SAMN10053502 ASM633814v1 GCF_006338145.1
HEPY001.0523.00058 1599658 1 1410 81 45 210 210 Helicobacter pylori strain 20-A-EK1 ['Bacteria', 'Campylobacterota', 'Epsilonproteobacteria', 'Campylobacterales', 'Helicobacteraceae', 'Helicobacter'] PRJNA224116 SAMN10053472 ASM633828v1 GCF_006338285.1
HEPY001.0523.00059 1706376 1 1516 72 45 210 210 Helicobacter pylori strain 19-A-EK3 ['Bacteria', 'Campylobacterota', 'Epsilonproteobacteria', 'Campylobacterales', 'Helicobacteraceae', 'Helicobacter'] PRJNA224116 SAMN10053444 ASM633841v1 GCF_006338415.1
HEPY001.0523.00060 1619300 1 1441 74 45 210 210 Helicobacter pylori strain 13-A-EK8 ['Bacteria', 'Campylobacterota', 'Epsilonproteobacteria', 'Campylobacterales', 'Helicobacteraceae', 'Helicobacter'] PRJNA224116 SAMN10053419 ASM633854v1 GCF_006338545.1
HEPY001.0523.00061 1637783 1 1428 74 45 210 210 Helicobacter pylori strain 479-C2-EK2 ['Bacteria', 'Campylobacterota', 'Epsilonproteobacteria', 'Campylobacterales', 'Helicobacteraceae', 'Helicobacter'] PRJNA224116 SAMN10053865 ASM633868v1 GCF_006338685.1
HEPY001.0523.00062 1601381 1 1424 88 45 210 210 Helicobacter pylori strain 476-C-EK5 ['Bacteria', 'Campylobacterota', 'Epsilonproteobacteria', 'Campylobacterales', 'Helicobacteraceae', 'Helicobacter'] PRJNA224116 SAMN10053808 ASM633881v1 GCF_006338815.1
HEPY001.0523.00063 1638022 1 1433 75 45 210 210 Helicobacter pylori strain 479-C-EK2 ['Bacteria', 'Proteobacteria', 'Epsilonproteobacteria', 'Campylobacterales', 'Helicobacteraceae', 'Helicobacter'] PRJNA224116 SAMN10053855 ASM633894v1 GCF_006338945.1
HEPY001.0523.00064 1628623 1 1441 77 45 210 210 Helicobacter pylori strain 478-A-EK1 ['Bacteria', 'Proteobacteria', 'Epsilonproteobacteria', 'Campylobacterales', 'Helicobacteraceae', 'Helicobacter'] PRJNA224116 SAMN10053819 ASM633906v1 GCF_006339065.1
HEPY001.0523.00065 1540214 1 1380 50 45 210 210 Helicobacter pylori strain 25-A-EK1 ['Bacteria', 'Campylobacterota', 'Epsilonproteobacteria', 'Campylobacterales', 'Helicobacteraceae', 'Helicobacter'] PRJNA224116 SAMN10053592 ASM633923v1 GCF_006339235.1
HEPY001.0523.00066 1596986 1 1408 74 45 210 210 Helicobacter pylori strain 5-A-EK1 ['Bacteria', 'Campylobacterota', 'Epsilonproteobacteria', 'Campylobacterales', 'Helicobacteraceae', 'Helicobacter'] PRJNA224116 SAMN10053382 ASM633936v1 GCF_006339365.1
HEPY001.0523.00067 1681590 2 1469 89 45 210 210 Helicobacter pylori strain B128 7.13 ['Bacteria', 'Campylobacterota', 'Epsilonproteobacteria', 'Campylobacterales', 'Helicobacteraceae', 'Helicobacter'] PRJNA224116 SAMN10083778 ASM779493v1 GCF_007794935.1
HEPY001.0523.00068 1643956 1 1471 63 45 210 210 Helicobacter pylori strain B125A ['Bacteria', 'Campylobacterota', 'Epsilonproteobacteria', 'Campylobacterales', 'Helicobacteraceae', 'Helicobacter'] PRJNA224116 SAMN08055031 ASM783673v1 GCF_007836735.1
HEPY001.0523.00069 1676743 1 1477 76 45 210 210 Helicobacter pylori strain 7.13 ['Bacteria', 'Campylobacterota', 'Epsilonproteobacteria', 'Campylobacterales', 'Helicobacteraceae', 'Helicobacter'] PRJNA224116 SAMN08055030 ASM783685v1 GCF_007836855.1
HEPY001.0523.00071 1648077 1 1479 60 45 210 210 Helicobacter pylori strain J182 ['Bacteria', 'Campylobacterota', 'Epsilonproteobacteria', 'Campylobacterales', 'Helicobacteraceae', 'Helicobacter'] PRJNA224116 SAMN08055036 ASM784371v1 GCF_007843715.1
HEPY001.0523.00072 1726836 1 1516 98 45 210 210 Helicobacter pylori strain B140 ['Bacteria', 'Campylobacterota', 'Epsilonproteobacteria', 'Campylobacterales', 'Helicobacteraceae', 'Helicobacter'] PRJNA224116 SAMN08055035 ASM784391v1 GCF_007843915.1
HEPY001.0523.00073 1663520 1 1458 87 45 210 210 Helicobacter pylori strain B136A ['Bacteria', 'Campylobacterota', 'Epsilonproteobacteria', 'Campylobacterales', 'Helicobacteraceae', 'Helicobacter'] PRJNA224116 SAMN08055034 ASM784403v1 GCF_007844035.1

HEPY001.0523.00074 1646042 1 1438 91 45 210 210 Helicobacter pylori strain B130A ['Bacteria', 'Campylobacterota', 'Epsilonproteobacteria', 'Campylobacterales', 'Helicobacteraceae', 'Helicobacter'] PRJNA224116 SAMN08055033 ASM784405v1 GCF_007844055.1
HEPY001.0523.00075 1604952 1 1419 88 45 210 210 Helicobacter pylori strain TN2wt ['Bacteria', 'Proteobacteria', 'Epsilonproteobacteria', 'Campylobacterales', 'Helicobacteraceae', 'Helicobacter'] PRJNA224116 SAMN02724990 ASM832652v1 GCF_008326525.1
HEPY001.0523.00076 1643850 1 1461 71 42 210 210 Helicobacter pylori strain GCT 27 ['Bacteria', 'Campylobacterota', 'Epsilonproteobacteria', 'Campylobacterales', 'Helicobacteraceae', 'Helicobacter'] PRJNA224116 SAMN13950472 ASM1091715v1 GCF_010917155.1
HEPY001.0523.00077 1642466 1 1441 97 42 210 210 Helicobacter pylori strain GCT 43 ['Bacteria', 'Campylobacterota', 'Epsilonproteobacteria', 'Campylobacterales', 'Helicobacteraceae', 'Helicobacter'] PRJNA224116 SAMN13950473 ASM1091755v1 GCF_010917555.1
HEPY001.0523.00078 1656646 1 1457 91 42 210 210 Helicobacter pylori strain GCT 97 ['Bacteria', 'Campylobacterota', 'Epsilonproteobacteria', 'Campylobacterales', 'Helicobacteraceae', 'Helicobacter'] PRJNA224116 SAMN13950474 ASM1091767v1 GCF_010917675.1
HEPY001.0523.00079 1563305 1 1395 68 45 210 210 Helicobacter pylori strain H1 ['Bacteria', 'Proteobacteria', 'Epsilonproteobacteria', 'Campylobacterales', 'Helicobacteraceae', 'Helicobacter'] PRJNA224116 SAMN10961649 ASM1145627v1 GCF_011456275.1
HEPY001.0523.00080 1563239 1 1402 66 45 210 210 Helicobacter pylori strain 125C7 ['Bacteria', 'Proteobacteria', 'Epsilonproteobacteria', 'Campylobacterales', 'Helicobacteraceae', 'Helicobacter'] PRJNA224116 SAMN10961648 ASM1145629v1 GCF_011456295.1
HEPY001.0523.00081 1563276 1 1402 66 45 210 210 Helicobacter pylori strain 125A3 ['Bacteria', 'Proteobacteria', 'Epsilonproteobacteria', 'Campylobacterales', 'Helicobacteraceae', 'Helicobacter'] PRJNA224116 SAMN10961647 ASM1145631v1 GCF_011456315.1
HEPY001.0523.00082 1563317 1 1390 74 45 210 210 Helicobacter pylori strain 119C10 ['Bacteria', 'Proteobacteria', 'Epsilonproteobacteria', 'Campylobacterales', 'Helicobacteraceae', 'Helicobacter'] PRJNA224116 SAMN10961646 ASM1145647v1 GCF_011456475.1
HEPY001.0523.00083 1563232 1 1395 77 45 210 210 Helicobacter pylori strain 119A2 ['Bacteria', 'Proteobacteria', 'Epsilonproteobacteria', 'Campylobacterales', 'Helicobacteraceae', 'Helicobacter'] PRJNA224116 SAMN10961645 ASM1145666v1 GCF_011456665.1
HEPY001.0523.00084 1566721 1 1396 72 45 210 210 Helicobacter pylori strain 103C8 ['Bacteria', 'Proteobacteria', 'Epsilonproteobacteria', 'Campylobacterales', 'Helicobacteraceae', 'Helicobacter'] PRJNA224116 SAMN10961644 ASM1145689v1 GCF_011456895.1
HEPY001.0523.00085 1563266 1 1399 69 45 210 210 Helicobacter pylori strain 103A4 ['Bacteria', 'Proteobacteria', 'Epsilonproteobacteria', 'Campylobacterales', 'Helicobacteraceae', 'Helicobacter'] PRJNA224116 SAMN10961643 ASM1145711v1 GCF_011457115.1
HEPY001.0523.00086 1563411 1 1396 69 45 210 210 Helicobacter pylori strain 87C7 ['Bacteria', 'Proteobacteria', 'Epsilonproteobacteria', 'Campylobacterales', 'Helicobacteraceae', 'Helicobacter'] PRJNA224116 SAMN10961642 ASM1145735v1 GCF_011457355.1
HEPY001.0523.00087 1563302 1 1395 69 45 210 210 Helicobacter pylori strain 87A3 ['Bacteria', 'Proteobacteria', 'Epsilonproteobacteria', 'Campylobacterales', 'Helicobacteraceae', 'Helicobacter'] PRJNA224116 SAMN10961641 ASM1145761v1 GCF_011457615.1
HEPY001.0523.00088 1563438 1 1401 69 45 210 210 Helicobacter pylori strain 81C9 ['Bacteria', 'Proteobacteria', 'Epsilonproteobacteria', 'Campylobacterales', 'Helicobacteraceae', 'Helicobacter'] PRJNA224116 SAMN10961640 ASM1145781v1 GCF_011457815.1
HEPY001.0523.00089 1563405 1 1397 68 45 210 210 Helicobacter pylori strain 81A1 ['Bacteria', 'Proteobacteria', 'Epsilonproteobacteria', 'Campylobacterales', 'Helicobacteraceae', 'Helicobacter'] PRJNA224116 SAMN10961639 ASM1145801v1 GCF_011458015.1
HEPY001.0523.00090 1563292 1 1386 81 45 210 210 Helicobacter pylori strain 78C8 ['Bacteria', 'Proteobacteria', 'Epsilonproteobacteria', 'Campylobacterales', 'Helicobacteraceae', 'Helicobacter'] PRJNA224116 SAMN10961638 ASM1145809v1 GCF_011458095.1
HEPY001.0523.00091 1563274 1 1391 75 45 210 210 Helicobacter pylori strain 78A3 ['Bacteria', 'Proteobacteria', 'Epsilonproteobacteria', 'Campylobacterales', 'Helicobacteraceae', 'Helicobacter'] PRJNA224116 SAMN10961637 ASM1145831v1 GCF_011458315.1
HEPY001.0523.00092 1563290 1 1399 66 45 210 210 Helicobacter pylori strain 48C8 ['Bacteria', 'Proteobacteria', 'Epsilonproteobacteria', 'Campylobacterales', 'Helicobacteraceae', 'Helicobacter'] PRJNA224116 SAMN10961636 ASM1146221v1 GCF_011462215.1
HEPY001.0523.00093 1563280 1 1405 67 45 210 210 Helicobacter pylori strain 48A2 ['Bacteria', 'Proteobacteria', 'Epsilonproteobacteria', 'Campylobacterales', 'Helicobacteraceae', 'Helicobacter'] PRJNA224116 SAMN10961635 ASM1146237v1 GCF_011462375.1
HEPY001.0523.00095 1563333 1 1390 74 45 210 210 Helicobacter pylori strain 29A2 ['Bacteria', 'Proteobacteria', 'Epsilonproteobacteria', 'Campylobacterales', 'Helicobacteraceae', 'Helicobacter'] PRJNA224116 SAMN10961633 ASM1146273v1 GCF_011462735.1
HEPY001.0523.00096 1563409 1 1398 70 45 210 210 Helicobacter pylori strain 12C8 ['Bacteria', 'Proteobacteria', 'Epsilonproteobacteria', 'Campylobacterales', 'Helicobacteraceae', 'Helicobacter'] PRJNA224116 SAMN10961632 ASM1146291v1 GCF_011462915.1
HEPY001.0523.00097 1563420 1 1394 71 45 210 210 Helicobacter pylori strain 12A3 ['Bacteria', 'Proteobacteria', 'Epsilonproteobacteria', 'Campylobacterales', 'Helicobacteraceae', 'Helicobacter'] PRJNA224116 SAMN10961631 ASM1146303v1 GCF_011463035.1
HEPY001.0523.00098 1563260 1 1398 67 45 210 210 Helicobacter pylori strain 8C10 ['Bacteria', 'Proteobacteria', 'Epsilonproteobacteria', 'Campylobacterales', 'Helicobacteraceae', 'Helicobacter'] PRJNA224116 SAMN10961630 ASM1146305v1 GCF_011463055.1
HEPY001.0523.00099 1563267 1 1406 66 45 210 210 Helicobacter pylori strain 8A3 ['Bacteria', 'Proteobacteria', 'Epsilonproteobacteria', 'Campylobacterales', 'Helicobacteraceae', 'Helicobacter'] PRJNA224116 SAMN10961629 ASM1146317v1 GCF_011463175.1
HEPY001.0523.00100 1673425 2 1466 84 45 210 210 Helicobacter pylori strain HP9192 ['Bacteria', 'Proteobacteria', 'Epsilonproteobacteria', 'Campylobacterales', 'Helicobacteraceae', 'Helicobacter'] PRJNA224116 SAMN14820694 ASM1312203v1 GCF_013122035.1
HEPY001.0523.00101 1674768 2 1470 85 45 210 210 Helicobacter pylori strain HP1352 ['Bacteria', 'Proteobacteria', 'Epsilonproteobacteria', 'Campylobacterales', 'Helicobacteraceae', 'Helicobacter'] PRJNA224116 SAMN14820693 ASM1312205v1 GCF_013122055.1
HEPY001.0523.00102 1674805 2 1471 82 45 210 210 Helicobacter pylori strain HPY ['Bacteria', 'Proteobacteria', 'Epsilonproteobacteria', 'Campylobacterales', 'Helicobacteraceae', 'Helicobacter'] PRJNA224116 SAMN14820692 ASM1312211v1 GCF_013122115.1
HEPY001.0523.00103 1616437 1 1451 66 45 210 210 Helicobacter pylori strain ASHA-001 ['Bacteria', 'Campylobacterota', 'Epsilonproteobacteria', 'Campylobacterales', 'Helicobacteraceae', 'Helicobacter'] PRJNA224116 SAMN14589633 ASM1674799v1 GCF_016747995.1
HEPY001.0523.00104 1640012 1 1476 78 46 210 210 Helicobacter pylori strain ASHA-002 ['Bacteria', 'Campylobacterota', 'Epsilonproteobacteria', 'Campylobacterales', 'Helicobacteraceae', 'Helicobacter'] PRJNA224116 SAMN14589635 ASM1674808v1 GCF_016748085.1
HEPY001.0523.00105 1704243 1 1492 76 45 210 210 Helicobacter pylori strain ASHA-003 ['Bacteria', 'Campylobacterota', 'Epsilonproteobacteria', 'Campylobacterales', 'Helicobacteraceae', 'Helicobacter'] PRJNA224116 SAMN14589656 ASM1674817v1 GCF_016748175.1
HEPY001.0523.00106 1615144 1 1462 80 45 210 210 Helicobacter pylori strain ASHA-004 ['Bacteria', 'Campylobacterota', 'Epsilonproteobacteria', 'Campylobacterales', 'Helicobacteraceae', 'Helicobacter'] PRJNA224116 SAMN14592714 ASM1674826v1 GCF_016748265.1
HEPY001.0523.00107 1630559 1 1446 85 45 210 210 Helicobacter pylori strain ASHA-005 ['Bacteria', 'Campylobacterota', 'Epsilonproteobacteria', 'Campylobacterales', 'Helicobacteraceae', 'Helicobacter'] PRJNA224116 SAMN14592743 ASM1674841v1 GCF_016748415.1
HEPY001.0523.00108 1745797 1 1528 89 45 210 210 Helicobacter pylori strain LIM-010 ['Bacteria', 'Campylobacterota', 'Epsilonproteobacteria', 'Campylobacterales', 'Helicobacteraceae', 'Helicobacter'] PRJNA224116 SAMN14593100 ASM1674855v1 GCF_016748555.1
HEPY001.0523.00109 1618010 1 1434 79 45 210 210 Helicobacter pylori strain LIM-009 ['Bacteria', 'Campylobacterota', 'Epsilonproteobacteria', 'Campylobacterales', 'Helicobacteraceae', 'Helicobacter'] PRJNA224116 SAMN14593099 ASM1674867v1 GCF_016748675.1
HEPY001.0523.00110 1667949 1 1489 65 45 210 210 Helicobacter pylori strain LIM-008 ['Bacteria', 'Campylobacterota', 'Epsilonproteobacteria', 'Campylobacterales', 'Helicobacteraceae', 'Helicobacter'] PRJNA224116 SAMN14593098 ASM1674879v1 GCF_016748795.1
HEPY001.0523.00111 1651043 1 1475 66 45 210 210 Helicobacter pylori strain LIM-007 ['Bacteria', 'Campylobacterota', 'Epsilonproteobacteria', 'Campylobacterales', 'Helicobacteraceae', 'Helicobacter'] PRJNA224116 SAMN14593097 ASM1674892v1 GCF_016748925.1
HEPY001.0523.00112 1745296 1 1527 84 45 210 210 Helicobacter pylori strain LIM-006 ['Bacteria', 'Campylobacterota', 'Epsilonproteobacteria', 'Campylobacterales', 'Helicobacteraceae', 'Helicobacter'] PRJNA224116 SAMN14593096 ASM1674902v1 GCF_016749025.1
HEPY001.0523.00113 1619464 1 1412 70 46 210 210 Helicobacter pylori strain LIM-005 ['Bacteria', 'Campylobacterota', 'Epsilonproteobacteria', 'Campylobacterales', 'Helicobacteraceae', 'Helicobacter'] PRJNA224116 SAMN14593095 ASM1674908v1 GCF_016749085.1
HEPY001.0523.00114 1639295 1 1446 70 45 210 210 Helicobacter pylori strain LIM-003 ['Bacteria', 'Campylobacterota', 'Epsilonproteobacteria', 'Campylobacterales', 'Helicobacteraceae', 'Helicobacter'] PRJNA224116 SAMN14593093 ASM1674921v1 GCF_016749215.1
HEPY001.0523.00115 1638284 1 1467 55 45 210 210 Helicobacter pylori strain LIM-001 ['Bacteria', 'Campylobacterota', 'Epsilonproteobacteria', 'Campylobacterales', 'Helicobacteraceae', 'Helicobacter'] PRJNA224116 SAMN14593091 ASM1674933v1 GCF_016749335.1
HEPY001.0523.00116 1691877 1 1482 69 45 210 210 Helicobacter pylori strain PUNO-010 ['Bacteria', 'Campylobacterota', 'Epsilonproteobacteria', 'Campylobacterales', 'Helicobacteraceae', 'Helicobacter'] PRJNA224116 SAMN14593090 ASM1674945v1 GCF_016749455.1
HEPY001.0523.00117 1629514 1 1454 55 45 210 210 Helicobacter pylori strain PUNO-009 ['Bacteria', 'Campylobacterota', 'Epsilonproteobacteria', 'Campylobacterales', 'Helicobacteraceae', 'Helicobacter'] PRJNA224116 SAMN14593089 ASM1674957v1 GCF_016749575.1
HEPY001.0523.00118 1653624 1 1479 71 45 210 210 Helicobacter pylori strain PUNO-008 ['Bacteria', 'Campylobacterota', 'Epsilonproteobacteria', 'Campylobacterales', 'Helicobacteraceae', 'Helicobacter'] PRJNA224116 SAMN14593088 ASM1674971v1 GCF_016749715.1
HEPY001.0523.00119 1628378 1 1444 55 45 210 210 Helicobacter pylori strain PUNO-007 ['Bacteria', 'Campylobacterota', 'Epsilonproteobacteria', 'Campylobacterales', 'Helicobacteraceae', 'Helicobacter'] PRJNA224116 SAMN14593087 ASM1674981v1 GCF_016749815.1
HEPY001.0523.00120 1614092 1 1442 52 45 210 210 Helicobacter pylori strain PUNO-004 ['Bacteria', 'Campylobacterota', 'Epsilonproteobacteria', 'Campylobacterales', 'Helicobacteraceae', 'Helicobacter'] PRJNA224116 SAMN14593085 ASM1675561v1 GCF_016755615.1
HEPY001.0523.00121 1630535 1 1453 59 45 210 210 Helicobacter pylori strain PUNO-003 ['Bacteria', 'Campylobacterota', 'Epsilonproteobacteria', 'Campylobacterales', 'Helicobacteraceae', 'Helicobacter'] PRJNA224116 SAMN14593084 ASM1675563v1 GCF_016755635.1
HEPY001.0523.00122 1567616 1 1416 57 46 210 210 Helicobacter pylori strain PUNO-002 ['Bacteria', 'Campylobacterota', 'Epsilonproteobacteria', 'Campylobacterales', 'Helicobacteraceae', 'Helicobacter'] PRJNA224116 SAMN14593083 ASM1675565v1 GCF_016755655.1
HEPY001.0523.00123 1644092 1 1462 68 45 210 210 Helicobacter pylori strain PUNO-001 ['Bacteria', 'Campylobacterota', 'Epsilonproteobacteria', 'Campylobacterales', 'Helicobacteraceae', 'Helicobacter'] PRJNA224116 SAMN14593082 ASM1675567v1 GCF_016755675.1
HEPY001.0523.00124 1612861 1 1435 69 46 210 210 Helicobacter pylori strain SHIM-014 ['Bacteria', 'Campylobacterota', 'Epsilonproteobacteria', 'Campylobacterales', 'Helicobacteraceae', 'Helicobacter'] PRJNA224116 SAMN14593081 ASM1675569v1 GCF_016755695.1
HEPY001.0523.00125 1592792 1 1438 64 45 210 210 Helicobacter pylori strain SHIM-007 ['Bacteria', 'Campylobacterota', 'Epsilonproteobacteria', 'Campylobacterales', 'Helicobacteraceae', 'Helicobacter'] PRJNA224116 SAMN14593078 ASM1675571v1 GCF_016755715.1
HEPY001.0523.00126 1675432 1 1505 67 46 210 210 Helicobacter pylori strain SHIM-002 ['Bacteria', 'Campylobacterota', 'Epsilonproteobacteria', 'Campylobacterales', 'Helicobacteraceae', 'Helicobacter'] PRJNA224116 SAMN14593077 ASM1675573v1 GCF_016755735.1
HEPY001.0523.00127 1635086 1 1463 71 45 210 210 Helicobacter pylori strain ASHA-010 ['Bacteria', 'Campylobacterota', 'Epsilonproteobacteria', 'Campylobacterales', 'Helicobacteraceae', 'Helicobacter'] PRJNA224116 SAMN14593076 ASM1675575v1 GCF_016755755.1
HEPY001.0523.00128 1611185 1 1443 76 45 210 210 Helicobacter pylori strain ASHA-009 ['Bacteria', 'Campylobacterota', 'Epsilonproteobacteria', 'Campylobacterales', 'Helicobacteraceae', 'Helicobacter'] PRJNA224116 SAMN14593075 ASM1675577v1 GCF_016755775.1
HEPY001.0523.00129 1627715 1 1449 70 45 210 210 Helicobacter pylori strain ASHA-008 ['Bacteria', 'Campylobacterota', 'Epsilonproteobacteria', 'Campylobacterales', 'Helicobacteraceae', 'Helicobacter'] PRJNA224116 SAMN14593074 ASM1675579v1 GCF_016755795.1
HEPY001.0523.00130 1616651 1 1467 65 45 210 210 Helicobacter pylori strain ASHA-006 ['Bacteria', 'Campylobacterota', 'Epsilonproteobacteria', 'Campylobacterales', 'Helicobacteraceae', 'Helicobacter'] PRJNA224116 SAMN14593073 ASM1675581v1 GCF_016755815.1
HEPY001.0523.00131 1615900 1 1433 69 45 210 210 Helicobacter pylori strain LIM-002 ['Bacteria', 'Campylobacterota', 'Epsilonproteobacteria', 'Campylobacterales', 'Helicobacteraceae', 'Helicobacter'] PRJNA224116 SAMN14593092 ASM1675753v1 GCF_016757535.1
HEPY001.0523.00132 1654099 1 1471 64 46 210 210 Helicobacter pylori strain PUNO-006 ['Bacteria', 'Campylobacterota', 'Epsilonproteobacteria', 'Campylobacterales', 'Helicobacteraceae', 'Helicobacter'] PRJNA224116 SAMN14593086 ASM1675755v1 GCF_016757555.1
HEPY001.0523.00133 1609650 1 1440 69 45 210 210 Helicobacter pylori strain SHIM-011 ['Bacteria', 'Campylobacterota', 'Epsilonproteobacteria', 'Campylobacterales', 'Helicobacteraceae', 'Helicobacter'] PRJNA224116 SAMN14593080 ASM1675757v1 GCF_016757575.1
HEPY001.0523.00134 1675471 1 1510 64 46 210 210 Helicobacter pylori strain SHIM-010 ['Bacteria', 'Campylobacterota', 'Epsilonproteobacteria', 'Campylobacterales', 'Helicobacteraceae', 'Helicobacter'] PRJNA224116 SAMN14593079 ASM1675759v1 GCF_016757595.1
HEPY001.0523.00135 1633985 2 1450 68 45 210 210 Helicobacter pylori strain MT5118 ['Bacteria', 'Proteobacteria', 'Epsilonproteobacteria', 'Campylobacterales', 'Helicobacteraceae', 'Helicobacter'] PRJNA224116 SAMN18350106 ASM1782123v1 GCF_017821235.1
HEPY001.0523.00136 1667928 1 1447 87 45 210 210 Helicobacter pylori strain MT5105 ['Bacteria', 'Proteobacteria', 'Epsilonproteobacteria', 'Campylobacterales', 'Helicobacteraceae', 'Helicobacter'] PRJNA224116 SAMN18350156 ASM1782127v1 GCF_017821275.1
HEPY001.0523.00137 1707147 3 1489 84 45 210 210 Helicobacter pylori strain MT5125 ['Bacteria', 'Proteobacteria', 'Epsilonproteobacteria', 'Campylobacterales', 'Helicobacteraceae', 'Helicobacter'] PRJNA224116 SAMN18350180 ASM1782135v1 GCF_017821355.1
HEPY001.0523.00138 1668381 1 1454 77 45 210 210 Helicobacter pylori strain MT5111 ['Bacteria', 'Campylobacterota', 'Epsilonproteobacteria', 'Campylobacterales', 'Helicobacteraceae', 'Helicobacter'] PRJNA224116 SAMN18350188 ASM1782147v1 GCF_017821475.1
HEPY001.0523.00139 1624458 2 1438 61 45 210 210 Helicobacter pylori strain MT5135 ['Bacteria', 'Campylobacterota', 'Epsilonproteobacteria', 'Campylobacterales', 'Helicobacteraceae', 'Helicobacter'] PRJNA224116 SAMN18350190 ASM1782153v1 GCF_017821535.1
HEPY001.0523.00140 1690568 1 1484 76 45 210 210 Helicobacter pylori strain MT5114 ['Bacteria', 'Campylobacterota', 'Epsilonproteobacteria', 'Campylobacterales', 'Helicobacteraceae', 'Helicobacter'] PRJNA224116 SAMN18350230 ASM1782159v1 GCF_017821595.1
HEPY001.0523.00141 1622324 1 1426 64 45 210 210 Helicobacter pylori strain MT5119 ['Bacteria', 'Proteobacteria', 'Epsilonproteobacteria', 'Campylobacterales', 'Helicobacteraceae', 'Helicobacter'] PRJNA224116 SAMN18350231 ASM1782161v1 GCF_017821615.1
HEPY001.0523.00142 1661144 1 1466 68 45 210 210 Helicobacter pylori strain MT5124 ['Bacteria', 'Proteobacteria', 'Epsilonproteobacteria', 'Campylobacterales', 'Helicobacteraceae', 'Helicobacter'] PRJNA224116 SAMN18350270 ASM1782163v1 GCF_017821635.1
HEPY001.0523.00143 1660636 1 1457 65 45 210 210 Helicobacter pylori strain MT5136 ['Bacteria', 'Campylobacterota', 'Epsilonproteobacteria', 'Campylobacterales', 'Helicobacteraceae', 'Helicobacter'] PRJNA224116 SAMN18350271 ASM1782165v1 GCF_017821655.1
HEPY001.0523.00144 1591853 1 1410 87 45 210 210 Helicobacter pylori strain 3401 ['Bacteria', 'Proteobacteria', 'Epsilonproteobacteria', 'Campylobacterales', 'Helicobacteraceae', 'Helicobacter'] PRJNA224116 SAMD00296812 ASM1967050v1 GCF_019670505.1
HEPY001.0523.00145 1585790 1 1388 96 45 210 210 Helicobacter pylori strain #3 ['Bacteria', 'Proteobacteria', 'Epsilonproteobacteria', 'Campylobacterales', 'Helicobacteraceae', 'Helicobacter'] PRJNA224116 SAMD00392799 ASM1997385v1 GCF_019973855.1
HEPY001.0523.00146 1608283 1 1440 75 45 210 210 Helicobacter pylori strain #5 ['Bacteria', 'Proteobacteria', 'Epsilonproteobacteria', 'Campylobacterales', 'Helicobacteraceae', 'Helicobacter'] PRJNA224116 SAMD00392800 ASM1997387v1 GCF_019973875.1
HEPY001.0523.00147 1630060 1 1444 82 45 210 210 Helicobacter pylori strain #6 ['Bacteria', 'Proteobacteria', 'Epsilonproteobacteria', 'Campylobacterales', 'Helicobacteraceae', 'Helicobacter'] PRJNA224116 SAMD00392801 ASM1997399v1 GCF_019973995.1
HEPY001.0523.00148 1668167 1 1462 118 45 210 210 Helicobacter pylori strain #8 ['Bacteria', 'Proteobacteria', 'Epsilonproteobacteria', 'Campylobacterales', 'Helicobacteraceae', 'Helicobacter'] PRJNA224116 SAMD00392802 ASM1997405v1 GCF_019974055.1
HEPY001.0523.00149 1644102 2 1464 87 45 210 210 Helicobacter pylori strain FDAARGOS_1601 ['Bacteria', 'Proteobacteria', 'Epsilonproteobacteria', 'Campylobacterales', 'Helicobacteraceae', 'Helicobacter'] PRJNA224116 SAMN22365130 ASM2073552v1 GCF_020735525.1
HEPY001.0523.00151 1637585 1 1458 67 45 210 210 Helicobacter pylori strain 3192 ['Bacteria', 'Proteobacteria', 'Epsilonproteobacteria', 'Campylobacterales', 'Helicobacteraceae', 'Helicobacter'] PRJNA224116 SAMN22488069 ASM2090658v1 GCF_020906585.1
HEPY001.0523.00152 1685094 1 1473 69 45 210 210 Helicobacter pylori strain J99 derivative isolate D1 ['Bacteria', 'Proteobacteria', 'Epsilonproteobacteria', 'Campylobacterales', 'Helicobacteraceae', 'Helicobacter'] PRJNA224116 SAMN23610989 ASM2118414v1 GCF_021184145.1
HEPY001.0523.00153 1575789 1 1401 73 45 210 210 Helicobacter pylori strain AL02 ['Bacteria', 'Pseudomonadota', 'Epsilonproteobacteria', 'Campylobacterales', 'Helicobacteraceae', 'Helicobacter'] PRJNA224116 SAMN15338960 ASM2172948v1 GCF_021729485.1
HEPY001.0523.00154 1656897 1 1450 84 45 210 210 Helicobacter pylori strain UBN18 ['Bacteria', 'Pseudomonadota', 'Epsilonproteobacteria', 'Campylobacterales', 'Helicobacteraceae', 'Helicobacter'] PRJNA224116 SAMN15339009 ASM2172950v1 GCF_021729505.1
HEPY001.0523.00155 1639844 1 1457 72 45 210 210 Helicobacter pylori strain NP05-124 ['Bacteria', 'Pseudomonadota', 'Epsilonproteobacteria', 'Campylobacterales', 'Helicobacteraceae', 'Helicobacter'] PRJNA224116 SAMN15339008 ASM2172952v1 GCF_021729525.1
HEPY001.0523.00156 1687334 1 1472 97 45 210 210 Helicobacter pylori strain Hk711 ['Bacteria', 'Pseudomonadota', 'Epsilonproteobacteria', 'Campylobacterales', 'Helicobacteraceae', 'Helicobacter'] PRJNA224116 SAMN15339005 ASM2172960v1 GCF_021729605.1
HEPY001.0523.00157 1619269 1 1459 64 45 210 210 Helicobacter pylori strain BT302 ['Bacteria', 'Pseudomonadota', 'Epsilonproteobacteria', 'Campylobacterales', 'Helicobacteraceae', 'Helicobacter'] PRJNA224116 SAMN15339001 ASM2172968v1 GCF_021729685.1
HEPY001.0523.00158 1639336 1 1468 55 45 210 210 Helicobacter pylori strain AL04 ['Bacteria', 'Pseudomonadota', 'Epsilonproteobacteria', 'Campylobacterales', 'Helicobacteraceae', 'Helicobacter'] PRJNA224116 SAMN15338999 ASM2172972v1 GCF_021729725.1
HEPY001.0523.00159 1617365 1 1420 84 45 210 210 Helicobacter pylori strain K115 ['Bacteria', 'Proteobacteria', 'Epsilonproteobacteria', 'Campylobacterales', 'Helicobacteraceae', 'Helicobacter'] PRJNA224116 SAMN25210003 ASM2201463v1 GCF_022014635.1
HEPY001.0523.00160 1577655 1 1412 80 45 210 210 Helicobacter pylori strain Hpfe127 ['Bacteria', 'Proteobacteria', 'Epsilonproteobacteria', 'Campylobacterales', 'Helicobacteraceae', 'Helicobacter'] PRJNA224116 SAMN26675611 ASM2292117v1 GCF_022921175.1
HEPY001.0523.00161 1627588 2 1439 85 45 210 210 Helicobacter pylori strain Hpfe126 ['Bacteria', 'Proteobacteria', 'Epsilonproteobacteria', 'Campylobacterales', 'Helicobacteraceae', 'Helicobacter'] PRJNA224116 SAMN26675610 ASM2292119v1 GCF_022921195.1
HEPY001.0523.00162 1557058 2 1375 75 45 210 210 Helicobacter pylori strain Hpfe125 ['Bacteria', 'Proteobacteria', 'Epsilonproteobacteria', 'Campylobacterales', 'Helicobacteraceae', 'Helicobacter'] PRJNA224116 SAMN26675609 ASM2292121v1 GCF_022921215.1
HEPY001.0523.00163 1552368 2 1394 65 45 210 210 Helicobacter pylori strain Hpfe124 ['Bacteria', 'Proteobacteria', 'Epsilonproteobacteria', 'Campylobacterales', 'Helicobacteraceae', 'Helicobacter'] PRJNA224116 SAMN26675608 ASM2292123v1 GCF_022921235.1
HEPY001.0523.00165 1563646 2 1395 67 45 210 210 Helicobacter pylori strain Hpfe122 ['Bacteria', 'Proteobacteria', 'Epsilonproteobacteria', 'Campylobacterales', 'Helicobacteraceae', 'Helicobacter'] PRJNA224116 SAMN26675606 ASM2292127v1 GCF_022921275.1
HEPY001.0523.00166 1609748 1 1415 79 45 210 210 Helicobacter pylori strain Hpfe120 ['Bacteria', 'Proteobacteria', 'Epsilonproteobacteria', 'Campylobacterales', 'Helicobacteraceae', 'Helicobacter'] PRJNA224116 SAMN26675605 ASM2292129v1 GCF_022921295.1
HEPY001.0523.00167 1637345 1 1432 88 45 210 210 Helicobacter pylori strain Hpfe118 ['Bacteria', 'Proteobacteria', 'Epsilonproteobacteria', 'Campylobacterales', 'Helicobacteraceae', 'Helicobacter'] PRJNA224116 SAMN26675604 ASM2292131v1 GCF_022921315.1
HEPY001.0523.00168 1618823 1 1439 85 45 210 210 Helicobacter pylori strain Hpfe115 ['Bacteria', 'Proteobacteria', 'Epsilonproteobacteria', 'Campylobacterales', 'Helicobacteraceae', 'Helicobacter'] PRJNA224116 SAMN26675603 ASM2292133v1 GCF_022921335.1
HEPY001.0523.00169 1557498 1 1388 79 45 210 210 Helicobacter pylori strain Hpfe114 ['Bacteria', 'Proteobacteria', 'Epsilonproteobacteria', 'Campylobacterales', 'Helicobacteraceae', 'Helicobacter'] PRJNA224116 SAMN26675602 ASM2292135v1 GCF_022921355.1
HEPY001.0523.00170 1644007 1 1450 85 45 210 210 Helicobacter pylori strain Hpfe112 ['Bacteria', 'Proteobacteria', 'Epsilonproteobacteria', 'Campylobacterales', 'Helicobacteraceae', 'Helicobacter'] PRJNA224116 SAMN26675600 ASM2292157v1 GCF_022921575.1
HEPY001.0523.00171 1664142 1 1488 86 45 210 210 Helicobacter pylori strain Hpfe110 ['Bacteria', 'Proteobacteria', 'Epsilonproteobacteria', 'Campylobacterales', 'Helicobacteraceae', 'Helicobacter'] PRJNA224116 SAMN26675599 ASM2292169v1 GCF_022921695.1
HEPY001.0523.00172 1581972 3 1408 87 45 210 210 Helicobacter pylori strain Hpfe108 ['Bacteria', 'Proteobacteria', 'Epsilonproteobacteria', 'Campylobacterales', 'Helicobacteraceae', 'Helicobacter'] PRJNA224116 SAMN26675597 ASM2292175v1 GCF_022921755.1
HEPY001.0523.00173 1620835 1 1419 93 45 210 210 Helicobacter pylori strain Hpfe107 ['Bacteria', 'Proteobacteria', 'Epsilonproteobacteria', 'Campylobacterales', 'Helicobacteraceae', 'Helicobacter'] PRJNA224116 SAMN26675596 ASM2292177v1 GCF_022921775.1

HEPY001.0523.00174 1594453 2 1411 76 45 210 210 Helicobacter pylori strain Hpfe106 ['Bacteria', 'Proteobacteria', 'Epsilonproteobacteria', 'Campylobacterales', 'Helicobacteraceae', 'Helicobacter'] PRJNA224116 SAMN26675595 ASM2292179v1 GCF_022921795.1
HEPY001.0523.00175 1574424 1 1366 90 45 210 210 Helicobacter pylori strain Hpfe104 ['Bacteria', 'Proteobacteria', 'Epsilonproteobacteria', 'Campylobacterales', 'Helicobacteraceae', 'Helicobacter'] PRJNA224116 SAMN26675593 ASM2292181v1 GCF_022921815.1
HEPY001.0523.00176 1602592 2 1431 64 45 210 210 Helicobacter pylori strain Hpfe103 ['Bacteria', 'Proteobacteria', 'Epsilonproteobacteria', 'Campylobacterales', 'Helicobacteraceae', 'Helicobacter'] PRJNA224116 SAMN26675592 ASM2292183v1 GCF_022921835.1
HEPY001.0523.00177 1616207 2 1419 76 45 210 210 Helicobacter pylori strain Hpfe102 ['Bacteria', 'Proteobacteria', 'Epsilonproteobacteria', 'Campylobacterales', 'Helicobacteraceae', 'Helicobacter'] PRJNA224116 SAMN26675591 ASM2292185v1 GCF_022921855.1
HEPY001.0523.00178 1572519 1 1402 71 45 210 210 Helicobacter pylori strain Hpfe101 ['Bacteria', 'Proteobacteria', 'Epsilonproteobacteria', 'Campylobacterales', 'Helicobacteraceae', 'Helicobacter'] PRJNA224116 SAMN26675590 ASM2292187v1 GCF_022921875.1
HEPY001.0523.00179 1555854 1 1371 79 45 210 210 Helicobacter pylori strain Hpfe100 ['Bacteria', 'Proteobacteria', 'Epsilonproteobacteria', 'Campylobacterales', 'Helicobacteraceae', 'Helicobacter'] PRJNA224116 SAMN26675589 ASM2292189v1 GCF_022921895.1
HEPY001.0523.00180 1649450 2 1471 77 45 210 210 Helicobacter pylori strain Hpfe099 ['Bacteria', 'Proteobacteria', 'Epsilonproteobacteria', 'Campylobacterales', 'Helicobacteraceae', 'Helicobacter'] PRJNA224116 SAMN26675588 ASM2292191v1 GCF_022921915.1
HEPY001.0523.00181 1640211 1 1443 77 45 210 210 Helicobacter pylori strain Hpfe097 ['Bacteria', 'Proteobacteria', 'Epsilonproteobacteria', 'Campylobacterales', 'Helicobacteraceae', 'Helicobacter'] PRJNA224116 SAMN26675586 ASM2292193v1 GCF_022921935.1
HEPY001.0523.00182 1564753 2 1401 78 45 210 210 Helicobacter pylori strain Hpfe096 ['Bacteria', 'Proteobacteria', 'Epsilonproteobacteria', 'Campylobacterales', 'Helicobacteraceae', 'Helicobacter'] PRJNA224116 SAMN26675585 ASM2292195v1 GCF_022921955.1
HEPY001.0523.00183 1603559 2 1417 72 45 210 210 Helicobacter pylori strain Hpfe095 ['Bacteria', 'Proteobacteria', 'Epsilonproteobacteria', 'Campylobacterales', 'Helicobacteraceae', 'Helicobacter'] PRJNA224116 SAMN26675584 ASM2292197v1 GCF_022921975.1
HEPY001.0523.00184 1604124 1 1422 77 45 210 210 Helicobacter pylori strain Hpfe093 ['Bacteria', 'Proteobacteria', 'Epsilonproteobacteria', 'Campylobacterales', 'Helicobacteraceae', 'Helicobacter'] PRJNA224116 SAMN26675582 ASM2292199v1 GCF_022921995.1
HEPY001.0523.00185 1624101 2 1443 78 45 210 210 Helicobacter pylori strain Hpfe092 ['Bacteria', 'Proteobacteria', 'Epsilonproteobacteria', 'Campylobacterales', 'Helicobacteraceae', 'Helicobacter'] PRJNA224116 SAMN26675581 ASM2292201v1 GCF_022922015.1
HEPY001.0523.00186 1625567 1 1443 73 45 210 210 Helicobacter pylori strain Hpfe089 ['Bacteria', 'Proteobacteria', 'Epsilonproteobacteria', 'Campylobacterales', 'Helicobacteraceae', 'Helicobacter'] PRJNA224116 SAMN26675579 ASM2292203v1 GCF_022922035.1
HEPY001.0523.00188 1588502 1 1409 75 45 210 210 Helicobacter pylori strain Hpfe086 ['Bacteria', 'Proteobacteria', 'Epsilonproteobacteria', 'Campylobacterales', 'Helicobacteraceae', 'Helicobacter'] PRJNA224116 SAMN26675576 ASM2292217v1 GCF_022922175.1
HEPY001.0523.00189 1574428 1 1385 78 45 210 210 Helicobacter pylori strain Hpfe085 ['Bacteria', 'Proteobacteria', 'Epsilonproteobacteria', 'Campylobacterales', 'Helicobacteraceae', 'Helicobacter'] PRJNA224116 SAMN26675575 ASM2292229v1 GCF_022922295.1
HEPY001.0523.00190 1629886 1 1447 88 45 210 210 Helicobacter pylori strain Hpfe084 ['Bacteria', 'Proteobacteria', 'Epsilonproteobacteria', 'Campylobacterales', 'Helicobacteraceae', 'Helicobacter'] PRJNA224116 SAMN26675574 ASM2292241v1 GCF_022922415.1
HEPY001.0523.00191 1577172 1 1403 64 45 210 210 Helicobacter pylori strain Hpfe083 ['Bacteria', 'Proteobacteria', 'Epsilonproteobacteria', 'Campylobacterales', 'Helicobacteraceae', 'Helicobacter'] PRJNA224116 SAMN26675573 ASM2292251v1 GCF_022922515.1
HEPY001.0523.00192 1617045 2 1423 75 45 210 210 Helicobacter pylori strain Hpfe082 ['Bacteria', 'Proteobacteria', 'Epsilonproteobacteria', 'Campylobacterales', 'Helicobacteraceae', 'Helicobacter'] PRJNA224116 SAMN26675572 ASM2292265v1 GCF_022922655.1
HEPY001.0523.00193 1573286 1 1387 79 45 210 210 Helicobacter pylori strain Hpfe081 ['Bacteria', 'Proteobacteria', 'Epsilonproteobacteria', 'Campylobacterales', 'Helicobacteraceae', 'Helicobacter'] PRJNA224116 SAMN26675571 ASM2292277v1 GCF_022922775.1
HEPY001.0523.00194 1566531 2 1384 71 45 210 210 Helicobacter pylori strain Hpfe080 ['Bacteria', 'Proteobacteria', 'Epsilonproteobacteria', 'Campylobacterales', 'Helicobacteraceae', 'Helicobacter'] PRJNA224116 SAMN26675570 ASM2292291v1 GCF_022922915.1
HEPY001.0523.00195 1622213 1 1410 101 45 210 210 Helicobacter pylori strain Hpfe079 ['Bacteria', 'Proteobacteria', 'Epsilonproteobacteria', 'Campylobacterales', 'Helicobacteraceae', 'Helicobacter'] PRJNA224116 SAMN26675569 ASM2292295v1 GCF_022922955.1
HEPY001.0523.00196 1609466 1 1416 84 45 210 210 Helicobacter pylori strain Hpfe078 ['Bacteria', 'Proteobacteria', 'Epsilonproteobacteria', 'Campylobacterales', 'Helicobacteraceae', 'Helicobacter'] PRJNA224116 SAMN26675568 ASM2292297v1 GCF_022922975.1
HEPY001.0523.00197 1573039 1 1376 74 45 210 210 Helicobacter pylori strain Hpfe076 ['Bacteria', 'Proteobacteria', 'Epsilonproteobacteria', 'Campylobacterales', 'Helicobacteraceae', 'Helicobacter'] PRJNA224116 SAMN26675566 ASM2292301v1 GCF_022923015.1
HEPY001.0523.00198 1652404 1 1443 85 45 210 210 Helicobacter pylori strain Hpfe075 ['Bacteria', 'Proteobacteria', 'Epsilonproteobacteria', 'Campylobacterales', 'Helicobacteraceae', 'Helicobacter'] PRJNA224116 SAMN26675565 ASM2292303v1 GCF_022923035.1
HEPY001.0523.00199 1602783 1 1409 73 45 210 210 Helicobacter pylori strain Hpfe074 ['Bacteria', 'Proteobacteria', 'Epsilonproteobacteria', 'Campylobacterales', 'Helicobacteraceae', 'Helicobacter'] PRJNA224116 SAMN26675564 ASM2292305v1 GCF_022923055.1
HEPY001.0523.00200 1610658 1 1405 72 45 210 210 Helicobacter pylori strain Hpfe072 ['Bacteria', 'Proteobacteria', 'Epsilonproteobacteria', 'Campylobacterales', 'Helicobacteraceae', 'Helicobacter'] PRJNA224116 SAMN26675562 ASM2292309v1 GCF_022923095.1
HEPY001.0523.00201 1618643 1 1429 70 45 210 210 Helicobacter pylori strain Hpfe071 ['Bacteria', 'Proteobacteria', 'Epsilonproteobacteria', 'Campylobacterales', 'Helicobacteraceae', 'Helicobacter'] PRJNA224116 SAMN26675561 ASM2292311v1 GCF_022923115.1
HEPY001.0523.00202 1583533 2 1424 56 45 210 210 Helicobacter pylori strain Hpfe070 ['Bacteria', 'Proteobacteria', 'Epsilonproteobacteria', 'Campylobacterales', 'Helicobacteraceae', 'Helicobacter'] PRJNA224116 SAMN26675560 ASM2292313v1 GCF_022923135.1
HEPY001.0523.00203 1583621 2 1427 52 45 210 210 Helicobacter pylori strain Hpfe069 ['Bacteria', 'Proteobacteria', 'Epsilonproteobacteria', 'Campylobacterales', 'Helicobacteraceae', 'Helicobacter'] PRJNA224116 SAMN26675559 ASM2292315v1 GCF_022923155.1
HEPY001.0523.00204 1566753 1 1371 78 45 210 210 Helicobacter pylori strain Hpfe068 ['Bacteria', 'Proteobacteria', 'Epsilonproteobacteria', 'Campylobacterales', 'Helicobacteraceae', 'Helicobacter'] PRJNA224116 SAMN26675558 ASM2292327v1 GCF_022923275.1
HEPY001.0523.00205 1586299 1 1394 92 45 210 210 Helicobacter pylori strain Hpfe066 ['Bacteria', 'Proteobacteria', 'Epsilonproteobacteria', 'Campylobacterales', 'Helicobacteraceae', 'Helicobacter'] PRJNA224116 SAMN26675556 ASM2292339v1 GCF_022923395.1
HEPY001.0523.00207 1615760 1 1424 79 45 210 210 Helicobacter pylori strain Hpfe064 ['Bacteria', 'Proteobacteria', 'Epsilonproteobacteria', 'Campylobacterales', 'Helicobacteraceae', 'Helicobacter'] PRJNA224116 SAMN26675554 ASM2292355v1 GCF_022923555.1
HEPY001.0523.00208 1608340 2 1422 82 45 210 210 Helicobacter pylori strain Hpfe063 ['Bacteria', 'Proteobacteria', 'Epsilonproteobacteria', 'Campylobacterales', 'Helicobacteraceae', 'Helicobacter'] PRJNA224116 SAMN26675553 ASM2292357v1 GCF_022923575.1
HEPY001.0523.00209 1560534 1 1389 71 45 210 210 Helicobacter pylori strain Hpfe062 ['Bacteria', 'Proteobacteria', 'Epsilonproteobacteria', 'Campylobacterales', 'Helicobacteraceae', 'Helicobacter'] PRJNA224116 SAMN26675552 ASM2292359v1 GCF_022923595.1
HEPY001.0523.00210 1613185 1 1427 75 45 210 210 Helicobacter pylori strain Hpfe061 ['Bacteria', 'Proteobacteria', 'Epsilonproteobacteria', 'Campylobacterales', 'Helicobacteraceae', 'Helicobacter'] PRJNA224116 SAMN26675551 ASM2292361v1 GCF_022923615.1
HEPY001.0523.00211 1576166 1 1411 79 45 210 210 Helicobacter pylori strain Hpfe060 ['Bacteria', 'Proteobacteria', 'Epsilonproteobacteria', 'Campylobacterales', 'Helicobacteraceae', 'Helicobacter'] PRJNA224116 SAMN26675550 ASM2292363v1 GCF_022923635.1
HEPY001.0523.00212 1588133 1 1396 76 45 210 210 Helicobacter pylori strain Hpfe059 ['Bacteria', 'Proteobacteria', 'Epsilonproteobacteria', 'Campylobacterales', 'Helicobacteraceae', 'Helicobacter'] PRJNA224116 SAMN26675549 ASM2292365v1 GCF_022923655.1
HEPY001.0523.00213 1625426 2 1450 76 45 210 210 Helicobacter pylori strain Hpfe058 ['Bacteria', 'Proteobacteria', 'Epsilonproteobacteria', 'Campylobacterales', 'Helicobacteraceae', 'Helicobacter'] PRJNA224116 SAMN26675548 ASM2292367v1 GCF_022923675.1
HEPY001.0523.00214 1580664 2 1395 83 45 210 210 Helicobacter pylori strain Hpfe057 ['Bacteria', 'Proteobacteria', 'Epsilonproteobacteria', 'Campylobacterales', 'Helicobacteraceae', 'Helicobacter'] PRJNA224116 SAMN26675547 ASM2292369v1 GCF_022923695.1
HEPY001.0523.00215 1659860 2 1448 96 45 210 210 Helicobacter pylori strain Hpfe056 ['Bacteria', 'Proteobacteria', 'Epsilonproteobacteria', 'Campylobacterales', 'Helicobacteraceae', 'Helicobacter'] PRJNA224116 SAMN26675546 ASM2292377v1 GCF_022923775.1
HEPY001.0523.00216 1572908 1 1390 77 45 210 210 Helicobacter pylori strain Hpfe055 ['Bacteria', 'Proteobacteria', 'Epsilonproteobacteria', 'Campylobacterales', 'Helicobacteraceae', 'Helicobacter'] PRJNA224116 SAMN26675545 ASM2292391v1 GCF_022923915.1
HEPY001.0523.00217 1563078 1 1399 64 45 210 210 Helicobacter pylori strain Hpfe054 ['Bacteria', 'Proteobacteria', 'Epsilonproteobacteria', 'Campylobacterales', 'Helicobacteraceae', 'Helicobacter'] PRJNA224116 SAMN26675544 ASM2292403v1 GCF_022924035.1
HEPY001.0523.00218 1617070 1 1426 92 45 210 210 Helicobacter pylori strain Hpfe053 ['Bacteria', 'Proteobacteria', 'Epsilonproteobacteria', 'Campylobacterales', 'Helicobacteraceae', 'Helicobacter'] PRJNA224116 SAMN26675543 ASM2292417v1 GCF_022924175.1
HEPY001.0523.00219 1560058 1 1384 63 45 210 210 Helicobacter pylori strain Hpfe052 ['Bacteria', 'Proteobacteria', 'Epsilonproteobacteria', 'Campylobacterales', 'Helicobacteraceae', 'Helicobacter'] PRJNA224116 SAMN26675542 ASM2292427v1 GCF_022924275.1
HEPY001.0523.00220 1616176 1 1432 74 45 210 210 Helicobacter pylori strain Hpfe051 ['Bacteria', 'Proteobacteria', 'Epsilonproteobacteria', 'Campylobacterales', 'Helicobacteraceae', 'Helicobacter'] PRJNA224116 SAMN26675541 ASM2292435v1 GCF_022924355.1
HEPY001.0523.00221 1613080 1 1434 69 45 210 210 Helicobacter pylori strain Hpfe049 ['Bacteria', 'Proteobacteria', 'Epsilonproteobacteria', 'Campylobacterales', 'Helicobacteraceae', 'Helicobacter'] PRJNA224116 SAMN26675540 ASM2292437v1 GCF_022924375.1
HEPY001.0523.00222 1613194 1 1426 74 45 210 210 Helicobacter pylori strain Hpfe048 ['Bacteria', 'Proteobacteria', 'Epsilonproteobacteria', 'Campylobacterales', 'Helicobacteraceae', 'Helicobacter'] PRJNA224116 SAMN26675539 ASM2292439v1 GCF_022924395.1
HEPY001.0523.00223 1566721 1 1409 63 45 210 210 Helicobacter pylori strain Hpfe040 ['Bacteria', 'Proteobacteria', 'Epsilonproteobacteria', 'Campylobacterales', 'Helicobacteraceae', 'Helicobacter'] PRJNA224116 SAMN26675538 ASM2292441v1 GCF_022924415.1
HEPY001.0523.00224 1626226 1 1435 75 45 210 210 Helicobacter pylori strain Hpfe039 ['Bacteria', 'Proteobacteria', 'Epsilonproteobacteria', 'Campylobacterales', 'Helicobacteraceae', 'Helicobacter'] PRJNA224116 SAMN26675537 ASM2292443v1 GCF_022924435.1
HEPY001.0523.00225 1576312 1 1410 65 45 210 210 Helicobacter pylori strain Hpfe038 ['Bacteria', 'Proteobacteria', 'Epsilonproteobacteria', 'Campylobacterales', 'Helicobacteraceae', 'Helicobacter'] PRJNA224116 SAMN26675536 ASM2292445v1 GCF_022924455.1
HEPY001.0523.00226 1596715 1 1399 87 45 210 210 Helicobacter pylori strain Hpfe037 ['Bacteria', 'Proteobacteria', 'Epsilonproteobacteria', 'Campylobacterales', 'Helicobacteraceae', 'Helicobacter'] PRJNA224116 SAMN26675535 ASM2292447v1 GCF_022924475.1
HEPY001.0523.00227 1561930 1 1405 58 45 210 210 Helicobacter pylori strain Hpfe036 ['Bacteria', 'Proteobacteria', 'Epsilonproteobacteria', 'Campylobacterales', 'Helicobacteraceae', 'Helicobacter'] PRJNA224116 SAMN26675534 ASM2292449v1 GCF_022924495.1
HEPY001.0523.00228 1597939 2 1396 81 45 210 210 Helicobacter pylori strain Hpfe035 ['Bacteria', 'Proteobacteria', 'Epsilonproteobacteria', 'Campylobacterales', 'Helicobacteraceae', 'Helicobacter'] PRJNA224116 SAMN26675533 ASM2292451v1 GCF_022924515.1
HEPY001.0523.00229 1559352 1 1393 66 45 210 210 Helicobacter pylori strain Hpfe033 ['Bacteria', 'Proteobacteria', 'Epsilonproteobacteria', 'Campylobacterales', 'Helicobacteraceae', 'Helicobacter'] PRJNA224116 SAMN26675532 ASM2292453v1 GCF_022924535.1
HEPY001.0523.00230 1626209 1 1420 71 45 210 210 Helicobacter pylori strain Hpfe032 ['Bacteria', 'Proteobacteria', 'Epsilonproteobacteria', 'Campylobacterales', 'Helicobacteraceae', 'Helicobacter'] PRJNA224116 SAMN26675531 ASM2292455v1 GCF_022924555.1
HEPY001.0523.00233 1581642 1 1405 65 45 210 210 Helicobacter pylori strain Hpfe027 ['Bacteria', 'Proteobacteria', 'Epsilonproteobacteria', 'Campylobacterales', 'Helicobacteraceae', 'Helicobacter'] PRJNA224116 SAMN26675528 ASM2292473v1 GCF_022924735.1
HEPY001.0523.00234 1595545 1 1410 81 45 210 210 Helicobacter pylori strain Hpfe024 ['Bacteria', 'Proteobacteria', 'Epsilonproteobacteria', 'Campylobacterales', 'Helicobacteraceae', 'Helicobacter'] PRJNA224116 SAMN26675527 ASM2292487v1 GCF_022924875.1
HEPY001.0523.00235 1608056 1 1423 74 45 210 210 Helicobacter pylori strain Hpfe023 ['Bacteria', 'Proteobacteria', 'Epsilonproteobacteria', 'Campylobacterales', 'Helicobacteraceae', 'Helicobacter'] PRJNA224116 SAMN26675526 ASM2292499v1 GCF_022924995.1
HEPY001.0523.00236 1617425 1 1423 73 45 210 210 Helicobacter pylori strain Hpfe022 ['Bacteria', 'Proteobacteria', 'Epsilonproteobacteria', 'Campylobacterales', 'Helicobacteraceae', 'Helicobacter'] PRJNA224116 SAMN26675525 ASM2292517v1 GCF_022925175.1
HEPY001.0523.00237 1662094 2 1442 92 45 210 210 Helicobacter pylori strain Hpfe019 ['Bacteria', 'Proteobacteria', 'Epsilonproteobacteria', 'Campylobacterales', 'Helicobacteraceae', 'Helicobacter'] PRJNA224116 SAMN26675524 ASM2292521v1 GCF_022925215.1
HEPY001.0523.00238 1586095 3 1410 84 45 210 210 Helicobacter pylori strain Hpfe010 ['Bacteria', 'Proteobacteria', 'Epsilonproteobacteria', 'Campylobacterales', 'Helicobacteraceae', 'Helicobacter'] PRJNA224116 SAMN26675523 ASM2292525v1 GCF_022925255.1
HEPY001.0523.00239 1595694 1 1428 60 45 210 210 Helicobacter pylori strain Hpfe005 ['Bacteria', 'Proteobacteria', 'Epsilonproteobacteria', 'Campylobacterales', 'Helicobacteraceae', 'Helicobacter'] PRJNA224116 SAMN26675521 ASM2292527v1 GCF_022925275.1
HEPY001.0523.00240 1620723 2 1426 70 45 210 210 Helicobacter pylori strain Hpfe0021 ['Bacteria', 'Proteobacteria', 'Epsilonproteobacteria', 'Campylobacterales', 'Helicobacteraceae', 'Helicobacter'] PRJNA224116 SAMN26675520 ASM2292529v1 GCF_022925295.1
HEPY001.0523.00241 1576980 1 1399 66 45 210 210 Helicobacter pylori strain Hpfe0020 ['Bacteria', 'Proteobacteria', 'Epsilonproteobacteria', 'Campylobacterales', 'Helicobacteraceae', 'Helicobacter'] PRJNA224116 SAMN26675519 ASM2292541v1 GCF_022925415.1
HEPY001.0523.00242 1564699 1 1397 72 45 210 210 Helicobacter pylori strain Hpfe0016 ['Bacteria', 'Proteobacteria', 'Epsilonproteobacteria', 'Campylobacterales', 'Helicobacteraceae', 'Helicobacter'] PRJNA224116 SAMN26675518 ASM2292557v1 GCF_022925575.1
HEPY001.0523.00243 1569162 2 1381 74 45 210 210 Helicobacter pylori strain Hpfe0015 ['Bacteria', 'Proteobacteria', 'Epsilonproteobacteria', 'Campylobacterales', 'Helicobacteraceae', 'Helicobacter'] PRJNA224116 SAMN26675517 ASM2292567v1 GCF_022925675.1
HEPY001.0523.00244 1613739 1 1416 93 45 210 210 Helicobacter pylori strain Hpfe0013 ['Bacteria', 'Proteobacteria', 'Epsilonproteobacteria', 'Campylobacterales', 'Helicobacteraceae', 'Helicobacter'] PRJNA224116 SAMN26675516 ASM2292575v1 GCF_022925755.1
HEPY001.0523.00245 1627796 1 1427 81 45 210 210 Helicobacter pylori strain Hpfe0012 ['Bacteria', 'Proteobacteria', 'Epsilonproteobacteria', 'Campylobacterales', 'Helicobacteraceae', 'Helicobacter'] PRJNA224116 SAMN26675515 ASM2292577v1 GCF_022925775.1
HEPY001.0523.00246 1628951 2 1433 85 45 210 210 Helicobacter pylori strain Hpfe0011 ['Bacteria', 'Proteobacteria', 'Epsilonproteobacteria', 'Campylobacterales', 'Helicobacteraceae', 'Helicobacter'] PRJNA224116 SAMN26675514 ASM2292579v1 GCF_022925795.1
HEPY001.0523.00247 1562288 1 1396 64 45 210 210 Helicobacter pylori strain Hpfe0007 ['Bacteria', 'Proteobacteria', 'Epsilonproteobacteria', 'Campylobacterales', 'Helicobacteraceae', 'Helicobacter'] PRJNA224116 SAMN26675512 ASM2292583v1 GCF_022925835.1
HEPY001.0523.00248 1563148 2 1386 66 45 210 210 Helicobacter pylori strain Hpfe0006 ['Bacteria', 'Proteobacteria', 'Epsilonproteobacteria', 'Campylobacterales', 'Helicobacteraceae', 'Helicobacter'] PRJNA224116 SAMN26675511 ASM2292585v1 GCF_022925855.1
HEPY001.0523.00249 1560176 1 1376 70 45 210 210 Helicobacter pylori strain Hpfe0004 ['Bacteria', 'Proteobacteria', 'Epsilonproteobacteria', 'Campylobacterales', 'Helicobacteraceae', 'Helicobacter'] PRJNA224116 SAMN26675510 ASM2292587v1 GCF_022925875.1
HEPY001.0523.00250 1564612 1 1394 60 47 210 210 Helicobacter pylori strain Hpfe0003 ['Bacteria', 'Proteobacteria', 'Epsilonproteobacteria', 'Campylobacterales', 'Helicobacteraceae', 'Helicobacter'] PRJNA224116 SAMN26675509 ASM2292590v1 GCF_022925905.1
HEPY001.0523.00251 1615483 1 1416 83 45 210 210 Helicobacter pylori strain Hpfe0002 ['Bacteria', 'Proteobacteria', 'Epsilonproteobacteria', 'Campylobacterales', 'Helicobacteraceae', 'Helicobacter'] PRJNA224116 SAMN26675508 ASM2292592v1 GCF_022925925.1
HEPY001.0523.00252 1649959 1 1461 84 45 210 210 Helicobacter pylori strain Hpfe0001 ['Bacteria', 'Proteobacteria', 'Epsilonproteobacteria', 'Campylobacterales', 'Helicobacteraceae', 'Helicobacter'] PRJNA224116 SAMN26675507 ASM2292594v1 GCF_022925945.1
HEPY001.0523.00253 1624837 1 1425 97 45 210 210 Helicobacter pylori strain JSHR3 ['Bacteria', 'Proteobacteria', 'Epsilonproteobacteria', 'Campylobacterales', 'Helicobacteraceae', 'Helicobacter'] PRJNA224116 SAMD00232837 ASM2316964v1 GCF_023169645.1
HEPY001.0523.00254 1619721 2 1424 71 45 210 210 Helicobacter pylori strain JSHR31 ['Bacteria', 'Proteobacteria', 'Epsilonproteobacteria', 'Campylobacterales', 'Helicobacteraceae', 'Helicobacter'] PRJNA224116 SAMD00232839 ASM2316966v1 GCF_023169665.1
HEPY001.0523.00255 1551197 1 1363 85 45 210 210 Helicobacter pylori strain JSHR6 ['Bacteria', 'Proteobacteria', 'Epsilonproteobacteria', 'Campylobacterales', 'Helicobacteraceae', 'Helicobacter'] PRJNA224116 SAMD00232838 ASM2316978v1 GCF_023169785.1
HEPY001.0523.00256 1696601 1 1504 68 45 210 210 Helicobacter pylori strain CHC155 ['Bacteria', 'Proteobacteria', 'Epsilonproteobacteria', 'Campylobacterales', 'Helicobacteraceae', 'Helicobacter'] PRJNA224116 SAMD00509734 ASM2599845v1 GCF_025998455.1
HEPY001.0523.00258 1627316 2 1439 77 45 210 210 Helicobacter pylori strain 444A6 ['Bacteria', 'Pseudomonadota', 'Epsilonproteobacteria', 'Campylobacterales', 'Helicobacteraceae', 'Helicobacter'] PRJNA224116 SAMN32959140 ASM2866205v1 GCF_028662055.1
HEPY001.0523.00260 1582823 1 1412 70 45 210 210 Helicobacter pylori strain HP22 ['Bacteria', 'Campylobacterota', 'Epsilonproteobacteria', 'Campylobacterales', 'Helicobacteraceae', 'Helicobacter'] PRJNA224116 SAMN34152678 ASM2984917v1 GCF_029849175.1
HEPY001.0523.00261 1667625 1 1474 77 45 210 210 Helicobacter pylori isolate HE171/09 ['Bacteria', 'Proteobacteria', 'Epsilonproteobacteria', 'Campylobacterales', 'Helicobacteraceae', 'Helicobacter'] PRJNA224116 SAMEA4554840 HE171_09 GCF_900119955.1
HEPY001.0523.00262 1667804 1 1450 99 45 210 210 Helicobacter pylori isolate HE143/09 ['Bacteria', 'Proteobacteria', 'Epsilonproteobacteria', 'Campylobacterales', 'Helicobacteraceae', 'Helicobacter'] PRJNA224116 SAMEA4554837 HE143_09 GCF_900119995.1
HEPY001.0523.00263 1667894 1 1466 86 45 210 210 Helicobacter pylori isolate HE178/09 ['Bacteria', 'Proteobacteria', 'Epsilonproteobacteria', 'Campylobacterales', 'Helicobacteraceae', 'Helicobacter'] PRJNA224116 SAMEA4554841 HE178_09 GCF_900120055.1
HEPY001.0523.00265 1667858 1 1470 81 45 210 210 Helicobacter pylori isolate HE134/09 ['Bacteria', 'Proteobacteria', 'Epsilonproteobacteria', 'Campylobacterales', 'Helicobacteraceae', 'Helicobacter'] PRJNA224116 SAMEA4554833 HE134_09 GCF_900120185.1
HEPY001.0523.00266 1670384 1 1472 83 45 210 210 Helicobacter pylori isolate HE141/09 ['Bacteria', 'Proteobacteria', 'Epsilonproteobacteria', 'Campylobacterales', 'Helicobacteraceae', 'Helicobacter'] PRJNA224116 SAMEA4554835 HE141_09 GCF_900120205.1
HEPY001.0523.00267 1667978 1 1458 93 45 210 210 Helicobacter pylori isolate HE136/09 ['Bacteria', 'Proteobacteria', 'Epsilonproteobacteria', 'Campylobacterales', 'Helicobacteraceae', 'Helicobacter'] PRJNA224116 SAMEA4554834 HE136_09 GCF_900120225.1
HEPY001.0523.00268 1667821 1 1462 90 45 210 210 Helicobacter pylori isolate HE101/09 ['Bacteria', 'Proteobacteria', 'Epsilonproteobacteria', 'Campylobacterales', 'Helicobacteraceae', 'Helicobacter'] PRJNA224116 SAMEA4554831 HE101_09 GCF_900120255.1
HEPY001.0523.00270 1670321 1 1473 83 45 210 210 Helicobacter pylori isolate HE170/09 ['Bacteria', 'Proteobacteria', 'Epsilonproteobacteria', 'Campylobacterales', 'Helicobacteraceae', 'Helicobacter'] PRJNA224116 SAMEA4554839 HE170_09 GCF_900120315.1
HEPY001.0523.00271 1667712 1 1463 87 45 210 210 Helicobacter pylori isolate HE147/09 ['Bacteria', 'Proteobacteria', 'Epsilonproteobacteria', 'Campylobacterales', 'Helicobacteraceae', 'Helicobacter'] PRJNA224116 SAMEA4554838 HE147_09 GCF_900120335.1
HEPY001.0523.00272 1667883 1 1472 82 45 210 210 Helicobacter pylori isolate BCM-300 ['Bacteria', 'Proteobacteria', 'Epsilonproteobacteria', 'Campylobacterales', 'Helicobacteraceae', 'Helicobacter'] PRJNA224116 SAMEA33005668 BCM-300 GCF_900149805.1
HEPY001.0523.00273 1682800 1 1481 87 45 210 210 Helicobacter pylori isolate HE93/10_v1 ['Bacteria', 'Proteobacteria', 'Epsilonproteobacteria', 'Campylobacterales', 'Helicobacteraceae', 'Helicobacter'] PRJNA224116 SAMEA103957791 HE93_10 GCF_900176185.1
HEPY001.0523.00274 1644315 1 1454 65 45 210 210 Helicobacter pylori strain NCTC13345 ['Bacteria', 'Proteobacteria', 'Epsilonproteobacteria', 'Campylobacterales', 'Helicobacteraceae', 'Helicobacter'] PRJNA224116 SAMEA104224776 57685_G01 GCF_900638475.1
HEPY001.0523.00275 1632224 1 1411 91 45 210 210 Helicobacter pylori strain NCTC12823 ['Bacteria', 'Proteobacteria', 'Epsilonproteobacteria', 'Campylobacterales', 'Helicobacteraceae', 'Helicobacter'] PRJNA224116 SAMEA104224786 57728_F01 GCF_900638505.1
HEPY001.0523.00276 1566655 1 1394 73 45 210 210 Helicobacter pylori isolate MGYG-HGUT-01357 ['Bacteria', 'Proteobacteria', 'Epsilonproteobacteria', 'Campylobacterales', 'Helicobacteraceae', 'Helicobacter'] PRJNA224116 SAMEA5850860 UHGG_MGYG-HGUT-01357 GCF_902381665.1
HEPY001.0523.00277 1667892 1 1454 99 45 85962 210 Helicobacter pylori 26695 ['Bacteria', 'Proteobacteria', 'Epsilonproteobacteria', 'Campylobacterales', 'Helicobacteraceae', 'Helicobacter'] PRJNA224116 SAMN02603761 ASM30779v1 GCF_000307795.1
HEPY001.0523.00278 1643831 1 1454 59 45 85963 210 Helicobacter pylori J99 ['Bacteria', 'Campylobacterota', 'Epsilonproteobacteria', 'Campylobacterales', 'Helicobacteraceae', 'Helicobacter'] PRJNA224116 SAMN02602990 ASM878v1 GCF_000008785.1

HEPY001.0523.00279 1698366 1 1469 78 45 85963 210 Helicobacter pylori J99 ['Bacteria', 'Campylobacterota', 'Epsilonproteobacteria', 'Campylobacterales', 'Helicobacteraceae', 'Helicobacter'] PRJNA224116 SAMN03487572 ASM98269v1 GCF_000982695.1
HEPY001.0523.00281 1650561 1 1451 69 45 102611 210 Helicobacter pylori J166 ['Bacteria', 'Proteobacteria', 'Epsilonproteobacteria', 'Campylobacterales', 'Helicobacteraceae', 'Helicobacter'] PRJNA224116 SAMN02736817 ASM68562v1 GCF_000685625.1
HEPY001.0523.00282 1624943 2 1435 70 45 102617 210 Helicobacter pylori SS1 ['Bacteria', 'Pseudomonadota', 'Epsilonproteobacteria', 'Campylobacterales', 'Helicobacteraceae', 'Helicobacter'] PRJNA224116 SAMN03331743 ASM200552v1 GCF_002005525.1
HEPY001.0523.00283 1680937 1 1493 86 45 102618 210 Helicobacter pylori NCTC 11637 = CCUG 17874 = ATCC 43504 = JCM 12093 strain NCTC 11637 ['Bacteria', 'Proteobacteria', 'Epsilonproteobacteria', 'Campylobacterales', 'Helicobacteraceae', 'Helicobacter'] PRJNA224116 SAMEA3178013 35377_D02 GCF_900478295.1
HEPY001.0523.00284 1589954 1 1409 78 45 290847 210 Helicobacter pylori 51 ['Bacteria', 'Proteobacteria', 'Epsilonproteobacteria', 'Campylobacterales', 'Helicobacteraceae', 'Helicobacter'] PRJNA224116 SAMN02603300 ASM1172v1 GCF_000011725.1
HEPY001.0523.00285 1605736 2 1409 78 45 357544 210 Helicobacter pylori HPAG1 ['Bacteria', 'Campylobacterota', 'Epsilonproteobacteria', 'Campylobacterales', 'Helicobacteraceae', 'Helicobacter'] PRJNA224116 SAMN02604311 ASM1324v1 GCF_000013245.1
HEPY001.0523.00286 1608548 1 1431 73 45 512562 210 Helicobacter pylori Shi470 ['Bacteria', 'Proteobacteria', 'Epsilonproteobacteria', 'Campylobacterales', 'Helicobacteraceae', 'Helicobacter'] PRJNA224116 SAMN02604319 ASM2024v1 GCF_000020245.1
HEPY001.0523.00287 1675360 1 1471 81 45 544406 210 Helicobacter pylori B128 ['Bacteria', 'Campylobacterota', 'Epsilonproteobacteria', 'Campylobacterales', 'Helicobacteraceae', 'Helicobacter'] PRJNA224116 SAMN08055032 ASM784407v1 GCF_007844075.1
HEPY001.0523.00288 1663013 2 1459 95 45 563041 210 Helicobacter pylori G27 ['Bacteria', 'Campylobacterota', 'Epsilonproteobacteria', 'Campylobacterales', 'Helicobacteraceae', 'Helicobacter'] PRJNA224116 SAMN02604237 ASM2116v1 GCF_000021165.1
HEPY001.0523.00289 1684038 2 1472 100 45 570508 210 Helicobacter pylori P12 ['Bacteria', 'Proteobacteria', 'Epsilonproteobacteria', 'Campylobacterales', 'Helicobacteraceae', 'Helicobacter'] PRJNA224116 SAMN02603595 ASM2146v1 GCF_000021465.1
HEPY001.0523.00290 1566655 1 1393 72 45 585535 210 Helicobacter pylori 35A ['Bacteria', 'Proteobacteria', 'Epsilonproteobacteria', 'Campylobacterales', 'Helicobacteraceae', 'Helicobacter'] PRJNA224116 SAMN02299423 ASM17893v2 GCF_000178935.2
HEPY001.0523.00291 1617426 1 1407 99 45 585538 210 Helicobacter pylori 83 ['Bacteria', 'Proteobacteria', 'Epsilonproteobacteria', 'Campylobacterales', 'Helicobacteraceae', 'Helicobacter'] PRJNA224116 SAMN00189522 ASM21313v1 GCF_000213135.1
HEPY001.0523.00292 1576758 1 1415 62 45 592205 210 Helicobacter pylori B38 ['Bacteria', 'Proteobacteria', 'Epsilonproteobacteria', 'Campylobacterales', 'Helicobacteraceae', 'Helicobacter'] PRJNA224116 SAMEA3138296 ASM9134v1 GCF_000091345.1
HEPY001.0523.00293 1595604 2 1427 75 45 637913 210 Helicobacter pylori v225d ['Bacteria', 'Campylobacterota', 'Epsilonproteobacteria', 'Campylobacterales', 'Helicobacteraceae', 'Helicobacter'] PRJNA224116 SAMN00003101 ASM9318v1 GCF_000093185.1
HEPY001.0523.00294 1568826 1 1371 89 45 684950 210 Helicobacter pylori 52 ['Bacteria', 'Proteobacteria', 'Epsilonproteobacteria', 'Campylobacterales', 'Helicobacteraceae', 'Helicobacter'] PRJNA224116 SAMN02603357 ASM2380v1 GCF_000023805.1
HEPY001.0523.00295 1680029 2 1477 77 45 693745 210 Helicobacter pylori B8 ['Bacteria', 'Proteobacteria', 'Epsilonproteobacteria', 'Campylobacterales', 'Helicobacteraceae', 'Helicobacter'] PRJNA224116 SAMEA2272698 ASM19675v1 GCF_000196755.1
HEPY001.0523.00296 1658051 1 1449 72 45 765962 210 Helicobacter pylori SJM180 ['Bacteria', 'Proteobacteria', 'Epsilonproteobacteria', 'Campylobacterales', 'Helicobacteraceae', 'Helicobacter'] PRJNA224116 SAMN02603202 ASM14885v1 GCF_000148855.1
HEPY001.0523.00297 1638269 2 1460 64 45 765963 210 Helicobacter pylori PeCan4 ['Bacteria', 'Proteobacteria', 'Epsilonproteobacteria', 'Campylobacterales', 'Helicobacteraceae', 'Helicobacter'] PRJNA224116 SAMN02603203 ASM14887v1 GCF_000148875.1
HEPY001.0523.00298 1635449 1 1478 60 46 765964 210 Helicobacter pylori Cuz20 ['Bacteria', 'Proteobacteria', 'Epsilonproteobacteria', 'Campylobacterales', 'Helicobacteraceae', 'Helicobacter'] PRJNA224116 SAMN02603204 ASM14889v1 GCF_000148895.1
HEPY001.0523.00299 1567570 2 1412 55 45 794851 210 Helicobacter pylori Sat464 ['Bacteria', 'Proteobacteria', 'Epsilonproteobacteria', 'Campylobacterales', 'Helicobacteraceae', 'Helicobacter'] PRJNA224116 SAMN02603200 ASM14891v1 GCF_000148915.1
HEPY001.0523.00300 1575399 1 1393 77 45 866344 210 Helicobacter pylori F16 ['Bacteria', 'Proteobacteria', 'Epsilonproteobacteria', 'Campylobacterales', 'Helicobacteraceae', 'Helicobacter'] PRJNA224116 SAMD00060971 ASM27000v1 GCF_000270005.1
HEPY001.0523.00302 1609006 1 1419 80 45 866346 210 Helicobacter pylori F57 ['Bacteria', 'Proteobacteria', 'Epsilonproteobacteria', 'Campylobacterales', 'Helicobacteraceae', 'Helicobacter'] PRJNA224116 SAMD00060974 ASM27006v1 GCF_000270065.1
HEPY001.0523.00303 1640673 2 1442 90 45 907237 210 Helicobacter pylori Lithuania75 ['Bacteria', 'Proteobacteria', 'Epsilonproteobacteria', 'Campylobacterales', 'Helicobacteraceae', 'Helicobacter'] PRJNA224116 SAMN02603023 ASM18522v1 GCF_000185225.1
HEPY001.0523.00304 1675918 1 1483 91 45 907238 210 Helicobacter pylori India7 ['Bacteria', 'Proteobacteria', 'Epsilonproteobacteria', 'Campylobacterales', 'Helicobacteraceae', 'Helicobacter'] PRJNA224116 SAMN02603026 ASM18518v1 GCF_000185185.1
HEPY001.0523.00305 1679829 2 1472 88 45 907239 210 Helicobacter pylori SouthAfrica7 ['Bacteria', 'Proteobacteria', 'Epsilonproteobacteria', 'Campylobacterales', 'Helicobacteraceae', 'Helicobacter'] PRJNA224116 SAMN02603025 ASM18524v1 GCF_000185245.1
HEPY001.0523.00306 1712468 2 1521 70 45 907240 210 Helicobacter pylori Gambia94/24 ['Bacteria', 'Proteobacteria', 'Epsilonproteobacteria', 'Campylobacterales', 'Helicobacteraceae', 'Helicobacter'] PRJNA224116 SAMN02603024 ASM18520v1 GCF_000185205.1
HEPY001.0523.00307 1669876 2 1463 87 45 1055527 210 Helicobacter pylori ELS37 ['Bacteria', 'Proteobacteria', 'Epsilonproteobacteria', 'Campylobacterales', 'Helicobacteraceae', 'Helicobacter'] PRJNA224116 SAMN02604318 ASM25595v1 GCF_000255955.1
HEPY001.0523.00308 1637762 2 1435 82 45 1055528 210 Helicobacter pylori Puno120 ['Bacteria', 'Proteobacteria', 'Epsilonproteobacteria', 'Campylobacterales', 'Helicobacteraceae', 'Helicobacter'] PRJNA224116 SAMN02604320 ASM22453v1 GCF_000224535.1
HEPY001.0523.00309 1646139 1 1482 53 45 1055529 210 Helicobacter pylori Puno135 ['Bacteria', 'Proteobacteria', 'Epsilonproteobacteria', 'Campylobacterales', 'Helicobacteraceae', 'Helicobacter'] PRJNA224116 SAMN02604321 ASM22455v1 GCF_000224555.1
HEPY001.0523.00310 1610830 2 1412 82 45 1055530 210 Helicobacter pylori SNT49 ['Bacteria', 'Proteobacteria', 'Epsilonproteobacteria', 'Campylobacterales', 'Helicobacteraceae', 'Helicobacter'] PRJNA224116 SAMN02604322 ASM22457v1 GCF_000224575.1
HEPY001.0523.00311 1636125 3 1465 59 45 1055531 210 Helicobacter pylori Aklavik117 ['Bacteria', 'Proteobacteria', 'Epsilonproteobacteria', 'Campylobacterales', 'Helicobacteraceae', 'Helicobacter'] PRJNA224116 SAMN02604323 ASM31595v1 GCF_000315955.1
HEPY001.0523.00312 1507930 3 1357 52 45 1055532 210 Helicobacter pylori Aklavik86 ['Bacteria', 'Proteobacteria', 'Epsilonproteobacteria', 'Campylobacterales', 'Helicobacteraceae', 'Helicobacter'] PRJNA224116 SAMN02604324 ASM31787v1 GCF_000317875.1
HEPY001.0523.00313 1665719 1 1481 61 45 1163739 210 Helicobacter pylori Shi417 ['Bacteria', 'Proteobacteria', 'Epsilonproteobacteria', 'Campylobacterales', 'Helicobacteraceae', 'Helicobacter'] PRJNA224116 SAMN02603193 ASM27736v1 GCF_000277365.1
HEPY001.0523.00314 1663456 1 1485 69 46 1163740 210 Helicobacter pylori Shi112 ['Bacteria', 'Proteobacteria', 'Epsilonproteobacteria', 'Campylobacterales', 'Helicobacteraceae', 'Helicobacter'] PRJNA224116 SAMN02603194 ASM27740v1 GCF_000277405.1
HEPY001.0523.00315 1616909 1 1437 74 45 1163741 210 Helicobacter pylori Shi169 ['Bacteria', 'Proteobacteria', 'Epsilonproteobacteria', 'Campylobacterales', 'Helicobacteraceae', 'Helicobacter'] PRJNA224116 SAMN02603195 ASM27738v1 GCF_000277385.1
HEPY001.0523.00316 1660685 1 1465 69 45 1163742 210 Helicobacter pylori PeCan18 ['Bacteria', 'Proteobacteria', 'Epsilonproteobacteria', 'Campylobacterales', 'Helicobacteraceae', 'Helicobacter'] PRJNA224116 SAMN02603196 ASM27742v1 GCF_000277425.1
HEPY001.0523.00317 1607584 2 1430 65 45 1163743 210 Helicobacter pylori HUP-B14 ['Bacteria', 'Proteobacteria', 'Epsilonproteobacteria', 'Campylobacterales', 'Helicobacteraceae', 'Helicobacter'] PRJNA224116 SAMN02603197 ASM25923v1 GCF_000259235.1
HEPY001.0523.00318 1673791 2 1465 85 45 1231719 210 Helicobacter pylori A45 ['Bacteria', 'Proteobacteria', 'Epsilonproteobacteria', 'Campylobacterales', 'Helicobacteraceae', 'Helicobacter'] PRJNA224116 SAMN02470830 ASM33383v2 GCF_000333835.2
HEPY001.0523.00319 1667883 1 1453 100 45 1234365 210 Helicobacter pylori Rif1 ['Bacteria', 'Proteobacteria', 'Epsilonproteobacteria', 'Campylobacterales', 'Helicobacteraceae', 'Helicobacter'] PRJNA224116 SAMN02603762 ASM30781v1 GCF_000307815.1
HEPY001.0523.00320 1667890 1 1454 98 45 1234600 210 Helicobacter pylori Rif2 ['Bacteria', 'Proteobacteria', 'Epsilonproteobacteria', 'Campylobacterales', 'Helicobacteraceae', 'Helicobacter'] PRJNA224116 SAMN02603763 ASM30783v1 GCF_000307835.1
HEPY001.0523.00321 1616617 1 1416 90 45 1248725 210 Helicobacter pylori OK113 ['Bacteria', 'Proteobacteria', 'Epsilonproteobacteria', 'Campylobacterales', 'Helicobacteraceae', 'Helicobacter'] PRJNA224116 SAMD00061056 ASM34886v1 GCF_000348865.1
HEPY001.0523.00322 1595436 2 1430 71 45 1248726 210 Helicobacter pylori OK310 ['Bacteria', 'Proteobacteria', 'Epsilonproteobacteria', 'Campylobacterales', 'Helicobacteraceae', 'Helicobacter'] PRJNA224116 SAMD00061057 ASM34888v1 GCF_000348885.1
HEPY001.0523.00323 1593537 1 1422 68 45 1311573 210 Helicobacter pylori UM032 ['Bacteria', 'Proteobacteria', 'Epsilonproteobacteria', 'Campylobacterales', 'Helicobacteraceae', 'Helicobacter'] PRJNA224116 SAMN02230257 ASM39245v3 GCF_000392455.3
HEPY001.0523.00324 1594569 1 1425 67 45 1321938 210 Helicobacter pylori UM299 ['Bacteria', 'Proteobacteria', 'Epsilonproteobacteria', 'Campylobacterales', 'Helicobacteraceae', 'Helicobacter'] PRJNA224116 SAMN02230259 ASM39247v3 GCF_000392475.3
HEPY001.0523.00325 1692794 1 1491 95 45 1321939 210 Helicobacter pylori UM037 ['Bacteria', 'Proteobacteria', 'Epsilonproteobacteria', 'Campylobacterales', 'Helicobacteraceae', 'Helicobacter'] PRJNA224116 SAMN02604194 ASM39251v3 GCF_000392515.3
HEPY001.0523.00326 1658047 1 1462 67 45 1321940 210 Helicobacter pylori UM066 ['Bacteria', 'Proteobacteria', 'Epsilonproteobacteria', 'Campylobacterales', 'Helicobacteraceae', 'Helicobacter'] PRJNA224116 SAMN02230271 ASM39253v3 GCF_000392535.3
HEPY001.0523.00328 1633212 1 1462 63 45 1382920 210 Helicobacter pylori oki102 ['Bacteria', 'Proteobacteria', 'Epsilonproteobacteria', 'Campylobacterales', 'Helicobacteraceae', 'Helicobacter'] PRJNA224116 SAMN03081488 ASM60004v1 GCF_000600045.1
HEPY001.0523.00329 1637925 1 1452 72 45 1382921 210 Helicobacter pylori oki112 ['Bacteria', 'Proteobacteria', 'Epsilonproteobacteria', 'Campylobacterales', 'Helicobacteraceae', 'Helicobacter'] PRJNA224116 SAMN03081489 ASM60008v1 GCF_000600085.1
HEPY001.0523.00330 1553826 1 1360 89 45 1382922 210 Helicobacter pylori oki128 ['Bacteria', 'Proteobacteria', 'Epsilonproteobacteria', 'Campylobacterales', 'Helicobacteraceae', 'Helicobacter'] PRJNA224116 SAMN03081490 ASM60012v1 GCF_000600125.1
HEPY001.0523.00331 1599700 1 1399 92 45 1382923 210 Helicobacter pylori oki154 ['Bacteria', 'Proteobacteria', 'Epsilonproteobacteria', 'Campylobacterales', 'Helicobacteraceae', 'Helicobacter'] PRJNA224116 SAMN03081491 ASM60014v1 GCF_000600145.1
HEPY001.0523.00332 1634852 1 1429 86 45 1382924 210 Helicobacter pylori oki422 ['Bacteria', 'Proteobacteria', 'Epsilonproteobacteria', 'Campylobacterales', 'Helicobacteraceae', 'Helicobacter'] PRJNA224116 SAMN03081492 ASM60016v1 GCF_000600165.1
HEPY001.0523.00333 1595058 1 1390 94 45 1382925 210 Helicobacter pylori oki673 ['Bacteria', 'Proteobacteria', 'Epsilonproteobacteria', 'Campylobacterales', 'Helicobacteraceae', 'Helicobacter'] PRJNA224116 SAMN03081493 ASM60018v1 GCF_000600185.1
HEPY001.0523.00334 1600345 1 1404 88 45 1382926 210 Helicobacter pylori oki828 ['Bacteria', 'Proteobacteria', 'Epsilonproteobacteria', 'Campylobacterales', 'Helicobacteraceae', 'Helicobacter'] PRJNA224116 SAMN03081494 ASM60020v1 GCF_000600205.1
HEPY001.0523.00335 1634875 1 1454 65 45 1382927 210 Helicobacter pylori oki898 ['Bacteria', 'Proteobacteria', 'Epsilonproteobacteria', 'Campylobacterales', 'Helicobacteraceae', 'Helicobacter'] PRJNA224116 SAMN03081495 ASM60022v1 GCF_000600225.1
HEPY001.0523.00336 1667638 1 1467 85 45 1391726 210 Helicobacter pylori 26695-1 ['Bacteria', 'Campylobacterota', 'Epsilonproteobacteria', 'Campylobacterales', 'Helicobacteraceae', 'Helicobacter'] PRJNA224116 SAMN03268385 ASM82698v1 GCF_000826985.1
HEPY001.0523.00337 1667638 1 1467 85 45 1391726 210 Helicobacter pylori 26695-1 ['Bacteria', 'Proteobacteria', 'Epsilonproteobacteria', 'Campylobacterales', 'Helicobacteraceae', 'Helicobacter'] PRJNA224116 SAMD00061015 ASM82909v1 GCF_000829095.1
HEPY001.0523.00338 1667239 1 1465 86 45 1391727 210 Helicobacter pylori 26695-1CL ['Bacteria', 'Proteobacteria', 'Epsilonproteobacteria', 'Campylobacterales', 'Helicobacteraceae', 'Helicobacter'] PRJNA224116 SAMD00061016 ASM82911v1 GCF_000829115.1
HEPY001.0523.00339 1667302 1 1466 86 45 1391728 210 Helicobacter pylori 26695-1CH ['Bacteria', 'Proteobacteria', 'Epsilonproteobacteria', 'Campylobacterales', 'Helicobacteraceae', 'Helicobacter'] PRJNA224116 SAMD00061017 ASM82913v1 GCF_000829135.1
HEPY001.0523.00340 1660425 1 1462 100 45 1407462 210 Helicobacter pylori BM012A ['Bacteria', 'Campylobacterota', 'Epsilonproteobacteria', 'Campylobacterales', 'Helicobacteraceae', 'Helicobacter'] PRJNA224116 SAMN02376623 ASM49831v1 GCF_000498315.1
HEPY001.0523.00341 1660469 1 1460 102 45 1407463 210 Helicobacter pylori BM012S ['Bacteria', 'Campylobacterota', 'Epsilonproteobacteria', 'Campylobacterales', 'Helicobacteraceae', 'Helicobacter'] PRJNA224116 SAMN02376624 ASM49833v1 GCF_000498335.1
HEPY001.0523.00342 1696917 1 1480 133 45 1426844 210 Helicobacter pylori NY40 ['Bacteria', 'Proteobacteria', 'Epsilonproteobacteria', 'Campylobacterales', 'Helicobacteraceae', 'Helicobacter'] PRJNA224116 SAMD00061026 ASM82895v1 GCF_000828955.1
HEPY001.0523.00343 1624538 2 1436 67 45 1431450 210 Helicobacter pylori PMSS1 ['Bacteria', 'Pseudomonadota', 'Epsilonproteobacteria', 'Campylobacterales', 'Helicobacteraceae', 'Helicobacter'] PRJNA224116 SAMN04362855 ASM199109v1 GCF_001991095.1
HESP003.0523.00001 1610826 5 1572 51 46 2849641 2849641 Helicobacter sp. NHP19-003 ['Bacteria', 'Campylobacterota', 'Epsilonproteobacteria', 'Campylobacterales', 'Helicobacteraceae', 'Helicobacter'] PRJNA224116 SAMD00334520 ASM1970330v1 GCF_019703305.1
HESP004.0523.00001 1612665 8 1566 93 46 2849642 2849642 Helicobacter sp. NHP19-012 ['Bacteria', 'Campylobacterota', 'Epsilonproteobacteria', 'Campylobacterales', 'Helicobacteraceae', 'Helicobacter'] PRJNA224116 SAMD00334521 ASM1970332v1 GCF_019703325.1
HESU001.0523.00001 1694897 3 1650 71 49 104628 104628 Helicobacter suis strain SNTW101c ['Bacteria', 'Proteobacteria', 'Epsilonproteobacteria', 'Campylobacterales', 'Helicobacteraceae', 'Helicobacter'] PRJNA224116 SAMD00173587 ASM703620v1 GCF_007036205.1
HESU001.0523.00002 1777109 3 1723 76 49 104628 104628 Helicobacter suis strain NHP19-0020 ['Bacteria', 'Proteobacteria', 'Epsilonproteobacteria', 'Campylobacterales', 'Helicobacteraceae', 'Helicobacter'] PRJNA224116 SAMD00215134 ASM1337415v1 GCF_013374155.1
HESU001.0523.00003 1715041 3 1676 62 49 104628 104628 Helicobacter suis strain NHP19-4003 ['Bacteria', 'Proteobacteria', 'Epsilonproteobacteria', 'Campylobacterales', 'Helicobacteraceae', 'Helicobacter'] PRJNA224116 SAMD00215131 ASM1337417v1 GCF_013374175.1
HESU001.0523.00004 1738770 4 1739 61 49 104628 104628 Helicobacter suis strain NHP19-4004 ['Bacteria', 'Proteobacteria', 'Epsilonproteobacteria', 'Campylobacterales', 'Helicobacteraceae', 'Helicobacter'] PRJNA224116 SAMD00215132 ASM1337419v1 GCF_013374195.1
HESU001.0523.00005 1682860 3 1652 62 49 104628 104628 Helicobacter suis strain NHP19-4022 ['Bacteria', 'Proteobacteria', 'Epsilonproteobacteria', 'Campylobacterales', 'Helicobacteraceae', 'Helicobacter'] PRJNA224116 SAMD00215133 ASM1337421v1 GCF_013374215.1
HESU001.0523.00006 1775777 3 1716 195 44 710393 104628 Helicobacter suis HS1 ['Bacteria', 'Proteobacteria', 'Epsilonproteobacteria', 'Campylobacterales', 'Helicobacteraceae', 'Helicobacter'] PRJNA224116 SAMD00532366 ASM2600029v1 GCF_026000295.1
HETY001.0523.00001 1920832 1 1902 29 48 76936 76936 Helicobacter typhlonius strain MIT 97-6810 isolate 1 ['Bacteria', 'Proteobacteria', 'Epsilonproteobacteria', 'Campylobacterales', 'Helicobacteraceae', 'Helicobacter'] PRJNA224116 SAMEA3507300 htyphlonius_mit97-6810 GCF_001460635.1
HEWI001.0523.00001 1688978 1 1665 13 53 157268 157268 Helicobacter winghamensis strain 2015D-0170 ['Bacteria', 'Campylobacterota', 'Epsilonproteobacteria', 'Campylobacterales', 'Helicobacteraceae', 'Helicobacter'] PRJNA224116 SAMN04545302 ASM1493117v1 GCF_014931175.1
WOSU001.0523.00001 2150449 1 2106 17 53 844 844 Wolinella succinogenes strain NCTC11488 ['Bacteria', 'Proteobacteria', 'Epsilonproteobacteria', 'Campylobacterales', 'Helicobacteraceae', 'Wolinella'] PRJNA224116 SAMEA4412664 50569_E01 GCF_900637325.1
WOSU001.0523.00002 2110355 1 2053 19 53 273121 844 Wolinella succinogenes DSM 1740 ['Bacteria', 'Proteobacteria', 'Epsilonproteobacteria', 'Campylobacterales', 'Helicobacteraceae', 'Wolinella'] PRJNA224116 SAMEA3138333 ASM19613v1 GCF_000196135.1
