# Supplementary material for: Unveiling a missing component of the atypical type IV secretion system required for natural transformation of Helicobacter pylori
Source: PLoS Pathog. 2026 Jul 14;22(7):e1014140. doi: 10.1371/journal.ppat.1014140 (PMC13395361; doi:10.1371/journal.ppat.1014140)
Supplement: S6 Table — (PDF) [file ppat.1014140.s014.pdf]

```
HMMER3/b [3.0 | March 2010]
NAME  T_virB11
LENG  328
ALPH  amino
RF    no
CS    no
MAP   yes
DATE  Fri Nov 23 17:23:46 2012
NSEQ  73
EFFN  0.717346
CKSUM 1137158280
STATS LOCAL MSV      -11.1088  0.70062
STATS LOCAL VITERBI  -12.1113  0.70062
STATS LOCAL FORWARD   -5.7466  0.70062
HMM          A        C        D        E        F        G        H        I        K        L        M        N        P        Q        R        S        T        V        W        Y   
            m->m     m->i     m->d     i->m     i->i     d->m     d->d
  COMPO   2.38854  4.45760  3.00357  2.66473  3.62695  2.78256  3.78549  2.72577  2.81836  2.33338  3.62017  3.19176  3.30926  3.19909  2.77998  2.73579  2.80516  2.48540  4.88696  3.88056
          2.68605  4.42214  2.77536  2.73130  3.46370  2.40518  3.72500  3.29345  2.67757  2.69362  4.24518  2.90355  2.73730  3.18147  2.89807  2.37881  2.77514  2.98519  4.58456  3.61519
          0.32636  1.65496  2.43781  1.51339  0.24867  0.00000        *
      1   2.45979  4.25749  3.43492  2.87307  3.39806  3.42384  3.72736  2.70828  2.78968  2.44639  2.33460  3.26861  3.51311  3.11687  3.05828  2.76998  2.68683  2.53016  4.84660  3.42289     12 - -
          2.68618  4.42225  2.77519  2.73123  3.46354  2.40513  3.72494  3.29354  2.67741  2.69355  4.24690  2.90347  2.73739  3.18146  2.89801  2.37887  2.77519  2.98518  4.58477  3.61503
          0.02930  3.94069  4.66304  0.61958  0.77255  0.53548  0.88042
      2   2.19468  4.67140  2.94043  2.34518  3.91022  3.34654  3.62500  3.31313  2.44382  2.94094  3.59797  2.96094  3.64792  2.75658  2.76149  2.37300  2.73353  2.86974  5.20868  3.89405     13 - -
          2.68618  4.42225  2.77519  2.73123  3.46354  2.40513  3.72494  3.29354  2.67741  2.69355  4.24690  2.90347  2.73739  3.18146  2.89801  2.37887  2.77519  2.98518  4.58477  3.61503
          0.02873  3.95996  4.68231  0.61958  0.77255  0.49457  0.94118
      3   2.41422  4.50682  3.06877  2.58883  3.69869  3.12546  3.68133  2.93284  2.48926  2.73072  3.35994  3.00288  3.24885  2.90144  2.98887  2.60869  2.57220  2.58746  5.06609  3.78593     14 - -
          2.68618  4.42225  2.77519  2.73123  3.46354  2.40513  3.72494  3.29354  2.67741  2.69355  4.24690  2.90347  2.73739  3.18146  2.89801  2.37887  2.77519  2.98518  4.58477  3.61503
          0.02777  3.99342  4.71576  0.61958  0.77255  0.49800  0.93584
      4   2.43197  4.78481  2.88003  2.35148  3.96592  3.33942  3.41067  3.26806  2.32745  3.00229  3.75035  2.81762  3.78571  2.67633  2.70481  2.33925  2.64533  3.07359  5.28361  3.94580     15 - -
          2.68618  4.42225  2.77519  2.73123  3.46354  2.40513  3.72494  3.29354  2.67741  2.69355  4.24690  2.90347  2.73739  3.18146  2.89801  2.37887  2.77519  2.98518  4.58477  3.61503
          0.02747  4.00411  4.72645  0.61958  0.77255  0.50504  0.92504
      5   2.39738  4.72655  2.92045  2.43477  3.91162  3.40180  3.28308  3.37130  2.42386  2.92624  3.80765  2.90855  3.17509  2.68387  2.85589  2.29522  2.63669  3.03421  5.02030  3.91686     16 - -
          2.68618  4.42225  2.77519  2.73123  3.46354  2.40513  3.72494  3.29354  2.67741  2.69355  4.24690  2.90347  2.73739  3.18146  2.89801  2.37887  2.77519  2.98518  4.58477  3.61503
          0.02747  4.00411  4.72645  0.61958  0.77255  0.50504  0.92504
      6   2.37019  4.46214  3.17414  2.63381  3.42813  3.46772  3.18816  2.83950  2.60579  2.64040  3.43640  3.07998  3.74924  2.76417  2.99234  2.38882  2.69007  2.70540  4.83878  3.74650     17 - -
          2.68618  4.42225  2.77519  2.73123  3.46354  2.40513  3.72494  3.29354  2.67741  2.69355  4.24690  2.90347  2.73739  3.18146  2.89801  2.37887  2.77519  2.98518  4.58477  3.61503
          0.02747  4.00411  4.72645  0.61958  0.77255  0.50504  0.92504
      7   2.44673  4.91962  2.87597  2.30434  4.13380  3.38186  3.13884  3.55878  2.19267  3.19453  3.97963  2.71257  3.77515  2.53407  2.56593  2.44505  2.76587  3.23718  5.38654  4.02586     18 - -
          2.68618  4.42225  2.77519  2.73123  3.46354  2.40513  3.72494  3.29354  2.67741  2.69355  4.24690  2.90347  2.73739  3.18146  2.89801  2.37887  2.77519  2.98518  4.58477  3.61503
          0.02747  4.00411  4.72645  0.61958  0.77255  0.50504  0.92504
      8   2.25322  4.74160  2.86530  2.39814  3.93260  3.39314  3.54156  3.39737  2.42709  2.85527  3.82867  2.86928  3.28566  2.71114  2.84136  2.37832  2.62073  3.07975  5.26136  3.93292     19 - -
          2.68618  4.42225  2.77519  2.73123  3.46354  2.40513  3.72494  3.29354  2.67741  2.69355  4.24690  2.90347  2.73739  3.18146  2.89801  2.37887  2.77519  2.98518  4.58477  3.61503
          0.03176  4.00411  4.34130  0.61958  0.77255  0.50504  0.92504
      9   2.36165  4.83661  2.78685  1.95202  4.12369  3.11695  3.62540  3.45949  2.41250  3.10725  3.94736  2.89579  3.78880  2.73712  2.89579  2.53868  2.50256  3.20344  5.36623  4.01804     20 - -
          2.68618  4.42226  2.77520  2.73119  3.46355  2.40514  3.72495  3.29355  2.67742  2.69354  4.24691  2.90348  2.73740  3.18147  2.89802  2.37888  2.77514  2.98518  4.58478  3.61504
          0.10236  2.42585  4.72228  0.41148  1.08670  0.50226  0.92928
     10   1.88718  4.41031  3.21223  2.66423  3.88209  2.87190  3.80455  3.26591  2.69004  2.93689  3.65273  3.08328  3.78434  3.02436  3.05284  2.14306  2.58403  2.80524  5.23214  3.96199     22 - -
          2.68618  4.42225  2.77519  2.73123  3.46354  2.40513  3.72494  3.29354  2.67741  2.69355  4.24690  2.90347  2.73739  3.18146  2.89801  2.37887  2.77519  2.98518  4.58477  3.61503
          0.02747  4.00411  4.72645  0.61958  0.77255  0.50504  0.92504
     11   2.40756  4.35300  3.24942  2.73345  3.22081  3.50100  3.64750  2.77926  2.71146  2.26271  3.38910  3.16139  3.88305  2.93328  2.85091  2.56864  2.69301  2.52673  4.92396  3.67483     23 - -
          2.68618  4.42225  2.77519  2.73123  3.46354  2.40513  3.72494  3.29354  2.67741  2.69355  4.24690  2.90347  2.73739  3.18146  2.89801  2.37887  2.77519  2.98518  4.58477  3.61503
          0.02747  4.00411  4.72645  0.61958  0.77255  0.50504  0.92504
     12   2.35706  4.93789  2.52488  2.16923  4.22232  3.25683  3.44531  3.66777  2.29438  3.15628  3.90190  2.84102  3.76780  2.64718  2.81136  2.41318  2.71708  3.23333  5.40996  4.03728     24 - -
          2.68618  4.42225  2.77519  2.73123  3.46354  2.40513  3.72494  3.29354  2.67741  2.69355  4.24690  2.90347  2.73739  3.18146  2.89801  2.37887  2.77519  2.98518  4.58477  3.61503
          0.02747  4.00411  4.72645  0.61958  0.77255  0.50504  0.92504
     13   3.15844  4.98527  3.66296  3.14861  4.37339  3.61912  3.92654  3.92556  2.30000  3.41765  4.41579  3.49757  4.11656  3.14154  0.79488  3.23711  3.41624  3.64417  5.40386  4.27364     25 - -
          2.68618  4.42225  2.77519  2.73123  3.46354  2.40513  3.72494  3.29354  2.67741  2.69355  4.24690  2.90347  2.73739  3.18146  2.89801  2.37887  2.77519  2.98518  4.58477  3.61503
          0.02747  4.00411  4.72645  0.61958  0.77255  0.50504  0.92504
     14   2.56485  4.65109  3.08286  2.59119  4.10487  2.37502  3.71552  3.51856  2.42265  2.97122  3.96039  3.04319  3.81605  2.88828  2.11334  2.27977  2.85581  3.16589  5.34712  4.05531     26 - -
          2.68618  4.42225  2.77519  2.73123  3.46354  2.40513  3.72494  3.29354  2.67741  2.69355  4.24690  2.90347  2.73739  3.18146  2.89801  2.37887  2.77519  2.98518  4.58477  3.61503
          0.02747  4.00411  4.72645  0.61958  0.77255  0.50504  0.92504
     15   2.16437  4.32617  3.37252  2.78963  3.48211  3.51259  3.67700  2.41367  2.67194  2.54552  3.35341  3.22736  3.89887  3.06851  2.88465  2.72758  2.62137  2.46361  4.91086  3.66966     27 - -
          2.68618  4.42225  2.77519  2.73123  3.46354  2.40513  3.72494  3.29354  2.67741  2.69355  4.24690  2.90347  2.73739  3.18146  2.89801  2.37887  2.77519  2.98518  4.58477  3.61503
          0.02747  4.00411  4.72645  0.61958  0.77255  0.50504  0.92504
     16   2.46174  4.83684  3.03429  2.49483  4.17438  3.41947  3.62497  3.57920  2.27160  3.12015  3.97341  2.98614  3.83541  2.35935  2.04668  2.47219  2.71786  3.24133  5.35285  4.05062     28 - -
          2.68618  4.42225  2.77519  2.73123  3.46354  2.40513  3.72494  3.29354  2.67741  2.69355  4.24690  2.90347  2.73739  3.18146  2.89801  2.37887  2.77519  2.98518  4.58477  3.61503
          0.02747  4.00411  4.72645  0.61958  0.77255  0.47390  0.97437
     17   3.09302  4.61941  4.21342  3.82053  3.28161  3.94754  4.52101  2.47256  3.57968  1.85572  1.11700  4.07878  4.41101  3.95287  3.76690  3.45673  3.40235  2.49560  5.14109  3.91495     29 - -
          2.68618  4.42225  2.77519  2.73123  3.46354  2.40513  3.72494  3.29354  2.67741  2.69355  4.24690  2.90347  2.73739  3.18146  2.89801  2.37887  2.77519  2.98518  4.58477  3.61503
          0.02696  4.02262  4.74496  0.61958  0.77255  0.48576  0.95510
     18   3.32709  4.74617  4.42998  4.07280  3.19879  4.17148  4.68160  2.41344  3.83907  0.71022  3.17896  4.33366  4.58250  4.17133  3.98965  3.76330  3.61534  2.48932  5.13017  3.88739     30 - -
          2.68618  4.42225  2.77519  2.73123  3.46354  2.40513  3.72494  3.29354  2.67741  2.69355  4.24690  2.90347  2.73739  3.18146  2.89801  2.37887  2.77519  2.98518  4.58477  3.61503
          0.02696  4.02262  4.74496  0.61958  0.77255  0.48576  0.95510
     19   2.92428  4.96539  3.37342  2.79780  4.28977  3.58146  3.41584  3.76052  2.04394  3.27447  4.16283  3.19922  4.00306  2.83644  1.24115  2.96467  2.94684  3.45209  5.35706  4.11620     31 - -
          2.68618  4.42225  2.77519  2.73123  3.46354  2.40513  3.72494  3.29354  2.67741  2.69355  4.24690  2.90347  2.73739  3.18146  2.89801  2.37887  2.77519  2.98518  4.58477  3.61503
```

	0.02696	4.02262	4.74496	0.61958	0.77255	0.48576	0.95510													
20	2.32754	4.23242	3.43794	3.17163	4.14106	3.04903	4.21350	3.38657	3.14650	3.21583	4.13437	3.35330	3.77615	3.50790	3.42624	2.33850	1.14665	2.97902	5.54496	4.30169	32 - -
	2.68618	4.42225	2.77519	2.73123	3.46354	2.40513	3.72494	3.29354	2.67741	2.69355	4.24690	2.90347	2.73739	3.18146	2.89801	2.37887	2.77519	2.98518	4.58477	3.61503	
	0.02696	4.02262	4.74496	0.61958	0.77255	0.48576	0.95510													
21	0.81728	4.27297	3.62041	3.42136	4.20706	3.09296	4.42492	3.35544	3.44162	3.22989	4.24718	3.53130	3.84663	3.76944	3.68019	2.62296	2.91086	2.98372	5.61916	4.43687	33 - -
	2.68618	4.42225	2.77519	2.73123	3.46354	2.40513	3.72494	3.29354	2.67741	2.69355	4.24690	2.90347	2.73739	3.18146	2.89801	2.37887	2.77519	2.98518	4.58477	3.61503	
	0.02696	4.02262	4.74496	0.61958	0.77255	0.48576	0.95510													
22	3.19985	4.55997	4.72221	4.14813	2.72494	4.41127	4.67527	2.19630	4.02916	1.14521	1.91779	4.40215	4.62096	4.13764	4.16163	3.73683	3.41869	2.23708	5.04138	3.93630	34 - -
	2.68618	4.42225	2.77519	2.73123	3.46354	2.40513	3.72494	3.29354	2.67741	2.69355	4.24690	2.90347	2.73739	3.18146	2.89801	2.37887	2.77519	2.98518	4.58477	3.61503	
	0.02696	4.02262	4.74496	0.61958	0.77255	0.48576	0.95510													
23	2.35923	3.41443	3.79079	3.52577	4.30782	0.89376	4.44179	3.70397	3.48788	3.46570	4.36057	3.56110	3.79794	3.79790	3.71475	2.56325	2.86499	3.20598	5.61468	4.48856	35 - -
	2.68618	4.42225	2.77519	2.73123	3.46354	2.40513	3.72494	3.29354	2.67741	2.69355	4.24690	2.90347	2.73739	3.18146	2.89801	2.37887	2.77519	2.98518	4.58477	3.61503	
	0.02696	4.02262	4.74496	0.61958	0.77255	0.48576	0.95510													
24	2.33471	4.46007	3.11827	2.76019	4.12964	3.12134	3.90036	3.52683	2.69882	3.13503	4.03984	3.13690	1.62368	3.11649	2.83449	2.51915	2.84142	3.13616	5.43408	4.16391	36 - -
	2.68618	4.42225	2.77519	2.73123	3.46354	2.40513	3.72494	3.29354	2.67741	2.69355	4.24690	2.90347	2.73739	3.18146	2.89801	2.37887	2.77519	2.98518	4.58477	3.61503	
	0.02696	4.02262	4.74496	0.61958	0.77255	0.48576	0.95510													
25	2.01566	4.40145	3.24369	2.62665	3.59127	3.47320	3.74965	2.93075	2.68222	2.32371	3.44358	3.16629	3.61127	3.01427	3.03741	2.66735	2.72847	2.55104	4.99820	3.74187	37 - -
	2.68618	4.42225	2.77519	2.73123	3.46354	2.40513	3.72494	3.29354	2.67741	2.69355	4.24690	2.90347	2.73739	3.18146	2.89801	2.37887	2.77519	2.98518	4.58477	3.61503	
	0.02696	4.02262	4.74496	0.61958	0.77255	0.48576	0.95510													
26	3.15498	4.47237	4.79650	4.26349	3.39751	4.48843	4.93221	1.10234	4.12894	1.79933	2.99301	4.51140	4.74729	4.35947	4.31280	3.85143	3.40216	1.66821	5.39618	4.23653	38 - -
	2.68618	4.42225	2.77519	2.73123	3.46354	2.40513	3.72494	3.29354	2.67741	2.69355	4.24690	2.90347	2.73739	3.18146	2.89801	2.37887	2.77519	2.98518	4.58477	3.61503	
	0.02696	4.02262	4.74496	0.61958	0.77255	0.48576	0.95510													
27	1.80235	4.45621	3.21952	2.67973	3.73620	3.13232	3.74490	3.03379	2.61984	2.70844	3.54302	3.12190	3.84049	2.90355	2.89174	2.57071	2.68569	2.82660	5.10582	3.83882	39 - -
	2.68618	4.42225	2.77519	2.73123	3.46354	2.40513	3.72494	3.29354	2.67741	2.69355	4.24690	2.90347	2.73739	3.18146	2.89801	2.37887	2.77519	2.98518	4.58477	3.61503	
	0.02696	4.02262	4.74496	0.61958	0.77255	0.48576	0.95510													
28	2.05546	4.79072	2.92552	2.22467	4.07264	2.95250	3.51561	3.48640	2.40688	3.03480	3.90489	2.94326	3.80864	2.78921	2.58098	2.63841	2.73859	3.15746	5.32172	3.99439	40 - -
	2.68618	4.42225	2.77519	2.73123	3.46354	2.40513	3.72494	3.29354	2.67741	2.69355	4.24690	2.90347	2.73739	3.18146	2.89801	2.37887	2.77519	2.98518	4.58477	3.61503	
	0.02696	4.02262	4.74496	0.61958	0.77255	0.48576	0.95510													
29	2.07985	4.20818	3.64798	3.07989	2.68925	3.60882	3.29566	2.68586	2.99999	2.26924	3.31518	3.41687	3.98277	3.28183	3.17035	2.87096	2.82577	2.47796	3.61001	3.20469	41 - -
	2.68618	4.42225	2.77519	2.73123	3.46354	2.40513	3.72494	3.29354	2.67741	2.69355	4.24690	2.90347	2.73739	3.18146	2.89801	2.37887	2.77519	2.98518	4.58477	3.61503	
	0.02696	4.02262	4.74496	0.61958	0.77255	0.48576	0.95510													
30	3.27698	4.70300	4.53222	4.08186	3.09512	4.31512	4.67655	2.26529	3.81083	0.80343	2.95659	4.34634	4.63983	4.12118	3.97388	3.74950	3.54301	2.35550	5.10743	3.86784	42 - -
	2.68618	4.42225	2.77519	2.73123	3.46354	2.40513	3.72494	3.29354	2.67741	2.69355	4.24690	2.90347	2.73739	3.18146	2.89801	2.37887	2.77519	2.98518	4.58477	3.61503	
	0.02696	4.02262	4.74496	0.61958	0.77255	0.48576	0.95510													
31	2.38721	5.16075	2.13940	1.71115	4.46703	3.31062	3.64473	3.93518	2.47673	3.45645	4.24789	2.78890	3.80050	2.75472	2.79872	2.64068	2.93235	3.53558	5.62829	4.21241	43 - -
	2.68618	4.42225	2.77519	2.73123	3.46354	2.40513	3.72494	3.29354	2.67741	2.69355	4.24690	2.90347	2.73739	3.18146	2.89801	2.37887	2.77519	2.98518	4.58477	3.61503	
	0.02696	4.02262	4.74496	0.61958	0.77255	0.48576	0.95510													
32	2.73724	5.37359	1.10776	2.09189	4.72965	3.22528	3.82330	4.18233	2.86040	3.76815	4.65102	2.73543	3.86519	3.00387	3.44246	2.84595	3.24372	3.78165	5.95718	4.47416	44 - -
	2.68618	4.42225	2.77519	2.73123	3.46354	2.40513	3.72494	3.29354	2.67741	2.69355	4.24690	2.90347	2.73739	3.18146	2.89801	2.37887	2.77519	2.98518	4.58477	3.61503	
	0.02696	4.02262	4.74496	0.61958	0.77255	0.48576	0.95510													
33	2.41449	4.68380	2.63936	2.48599	4.20897	3.23594	3.79740	3.62939	2.66231	3.25209	4.08832	2.89258	1.76610	2.94652	3.04132	2.57616	2.86285	3.25044	5.49048	4.16569	45 - -
	2.68618	4.42225	2.77519	2.73123	3.46354	2.40513	3.72494	3.29354	2.67741	2.69355	4.24690	2.90347	2.73739	3.18146	2.89801	2.37887	2.77519	2.98518	4.58477	3.61503	
	0.02696	4.02262	4.74496	0.61958	0.77255	0.48576	0.95510													
34	2.31098	4.92840	2.36516	2.27789	4.21761	3.10071	3.59749	3.65928	2.38193	3.12305	3.94624	2.86722	3.77865	2.65020	2.77081	2.34647	2.79901	3.29302	5.41849	4.04914	46 - -
	2.68618	4.42225	2.77519	2.73123	3.46354	2.40513	3.72494	3.29354	2.67741	2.69355	4.24690	2.90347	2.73739	3.18146	2.89801	2.37887	2.77519	2.98518	4.58477	3.61503	
	0.02696	4.02262	4.74496	0.61958	0.77255	0.48576	0.95510													
35	2.96947	4.35886	4.55241	4.05094	3.60581	4.20806	4.80235	1.61031	3.93576	2.19753	3.43296	4.28088	4.58679	4.22750	4.16177	3.58108	2.98882	1.06698	5.46010	4.24928	47 - -
	2.68618	4.42225	2.77519	2.73123	3.46354	2.40513	3.72494	3.29354	2.67741	2.69355	4.24690	2.90347	2.73739	3.18146	2.89801	2.37887	2.77519	2.98518	4.58477	3.61503	
	0.02696	4.02262	4.74496	0.61958	0.77255	0.48576	0.95510													
36	2.63582	4.27885	4.27330	3.76898	3.57396	3.71743	4.52054	1.75265	3.66133	2.31919	3.45423	4.00192	4.36702	3.95538	3.90072	3.29287	3.09497	1.17027	5.28335	4.06646	48 - -
	2.68618	4.42225	2.77519	2.73123	3.46354	2.40513	3.72494	3.29354	2.67741	2.69355	4.24690	2.90347	2.73739	3.18146	2.89801	2.37887	2.77519	2.98518	4.58477	3.61503	
	0.02696	4.02262	4.74496	0.61958	0.77255	0.48576	0.95510													
37	3.08846	5.22327	2.56059	0.82931	4.58940	3.37593	3.97883	4.07348	2.87880	3.66752	4.63740	3.02771	3.98839	3.19292	3.30140	3.05703	3.39464	3.74275	5.73268	4.45792	49 - -
	2.68618	4.42225	2.77519	2.73123	3.46354	2.40513	3.72494	3.29354	2.67741	2.69355	4.24690	2.90347	2.73739	3.18146	2.89801	2.37887	2.77519	2.98518	4.58477	3.61503	
	0.02696	4.02262	4.74496	0.61958	0.77255	0.48576	0.95510													
38	3.07605	4.41612	4.73312	4.24631	3.58108	4.37861	4.97828	1.52935	4.12658	2.10237	3.39984	4.46738	4.72077	4.40835	4.33479	3.77100	3.35083	1.01637	5.53084	4.31194	50 - -
	2.68618	4.42225	2.77519	2.73123	3.46354	2.40513	3.72494	3.29354	2.67741	2.69355	4.24690	2.90347	2.73739	3.18146	2.89801	2.37887	2.77519	2.98518	4.58477	3.61503	
	0.02696	4.02262	4.74496	0.61958	0.77255	0.48576	0.95510													
39	3.09302	4.61941	4.21342	3.82053	3.28161	3.94754	4.52101	2.47256	3.57968	1.85572	1.11700	4.07878	4.41101	3.95287	3.76690	3.45673	3.40235	2.49560	5.14109	3.91495	51 - -
	2.68618	4.42225	2.77519	2.73123	3.46354	2.40513	3.72494	3.29354	2.67741	2.69355	4.24690	2.90347	2.73739	3.18146	2.89801	2.37887	2.77519	2.98518	4.58477	3.61503	
	0.02696	4.02262	4.74496	0.61958	0.77255	0.48576	0.95510													
40	2.82966	4.49111	4.37636	3.88327	3.27203	4.10209	4.59636	2.14998	3.71128	1.05610	3.12456	4.14348	4.48059	3.99594	3.91771	3.47546	3.29518	1.94826	5.19921	4.02699	52 - -
	2.68618	4.42225	2.77519	2.73123	3.46354	2.40513	3.72494	3.29354	2.67741	2.69355	4.24690	2.90347	2.73739	3.18146	2.89801	2.37887	2.77519	2.98518	4.58477	3.61503	
	0.02696	4.02262	4.74496	0.61958	0.77255	0.48576	0.95510													
41	2.85426	4.85176	2.80169	2.70353	4.23951	3.28976	4.05172	3.98473	2.96940	3.61516	4.57129	0.91734	3.95002	3.32667	3.32750	2.91694	3.24227	3.59163	5.52835	4.17856	53 - -
	2.68618	4.42225	2.77519	2.73123	3.46354	2.40513	3.72494	3.29354	2.67741	2.69355	4.24690	2.90347	2.73739	3.18146	2.89801	2.37887	2.77519	2.98518	4.58477	3.61503	
	0.02696	4.02262	4.74496	0.61958	0.77255	0.48576	0.95510													
42	2.48890	4.47103	3.32141	3.14997	4.26228	3.18238	4.28322	3.68779	3.21714	3.37082	4.36974	3.42592	0.94042	3.59333	3.49540	2.75487	3.05099	3.29393	5.57869	4.38375	54 - -
	2.68618	4.42225	2.77519	2.73123	3.46354	2.40513	3.72494	3.29354	2.67741	2.69355	4.24690	2.90347	2.73739	3.18146	2.89801	2.37887	2.77519	2.98518	4.58477	3.61503	
	0.02696	4.02262	4.74496	0.61958	0.77255	0.48576	0.95510													
43	3.15706	5.30096	0.72443	2.44038	4.69960	3.32995	4.07187	4.32326	3.18574	3.90577	4.88941	3.00343	3.99358	3.31287	3.72060	3.10404	3.49962	3.94721	5.83844	4.55063	55 - -
	2.68618	4.42225	2.77519	2.73123	3.46354	2.40513	3.72494	3.29354	2.67741	2.69355	4.24690	2.90347	2.73739	3.18146	2.89801	2.37887	2.77519	2.98518	4.58477	3.61503	
	0.02696	4.02262	4.74496	0.61958	0.77255	0.48576	0.95510													
44	2.65527	4.63846	3.16157	2.82806	4.30888	1.34001	3.94341	3.78300	2.63266	3.38504	4.25591	3.21742	3.88439	3.15663	2.17360	2.75924	3.01835	3.38059	5.49881	4.27630	56 - -
	2.68618	4.42225	2.77519	2.73123	3.46354	2.40513	3.72494	3.29354	2.67741	2.69355	4.24690	2.90347	2.73739	3.18146	2.89801	2.37887	2.77519	2.98518	4.58477	3.61503	

	0.02696	4.02262	4.74496	0.61958	0.77255	0.48576	0.95510														
45	2.47470	4.53148	3.11870	2.55423	4.00522	3.41774	3.65840	3.39503	2.29522	3.01420	3.84899	3.03152	3.83526	2.79912	2.01273	2.53393	2.47474	3.00119	5.26075	3.97074	57 - -
	2.68618	4.42225	2.77519	2.73123	3.46354	2.40513	3.72494	3.29354	2.67741	2.69355	4.24690	2.90347	2.73739	3.18146	2.89801	2.37887	2.77519	2.98518	4.58477	3.61503	
	0.02696	4.02262	4.74496	0.61958	0.77255	0.48576	0.95510														
46	3.26136	4.60353	4.78892	4.24967	3.17004	4.51192	4.86370	1.81572	4.08903	0.96343	2.97918	4.51855	4.73540	4.27160	4.24576	3.87321	3.49843	1.99622	5.23714	4.11677	58 - -
	2.68618	4.42225	2.77519	2.73123	3.46354	2.40513	3.72494	3.29354	2.67741	2.69355	4.24690	2.90347	2.73739	3.18146	2.89801	2.37887	2.77519	2.98518	4.58477	3.61503	
	0.02696	4.02262	4.74496	0.61958	0.77255	0.48576	0.95510														
47	3.03248	4.65789	3.84572	3.34493	2.93094	3.75268	3.80923	3.24819	2.83659	2.77431	3.83359	3.61761	4.19286	3.41374	2.53452	3.21214	3.28451	3.06564	1.29506	2.94089	59 - -
	2.68618	4.42225	2.77519	2.73123	3.46354	2.40513	3.72494	3.29354	2.67741	2.69355	4.24690	2.90347	2.73739	3.18146	2.89801	2.37887	2.77519	2.98518	4.58477	3.61503	
	0.02696	4.02262	4.74496	0.61958	0.77255	0.48576	0.95510														
48	3.09388	4.40673	4.77894	4.25035	3.49907	4.44873	4.93004	1.45136	4.14092	1.81832	3.30518	4.48336	4.72835	4.38009	4.33353	3.80974	3.28063	1.21222	5.44029	4.26120	60 - -
	2.68618	4.42225	2.77519	2.73123	3.46354	2.40513	3.72494	3.29354	2.67741	2.69355	4.24690	2.90347	2.73739	3.18146	2.89801	2.37887	2.77519	2.98518	4.58477	3.61503	
	0.02696	4.02262	4.74496	0.61958	0.77255	0.48576	0.95510														
49	3.02893	5.48400	0.98636	2.05910	4.79876	3.24293	3.86424	4.32108	2.93478	3.87410	4.77862	2.75157	3.89584	3.05466	3.52904	2.92156	3.34072	3.91678	5.99549	4.52634	61 - -
	2.68618	4.42225	2.77519	2.73123	3.46354	2.40513	3.72494	3.29354	2.67741	2.69355	4.24690	2.90347	2.73739	3.18146	2.89801	2.37887	2.77519	2.98518	4.58477	3.61503	
	0.02696	4.02262	4.74496	0.61958	0.77255	0.48576	0.95510														
50	2.91649	4.71513	3.49459	2.83325	4.26425	3.61800	3.66509	3.62926	1.96064	3.02631	4.08375	3.22724	4.01096	2.82722	1.27299	2.96958	3.12216	3.34530	5.33743	4.12317	62 - -
	2.68618	4.42225	2.77519	2.73123	3.46354	2.40513	3.72494	3.29354	2.67741	2.69355	4.24690	2.90347	2.73739	3.18146	2.89801	2.37887	2.77519	2.98518	4.58477	3.61503	
	0.02696	4.02262	4.74496	0.61958	0.77255	0.48576	0.95510														
51	2.89892	4.56515	4.39049	3.90279	2.92728	4.18185	4.52506	2.25266	3.75023	0.94788	3.04327	4.17187	4.51816	3.99526	3.94638	3.54976	3.36777	2.28750	5.02583	3.76945	63 - -
	2.68618	4.42225	2.77519	2.73123	3.46354	2.40513	3.72494	3.29354	2.67741	2.69355	4.24690	2.90347	2.73739	3.18146	2.89801	2.37887	2.77519	2.98518	4.58477	3.61503	
	0.02696	4.02262	4.74496	0.61958	0.77255	0.48576	0.95510														
52	2.36558	4.69870	2.75321	2.47022	4.17061	2.66434	3.70982	3.59746	2.52445	3.19535	4.00597	2.95790	3.77813	2.86879	2.65422	1.90356	2.82855	3.18707	5.42152	4.09106	64 - -
	2.68618	4.42225	2.77519	2.73123	3.46354	2.40513	3.72494	3.29354	2.67741	2.69355	4.24690	2.90347	2.73739	3.18146	2.89801	2.37887	2.77519	2.98518	4.58477	3.61503	
	0.02696	4.02262	4.74496	0.61958	0.77255	0.48576	0.95510														
53	2.56802	4.87373	2.75394	1.98315	4.14744	3.12210	3.61193	3.57642	2.40698	2.97671	3.95925	2.88914	3.78867	2.65771	2.87865	2.26832	2.79682	3.04124	5.37671	4.02130	65 - -
	2.68618	4.42225	2.77519	2.73123	3.46354	2.40513	3.72494	3.29354	2.67741	2.69355	4.24690	2.90347	2.73739	3.18146	2.89801	2.37887	2.77519	2.98518	4.58477	3.61503	
	0.02696	4.02262	4.74496	0.61958	0.77255	0.48576	0.95510														
54	2.86181	4.62005	3.56615	3.48578	4.62096	0.53093	4.59479	4.25913	3.68734	3.89379	4.87234	3.73076	4.01589	3.99980	3.90487	3.03839	3.35475	3.75848	5.67431	4.72868	66 - -
	2.68618	4.42225	2.77519	2.73123	3.46354	2.40513	3.72494	3.29354	2.67741	2.69355	4.24690	2.90347	2.73739	3.18146	2.89801	2.37887	2.77519	2.98518	4.58477	3.61503	
	0.02696	4.02262	4.74496	0.61958	0.77255	0.48576	0.95510														
55	2.76103	4.44224	3.67578	3.08390	3.39328	3.73718	3.94570	2.46349	2.81521	1.67935	3.10812	3.47406	4.09229	3.25292	2.26228	3.00913	2.98718	2.51542	4.98560	3.77088	67 - -
	2.68618	4.42225	2.77519	2.73123	3.46354	2.40513	3.72494	3.29354	2.67741	2.69355	4.24690	2.90347	2.73739	3.18146	2.89801	2.37887	2.77519	2.98518	4.58477	3.61503	
	0.02696	4.02262	4.74496	0.61958	0.77255	0.48576	0.95510														
56	2.04298	4.49917	3.16374	2.39385	3.62329	3.33692	3.70350	2.90730	2.59106	2.75170	3.61437	3.08702	3.84800	2.90177	2.94361	2.58262	2.75558	2.57565	5.07371	3.79745	68 - -
	2.68618	4.42225	2.77519	2.73123	3.46354	2.40513	3.72494	3.29354	2.67741	2.69355	4.24690	2.90347	2.73739	3.18146	2.89801	2.37887	2.77519	2.98518	4.58477	3.61503	
	0.02696	4.02262	4.74496	0.61958	0.77255	0.48576	0.95510														
57	2.54500	4.28455	2.05053	2.34578	4.14339	3.34519	3.63852	3.56036	2.44483	3.15722	3.96441	2.90752	2.91204	2.78414	2.88545	2.58670	2.83549	3.07106	5.38452	4.03429	69 - -
	2.68618	4.42225	2.77519	2.73123	3.46354	2.40513	3.72494	3.29354	2.67741	2.69355	4.24690	2.90347	2.73739	3.18146	2.89801	2.37887	2.77519	2.98518	4.58477	3.61503	
	0.02696	4.02262	4.74496	0.61958	0.77255	0.48576	0.95510														
58	2.46977	4.30570	3.49208	2.95782	3.56469	3.45876	3.90135	2.75552	2.86923	2.39653	3.03886	3.32661	3.92376	3.20991	3.20727	2.60305	1.82647	2.52907	5.03029	3.79435	70 - -
	2.68618	4.42225	2.77519	2.73123	3.46354	2.40513	3.72494	3.29354	2.67741	2.69355	4.24690	2.90347	2.73739	3.18146	2.89801	2.37887	2.77519	2.98518	4.58477	3.61503	
	0.02696	4.02262	4.74496	0.61958	0.77255	0.48576	0.95510														
59	2.55814	4.78661	2.20252	2.40955	4.48845	1.40568	3.90813	3.95418	2.89466	3.56970	4.41601	2.85537	3.80615	3.10747	3.38282	2.67149	3.01172	3.49965	5.75934	4.38810	71 - -
	2.68618	4.42225	2.77519	2.73123	3.46354	2.40513	3.72494	3.29354	2.67741	2.69355	4.24690	2.90347	2.73739	3.18146	2.89801	2.37887	2.77519	2.98518	4.58477	3.61503	
	0.02696	4.02262	4.74496	0.61958	0.77255	0.48576	0.95510														
60	2.49597	4.42122	2.86903	2.17954	3.86172	3.42756	3.64103	3.15750	2.46078	2.74588	3.68718	2.99304	3.81670	2.70361	2.84914	2.64060	2.76813	2.50969	5.17864	3.87089	72 - -
	2.68618	4.42225	2.77519	2.73123	3.46354	2.40513	3.72494	3.29354	2.67741	2.69355	4.24690	2.90347	2.73739	3.18146	2.89801	2.37887	2.77519	2.98518	4.58477	3.61503	
	0.02696	4.02262	4.74496	0.61958	0.77255	0.48576	0.95510														
61	2.58465	4.71451	3.02625	2.23041	3.87480	3.42387	3.53774	3.31548	2.40539	2.94119	3.72586	2.97462	3.81348	2.79103	2.43175	2.56168	2.42783	2.81916	5.22585	3.86549	73 - -
	2.68618	4.42225	2.77519	2.73123	3.46354	2.40513	3.72494	3.29354	2.67741	2.69355	4.24690	2.90347	2.73739	3.18146	2.89801	2.37887	2.77519	2.98518	4.58477	3.61503	
	0.02696	4.02262	4.74496	0.61958	0.77255	0.48576	0.95510														
62	3.26318	4.59092	4.83811	4.27045	3.02364	4.53245	4.83761	1.84331	4.15294	0.98619	2.62230	4.53422	4.71827	4.26015	4.28454	3.87146	3.48436	2.10831	5.17154	4.10067	74 - -
	2.68618	4.42225	2.77519	2.73123	3.46354	2.40513	3.72494	3.29354	2.67741	2.69355	4.24690	2.90347	2.73739	3.18146	2.89801	2.37887	2.77519	2.98518	4.58477	3.61503	
	0.02696	4.02262	4.74496	0.61958	0.77255	0.48576	0.95510														
63	2.41360	4.71558	2.90569	2.39179	4.10021	3.15658	3.56688	3.52150	2.50346	3.12605	3.94187	2.77482	3.06732	2.84311	2.95778	2.03321	2.53955	3.16815	5.36540	4.03563	75 - -
	2.68618	4.42225	2.77519	2.73123	3.46354	2.40513	3.72494	3.29354	2.67741	2.69355	4.24690	2.90347	2.73739	3.18146	2.89801	2.37887	2.77519	2.98518	4.58477	3.61503	
	0.02696	4.02262	4.74496	0.61958	0.77255	0.48576	0.95510														
64	2.13160	4.79902	2.81838	2.12229	4.16555	3.33317	3.68907	3.57452	2.48250	3.17886	4.00510	2.93642	2.44202	2.85047	2.79646	2.65287	2.88790	3.22745	5.41251	4.08062	76 - -
	2.68618	4.42225	2.77519	2.73123	3.46354	2.40513	3.72494	3.29354	2.67741	2.69355	4.24690	2.90347	2.73739	3.18146	2.89801	2.37887	2.77519	2.98518	4.58477	3.61503	
	0.02696	4.02262	4.74496	0.61958	0.77255	0.48576	0.95510														
65	1.62852	4.58982	2.97830	2.52065	3.97509	3.27586	3.48532	3.37593	2.58855	3.02051	3.86970	3.03960	3.82471	2.92387	2.92608	2.41843	2.82007	3.05394	5.29164	4.00107	77 - -
	2.68618	4.42225	2.77519	2.73123	3.46354	2.40513	3.72494	3.29354	2.67741	2.69355	4.24690	2.90347	2.73739	3.18146	2.89801	2.37887	2.77519	2.98518	4.58477	3.61503	
	0.02696	4.02262	4.74496	0.61958	0.77255	0.48576	0.95510														
66	2.98340	5.56072	1.14813	1.91447	4.83212	3.22706	3.79017	4.36120	2.82860	3.86249	4.72613	2.64543	3.86264	2.87247	3.43102	2.86008	3.27741	3.93585	6.02356	4.50327	78 - -
	2.68618	4.42225	2.77519	2.73123	3.46354	2.40513	3.72494	3.29354	2.67741	2.69355	4.24690	2.90347	2.73739	3.18146	2.89801	2.37887	2.77519	2.98518	4.58477	3.61503	
	0.02696	4.02262	4.74496	0.61958	0.77255	0.48576	0.95510														
67	1.89806	4.30778	3.32279	2.90556	4.03263	1.77211	3.97959	3.37967	2.87514	3.09033	3.94777	3.21786	3.76752	3.22262	3.00124	2.52488	2.76373	2.68900	5.38856	4.14179	79 - -
	2.68618	4.42225	2.77519	2.73123	3.46354	2.40513	3.72494	3.29354	2.67741	2.69355	4.24690	2.90347	2.73739	3.18146	2.89801	2.37887	2.77519	2.98518	4.58477	3.61503	
	0.02696	4.02262	4.74496	0.61958	0.77255	0.48576	0.95510														
68	3.01368	5.26401	2.39764	0.93623	4.61151	3.32603	3.89459	4.08734	2.81135	3.68249	4.60583	2.91001	3.93780	3.09101	3.27166	2.96207	3.31408	3.73183	5.77856	4.43062	80 - -
	2.68618	4.42225	2.77519	2.73123	3.46354	2.40513	3.72494	3.29354	2.67741	2.69355	4.24690	2.90347	2.73739	3.18146	2.89801	2.37887	2.77519	2.98518	4.58477	3.61503	
	0.02696	4.02262	4.74496	0.61958	0.77255	0.48576	0.95510														
69	3.18120	5.00542	3.68780	3.17038	4.39834	3.63746	3.94268	3.95237	2.31116	3.44111	4.43934	3.51762	4.13451	3.15727	0.77162	3.25933	3.43807	3.67032	5.42160	4.29519	81 - -
	2.68618	4.42225	2.77519	2.73123	3.46354	2.40513	3.72494	3.29354	2.67741	2.69355	4.24690	2.90347	2.73739	3.18146	2.89801	2.37887	2.77519	2.98518	4.58477	3.61503	

```
    0.02696 4.02262 4.74496 0.61958 0.77255 0.48576 0.95510
70  3.11119 4.46777 4.70557 4.24500 3.48636 4.35843 4.95445 1.01305 4.09156 1.98149 3.32576 4.46366 4.71286 4.38255 4.28469 3.76975 3.39259 1.63714 5.46980 4.24688   82 - -
    2.68618 4.42225 2.77519 2.73123 3.46354 2.40513 3.72494 3.29354 2.67741 2.69355 4.24690 2.90347 2.73739 3.18146 2.89801 2.37887 2.77519 2.98518 4.58477 3.61503
    0.02696 4.02262 4.74496 0.61958 0.77255 0.48576 0.95510
71  3.13656 4.43568 4.84937 4.33147 3.50523 4.51234 5.01671 1.21193 4.21771 1.87999 3.30545 4.56155 4.78359 4.45774 4.40603 3.88627 3.39000 1.34692 5.49058 4.30712   83 - -
    2.68618 4.42225 2.77519 2.73123 3.46354 2.40513 3.72494 3.29354 2.67741 2.69355 4.24690 2.90347 2.73739 3.18146 2.89801 2.37887 2.77519 2.98518 4.58477 3.61503
    0.02696 4.02262 4.74496 0.61958 0.77255 0.48576 0.95510
72  3.07513 5.07244 3.51319 2.91331 4.49254 3.65339 3.70331 3.89347 1.91091 3.37854 4.29270 3.28796 4.07154 2.86318 1.06253 3.10641 3.27420 3.59448 5.42882 4.26099   84 - -
    2.68618 4.42225 2.77519 2.73123 3.46354 2.40513 3.72494 3.29354 2.67741 2.69355 4.24690 2.90347 2.73739 3.18146 2.89801 2.37887 2.77519 2.98518 4.58477 3.61503
    0.02696 4.02262 4.74496 0.61958 0.77255 0.48576 0.95510
73  3.32709 4.74617 4.42998 4.07280 3.19879 4.17148 4.68160 2.41344 3.83907 0.71022 3.17896 4.33366 4.58250 4.17133 3.98965 3.76330 3.61534 2.48932 5.13017 3.88739   85 - -
    2.68618 4.42225 2.77519 2.73123 3.46354 2.40513 3.72494 3.29354 2.67741 2.69355 4.24690 2.90347 2.73739 3.18146 2.89801 2.37887 2.77519 2.98518 4.58477 3.61503
    0.02696 4.02262 4.74496 0.61958 0.77255 0.48576 0.95510
74  2.92784 4.43147 4.33116 4.00117 3.66379 3.87690 4.77821 2.05427 3.87125 2.32619 3.60830 4.16627 4.43068 4.22125 4.06895 3.39165 3.28677 0.85478 5.47286 4.21283   86 - -
    2.68618 4.42225 2.77519 2.73123 3.46354 2.40513 3.72494 3.29354 2.67741 2.69355 4.24690 2.90347 2.73739 3.18146 2.89801 2.37887 2.77519 2.98518 4.58477 3.61503
    0.02696 4.02262 4.74496 0.61958 0.77255 0.48576 0.95510
75  0.81728 4.27297 3.62041 3.42136 4.20706 3.09296 4.42492 3.35544 3.44162 3.22989 4.24718 3.53130 3.84663 3.76944 3.68019 2.62296 2.91086 2.98372 5.61916 4.43687   87 - -
    2.68618 4.42225 2.77519 2.73123 3.46354 2.40513 3.72494 3.29354 2.67741 2.69355 4.24690 2.90347 2.73739 3.18146 2.89801 2.37887 2.77519 2.98518 4.58477 3.61503
    0.02696 4.02262 4.74496 0.61958 0.77255 0.48576 0.95510
76  2.11891 4.58616 2.99596 2.61073 3.98428 3.28063 2.10710 3.47818 2.60966 3.11274 3.96273 3.05526 3.81792 2.99402 2.99745 2.23522 2.86485 3.12786 5.31137 3.98071   88 - -
    2.68618 4.42225 2.77519 2.73123 3.46354 2.40513 3.72494 3.29354 2.67741 2.69355 4.24690 2.90347 2.73739 3.18146 2.89801 2.37887 2.77519 2.98518 4.58477 3.61503
    0.02696 4.02262 4.74496 0.61958 0.77255 0.48576 0.95510
77  2.55845 4.16799 3.16223 2.73689 3.77741 3.10106 1.67599 3.41067 2.61815 3.05227 3.92223 3.14266 3.84995 3.05163 2.96249 2.55104 2.88084 3.07711 5.15823 3.78866   89 - -
    2.68618 4.42225 2.77519 2.73123 3.46354 2.40513 3.72494 3.29354 2.67741 2.69355 4.24690 2.90347 2.73739 3.18146 2.89801 2.37887 2.77519 2.98518 4.58477 3.61503
    0.02696 4.02262 4.74496 0.61958 0.77255 0.48576 0.95510
78  2.67212 4.31809 4.31590 3.84339 3.65941 3.92159 4.64286 1.86860 3.72500 2.32056 3.50773 4.05737 4.39852 4.04052 3.96752 3.30956 2.94481 1.05963 5.43160 4.21520   90 - -
    2.68618 4.42225 2.77519 2.73123 3.46354 2.40513 3.72494 3.29354 2.67741 2.69355 4.24690 2.90347 2.73739 3.18146 2.89801 2.37887 2.77519 2.98518 4.58477 3.61503
    0.02696 4.02262 4.74496 0.61958 0.77255 0.48576 0.95510
79  2.63366 4.72581 2.86167 2.56163 4.25999 1.60066 3.79245 3.71032 2.51687 3.30394 4.14911 2.83983 3.84079 2.88352 2.54690 2.69673 2.95621 3.32806 5.48853 4.18439   91 - -
    2.68618 4.42225 2.77519 2.73123 3.46354 2.40513 3.72494 3.29354 2.67741 2.69355 4.24690 2.90347 2.73739 3.18146 2.89801 2.37887 2.77519 2.98518 4.58477 3.61503
    0.02696 4.02262 4.74496 0.61958 0.77255 0.48576 0.95510
80  1.51326 4.31773 3.36109 2.77072 3.82680 3.22387 3.96609 2.96674 2.90247 2.84015 3.74888 3.26917 3.85052 3.23734 3.25495 2.64439 2.66183 2.31465 5.24719 4.00473   92 - -
    2.68618 4.42225 2.77519 2.73123 3.46354 2.40513 3.72494 3.29354 2.67741 2.69355 4.24690 2.90347 2.73739 3.18146 2.89801 2.37887 2.77519 2.98518 4.58477 3.61503
    0.02696 4.02262 4.74496 0.61958 0.77255 0.48576 0.95510
81  2.94064 5.19180 2.44185 1.00964 4.62051 2.98738 3.87307 4.09629 2.77536 3.67472 4.56368 2.89869 3.91056 3.06210 3.23411 2.90217 3.24790 3.71382 5.77696 4.43117   93 - -
    2.68618 4.42225 2.77519 2.73123 3.46354 2.40513 3.72494 3.29354 2.67741 2.69355 4.24690 2.90347 2.73739 3.18146 2.89801 2.37887 2.77519 2.98518 4.58477 3.61503
    0.02696 4.02262 4.74496 0.61958 0.77255 0.48576 0.95510
82  2.80679 4.37419 4.40040 3.98188 3.63585 3.97781 4.76981 1.88066 3.84041 2.24623 3.50598 4.16802 4.47374 4.17563 4.06235 3.40046 3.22342 0.96348 5.49091 4.24082   94 - -
    2.68618 4.42225 2.77519 2.73123 3.46354 2.40513 3.72494 3.29354 2.67741 2.69355 4.24690 2.90347 2.73739 3.18146 2.89801 2.37887 2.77519 2.98518 4.58477 3.61503
    0.02696 4.02262 4.74496 0.61958 0.77255 0.48576 0.95510
83  2.82504 5.01149 2.47955 2.40249 3.92878 3.35898 1.56515 3.79138 2.58520 3.35324 4.23089 2.76247 3.89684 2.95790 2.99303 2.82050 3.09922 3.45309 5.29350 3.81801   95 - -
    2.68618 4.42225 2.77519 2.73123 3.46354 2.40513 3.72494 3.29354 2.67741 2.69355 4.24690 2.90347 2.73739 3.18146 2.89801 2.37887 2.77519 2.98518 4.58477 3.61503
    0.02696 4.02262 4.74496 0.61958 0.77255 0.48576 0.95510
84  2.16886 4.89197 2.85922 2.21935 4.17411 3.30634 3.50980 3.60427 2.35640 3.17243 3.97166 2.89945 3.47503 2.65442 2.42297 2.50310 2.81941 3.25282 5.37978 4.02971   96 - -
    2.68618 4.42225 2.77519 2.73123 3.46354 2.40513 3.72494 3.29354 2.67741 2.69355 4.24690 2.90347 2.73739 3.18146 2.89801 2.37887 2.77519 2.98518 4.58477 3.61503
    0.02696 4.02262 4.74496 0.61958 0.77255 0.48576 0.95510
85  2.27940 4.75254 2.81616 2.38407 4.09946 2.22472 3.61569 3.51680 2.48646 3.09121 3.94484 2.94562 3.80342 2.84191 2.69389 2.46219 2.85535 3.17536 5.36246 4.03476   97 - -
    2.68618 4.42225 2.77519 2.73123 3.46354 2.40513 3.72494 3.29354 2.67741 2.69355 4.24690 2.90347 2.73739 3.18146 2.89801 2.37887 2.77519 2.98518 4.58477 3.61503
    0.02696 4.02262 4.74496 0.61958 0.77255 0.48576 0.95510
86  2.21857 4.76905 2.95438 2.44684 4.10239 3.36501 3.64329 3.52007 2.40904 3.11426 3.92779 2.78458 3.79880 2.44737 2.65529 2.21637 2.69959 3.14188 5.34236 4.01536   98 - -
    2.68618 4.42225 2.77519 2.73123 3.46354 2.40513 3.72494 3.29354 2.67741 2.69355 4.24690 2.90347 2.73739 3.18146 2.89801 2.37887 2.77519 2.98518 4.58477 3.61503
    0.02696 4.02262 4.74496 0.61958 0.77255 0.48576 0.95510
87  2.52616 4.43809 3.23851 3.06762 4.22632 3.13794 4.21697 3.72990 3.14243 3.41577 4.36624 3.34888 1.02423 3.51864 3.43129 2.57508 2.98769 3.29832 5.55894 4.30616   99 - -
    2.68618 4.42225 2.77519 2.73123 3.46354 2.40513 3.72494 3.29354 2.67741 2.69355 4.24690 2.90347 2.73739 3.18146 2.89801 2.37887 2.77519 2.98518 4.58477 3.61503
    0.02696 4.02262 4.74496 0.61958 0.77255 0.48576 0.95510
88  2.75470 4.49702 3.58748 2.99219 3.52024 3.68998 3.88402 2.34992 2.65776 2.03049 3.44264 3.39519 4.05898 3.15731 1.97673 2.96708 2.98267 2.51467 5.04511 3.80701  100 - -
    2.68618 4.42225 2.77519 2.73123 3.46354 2.40513 3.72494 3.29354 2.67741 2.69355 4.24690 2.90347 2.73739 3.18146 2.89801 2.37887 2.77519 2.98518 4.58477 3.61503
    0.02696 4.02262 4.74496 0.61958 0.77255 0.48576 0.95510
89  3.10771 4.45874 4.68120 4.15161 3.36896 4.38313 4.82125 1.83312 4.01567 1.46510 3.19678 4.40195 4.67218 4.25614 4.20876 3.74180 3.35950 1.25833 5.32748 4.15040  101 - -
    2.68618 4.42225 2.77519 2.73123 3.46354 2.40513 3.72494 3.29354 2.67741 2.69355 4.24690 2.90347 2.73739 3.18146 2.89801 2.37887 2.77519 2.98518 4.58477 3.61503
    0.02696 4.02262 4.74496 0.61958 0.77255 0.48576 0.95510
90  2.21700 4.20982 3.38425 3.11040 4.28641 2.97978 4.20097 3.63603 3.14609 3.37605 4.23061 3.29506 3.72025 3.46786 3.45501 1.14168 2.32869 3.13282 5.64011 4.40878  102 - -
    2.68618 4.42225 2.77519 2.73123 3.46354 2.40513 3.72494 3.29354 2.67741 2.69355 4.24690 2.90347 2.73739 3.18146 2.89801 2.37887 2.77519 2.98518 4.58477 3.61503
    0.02696 4.02262 4.74496 0.61958 0.77255 0.48576 0.95510
91  0.88356 4.21198 3.52839 3.32006 4.28353 2.83291 4.35865 3.52879 3.36974 3.36755 4.28205 3.43317 3.77304 3.67878 3.62827 2.51503 2.81796 3.07330 5.65280 4.46827  103 - -
    2.68618 4.42225 2.77519 2.73123 3.46354 2.40513 3.72494 3.29354 2.67741 2.69355 4.24690 2.90347 2.73739 3.18146 2.89801 2.37887 2.77519 2.98518 4.58477 3.61503
    0.02696 4.02262 4.74496 0.61958 0.77255 0.48576 0.95510
92  2.89870 5.28625 2.28738 1.15446 4.59342 3.29409 3.76406 4.06859 2.64285 3.61654 4.47537 2.79924 3.87247 2.93158 3.00362 2.83262 3.17576 3.68859 5.77155 4.35608  104 - -
    2.68618 4.42225 2.77519 2.73123 3.46354 2.40513 3.72494 3.29354 2.67741 2.69355 4.24690 2.90347 2.73739 3.18146 2.89801 2.37887 2.77519 2.98518 4.58477 3.61503
    0.02696 4.02262 4.74496 0.61958 0.77255 0.48576 0.95510
93  3.32779 4.70936 4.63990 4.16729 2.76600 4.41443 4.61773 2.27565 3.99584 0.80986 2.97704 4.41297 4.68477 4.18479 4.14582 3.82145 3.57394 2.38831 4.94145 3.61905  105 - -
    2.68618 4.42225 2.77519 2.73123 3.46354 2.40513 3.72494 3.29354 2.67741 2.69355 4.24690 2.90347 2.73739 3.18146 2.89801 2.37887 2.77519 2.98518 4.58477 3.61503
    0.02696 4.02262 4.74496 0.61958 0.77255 0.48576 0.95510
94  2.96212 4.69205 3.58591 3.44827 4.42684 3.37117 4.51157 4.01315 3.51887 3.62279 4.68053 3.73880 0.59042 3.90306 3.74552 3.13480 3.41594 3.64102 5.57002 4.55027  106 - -
    2.68618 4.42225 2.77519 2.73123 3.46354 2.40513 3.72494 3.29354 2.67741 2.69355 4.24690 2.90347 2.73739 3.18146 2.89801 2.37887 2.77519 2.98518 4.58477 3.61503
```

	0.02696	4.02262	4.74496	0.61958	0.77255	0.48576	0.95510														
95	2.65219	4.95592	2.62692	1.68444	4.30124	2.74063	3.66268	3.74091	2.48686	3.21150	4.11369	2.85430	3.43828	2.80748	2.96773	2.60141	2.70018	3.36711	5.51318	4.13874	107 - -
	2.68613	4.42227	2.77522	2.73121	3.46356	2.40507	3.72497	3.29356	2.67743	2.69354	4.24692	2.90349	2.73742	3.18142	2.89794	2.37889	2.77522	2.98521	4.58479	3.61505	
	0.11455	3.02711	2.81713	0.95317	0.48697	0.48576	0.95510														
96	2.28814	4.33090	3.17575	2.79628	4.02893	2.93342	3.68262	3.42996	2.81725	3.10060	3.94543	3.14353	3.74413	3.15540	3.18634	2.08022	1.68675	3.03391	5.37129	4.11214	113 - -
	2.68618	4.42225	2.77519	2.73123	3.46354	2.40513	3.72494	3.29354	2.67741	2.69355	4.24690	2.90347	2.73739	3.18146	2.89801	2.37887	2.77519	2.98518	4.58477	3.61503	
	0.02843	3.97018	4.69253	0.61958	0.77255	0.45411	1.00788														
97	2.74140	4.58369	3.29463	3.10313	4.53895	0.68244	4.40968	4.09340	3.42546	3.75580	4.69783	3.50567	3.94887	3.74626	3.70160	2.89623	3.21533	3.60931	5.67744	4.61396	114 - -
	2.68618	4.42225	2.77519	2.73123	3.46354	2.40513	3.72494	3.29354	2.67741	2.69355	4.24690	2.90347	2.73739	3.18146	2.89801	2.37887	2.77519	2.98518	4.58477	3.61503	
	0.02696	4.02262	4.74496	0.61958	0.77255	0.48576	0.95510														
98	2.87240	5.16455	2.39918	1.12397	4.53346	3.27752	3.81279	3.98750	2.69176	3.58874	4.47755	2.84449	3.88136	2.99498	3.14617	2.66603	3.17787	3.61504	5.75291	4.34606	115 - -
	2.68618	4.42225	2.77519	2.73123	3.46354	2.40513	3.72494	3.29354	2.67741	2.69355	4.24690	2.90347	2.73739	3.18146	2.89801	2.37887	2.77519	2.98518	4.58477	3.61503	
	0.02696	4.02262	4.74496	0.61958	0.77255	0.48576	0.95510														
99	3.18120	5.00542	3.68780	3.17038	4.39834	3.63746	3.94268	3.95237	2.31116	3.44111	4.43934	3.51762	4.13451	3.15727	0.77162	3.25933	3.43807	3.67032	5.42160	4.29519	116 - -
	2.68618	4.42225	2.77519	2.73123	3.46354	2.40513	3.72494	3.29354	2.67741	2.69355	4.24690	2.90347	2.73739	3.18146	2.89801	2.37887	2.77519	2.98518	4.58477	3.61503	
	0.02696	4.02262	4.74496	0.61958	0.77255	0.48576	0.95510														
100	3.45487	4.81335	4.44471	4.18978	0.79468	4.10525	3.95198	2.96241	4.09358	2.29884	3.67170	4.19112	4.56204	4.22068	4.17611	3.71567	3.75688	2.96378	4.16042	2.51021	117 - -
	2.68618	4.42225	2.77519	2.73123	3.46354	2.40513	3.72494	3.29354	2.67741	2.69355	4.24690	2.90347	2.73739	3.18146	2.89801	2.37887	2.77519	2.98518	4.58477	3.61503	
	0.02696	4.02262	4.74496	0.61958	0.77255	0.48576	0.95510														
101	2.93884	5.28356	2.38424	1.12246	4.56270	3.31397	3.78387	4.05273	2.62255	3.58831	4.48514	2.83271	3.89833	2.77076	3.06132	2.87680	3.21804	3.69365	5.75043	4.34836	118 - -
	2.68618	4.42225	2.77519	2.73123	3.46354	2.40513	3.72494	3.29354	2.67741	2.69355	4.24690	2.90347	2.73739	3.18146	2.89801	2.37887	2.77519	2.98518	4.58477	3.61503	
	0.02696	4.02262	4.74496	0.61958	0.77255	0.48576	0.95510														
102	2.28395	4.33971	3.28698	3.16844	4.44460	0.91026	4.34967	3.88261	3.36162	3.60445	4.50514	3.38042	3.80439	3.65957	3.64342	2.59640	2.92752	3.35318	5.72734	4.55379	119 - -
	2.68618	4.42225	2.77519	2.73123	3.46354	2.40513	3.72494	3.29354	2.67741	2.69355	4.24690	2.90347	2.73739	3.18146	2.89801	2.37887	2.77519	2.98518	4.58477	3.61503	
	0.02696	4.02262	4.74496	0.61958	0.77255	0.48576	0.95510														
103	3.23968	4.56415	4.83033	4.28832	3.21358	4.52719	4.89761	1.61296	4.14381	1.08454	3.01463	4.54523	4.74794	4.31649	4.29619	3.88701	3.47620	1.90106	5.27031	4.15483	120 - -
	2.68618	4.42225	2.77519	2.73123	3.46354	2.40513	3.72494	3.29354	2.67741	2.69355	4.24690	2.90347	2.73739	3.18146	2.89801	2.37887	2.77519	2.98518	4.58477	3.61503	
	0.02696	4.02262	4.74496	0.61958	0.77255	0.48576	0.95510														
104	3.24147	4.64085	4.64480	4.13885	3.13357	4.38828	4.75888	2.10411	3.92543	0.87652	2.99067	4.40558	4.67153	4.17255	4.09458	3.77006	3.49695	2.10383	5.17787	3.99817	121 - -
	2.68618	4.42225	2.77519	2.73123	3.46354	2.40513	3.72494	3.29354	2.67741	2.69355	4.24690	2.90347	2.73739	3.18146	2.89801	2.37887	2.77519	2.98518	4.58477	3.61503	
	0.02696	4.02262	4.74496	0.61958	0.77255	0.48576	0.95510														
105	2.96212	4.69205	3.58591	3.44827	4.42684	3.37117	4.51157	4.01315	3.51887	3.62279	4.68053	3.73880	0.59042	3.90306	3.74552	3.13480	3.41594	3.64102	5.57002	4.55027	122 - -
	2.68618	4.42225	2.77519	2.73123	3.46354	2.40513	3.72494	3.29354	2.67741	2.69355	4.24690	2.90347	2.73739	3.18146	2.89801	2.37887	2.77519	2.98518	4.58477	3.61503	
	0.02696	4.02262	4.74496	0.61958	0.77255	0.48576	0.95510														
106	2.96212	4.69205	3.58591	3.44827	4.42684	3.37117	4.51157	4.01315	3.51887	3.62279	4.68053	3.73880	0.59042	3.90306	3.74552	3.13480	3.41594	3.64102	5.57002	4.55027	123 - -
	2.68618	4.42225	2.77519	2.73123	3.46354	2.40513	3.72494	3.29354	2.67741	2.69355	4.24690	2.90347	2.73739	3.18146	2.89801	2.37887	2.77519	2.98518	4.58477	3.61503	
	0.02696	4.02262	4.74496	0.61958	0.77255	0.48576	0.95510														
107	2.31968	4.31660	4.27526	3.75994	3.57402	3.97700	4.54479	1.86385	3.65554	2.19576	3.43669	4.01686	4.39680	3.95243	3.91027	3.33030	3.12713	1.20795	5.32010	4.10529	124 - -
	2.68618	4.42225	2.77519	2.73123	3.46354	2.40513	3.72494	3.29354	2.67741	2.69355	4.24690	2.90347	2.73739	3.18146	2.89801	2.37887	2.77519	2.98518	4.58477	3.61503	
	0.02696	4.02262	4.74496	0.61958	0.77255	0.48576	0.95510														
108	2.00555	3.64933	3.98160	3.49800	3.63901	3.34810	4.25408	2.50075	3.40042	2.60819	3.58499	3.62731	3.95486	3.67222	3.64316	2.55010	2.81787	1.43376	5.17237	3.97177	125 - -
	2.68618	4.42225	2.77519	2.73123	3.46354	2.40513	3.72494	3.29354	2.67741	2.69355	4.24690	2.90347	2.73739	3.18146	2.89801	2.37887	2.77519	2.98518	4.58477	3.61503	
	0.02696	4.02262	4.74496	0.61958	0.77255	0.48576	0.95510														
109	2.13202	4.50055	3.16694	2.54177	3.71174	3.43871	3.70208	3.05372	2.58708	2.72177	3.46544	3.08381	3.04005	2.92481	3.00350	2.54183	2.54965	2.75430	5.08100	3.68158	126 - -
	2.68618	4.42225	2.77519	2.73123	3.46354	2.40513	3.72494	3.29354	2.67741	2.69355	4.24690	2.90347	2.73739	3.18146	2.89801	2.37887	2.77519	2.98518	4.58477	3.61503	
	0.02696	4.02262	4.74496	0.61958	0.77255	0.48576	0.95510														
110	1.20300	4.16727	3.41926	3.17206	4.32646	1.99262	4.26035	3.70110	3.26544	3.42843	4.26362	3.31681	3.70147	3.54192	3.56976	2.34877	2.72401	3.15937	5.67735	4.46922	127 - -
	2.68618	4.42225	2.77519	2.73123	3.46354	2.40513	3.72494	3.29354	2.67741	2.69355	4.24690	2.90347	2.73739	3.18146	2.89801	2.37887	2.77519	2.98518	4.58477	3.61503	
	0.02696	4.02262	4.74496	0.61958	0.77255	0.48576	0.95510														
111	2.96212	4.69205	3.58591	3.44827	4.42684	3.37117	4.51157	4.01315	3.51887	3.62279	4.68053	3.73880	0.59042	3.90306	3.74552	3.13480	3.41594	3.64102	5.57002	4.55027	128 - -
	2.68618	4.42225	2.77519	2.73123	3.46354	2.40513	3.72494	3.29354	2.67741	2.69355	4.24690	2.90347	2.73739	3.18146	2.89801	2.37887	2.77519	2.98518	4.58477	3.61503	
	0.02696	4.02262	4.74496	0.61958	0.77255	0.48576	0.95510														
112	1.64158	2.87951	3.77264	3.28641	3.74519	3.14364	4.12495	3.01570	3.21488	2.81578	3.70406	3.43437	3.79244	3.48786	3.49682	2.20493	2.33512	2.44775	5.19129	3.99235	129 - -
	2.68618	4.42225	2.77519	2.73123	3.46354	2.40513	3.72494	3.29354	2.67741	2.69355	4.24690	2.90347	2.73739	3.18146	2.89801	2.37887	2.77519	2.98518	4.58477	3.61503	
	0.02696	4.02262	4.74496	0.61958	0.77255	0.48576	0.95510														
113	3.45487	4.81335	4.44471	4.18978	0.79468	4.10525	3.95198	2.96241	4.09358	2.29884	3.67170	4.19112	4.56204	4.22068	4.17611	3.71567	3.75688	2.96378	4.16042	2.51021	130 - -
	2.68618	4.42225	2.77519	2.73123	3.46354	2.40513	3.72494	3.29354	2.67741	2.69355	4.24690	2.90347	2.73739	3.18146	2.89801	2.37887	2.77519	2.98518	4.58477	3.61503	
	0.02696	4.02262	4.74496	0.61958	0.77255	0.48576	0.95510														
114	1.10765	4.14602	3.52909	3.23897	4.24736	2.95997	4.26460	3.54206	3.25943	3.32465	4.18178	3.35509	3.71199	3.55564	3.54801	2.05437	2.54140	3.05252	5.62293	4.41973	131 - -
	2.68618	4.42225	2.77519	2.73123	3.46354	2.40513	3.72494	3.29354	2.67741	2.69355	4.24690	2.90347	2.73739	3.18146	2.89801	2.37887	2.77519	2.98518	4.58477	3.61503	
	0.02696	4.02262	4.74496	0.61958	0.77255	0.48576	0.95510														
115	3.19003	4.50278	4.83006	4.30409	3.34729	4.51744	4.94767	1.18640	4.16160	1.53232	3.15764	4.54821	4.76672	4.37976	4.33261	3.88731	3.43647	1.73357	5.37013	4.20960	132 - -
	2.68618	4.42225	2.77519	2.73123	3.46354	2.40513	3.72494	3.29354	2.67741	2.69355	4.24690	2.90347	2.73739	3.18146	2.89801	2.37887	2.77519	2.98518	4.58477	3.61503	
	0.02696	4.02262	4.74496	0.61958	0.77255	0.48576	0.95510														
116	3.18120	5.00542	3.68780	3.17038	4.39834	3.63746	3.94268	3.95237	2.31116	3.44111	4.43934	3.51762	4.13451	3.15727	0.77162	3.25933	3.43807	3.67032	5.42160	4.29519	133 - -
	2.68618	4.42225	2.77519	2.73123	3.46354	2.40513	3.72494	3.29354	2.67741	2.69355	4.24690	2.90347	2.73739	3.18146	2.89801	2.37887	2.77519	2.98518	4.58477	3.61503	
	0.02696	4.02262	4.74496	0.61958	0.77255	0.48576	0.95510														
117	3.04481	5.12346	3.32454	2.79158	4.54915	3.61845	3.68110	3.93741	1.04397	3.41560	4.30981	3.20479	4.03898	2.83125	2.11594	3.05269	3.24275	3.62293	5.46930	4.28059	134 - -
	2.68618	4.42225	2.77519	2.73123	3.46354	2.40513	3.72494	3.29354	2.67741	2.69355	4.24690	2.90347	2.73739	3.18146	2.89801	2.37887	2.77519	2.98518	4.58477	3.61503	
	0.02696	4.02262	4.74496	0.61958	0.77255	0.48576	0.95510														
118	2.71376	4.85168	3.02402	2.54322	4.18588	3.43934	3.51627	3.60062	2.20246	3.17450	4.01212	3.02248	2.06530	2.81759	2.23169	2.66462	2.95395	3.27258	5.36260	4.07380	135 - -
	2.68618	4.42225	2.77519	2.73123	3.46354	2.40513	3.72494	3.29354	2.67741	2.69355	4.24690	2.90347	2.73739	3.18146	2.89801	2.37887	2.77519	2.98518	4.58477	3.61503	
	0.02696	4.02262	4.74496	0.61958	0.77255	0.48576	0.95510														
119	0.81728	4.27297	3.62041	3.42136	4.20706	3.09296	4.42492	3.35544	3.44162	3.22989	4.24718	3.53130	3.84663	3.76944	3.68019	2.62296	2.91086	2.98372	5.61916	4.43687	136 - -
	2.68618	4.42225	2.77519	2.73123	3.46354	2.40513	3.72494	3.29354	2.67741	2.69355	4.24690	2.90347	2.73739	3.18146	2.89801	2.37887	2.77519	2.98518	4.58477	3.61503	

	0.02696	4.02262	4.74496	0.61958	0.77255	0.48576	0.95510													
120	2.50248	4.18529	3.70776	3.14478	3.22355	3.56819	3.93459	2.33797	3.06228	2.29683	3.30514	3.47660	4.00783	3.18428	3.35220	2.63381	2.82923	1.73787	4.83230	3.61500	137 - -
	2.68618	4.42225	2.77519	2.73123	3.46354	2.40513	3.72494	3.29354	2.67741	2.69355	4.24690	2.90347	2.73739	3.18146	2.89801	2.37887	2.77519	2.98518	4.58477	3.61503	
	0.02696	4.02262	4.74496	0.61958	0.77255	0.48576	0.95510													
121	1.96173	4.53601	3.14185	2.59872	3.80683	2.93498	3.70327	3.19221	2.51487	2.76719	3.49472	3.01024	3.82746	2.90970	2.77694	2.48230	2.80057	2.86249	5.14942	3.86638	138 - -
	2.68618	4.42225	2.77519	2.73123	3.46354	2.40513	3.72494	3.29354	2.67741	2.69355	4.24690	2.90347	2.73739	3.18146	2.89801	2.37887	2.77519	2.98518	4.58477	3.61503	
	0.02696	4.02262	4.74496	0.61958	0.77255	0.48576	0.95510													
122	2.80053	4.28588	4.15671	3.62202	3.45066	3.92299	4.36999	1.81150	3.51225	2.17258	3.34900	3.89814	3.44705	3.80092	3.76245	3.25008	3.06496	1.31665	5.13956	3.92923	139 - -
	2.68618	4.42225	2.77519	2.73123	3.46354	2.40513	3.72494	3.29354	2.67741	2.69355	4.24690	2.90347	2.73739	3.18146	2.89801	2.37887	2.77519	2.98518	4.58477	3.61503	
	0.02696	4.02262	4.74496	0.61958	0.77255	0.48576	0.95510													
123	2.90771	4.34739	4.19218	3.66934	1.56893	3.92724	3.41280	2.23297	3.53737	2.25267	3.37167	3.79240	4.27181	3.71450	3.70451	3.22844	3.13584	2.58391	4.11860	2.35511	140 - -
	2.68618	4.42225	2.77519	2.73123	3.46354	2.40513	3.72494	3.29354	2.67741	2.69355	4.24690	2.90347	2.73739	3.18146	2.89801	2.37887	2.77519	2.98518	4.58477	3.61503	
	0.02696	4.02262	4.74496	0.61958	0.77255	0.48576	0.95510													
124	2.22352	4.35819	3.26663	2.79107	3.96812	2.89434	3.88316	3.35665	2.77947	3.02825	3.69045	3.10867	3.03412	3.10897	3.16965	2.39585	1.76523	2.98763	5.31468	4.05389	141 - -
	2.68618	4.42225	2.77519	2.73123	3.46354	2.40513	3.72494	3.29354	2.67741	2.69355	4.24690	2.90347	2.73739	3.18146	2.89801	2.37887	2.77519	2.98518	4.58477	3.61503	
	0.02696	4.02262	4.74496	0.61958	0.77255	0.48576	0.95510													
125	3.28700	4.68723	4.60986	4.13576	3.09929	4.38586	4.72763	2.12651	3.89188	0.82702	2.99679	4.40310	4.67769	4.16657	4.05383	3.79789	3.54357	2.27778	5.13226	3.91227	142 - -
	2.68618	4.42225	2.77519	2.73123	3.46354	2.40513	3.72494	3.29354	2.67741	2.69355	4.24690	2.90347	2.73739	3.18146	2.89801	2.37887	2.77519	2.98518	4.58477	3.61503	
	0.02696	4.02262	4.74496	0.61958	0.77255	0.48576	0.95510													
126	2.37076	5.19624	1.94110	2.00711	4.49750	3.22083	3.59647	3.97530	2.49969	3.48861	4.27793	2.58374	3.79886	2.63938	3.02216	2.49147	2.94990	3.56886	5.65764	4.22997	143 - -
	2.68618	4.42225	2.77519	2.73123	3.46354	2.40513	3.72494	3.29354	2.67741	2.69355	4.24690	2.90347	2.73739	3.18146	2.89801	2.37887	2.77519	2.98518	4.58477	3.61503	
	0.02696	4.02262	4.74496	0.61958	0.77255	0.48576	0.95510													
127	2.72767	5.17971	1.52695	2.24587	4.49888	3.30256	3.69017	3.97896	2.51918	3.50774	4.32425	2.73770	3.82984	2.80546	2.45935	2.73079	3.04205	3.58530	5.66960	4.25696	144 - -
	2.68618	4.42225	2.77519	2.73123	3.46354	2.40513	3.72494	3.29354	2.67741	2.69355	4.24690	2.90347	2.73739	3.18146	2.89801	2.37887	2.77519	2.98518	4.58477	3.61503	
	0.02696	4.02262	4.74496	0.61958	0.77255	0.48576	0.95510													
128	3.47032	4.89825	4.06283	3.82242	2.31343	4.00733	3.69832	3.45928	3.67573	2.87390	4.11913	3.91273	4.47865	3.94536	3.82775	3.59122	3.75827	3.33721	3.96223	0.74848	145 - -
	2.68618	4.42225	2.77519	2.73123	3.46354	2.40513	3.72494	3.29354	2.67741	2.69355	4.24690	2.90347	2.73739	3.18146	2.89801	2.37887	2.77519	2.98518	4.58477	3.61503	
	0.02696	4.02262	4.74496	0.61958	0.77255	0.48576	0.95510													
129	2.84338	4.37881	4.51463	4.04635	3.61901	4.13984	4.81976	1.74356	3.91852	2.19866	3.45714	4.26155	4.56425	4.22974	4.14497	3.53584	3.25747	0.98864	5.49463	4.26683	146 - -
	2.68618	4.42225	2.77519	2.73123	3.46354	2.40513	3.72494	3.29354	2.67741	2.69355	4.24690	2.90347	2.73739	3.18146	2.89801	2.37887	2.77519	2.98518	4.58477	3.61503	
	0.02696	4.02262	4.74496	0.61958	0.77255	0.48576	0.95510													
130	1.99628	4.78509	2.76794	2.29789	4.04057	3.38823	3.56230	3.39105	2.42328	3.05996	3.87564	2.83216	3.79770	2.77524	2.73992	2.54714	2.77462	2.99407	5.30250	3.96740	147 - -
	2.68618	4.42225	2.77519	2.73123	3.46354	2.40513	3.72494	3.29354	2.67741	2.69355	4.24690	2.90347	2.73739	3.18146	2.89801	2.37887	2.77519	2.98518	4.58477	3.61503	
	0.02696	4.02262	4.74496	0.61958	0.77255	0.48576	0.95510													
131	1.95523	5.02157	2.06482	2.24528	4.34819	3.30602	3.67414	3.79200	2.52572	3.35635	4.16645	2.82708	3.80557	2.53938	3.02271	2.59952	2.88465	3.41743	5.56149	4.17263	148 - -
	2.68618	4.42225	2.77519	2.73123	3.46354	2.40513	3.72494	3.29354	2.67741	2.69355	4.24690	2.90347	2.73739	3.18146	2.89801	2.37887	2.77519	2.98518	4.58477	3.61503	
	0.02696	4.02262	4.74496	0.61958	0.77255	0.48576	0.95510													
132	2.51310	4.76602	2.67222	2.40602	4.24302	1.69815	3.76543	3.66299	2.58014	3.27025	4.10691	2.91371	3.82079	2.94411	2.70076	2.67406	2.93474	3.29353	5.49558	4.17032	149 - -
	2.68618	4.42225	2.77519	2.73123	3.46354	2.40513	3.72494	3.29354	2.67741	2.69355	4.24690	2.90347	2.73739	3.18146	2.89801	2.37887	2.77519	2.98518	4.58477	3.61503	
	0.02696	4.02262	4.74496	0.61958	0.77255	0.48576	0.95510													
133	2.81289	4.25577	4.26395	3.70905	3.37540	3.97629	4.37541	1.38820	3.58650	2.12835	2.80316	3.95915	4.33574	3.85056	3.80495	3.09585	2.95165	1.80418	5.06904	3.87674	150 - -
	2.68618	4.42225	2.77519	2.73123	3.46354	2.40513	3.72494	3.29354	2.67741	2.69355	4.24690	2.90347	2.73739	3.18146	2.89801	2.37887	2.77519	2.98518	4.58477	3.61503	
	0.02696	4.02262	4.74496	0.61958	0.77255	0.48576	0.95510													
134	3.20687	4.59535	4.62664	4.06447	3.10281	4.39404	4.68800	2.12086	3.89636	1.29935	1.60876	4.35357	4.62613	4.09280	4.06808	3.72870	3.43479	2.24233	5.12496	4.01622	151 - -
	2.68618	4.42225	2.77519	2.73123	3.46354	2.40513	3.72494	3.29354	2.67741	2.69355	4.24690	2.90347	2.73739	3.18146	2.89801	2.37887	2.77519	2.98518	4.58477	3.61503	
	0.02696	4.02262	4.74496	0.61958	0.77255	0.48576	0.95510													
135	2.44054	4.56869	3.03964	2.54197	3.74133	3.35910	3.68746	3.17116	2.54158	2.79578	3.71039	3.03919	3.76853	2.79014	2.92623	2.33935	2.10346	2.89832	5.15802	3.86654	152 - -
	2.68618	4.42225	2.77519	2.73123	3.46354	2.40513	3.72494	3.29354	2.67741	2.69355	4.24690	2.90347	2.73739	3.18146	2.89801	2.37887	2.77519	2.98518	4.58477	3.61503	
	0.02696	4.02262	4.74496	0.61958	0.77255	0.48576	0.95510													
136	1.91982	4.73368	2.81825	2.34527	4.03372	3.36179	3.65584	3.44597	2.47390	3.05932	3.88132	2.94793	3.38686	2.79315	2.83078	2.45717	2.75284	3.04307	5.30989	3.98275	153 - -
	2.68618	4.42225	2.77519	2.73123	3.46354	2.40513	3.72494	3.29354	2.67741	2.69355	4.24690	2.90347	2.73739	3.18146	2.89801	2.37887	2.77519	2.98518	4.58477	3.61503	
	0.02696	4.02262	4.74496	0.61958	0.77255	0.48576	0.95510													
137	2.24661	4.54238	2.85072	2.28302	4.02119	2.94611	3.60638	3.43839	2.36413	2.99212	3.70721	2.93094	3.69679	2.73173	2.71012	2.59210	2.67775	3.04616	5.28182	3.94627	154 - -
	2.68618	4.42225	2.77519	2.73123	3.46354	2.40513	3.72494	3.29354	2.67741	2.69355	4.24690	2.90347	2.73739	3.18146	2.89801	2.37887	2.77519	2.98518	4.58477	3.61503	
	0.02696	4.02262	4.74496	0.61958	0.77255	0.48576	0.95510													
138	2.54168	4.95791	2.77163	2.32179	4.27340	3.38731	3.51906	3.69538	2.36443	3.26234	4.09312	2.92557	3.85186	1.69990	2.75052	2.70673	2.83386	3.35279	5.45743	4.12706	155 - -
	2.68618	4.42225	2.77519	2.73123	3.46354	2.40513	3.72494	3.29354	2.67741	2.69355	4.24690	2.90347	2.73739	3.18146	2.89801	2.37887	2.77519	2.98518	4.58477	3.61503	
	0.02696	4.02262	4.74496	0.61958	0.77255	0.48576	0.95510													
139	1.01938	4.25231	3.30658	3.08348	4.22286	2.97446	4.19946	3.54735	3.15840	3.33727	4.21571	3.00083	3.75767	3.48451	3.46025	2.50119	2.79807	3.09128	5.59350	4.34834	156 - -
	2.68618	4.42225	2.77519	2.73123	3.46354	2.40513	3.72494	3.29354	2.67741	2.69355	4.24690	2.90347	2.73739	3.18146	2.89801	2.37887	2.77519	2.98518	4.58477	3.61503	
	0.02696	4.02262	4.74496	0.61958	0.77255	0.48576	0.95510													
140	2.35005	5.00569	2.33006	2.06359	4.30733	2.96858	3.60787	3.76058	2.39371	3.30284	4.08770	2.83611	3.78036	2.69744	2.85836	2.42731	2.83475	3.22593	5.49040	4.10339	157 - -
	2.68618	4.42225	2.77519	2.73123	3.46354	2.40513	3.72494	3.29354	2.67741	2.69355	4.24690	2.90347	2.73739	3.18146	2.89801	2.37887	2.77519	2.98518	4.58477	3.61503	
	0.02696	4.02262	4.74496	0.61958	0.77255	0.48576	0.95510													
141	2.21356	4.11875	3.91952	3.34391	2.50129	3.68932	3.99137	2.25213	3.24637	2.08080	3.19618	3.61267	4.05898	3.49297	3.47853	2.91790	2.71708	2.14215	4.74229	3.43428	158 - -
	2.68618	4.42225	2.77519	2.73123	3.46354	2.40513	3.72494	3.29354	2.67741	2.69355	4.24690	2.90347	2.73739	3.18146	2.89801	2.37887	2.77519	2.98518	4.58477	3.61503	
	0.02696	4.02262	4.74496	0.61958	0.77255	0.48576	0.95510													
142	3.27669	4.70308	4.53056	4.08115	3.09546	4.31347	4.67589	2.26615	3.81016	0.80315	2.95886	4.34544	4.63914	4.12099	3.97320	3.74867	3.54303	2.35614	5.10714	3.86687	159 - -
	2.68618	4.42225	2.77519	2.73123	3.46354	2.40513	3.72494	3.29354	2.67741	2.69355	4.24690	2.90347	2.73739	3.18146	2.89801	2.37887	2.77519	2.98518	4.58477	3.61503	
	0.02696	4.02262	4.74496	0.61958	0.77255	0.48576	0.95510													
143	2.49352	4.11169	3.24134	2.66319	3.79189	3.47228	3.64977	3.08723	2.45385	2.81778	3.68846	3.11714	3.87423	2.80121	1.90656	2.72390	2.84836	2.64182	5.12388	3.85821	160 - -
	2.68618	4.42225	2.77519	2.73123	3.46354	2.40513	3.72494	3.29354	2.67741	2.69355	4.24690	2.90347	2.73739	3.18146	2.89801	2.37887	2.77519	2.98518	4.58477	3.61503	
	0.02696	4.02262	4.74496	0.61958	0.77255	0.48576	0.95510													
144	2.44458	4.46018	2.79365	2.30427	4.11989	3.25868	3.51466	3.50701	2.32878	2.98160	3.86476	2.84923	3.78310	2.57255	2.30267	2.59478	2.81173	3.16802	5.33947	3.98980	161 - -
	2.68618	4.42225	2.77519	2.73123	3.46354	2.40513	3.72494	3.29354	2.67741	2.69355	4.24690	2.90347	2.73739	3.18146	2.89801	2.37887	2.77519	2.98518	4.58477	3.61503	

	0.02696	4.02262	4.74496	0.61958	0.77255	0.48576	0.95510													
145	1.00844	4.18463	3.46597	3.23808	4.31702	2.36946	4.30669	3.64577	3.31963	3.41393	4.27803	3.36765	3.73054	3.60562	3.60371	2.45380	2.76392	3.13602	5.66954	4.47893	162 - -
	2.68618	4.42225	2.77519	2.73123	3.46354	2.40513	3.72494	3.29354	2.67741	2.69355	4.24690	2.90347	2.73739	3.18146	2.89801	2.37887	2.77519	2.98518	4.58477	3.61503	
	0.02696	4.02262	4.74496	0.61958	0.77255	0.48576	0.95510													
146	3.03454	4.41781	4.61544	4.12674	3.55141	4.26975	4.86759	1.73697	3.98028	2.09822	3.32328	4.35454	4.64406	4.28626	4.19828	3.65866	3.31802	0.97242	5.47279	4.24020	163 - -
	2.68618	4.42225	2.77519	2.73123	3.46354	2.40513	3.72494	3.29354	2.67741	2.69355	4.24690	2.90347	2.73739	3.18146	2.89801	2.37887	2.77519	2.98518	4.58477	3.61503	
	0.02696	4.02262	4.74496	0.61958	0.77255	0.48576	0.95510													
147	2.18453	4.64369	3.04915	2.49845	3.85323	3.39677	3.27566	3.16404	2.43237	2.88787	3.57110	3.00933	3.82576	2.80855	2.44472	2.58611	2.81053	2.80490	5.16823	3.81737	164 - -
	2.68618	4.42225	2.77519	2.73123	3.46354	2.40513	3.72494	3.29354	2.67741	2.69355	4.24690	2.90347	2.73739	3.18146	2.89801	2.37887	2.77519	2.98518	4.58477	3.61503	
	0.02696	4.02262	4.74496	0.61958	0.77255	0.48576	0.95510													
148	2.42477	5.12404	2.29203	1.76670	4.43970	3.08702	3.64099	3.90871	2.47737	3.43305	4.22123	2.63709	3.79271	2.77263	2.95876	2.50973	2.80557	3.50821	5.60855	4.19525	165 - -
	2.68618	4.42225	2.77519	2.73123	3.46354	2.40513	3.72494	3.29354	2.67741	2.69355	4.24690	2.90347	2.73739	3.18146	2.89801	2.37887	2.77519	2.98518	4.58477	3.61503	
	0.02696	4.02262	4.74496	0.61958	0.77255	0.48576	0.95510													
149	3.04214	5.10914	3.43301	2.81133	4.44199	3.66346	3.31765	3.88614	1.87400	3.35537	4.23513	3.21148	4.04454	2.78605	1.19603	3.05314	3.21768	3.58093	5.39235	4.18518	166 - -
	2.68618	4.42225	2.77519	2.73123	3.46354	2.40513	3.72494	3.29354	2.67741	2.69355	4.24690	2.90347	2.73739	3.18146	2.89801	2.37887	2.77519	2.98518	4.58477	3.61503	
	0.02696	4.02262	4.74496	0.61958	0.77255	0.48576	0.95510													
150	2.31083	4.93962	2.93806	2.40822	4.23487	3.42972	3.46622	3.65721	2.09342	3.06186	4.00571	2.91569	3.82102	2.29352	2.34990	2.63913	2.87883	3.30603	5.38625	4.05703	167 - -
	2.68618	4.42225	2.77519	2.73123	3.46354	2.40513	3.72494	3.29354	2.67741	2.69355	4.24690	2.90347	2.73739	3.18146	2.89801	2.37887	2.77519	2.98518	4.58477	3.61503	
	0.02696	4.02262	4.74496	0.61958	0.77255	0.48576	0.95510													
151	2.85426	4.85176	2.80169	2.70353	4.23951	3.28976	4.05172	3.98473	2.96940	3.61516	4.57129	0.91734	3.95002	3.32667	3.32750	2.91694	3.24227	3.59163	5.52835	4.17856	168 - -
	2.68618	4.42225	2.77519	2.73123	3.46354	2.40513	3.72494	3.29354	2.67741	2.69355	4.24690	2.90347	2.73739	3.18146	2.89801	2.37887	2.77519	2.98518	4.58477	3.61503	
	0.02696	4.02262	4.74496	0.61958	0.77255	0.48576	0.95510													
152	3.12892	4.44458	4.81217	4.31197	3.51780	4.47015	5.01554	1.09984	4.18591	1.97019	3.32600	4.53903	4.76984	4.44670	4.37987	3.85642	3.39226	1.44382	5.50729	4.30911	169 - -
	2.68618	4.42225	2.77519	2.73123	3.46354	2.40513	3.72494	3.29354	2.67741	2.69355	4.24690	2.90347	2.73739	3.18146	2.89801	2.37887	2.77519	2.98518	4.58477	3.61503	
	0.02696	4.02262	4.74496	0.61958	0.77255	0.48576	0.95510													
153	3.19779	4.62134	4.56816	4.09107	3.16038	4.29710	4.72639	2.16792	3.88196	0.89667	3.03277	4.34722	4.62517	4.14718	4.05476	3.69942	3.46738	2.03918	5.17879	3.98466	170 - -
	2.68618	4.42225	2.77519	2.73123	3.46354	2.40513	3.72494	3.29354	2.67741	2.69355	4.24690	2.90347	2.73739	3.18146	2.89801	2.37887	2.77519	2.98518	4.58477	3.61503	
	0.02696	4.02262	4.74496	0.61958	0.77255	0.48576	0.95510													
154	3.09348	4.41609	4.78567	4.29381	3.58255	4.42722	5.01586	1.40640	4.17989	2.09231	3.39199	4.51264	4.75084	4.45115	4.38221	3.81760	3.36313	1.08058	5.54385	4.33301	171 - -
	2.68618	4.42225	2.77519	2.73123	3.46354	2.40513	3.72494	3.29354	2.67741	2.69355	4.24690	2.90347	2.73739	3.18146	2.89801	2.37887	2.77519	2.98518	4.58477	3.61503	
	0.02696	4.02262	4.74496	0.61958	0.77255	0.48576	0.95510													
155	1.14448	4.16385	3.68284	3.32025	4.03703	3.08296	4.27056	3.04497	3.26685	3.01302	3.96465	3.44470	3.79637	3.58119	3.54652	2.48522	2.59350	2.44682	5.49798	4.28909	172 - -
	2.68618	4.42225	2.77519	2.73123	3.46354	2.40513	3.72494	3.29354	2.67741	2.69355	4.24690	2.90347	2.73739	3.18146	2.89801	2.37887	2.77519	2.98518	4.58477	3.61503	
	0.02696	4.02262	4.74496	0.61958	0.77255	0.48576	0.95510													
156	2.86181	4.62005	3.56615	3.48578	4.62096	0.53093	4.59479	4.25913	3.68734	3.89379	4.87234	3.73076	4.01589	3.99980	3.90487	3.03839	3.35475	3.75848	5.67431	4.72868	173 - -
	2.68618	4.42225	2.77519	2.73123	3.46354	2.40513	3.72494	3.29354	2.67741	2.69355	4.24690	2.90347	2.73739	3.18146	2.89801	2.37887	2.77519	2.98518	4.58477	3.61503	
	0.02696	4.02262	4.74496	0.61958	0.77255	0.48576	0.95510													
157	1.96574	4.23718	3.26384	3.06259	4.39116	1.22970	4.23059	3.79372	3.21660	3.49103	4.32962	3.27144	3.33965	3.49868	3.53762	2.45067	2.77267	3.24214	5.71482	4.50007	174 - -
	2.68618	4.42225	2.77519	2.73123	3.46354	2.40513	3.72494	3.29354	2.67741	2.69355	4.24690	2.90347	2.73739	3.18146	2.89801	2.37887	2.77519	2.98518	4.58477	3.61503	
	0.02696	4.02262	4.74496	0.61958	0.77255	0.48576	0.95510													
158	2.49976	4.34431	3.56805	3.33000	4.10800	3.17788	4.33500	3.25370	3.27593	3.11180	4.15459	3.51326	3.89436	3.66375	3.52580	2.70296	0.94063	2.93233	5.53249	4.32538	175 - -
	2.68618	4.42225	2.77519	2.73123	3.46354	2.40513	3.72494	3.29354	2.67741	2.69355	4.24690	2.90347	2.73739	3.18146	2.89801	2.37887	2.77519	2.98518	4.58477	3.61503	
	0.02696	4.02262	4.74496	0.61958	0.77255	0.48576	0.95510													
159	2.29660	4.25470	3.26092	3.06305	4.37522	2.53496	4.22530	3.85825	3.20034	3.53542	4.37056	3.27425	3.72648	3.49775	3.51644	1.03762	2.78809	3.28834	5.69711	4.45772	176 - -
	2.68618	4.42225	2.77519	2.73123	3.46354	2.40513	3.72494	3.29354	2.67741	2.69355	4.24690	2.90347	2.73739	3.18146	2.89801	2.37887	2.77519	2.98518	4.58477	3.61503	
	0.02696	4.02262	4.74496	0.61958	0.77255	0.48576	0.95510													
160	2.20241	4.20753	3.42640	3.09539	4.19452	3.01138	4.15578	3.52333	3.10202	3.26886	4.12309	3.29550	3.72813	3.42231	3.42068	1.72686	1.51419	3.06026	5.55604	4.33069	177 - -
	2.68618	4.42225	2.77519	2.73123	3.46354	2.40513	3.72494	3.29354	2.67741	2.69355	4.24690	2.90347	2.73739	3.18146	2.89801	2.37887	2.77519	2.98518	4.58477	3.61503	
	0.02696	4.02262	4.74496	0.61958	0.77255	0.48576	0.95510													
161	2.86181	4.62005	3.56615	3.48578	4.62096	0.53093	4.59479	4.25913	3.68734	3.89379	4.87234	3.73076	4.01589	3.99980	3.90487	3.03839	3.35475	3.75848	5.67431	4.72868	178 - -
	2.68618	4.42225	2.77519	2.73123	3.46354	2.40513	3.72494	3.29354	2.67741	2.69355	4.24690	2.90347	2.73739	3.18146	2.89801	2.37887	2.77519	2.98518	4.58477	3.61503	
	0.02696	4.02262	4.74496	0.61958	0.77255	0.48576	0.95510													
162	3.11448	5.07907	3.30011	2.89647	4.49993	3.58511	3.85036	3.93922	0.84983	3.47273	4.42835	3.31347	4.07671	3.03496	2.47086	3.14796	3.35771	3.63935	5.49460	4.33003	179 - -
	2.68618	4.42225	2.77519	2.73123	3.46354	2.40513	3.72494	3.29354	2.67741	2.69355	4.24690	2.90347	2.73739	3.18146	2.89801	2.37887	2.77519	2.98518	4.58477	3.61503	
	0.02696	4.02262	4.74496	0.61958	0.77255	0.48576	0.95510													
163	2.49976	4.34431	3.56805	3.33000	4.10800	3.17788	4.33500	3.25370	3.27593	3.11180	4.15459	3.51326	3.89436	3.66375	3.52580	2.70296	0.94063	2.93233	5.53249	4.32538	180 - -
	2.68618	4.42225	2.77519	2.73123	3.46354	2.40513	3.72494	3.29354	2.67741	2.69355	4.24690	2.90347	2.73739	3.18146	2.89801	2.37887	2.77519	2.98518	4.58477	3.61503	
	0.02696	4.02262	4.74496	0.61958	0.77255	0.48576	0.95510													
164	2.49976	4.34431	3.56805	3.33000	4.10800	3.17788	4.33500	3.25370	3.27593	3.11180	4.15459	3.51326	3.89436	3.66375	3.52580	2.70296	0.94063	2.93233	5.53249	4.32538	181 - -
	2.68618	4.42225	2.77519	2.73123	3.46354	2.40513	3.72494	3.29354	2.67741	2.69355	4.24690	2.90347	2.73739	3.18146	2.89801	2.37887	2.77519	2.98518	4.58477	3.61503	
	0.02696	4.02262	4.74496	0.61958	0.77255	0.48576	0.95510													
165	3.33472	4.70480	4.67585	4.19023	2.64262	4.44145	4.59352	2.27986	4.03098	0.82562	2.96078	4.42630	4.69334	4.19265	4.17392	3.83352	3.57422	2.40018	4.89527	3.55683	182 - -
	2.68618	4.42225	2.77519	2.73123	3.46354	2.40513	3.72494	3.29354	2.67741	2.69355	4.24690	2.90347	2.73739	3.18146	2.89801	2.37887	2.77519	2.98518	4.58477	3.61503	
	0.02696	4.02262	4.74496	0.61958	0.77255	0.48576	0.95510													
166	1.80169	4.20262	3.70877	3.25052	3.80703	3.26274	4.17245	2.72083	3.18800	2.75607	3.72601	3.46750	3.88718	3.49520	3.49256	2.65474	1.93794	1.98352	5.30145	4.08652	183 - -
	2.68618	4.42225	2.77519	2.73123	3.46354	2.40513	3.72494	3.29354	2.67741	2.69355	4.24690	2.90347	2.73739	3.18146	2.89801	2.37887	2.77519	2.98518	4.58477	3.61503	
	0.02696	4.02262	4.74496	0.61958	0.77255	0.48576	0.95510													
167	2.85426	4.85176	2.80169	2.70353	4.23951	3.28976	4.05172	3.98473	2.96940	3.61516	4.57129	0.91734	3.95002	3.32667	3.32750	2.91694	3.24227	3.59163	5.52835	4.17856	184 - -
	2.68618	4.42225	2.77519	2.73123	3.46354	2.40513	3.72494	3.29354	2.67741	2.69355	4.24690	2.90347	2.73739	3.18146	2.89801	2.37887	2.77519	2.98518	4.58477	3.61503	
	0.02696	4.02262	4.74496	0.61958	0.77255	0.48576	0.95510													
168	0.81728	4.27297	3.62041	3.42136	4.20706	3.09296	4.42492	3.35544	3.44162	3.22989	4.24718	3.53130	3.84663	3.76944	3.68019	2.62296	2.91086	2.98372	5.61916	4.43687	185 - -
	2.68618	4.42225	2.77519	2.73123	3.46354	2.40513	3.72494	3.29354	2.67741	2.69355	4.24690	2.90347	2.73739	3.18146	2.89801	2.37887	2.77519	2.98518	4.58477	3.61503	
	0.02696	4.02262	4.74496	0.61958	0.77255	0.48576	0.95510													
169	3.27212	4.70198	4.49805	4.07494	3.10839	4.27953	4.67075	2.25113	3.81850	0.79860	3.05236	4.33404	4.62752	4.13153	3.98072	3.73765	3.54596	2.35525	5.10451	3.84781	186 - -
	2.68618	4.42225	2.77519	2.73123	3.46354	2.40513	3.72494	3.29354	2.67741	2.69355	4.24690	2.90347	2.73739	3.18146	2.89801	2.37887	2.77519	2.98518	4.58477	3.61503	

	0.02696	4.02262	4.74496	0.61958	0.77255	0.48576	0.95510														
170	3.32709	4.74617	4.42998	4.07280	3.19879	4.17148	4.68160	2.41344	3.83907	0.71022	3.17896	4.33366	4.58250	4.17133	3.98965	3.76330	3.61534	2.48932	5.13017	3.88739	187 - -
	2.68618	4.42225	2.77519	2.73123	3.46354	2.40513	3.72494	3.29354	2.67741	2.69355	4.24690	2.90347	2.73739	3.18146	2.89801	2.37887	2.77519	2.98518	4.58477	3.61503	
	0.02696	4.02262	4.74496	0.61958	0.77255	0.48576	0.95510														
171	1.33026	4.49741	2.85604	2.66471	4.05013	3.03476	3.51121	3.47734	2.79721	3.14707	4.00602	3.08320	3.80580	3.12186	3.18982	2.61945	2.86436	3.11056	5.39524	4.09629	188 - -
	2.68618	4.42225	2.77519	2.73123	3.46354	2.40513	3.72494	3.29354	2.67741	2.69355	4.24690	2.90347	2.73739	3.18146	2.89801	2.37887	2.77519	2.98518	4.58477	3.61503	
	0.02696	4.02262	4.74496	0.61958	0.77255	0.48576	0.95510														
172	2.88423	5.09939	2.51921	1.10347	4.39405	3.32987	3.83693	3.76707	2.68488	3.41692	4.36703	2.92059	3.91307	3.02552	3.11199	2.87041	3.18214	3.30243	5.65927	4.28131	189 - -
	2.68618	4.42225	2.77519	2.73123	3.46354	2.40513	3.72494	3.29354	2.67741	2.69355	4.24690	2.90347	2.73739	3.18146	2.89801	2.37887	2.77519	2.98518	4.58477	3.61503	
	0.02696	4.02262	4.74496	0.61958	0.77255	0.48576	0.95510														
173	2.76777	4.26960	3.77066	3.64091	3.44551	4.00785	4.37559	1.59308	3.55353	2.14559	3.21016	3.93106	4.35262	3.82276	3.80566	3.31286	3.06842	1.44126	5.12282	3.91540	190 - -
	2.68618	4.42225	2.77519	2.73123	3.46354	2.40513	3.72494	3.29354	2.67741	2.69355	4.24690	2.90347	2.73739	3.18146	2.89801	2.37887	2.77519	2.98518	4.58477	3.61503	
	0.02696	4.02262	4.74496	0.61958	0.77255	0.48576	0.95510														
174	1.07037	4.14639	3.47667	3.21114	4.29290	2.57273	4.26565	3.64974	3.27179	3.38784	4.22455	3.33439	3.69956	3.55372	3.56621	2.25157	2.63205	3.12114	5.65249	4.45172	191 - -
	2.68618	4.42225	2.77519	2.73123	3.46354	2.40513	3.72494	3.29354	2.67741	2.69355	4.24690	2.90347	2.73739	3.18146	2.89801	2.37887	2.77519	2.98518	4.58477	3.61503	
	0.02696	4.02262	4.74496	0.61958	0.77255	0.48576	0.95510														
175	2.24353	4.86570	2.82161	2.36030	3.84453	3.31375	3.59929	3.55950	1.92914	3.13617	3.93924	2.89354	3.79591	2.68336	2.69220	2.61866	2.80143	3.21668	5.35097	4.00705	192 - -
	2.68618	4.42225	2.77519	2.73123	3.46354	2.40513	3.72494	3.29354	2.67741	2.69355	4.24690	2.90347	2.73739	3.18146	2.89801	2.37887	2.77519	2.98518	4.58477	3.61503	
	0.02696	4.02262	4.74496	0.61958	0.77255	0.48576	0.95510														
176	2.27603	4.04762	3.35987	2.86377	3.84577	3.02125	3.66681	3.22438	2.81795	2.85584	3.77203	3.20968	3.78904	3.15181	3.18535	2.30453	1.68449	2.88731	5.22278	3.97528	193 - -
	2.68618	4.42225	2.77519	2.73123	3.46354	2.40513	3.72494	3.29354	2.67741	2.69355	4.24690	2.90347	2.73739	3.18146	2.89801	2.37887	2.77519	2.98518	4.58477	3.61503	
	0.02696	4.02262	4.74496	0.61958	0.77255	0.48576	0.95510														
177	2.48194	4.88785	2.50491	2.26853	4.08812	2.42580	3.64314	3.64629	2.46443	3.22244	4.02596	2.87467	3.78822	2.72275	2.94688	2.29301	2.71048	3.28411	5.43902	4.07534	194 - -
	2.68618	4.42225	2.77519	2.73123	3.46354	2.40513	3.72494	3.29354	2.67741	2.69355	4.24690	2.90347	2.73739	3.18146	2.89801	2.37887	2.77519	2.98518	4.58477	3.61503	
	0.02696	4.02262	4.74496	0.61958	0.77255	0.48576	0.95510														
178	3.01138	5.55566	1.10566	1.85535	4.84546	3.23282	3.82099	4.36701	2.87843	3.88665	4.76707	2.70829	3.87685	2.99945	3.48561	2.88929	3.31173	3.94792	6.03190	4.52873	195 - -
	2.68618	4.42225	2.77519	2.73123	3.46354	2.40513	3.72494	3.29354	2.67741	2.69355	4.24690	2.90347	2.73739	3.18146	2.89801	2.37887	2.77519	2.98518	4.58477	3.61503	
	0.02696	4.02262	4.74496	0.61958	0.77255	0.48576	0.95510														
179	3.18120	5.00542	3.68780	3.17038	4.39834	3.63746	3.94268	3.95237	2.31116	3.44111	4.43934	3.51762	4.13451	3.15727	0.77162	3.25933	3.43807	3.67032	5.42160	4.29519	196 - -
	2.68618	4.42225	2.77519	2.73123	3.46354	2.40513	3.72494	3.29354	2.67741	2.69355	4.24690	2.90347	2.73739	3.18146	2.89801	2.37887	2.77519	2.98518	4.58477	3.61503	
	0.02696	4.02262	4.74496	0.61958	0.77255	0.48576	0.95510														
180	3.09019	4.42266	4.73426	4.22835	3.54627	4.41171	4.95066	1.58985	4.10462	2.00703	3.36604	4.46434	4.72741	4.38098	4.31438	3.78980	3.35501	1.02151	5.49591	4.28201	197 - -
	2.68618	4.42225	2.77519	2.73123	3.46354	2.40513	3.72494	3.29354	2.67741	2.69355	4.24690	2.90347	2.73739	3.18146	2.89801	2.37887	2.77519	2.98518	4.58477	3.61503	
	0.02696	4.02262	4.74496	0.61958	0.77255	0.48576	0.95510														
181	3.11348	4.43724	4.75366	4.22299	3.43368	4.44249	4.89745	1.66694	4.10486	1.64011	3.24680	4.46643	4.71781	4.33858	4.29670	3.80195	3.36289	1.17939	5.39404	4.21883	198 - -
	2.68618	4.42225	2.77519	2.73123	3.46354	2.40513	3.72494	3.29354	2.67741	2.69355	4.24690	2.90347	2.73739	3.18146	2.89801	2.37887	2.77519	2.98518	4.58477	3.61503	
	0.02696	4.02262	4.74496	0.61958	0.77255	0.48576	0.95510														
182	3.17148	4.48712	4.82190	4.27904	3.30774	4.49359	4.90156	1.57991	4.15852	1.28434	3.10664	4.52092	4.73273	4.34068	4.31994	3.84906	3.41120	1.57930	5.31817	4.19031	199 - -
	2.68618	4.42225	2.77519	2.73123	3.46354	2.40513	3.72494	3.29354	2.67741	2.69355	4.24690	2.90347	2.73739	3.18146	2.89801	2.37887	2.77519	2.98518	4.58477	3.61503	
	0.02696	4.02262	4.74496	0.61958	0.77255	0.48576	0.95510														
183	3.22147	4.54141	4.80627	4.29083	3.28022	4.51184	4.90766	1.22924	4.12279	1.39926	3.11120	4.53824	4.75969	4.34732	4.28501	3.89111	3.46660	1.87730	5.30940	4.13657	200 - -
	2.68618	4.42225	2.77519	2.73123	3.46354	2.40513	3.72494	3.29354	2.67741	2.69355	4.24690	2.90347	2.73739	3.18146	2.89801	2.37887	2.77519	2.98518	4.58477	3.61503	
	0.02696	4.02262	4.74496	0.61958	0.77255	0.48576	0.95510														
184	2.94977	5.27365	2.38943	1.08504	4.55504	3.31409	3.80363	4.04597	2.65401	3.59069	4.49990	2.84570	3.90580	2.85087	3.09329	2.89103	3.23463	3.69156	5.75148	4.35362	201 - -
	2.68618	4.42225	2.77519	2.73123	3.46354	2.40513	3.72494	3.29354	2.67741	2.69355	4.24690	2.90347	2.73739	3.18146	2.89801	2.37887	2.77519	2.98518	4.58477	3.61503	
	0.02696	4.02262	4.74496	0.61958	0.77255	0.48576	0.95510														
185	3.15706	5.30096	0.72443	2.44038	4.69960	3.32995	4.07187	4.32326	3.18574	3.90577	4.88941	3.00343	3.99358	3.31287	3.72060	3.10404	3.49962	3.94721	5.83844	4.55063	202 - -
	2.68618	4.42225	2.77519	2.73123	3.46354	2.40513	3.72494	3.29354	2.67741	2.69355	4.24690	2.90347	2.73739	3.18146	2.89801	2.37887	2.77519	2.98518	4.58477	3.61503	
	0.02696	4.02262	4.74496	0.61958	0.77255	0.48576	0.95510														
186	2.46810	4.28774	3.64662	3.25869	3.83532	3.27031	4.20850	2.79949	3.13315	2.65992	3.79883	3.47895	3.91836	3.51550	3.41506	2.69502	1.29071	2.41936	5.37651	4.12085	203 - -
	2.68618	4.42225	2.77519	2.73123	3.46354	2.40513	3.72494	3.29354	2.67741	2.69355	4.24690	2.90347	2.73739	3.18146	2.89801	2.37887	2.77519	2.98518	4.58477	3.61503	
	0.02696	4.02262	4.74496	0.61958	0.77255	0.48576	0.95510														
187	2.65080	4.53562	3.33486	2.75393	3.71186	3.53422	3.75099	2.72485	2.50951	2.59268	3.61879	3.19454	3.63194	2.97918	1.90383	2.79350	2.88601	2.50113	5.10074	3.84391	204 - -
	2.68618	4.42225	2.77519	2.73123	3.46354	2.40513	3.72494	3.29354	2.67741	2.69355	4.24690	2.90347	2.73739	3.18146	2.89801	2.37887	2.77519	2.98518	4.58477	3.61503	
	0.02696	4.02262	4.74496	0.61958	0.77255	0.48576	0.95510														
188	3.08846	5.22327	2.56059	0.82931	4.58940	3.37593	3.97883	4.07348	2.87880	3.66752	4.63740	3.02771	3.98839	3.19292	3.30140	3.05703	3.39464	3.74275	5.73268	4.45792	205 - -
	2.68618	4.42225	2.77519	2.73123	3.46354	2.40513	3.72494	3.29354	2.67741	2.69355	4.24690	2.90347	2.73739	3.18146	2.89801	2.37887	2.77519	2.98518	4.58477	3.61503	
	0.02696	4.02262	4.74496	0.61958	0.77255	0.48576	0.95510														
189	3.32709	4.74617	4.42998	4.07280	3.19879	4.17148	4.68160	2.41344	3.83907	0.71022	3.17896	4.33366	4.58250	4.17133	3.98965	3.76330	3.61534	2.48932	5.13017	3.88739	206 - -
	2.68618	4.42225	2.77519	2.73123	3.46354	2.40513	3.72494	3.29354	2.67741	2.69355	4.24690	2.90347	2.73739	3.18146	2.89801	2.37887	2.77519	2.98518	4.58477	3.61503	
	0.02696	4.02262	4.74496	0.61958	0.77255	0.48576	0.95510														
190	2.90640	5.07149	2.92382	2.56368	4.38380	3.48385	3.68733	3.84236	2.10996	3.35218	4.23505	3.04127	3.95203	1.40617	2.42175	2.90579	3.13750	3.51812	5.47576	4.18966	207 - -
	2.68618	4.42225	2.77519	2.73123	3.46354	2.40513	3.72494	3.29354	2.67741	2.69355	4.24690	2.90347	2.73739	3.18146	2.89801	2.37887	2.77519	2.98518	4.58477	3.61503	
	0.02696	4.02262	4.74496	0.61958	0.77255	0.48576	0.95510														
191	2.34185	1.11971	4.07533	3.78040	3.89092	3.09438	4.45797	3.08418	3.59041	3.02023	4.01893	3.67404	3.84704	3.91796	3.74675	2.44498	2.83296	2.74810	5.38541	4.14433	208 - -
	2.68618	4.42225	2.77519	2.73123	3.46354	2.40513	3.72494	3.29354	2.67741	2.69355	4.24690	2.90347	2.73739	3.18146	2.89801	2.37887	2.77519	2.98518	4.58477	3.61503	
	0.02696	4.02262	4.74496	0.61958	0.77255	0.48576	0.95510														
192	1.77936	4.55796	3.02522	2.58512	3.89197	3.35655	3.72107	3.27927	2.53765	2.88911	3.77832	3.05000	3.52390	2.87115	2.82956	2.59977	2.56306	2.93282	5.21875	3.93027	209 - -
	2.68618	4.42225	2.77519	2.73123	3.46354	2.40513	3.72494	3.29354	2.67741	2.69355	4.24690	2.90347	2.73739	3.18146	2.89801	2.37887	2.77519	2.98518	4.58477	3.61503	
	0.02696	4.02262	4.74496	0.61958	0.77255	0.48576	0.95510														
193	1.01885	4.20538	3.37500	3.14355	4.22660	2.99160	4.22856	3.54914	3.20134	3.34196	4.21278	3.14217	3.73771	3.52004	3.49296	2.35186	2.76414	3.07653	5.60671	4.36644	210 - -
	2.68618	4.42225	2.77519	2.73123	3.46354	2.40513	3.72494	3.29354	2.67741	2.69355	4.24690	2.90347	2.73739	3.18146	2.89801	2.37887	2.77519	2.98518	4.58477	3.61503	
	0.02696	4.02262	4.74496	0.61958	0.77255	0.48576	0.95510														
194	2.54307	4.76413	3.00064	2.44990	4.08069	3.39741	3.64954	3.48589	2.34277	3.08650	3.91218	2.98397	2.39784	2.78848	2.37473	2.47113	2.86184	3.05078	5.31783	4.00935	211 - -
	2.68618	4.42225	2.77519	2.73123	3.46354	2.40513	3.72494	3.29354	2.67741	2.69355	4.24690	2.90347	2.73739	3.18146	2.89801	2.37887	2.77519	2.98518	4.58477	3.61503	

	0.02696	4.02262	4.74496	0.61958	0.77255	0.48576	0.95510														
195	2.90488	5.37085	1.62114	2.16168	4.69446	3.23262	3.79205	4.25025	2.79472	3.77423	4.62671	1.58783	3.85355	2.96521	3.36478	2.81858	3.20711	3.82337	5.90248	4.41770	212 - -
	2.68618	4.42225	2.77519	2.73123	3.46354	2.40513	3.72494	3.29354	2.67741	2.69355	4.24690	2.90347	2.73739	3.18146	2.89801	2.37887	2.77519	2.98518	4.58477	3.61503	
	0.02696	4.02262	4.74496	0.61958	0.77255	0.48576	0.95510														
196	2.56503	3.75574	3.58294	3.01114	3.25818	3.57262	3.05553	2.65392	2.92400	1.89247	3.31204	3.37329	3.95292	3.22784	3.10340	2.83311	2.64726	2.36783	4.79717	3.56942	213 - -
	2.68618	4.42225	2.77519	2.73123	3.46354	2.40513	3.72494	3.29354	2.67741	2.69355	4.24690	2.90347	2.73739	3.18146	2.89801	2.37887	2.77519	2.98518	4.58477	3.61503	
	0.02696	4.02262	4.74496	0.61958	0.77255	0.48576	0.95510														
197	3.08660	4.42397	4.71842	4.21259	3.54101	4.39957	4.93629	1.62073	4.08602	1.99767	3.36422	4.45033	4.71870	4.36528	4.29732	3.77727	3.35213	1.01484	5.48752	4.27138	214 - -
	2.68618	4.42225	2.77519	2.73123	3.46354	2.40513	3.72494	3.29354	2.67741	2.69355	4.24690	2.90347	2.73739	3.18146	2.89801	2.37887	2.77519	2.98518	4.58477	3.61503	
	0.02696	4.02262	4.74496	0.61958	0.77255	0.48576	0.95510														
198	1.58225	4.37678	3.24358	2.78700	3.51872	3.26248	3.86533	3.20410	2.78129	2.89874	3.77336	3.17174	2.83628	3.02005	3.16247	2.54997	2.78838	2.88747	5.21603	3.94565	215 - -
	2.68618	4.42225	2.77519	2.73123	3.46354	2.40513	3.72494	3.29354	2.67741	2.69355	4.24690	2.90347	2.73739	3.18146	2.89801	2.37887	2.77519	2.98518	4.58477	3.61503	
	0.02696	4.02262	4.74496	0.61958	0.77255	0.48576	0.95510														
199	3.30228	4.67642	4.71043	4.16693	3.06443	4.47559	4.76357	2.21771	3.96557	0.89985	2.24821	4.45424	4.69143	4.16177	4.11777	3.83535	3.53106	2.34864	5.13601	4.02609	216 - -
	2.68618	4.42225	2.77519	2.73123	3.46354	2.40513	3.72494	3.29354	2.67741	2.69355	4.24690	2.90347	2.73739	3.18146	2.89801	2.37887	2.77519	2.98518	4.58477	3.61503	
	0.02696	4.02262	4.74496	0.61958	0.77255	0.48576	0.95510														
200	2.81525	4.00430	3.60822	3.01444	4.14371	3.49715	3.84816	3.54077	2.31751	3.15680	4.10292	3.35846	3.99635	3.05427	1.09884	2.92901	3.10726	3.24776	5.33523	4.12283	217 - -
	2.68618	4.42225	2.77519	2.73123	3.46354	2.40513	3.72494	3.29354	2.67741	2.69355	4.24690	2.90347	2.73739	3.18146	2.89801	2.37887	2.77519	2.98518	4.58477	3.61503	
	0.02696	4.02262	4.74496	0.61958	0.77255	0.48576	0.95510														
201	2.23321	4.24097	3.51628	3.24665	4.11727	3.09040	4.27092	3.19028	3.20683	3.09095	4.07774	3.41430	3.81487	3.56962	3.47753	2.56210	1.12788	2.84215	5.56418	4.34043	218 - -
	2.68618	4.42225	2.77519	2.73123	3.46354	2.40513	3.72494	3.29354	2.67741	2.69355	4.24690	2.90347	2.73739	3.18146	2.89801	2.37887	2.77519	2.98518	4.58477	3.61503	
	0.02696	4.02262	4.74496	0.61958	0.77255	0.48576	0.95510														
202	2.77447	5.08145	3.29265	2.65033	4.45951	3.59253	3.33919	3.83439	1.58982	3.20960	4.15635	3.10065	3.95752	2.69095	1.70229	2.89078	3.03552	3.50365	5.41981	4.19058	219 - -
	2.68618	4.42225	2.77519	2.73123	3.46354	2.40513	3.72494	3.29354	2.67741	2.69355	4.24690	2.90347	2.73739	3.18146	2.89801	2.37887	2.77519	2.98518	4.58477	3.61503	
	0.02696	4.02262	4.74496	0.61958	0.77255	0.48576	0.95510														
203	2.31987	5.13210	1.91899	2.10237	4.43112	3.31865	3.63279	3.89784	2.37912	3.42130	4.20959	2.79646	3.32170	2.68959	2.90087	2.65593	2.94495	3.50209	5.59579	4.18550	220 - -
	2.68618	4.42225	2.77519	2.73123	3.46354	2.40513	3.72494	3.29354	2.67741	2.69355	4.24690	2.90347	2.73739	3.18146	2.89801	2.37887	2.77519	2.98518	4.58477	3.61503	
	0.02696	4.02262	4.74496	0.61958	0.77255	0.48576	0.95510														
204	2.70093	4.66990	2.58860	2.77616	4.51323	0.92064	4.17171	4.04322	3.19730	3.68704	4.57914	3.19982	3.88755	3.43640	3.59320	2.80170	3.13307	3.57047	5.71158	4.50887	221 - -
	2.68618	4.42225	2.77519	2.73123	3.46354	2.40513	3.72494	3.29354	2.67741	2.69355	4.24690	2.90347	2.73739	3.18146	2.89801	2.37887	2.77519	2.98518	4.58477	3.61503	
	0.02696	4.02262	4.74496	0.61958	0.77255	0.48576	0.95510														
205	2.54167	4.26839	3.86348	3.33926	3.51445	3.72197	4.16223	2.16236	3.02275	2.37125	3.44167	3.65099	4.15040	3.54609	3.47754	2.96951	2.96091	1.34513	5.08802	3.86493	222 - -
	2.68618	4.42225	2.77519	2.73123	3.46354	2.40513	3.72494	3.29354	2.67741	2.69355	4.24690	2.90347	2.73739	3.18146	2.89801	2.37887	2.77519	2.98518	4.58477	3.61503	
	0.03705	4.02262	3.99187	0.61958	0.77255	0.48576	0.95510														
206	1.78024	4.26459	3.99795	3.52134	3.65093	3.60830	4.37039	2.13719	3.43151	2.43172	3.54334	3.75049	4.14835	3.73909	3.70675	2.98869	2.97971	1.48753	5.30126	4.08114	223 - -
	2.68618	4.42225	2.77519	2.73123	3.46354	2.40513	3.72494	3.29354	2.67741	2.69355	4.24690	2.90347	2.73739	3.18146	2.89801	2.37887	2.77519	2.98518	4.58477	3.61503	
	0.02723	4.01280	4.73514	0.61958	0.77255	0.47937	0.96541														
207	2.31365	4.28024	3.26925	2.95180	4.22136	3.03653	4.07691	3.62077	2.99097	3.31416	4.15142	3.05500	3.73332	3.32091	3.33518	1.39212	1.99173	3.14458	5.55564	4.30420	224 - -
	2.68618	4.42225	2.77519	2.73123	3.46354	2.40513	3.72494	3.29354	2.67741	2.69355	4.24690	2.90347	2.73739	3.18146	2.89801	2.37887	2.77519	2.98518	4.58477	3.61503	
	0.02696	4.02262	4.74496	0.61958	0.77255	0.48576	0.95510														
208	3.28217	4.65965	4.69372	4.14678	3.06488	4.45454	4.74266	2.22272	3.95124	0.98810	1.99286	4.43215	4.67406	4.14506	4.10443	3.80962	3.51033	2.35036	5.12527	4.01757	225 - -
	2.68618	4.42225	2.77519	2.73123	3.46354	2.40513	3.72494	3.29354	2.67741	2.69355	4.24690	2.90347	2.73739	3.18146	2.89801	2.37887	2.77519	2.98518	4.58477	3.61503	
	0.02696	4.02262	4.74496	0.61958	0.77255	0.48576	0.95510														
209	2.24312	4.56654	3.01168	2.58140	4.08806	3.25177	3.77405	3.49676	2.60603	3.12693	3.95507	2.88556	3.78365	2.65136	3.02471	1.89373	2.30799	3.12768	5.37828	4.07815	226 - -
	2.68618	4.42225	2.77519	2.73123	3.46354	2.40513	3.72494	3.29354	2.67741	2.69355	4.24690	2.90347	2.73739	3.18146	2.89801	2.37887	2.77519	2.98518	4.58477	3.61503	
	0.02696	4.02262	4.74496	0.61958	0.77255	0.48576	0.95510														
210	2.94549	5.54281	1.39623	1.64036	4.81802	3.23740	3.75870	4.34229	2.75829	3.82705	4.66776	2.65606	3.84973	2.74303	3.34939	2.82647	3.22791	3.90970	5.98426	4.47283	227 - -
	2.68618	4.42225	2.77519	2.73123	3.46354	2.40513	3.72494	3.29354	2.67741	2.69355	4.24690	2.90347	2.73739	3.18146	2.89801	2.37887	2.77519	2.98518	4.58477	3.61503	
	0.02696	4.02262	4.74496	0.61958	0.77255	0.48576	0.95510														
211	3.32709	4.74617	4.42998	4.07280	3.19879	4.17148	4.68160	2.41344	3.83907	0.71022	3.17896	4.33366	4.58250	4.17133	3.98965	3.76330	3.61534	2.48932	5.13017	3.88739	228 - -
	2.68618	4.42225	2.77519	2.73123	3.46354	2.40513	3.72494	3.29354	2.67741	2.69355	4.24690	2.90347	2.73739	3.18146	2.89801	2.37887	2.77519	2.98518	4.58477	3.61503	
	0.02696	4.02262	4.74496	0.61958	0.77255	0.48576	0.95510														
212	2.92784	4.43147	4.33116	4.00117	3.66379	3.87690	4.77821	2.05427	3.87125	2.32619	3.60830	4.16627	4.43068	4.22125	4.06895	3.39165	3.28677	0.85478	5.47286	4.21283	229 - -
	2.68618	4.42225	2.77519	2.73123	3.46354	2.40513	3.72494	3.29354	2.67741	2.69355	4.24690	2.90347	2.73739	3.18146	2.89801	2.37887	2.77519	2.98518	4.58477	3.61503	
	0.02696	4.02262	4.74496	0.61958	0.77255	0.48576	0.95510														
213	3.07753	5.08484	3.51154	2.90282	4.50949	3.65942	3.69172	3.90063	1.88010	3.38139	4.29045	3.27943	4.07056	2.84846	1.07980	3.10473	3.27109	3.60070	5.43168	4.26586	230 - -
	2.68618	4.42225	2.77519	2.73123	3.46354	2.40513	3.72494	3.29354	2.67741	2.69355	4.24690	2.90347	2.73739	3.18146	2.89801	2.37887	2.77519	2.98518	4.58477	3.61503	
	0.02696	4.02262	4.74496	0.61958	0.77255	0.48576	0.95510														
214	1.86457	4.19229	3.40251	3.15231	4.30801	2.95953	4.24251	3.67584	3.21684	3.41670	4.27050	3.31239	3.71389	3.51961	3.51977	1.14114	2.74148	3.15250	5.66669	4.43630	231 - -
	2.68618	4.42225	2.77519	2.73123	3.46354	2.40513	3.72494	3.29354	2.67741	2.69355	4.24690	2.90347	2.73739	3.18146	2.89801	2.37887	2.77519	2.98518	4.58477	3.61503	
	0.02696	4.02262	4.74496	0.61958	0.77255	0.48576	0.95510														
215	1.98750	4.19746	3.43721	3.07663	4.22190	2.88339	4.14075	3.60022	3.09572	3.30264	4.13178	3.27885	3.70837	3.39711	3.43124	1.50608	1.92179	3.10542	5.56597	4.34478	232 - -
	2.68618	4.42225	2.77519	2.73123	3.46354	2.40513	3.72494	3.29354	2.67741	2.69355	4.24690	2.90347	2.73739	3.18146	2.89801	2.37887	2.77519	2.98518	4.58477	3.61503	
	0.02696	4.02262	4.74496	0.61958	0.77255	0.48576	0.95510														
216	3.29838	4.67256	4.70927	4.16444	3.06387	4.47257	4.76025	2.21884	3.96534	0.91790	2.18554	4.45120	4.68822	4.15930	4.11722	3.83089	3.52672	2.34982	5.13361	4.02563	233 - -
	2.68618	4.42225	2.77519	2.73123	3.46354	2.40513	3.72494	3.29354	2.67741	2.69355	4.24690	2.90347	2.73739	3.18146	2.89801	2.37887	2.77519	2.98518	4.58477	3.61503	
	0.02696	4.02262	4.74496	0.61958	0.77255	0.48576	0.95510														
217	3.18120	5.00542	3.68780	3.17038	4.39834	3.63746	3.94268	3.95237	2.31116	3.44111	4.43934	3.51762	4.13451	3.15727	0.77162	3.25933	3.43807	3.67032	5.42160	4.29519	234 - -
	2.68618	4.42225	2.77519	2.73123	3.46354	2.40513	3.72494	3.29354	2.67741	2.69355	4.24690	2.90347	2.73739	3.18146	2.89801	2.37887	2.77519	2.98518	4.58477	3.61503	
	0.02696	4.02262	4.74496	0.61958	0.77255	0.48576	0.95510														
218	3.13684	4.63695	4.19709	3.75073	3.01359	4.12287	4.40098	2.40230	3.51639	0.90526	3.07119	4.05056	4.47478	3.62184	3.71824	3.51510	3.39411	2.47671	4.96341	3.66446	235 - -
	2.68618	4.42225	2.77519	2.73123	3.46354	2.40513	3.72494	3.29354	2.67741	2.69355	4.24690	2.90347	2.73739	3.18146	2.89801	2.37887	2.77519	2.98518	4.58477	3.61503	
	0.02696	4.02262	4.74496	0.61958	0.77255	0.48576	0.95510														
219	3.18120	5.00542	3.68780	3.17038	4.39834	3.63746	3.94268	3.95237	2.31116	3.44111	4.43934	3.51762	4.13451	3.15727	0.77162	3.25933	3.43807	3.67032	5.42160	4.29519	236 - -
	2.68618	4.42225	2.77519	2.73123	3.46354	2.40513	3.72494	3.29354	2.67741	2.69355	4.24690	2.90347	2.73739	3.18146	2.89801	2.37887	2.77519	2.98518	4.58477	3.61503	

	0.02696	4.02262	4.74496	0.61958	0.77255	0.48576	0.95510														
220	2.96212	4.69205	3.58591	3.44827	4.42684	3.37117	4.51157	4.01315	3.51887	3.62279	4.68053	3.73880	0.59042	3.90306	3.74552	3.13480	3.41594	3.64102	5.57002	4.55027	237 - -
	2.68618	4.42225	2.77519	2.73123	3.46354	2.40513	3.72494	3.29354	2.67741	2.69355	4.24690	2.90347	2.73739	3.18146	2.89801	2.37887	2.77519	2.98518	4.58477	3.61503	
	0.02696	4.02262	4.74496	0.61958	0.77255	0.48576	0.95510														
221	2.90927	5.17012	1.01308	2.30580	4.48242	3.25612	3.90184	3.96044	2.93996	3.61936	4.57050	2.85980	3.89913	3.10959	3.47715	2.87475	3.24416	3.40056	5.78335	4.34427	238 - -
	2.68618	4.42225	2.77519	2.73123	3.46354	2.40513	3.72494	3.29354	2.67741	2.69355	4.24690	2.90347	2.73739	3.18146	2.89801	2.37887	2.77519	2.98518	4.58477	3.61503	
	0.02696	4.02262	4.74496	0.61958	0.77255	0.48576	0.95510														
222	3.18120	5.00542	3.68780	3.17038	4.39834	3.63746	3.94268	3.95237	2.31116	3.44111	4.43934	3.51762	4.13451	3.15727	0.77162	3.25933	3.43807	3.67032	5.42160	4.29519	239 - -
	2.68618	4.42225	2.77519	2.73123	3.46354	2.40513	3.72494	3.29354	2.67741	2.69355	4.24690	2.90347	2.73739	3.18146	2.89801	2.37887	2.77519	2.98518	4.58477	3.61503	
	0.02696	4.02262	4.74496	0.61958	0.77255	0.48576	0.95510														
223	3.11435	4.44022	4.79051	4.30435	3.53699	4.43291	5.01716	1.08905	4.17939	2.02370	3.34807	4.52365	4.75500	4.44729	4.37454	3.82868	3.38503	1.43335	5.52166	4.31775	240 - -
	2.68618	4.42225	2.77519	2.73123	3.46354	2.40513	3.72494	3.29354	2.67741	2.69355	4.24690	2.90347	2.73739	3.18146	2.89801	2.37887	2.77519	2.98518	4.58477	3.61503	
	0.02696	4.02262	4.74496	0.61958	0.77255	0.48576	0.95510														
224	2.67322	4.31233	3.77727	3.25786	3.49483	3.66191	4.13428	1.97259	3.17004	2.32524	3.41612	3.58927	2.25431	3.48813	3.47532	2.98427	2.95695	1.90455	5.09791	3.87111	241 - -
	2.68618	4.42225	2.77519	2.73123	3.46354	2.40513	3.72494	3.29354	2.67741	2.69355	4.24690	2.90347	2.73739	3.18146	2.89801	2.37887	2.77519	2.98518	4.58477	3.61503	
	0.02696	4.02262	4.74496	0.61958	0.77255	0.48576	0.95510														
225	3.10538	4.41955	4.81540	4.32210	3.57684	4.45314	5.03708	1.27313	4.20958	2.07696	3.38171	4.53899	4.76720	4.47453	4.40721	3.84367	3.37280	1.18355	5.54803	4.34251	242 - -
	2.68618	4.42225	2.77519	2.73123	3.46354	2.40513	3.72494	3.29354	2.67741	2.69355	4.24690	2.90347	2.73739	3.18146	2.89801	2.37887	2.77519	2.98518	4.58477	3.61503	
	0.02696	4.02262	4.74496	0.61958	0.77255	0.48576	0.95510														
226	2.86181	4.62005	3.56615	3.48578	4.62096	0.53093	4.59479	4.25913	3.68734	3.89379	4.87234	3.73076	4.01589	3.99980	3.90487	3.03839	3.35475	3.75848	5.67431	4.72868	243 - -
	2.68618	4.42225	2.77519	2.73123	3.46354	2.40513	3.72494	3.29354	2.67741	2.69355	4.24690	2.90347	2.73739	3.18146	2.89801	2.37887	2.77519	2.98518	4.58477	3.61503	
	0.02696	4.02262	4.74496	0.61958	0.77255	0.48576	0.95510														
227	3.08846	5.22327	2.56059	0.82931	4.58940	3.37593	3.97883	4.07348	2.87880	3.66752	4.63740	3.02771	3.98839	3.19292	3.30140	3.05703	3.39464	3.74275	5.73268	4.45792	244 - -
	2.68618	4.42225	2.77519	2.73123	3.46354	2.40513	3.72494	3.29354	2.67741	2.69355	4.24690	2.90347	2.73739	3.18146	2.89801	2.37887	2.77519	2.98518	4.58477	3.61503	
	0.02696	4.02262	4.74496	0.61958	0.77255	0.48576	0.95510														
228	2.92784	4.43147	4.33116	4.00117	3.66379	3.87690	4.77821	2.05427	3.87125	2.32619	3.60830	4.16627	4.43068	4.22125	4.06895	3.39165	3.28677	0.85478	5.47286	4.21283	245 - -
	2.68618	4.42225	2.77519	2.73123	3.46354	2.40513	3.72494	3.29354	2.67741	2.69355	4.24690	2.90347	2.73739	3.18146	2.89801	2.37887	2.77519	2.98518	4.58477	3.61503	
	0.02696	4.02262	4.74496	0.61958	0.77255	0.48576	0.95510														
229	3.18120	5.00542	3.68780	3.17038	4.39834	3.63746	3.94268	3.95237	2.31116	3.44111	4.43934	3.51762	4.13451	3.15727	0.77162	3.25933	3.43807	3.67032	5.42160	4.29519	246 - -
	2.68618	4.42225	2.77519	2.73123	3.46354	2.40513	3.72494	3.29354	2.67741	2.69355	4.24690	2.90347	2.73739	3.18146	2.89801	2.37887	2.77519	2.98518	4.58477	3.61503	
	0.02696	4.02262	4.74496	0.61958	0.77255	0.48576	0.95510														
230	2.86181	4.62005	3.56615	3.48578	4.62096	0.53093	4.59479	4.25913	3.68734	3.89379	4.87234	3.73076	4.01589	3.99980	3.90487	3.03839	3.35475	3.75848	5.67431	4.72868	247 - -
	2.68618	4.42225	2.77519	2.73123	3.46354	2.40513	3.72494	3.29354	2.67741	2.69355	4.24690	2.90347	2.73739	3.18146	2.89801	2.37887	2.77519	2.98518	4.58477	3.61503	
	0.02696	4.02262	4.74496	0.61958	0.77255	0.48576	0.95510														
231	1.61188	4.38469	3.01311	2.74962	4.19754	2.40134	3.94054	3.61821	2.84977	3.26060	4.08058	3.11450	2.87438	3.14874	3.25966	2.26223	2.76552	3.16767	5.50712	4.23141	248 - -
	2.68618	4.42225	2.77519	2.73123	3.46354	2.40513	3.72494	3.29354	2.67741	2.69355	4.24690	2.90347	2.73739	3.18146	2.89801	2.37887	2.77519	2.98518	4.58477	3.61503	
	0.02696	4.02262	4.74496	0.61958	0.77255	0.48576	0.95510														
232	3.08846	5.22327	2.56059	0.82931	4.58940	3.37593	3.97883	4.07348	2.87880	3.66752	4.63740	3.02771	3.98839	3.19292	3.30140	3.05703	3.39464	3.74275	5.73268	4.45792	249 - -
	2.68618	4.42225	2.77519	2.73123	3.46354	2.40513	3.72494	3.29354	2.67741	2.69355	4.24690	2.90347	2.73739	3.18146	2.89801	2.37887	2.77519	2.98518	4.58477	3.61503	
	0.02696	4.02262	4.74496	0.61958	0.77255	0.48576	0.95510														
233	0.81728	4.27297	3.62041	3.42136	4.20706	3.09296	4.42492	3.35544	3.44162	3.22989	4.24718	3.53130	3.84663	3.76944	3.68019	2.62296	2.91086	2.98372	5.61916	4.43687	250 - -
	2.68618	4.42225	2.77519	2.73123	3.46354	2.40513	3.72494	3.29354	2.67741	2.69355	4.24690	2.90347	2.73739	3.18146	2.89801	2.37887	2.77519	2.98518	4.58477	3.61503	
	0.02696	4.02262	4.74496	0.61958	0.77255	0.48576	0.95510														
234	3.32709	4.74617	4.42998	4.07280	3.19879	4.17148	4.68160	2.41344	3.83907	0.71022	3.17896	4.33366	4.58250	4.17133	3.98965	3.76330	3.61534	2.48932	5.13017	3.88739	251 - -
	2.68618	4.42225	2.77519	2.73123	3.46354	2.40513	3.72494	3.29354	2.67741	2.69355	4.24690	2.90347	2.73739	3.18146	2.89801	2.37887	2.77519	2.98518	4.58477	3.61503	
	0.02696	4.02262	4.74496	0.61958	0.77255	0.48576	0.95510														
235	2.93419	5.38162	1.08643	2.10703	4.73536	3.21579	3.82738	4.24599	2.87591	3.80745	4.68602	2.72969	3.86347	3.01111	3.46240	2.67125	3.25179	3.82869	5.96703	4.47181	252 - -
	2.68618	4.42225	2.77519	2.73123	3.46354	2.40513	3.72494	3.29354	2.67741	2.69355	4.24690	2.90347	2.73739	3.18146	2.89801	2.37887	2.77519	2.98518	4.58477	3.61503	
	0.02696	4.02262	4.74496	0.61958	0.77255	0.48576	0.95510														
236	3.30910	4.69575	4.67257	4.14994	3.07556	4.45295	4.75210	2.21647	3.91691	0.82780	2.63893	4.43612	4.69281	4.15499	4.07615	3.83076	3.54706	2.33403	5.14069	3.99014	253 - -
	2.68618	4.42225	2.77519	2.73123	3.46354	2.40513	3.72494	3.29354	2.67741	2.69355	4.24690	2.90347	2.73739	3.18146	2.89801	2.37887	2.77519	2.98518	4.58477	3.61503	
	0.02696	4.02262	4.74496	0.61958	0.77255	0.48576	0.95510														
237	3.23861	4.57004	4.80978	4.26621	3.20344	4.51507	4.88144	1.72390	4.12245	1.03749	3.00432	4.52800	4.73763	4.29560	4.27813	3.87277	3.47498	1.89195	5.26020	4.14736	254 - -
	2.68618	4.42225	2.77519	2.73123	3.46354	2.40513	3.72494	3.29354	2.67741	2.69355	4.24690	2.90347	2.73739	3.18146	2.89801	2.37887	2.77519	2.98518	4.58477	3.61503	
	0.02696	4.02262	4.74496	0.61958	0.77255	0.48576	0.95510														
238	3.11448	5.07907	3.30011	2.89647	4.49993	3.58511	3.85036	3.93922	0.84983	3.47273	4.42835	3.31347	4.07671	3.03496	2.47086	3.14796	3.35771	3.63935	5.49460	4.33003	255 - -
	2.68618	4.42225	2.77519	2.73123	3.46354	2.40513	3.72494	3.29354	2.67741	2.69355	4.24690	2.90347	2.73739	3.18146	2.89801	2.37887	2.77519	2.98518	4.58477	3.61503	
	0.02696	4.02262	4.74496	0.61958	0.77255	0.48576	0.95510														
239	1.27292	4.21216	3.80005	3.41997	3.87285	3.24446	4.33277	2.67009	3.35319	2.74215	3.79996	3.56516	3.91955	3.66792	3.62422	2.67781	2.85438	1.96379	5.43373	4.21265	256 - -
	2.68618	4.42225	2.77519	2.73123	3.46354	2.40513	3.72494	3.29354	2.67741	2.69355	4.24690	2.90347	2.73739	3.18146	2.89801	2.37887	2.77519	2.98518	4.58477	3.61503	
	0.02696	4.02262	4.74496	0.61958	0.77255	0.48576	0.95510														
240	3.59642	4.91567	4.32809	4.07288	2.83521	3.90453	4.09650	3.62874	3.79302	2.99671	4.25522	4.19217	4.43527	4.17286	3.89421	3.79859	3.90492	3.52317	0.63014	2.82983	257 - -
	2.68618	4.42225	2.77519	2.73123	3.46354	2.40513	3.72494	3.29354	2.67741	2.69355	4.24690	2.90347	2.73739	3.18146	2.89801	2.37887	2.77519	2.98518	4.58477	3.61503	
	0.02696	4.02262	4.74496	0.61958	0.77255	0.48576	0.95510														
241	2.53851	4.57434	2.80341	2.67185	4.42164	1.29886	4.03707	3.95495	2.97545	3.58070	4.43329	2.26873	3.80554	3.26931	3.36661	2.63731	2.96755	3.45641	5.69216	4.38806	258 - -
	2.68618	4.42225	2.77519	2.73123	3.46354	2.40513	3.72494	3.29354	2.67741	2.69355	4.24690	2.90347	2.73739	3.18146	2.89801	2.37887	2.77519	2.98518	4.58477	3.61503	
	0.02696	4.02262	4.74496	0.61958	0.77255	0.48576	0.95510														
242	2.49976	4.34431	3.56805	3.33000	4.10800	3.17788	4.33500	3.25370	3.27593	3.11180	4.15459	3.51326	3.89436	3.66375	3.52580	2.70296	0.94063	2.93233	5.53249	4.32538	259 - -
	2.68618	4.42225	2.77519	2.73123	3.46354	2.40513	3.72494	3.29354	2.67741	2.69355	4.24690	2.90347	2.73739	3.18146	2.89801	2.37887	2.77519	2.98518	4.58477	3.61503	
	0.02696	4.02262	4.74496	0.61958	0.77255	0.48576	0.95510														
243	2.86181	4.62005	3.56615	3.48578	4.62096	0.53093	4.59479	4.25913	3.68734	3.89379	4.87234	3.73076	4.01589	3.99980	3.90487	3.03839	3.35475	3.75848	5.67431	4.72868	260 - -
	2.68618	4.42225	2.77519	2.73123	3.46354	2.40513	3.72494	3.29354	2.67741	2.69355	4.24690	2.90347	2.73739	3.18146	2.89801	2.37887	2.77519	2.98518	4.58477	3.61503	
	0.02696	4.02262	4.74496	0.61958	0.77255	0.48576	0.95510														
244	3.17169	4.95855	3.28396	3.06125	3.39905	3.58234	0.86156	3.89784	2.90062	3.37193	4.42840	3.46359	4.14428	3.43008	3.15617	3.24168	3.48110	3.63022	4.86163	3.34615	261 - -
	2.68618	4.42225	2.77519	2.73123	3.46354	2.40513	3.72494	3.29354	2.67741	2.69355	4.24690	2.90347	2.73739	3.18146	2.89801	2.37887	2.77519	2.98518	4.58477	3.61503	

	0.02696	4.02262	4.74496	0.61958	0.77255	0.48576	0.95510														
245	2.96212	4.69205	3.58591	3.44827	4.42684	3.37117	4.51157	4.01315	3.51887	3.62279	4.68053	3.73880	0.59042	3.90306	3.74552	3.13480	3.41594	3.64102	5.57002	4.55027	262 - -
	2.68618	4.42225	2.77519	2.73123	3.46354	2.40513	3.72494	3.29354	2.67741	2.69355	4.24690	2.90347	2.73739	3.18146	2.89801	2.37887	2.77519	2.98518	4.58477	3.61503	
	0.02696	4.02262	4.74496	0.61958	0.77255	0.48576	0.95510														
246	2.86181	4.62005	3.56615	3.48578	4.62096	0.53093	4.59479	4.25913	3.68734	3.89379	4.87234	3.73076	4.01589	3.99980	3.90487	3.03839	3.35475	3.75848	5.67431	4.72868	263 - -
	2.68618	4.42225	2.77519	2.73123	3.46354	2.40513	3.72494	3.29354	2.67741	2.69355	4.24690	2.90347	2.73739	3.18146	2.89801	2.37887	2.77519	2.98518	4.58477	3.61503	
	0.02696	4.02262	4.74496	0.61958	0.77255	0.48576	0.95510														
247	2.55426	4.46611	3.38098	3.28393	4.49890	0.71724	4.44424	4.03451	3.48427	3.71982	4.65753	3.51545	3.89781	3.79214	3.74191	2.77855	3.10585	3.52013	5.69336	4.60055	264 - -
	2.68618	4.42225	2.77519	2.73123	3.46354	2.40513	3.72494	3.29354	2.67741	2.69355	4.24690	2.90347	2.73739	3.18146	2.89801	2.37887	2.77519	2.98518	4.58477	3.61503	
	0.02696	4.02262	4.74496	0.61958	0.77255	0.48576	0.95510														
248	2.94991	3.81794	4.70253	4.16730	3.52028	4.30317	4.80608	1.21509	4.05805	2.04759	3.35614	4.36838	4.62520	4.29813	4.24443	3.65802	3.24824	1.44072	5.36357	4.16959	265 - -
	2.68618	4.42225	2.77519	2.73123	3.46354	2.40513	3.72494	3.29354	2.67741	2.69355	4.24690	2.90347	2.73739	3.18146	2.89801	2.37887	2.77519	2.98518	4.58477	3.61503	
	0.02696	4.02262	4.74496	0.61958	0.77255	0.48576	0.95510														
249	1.55533	4.16934	3.42406	3.13381	4.32015	1.65557	4.21955	3.71431	3.20949	3.41272	4.23385	3.29556	3.69241	3.48651	3.53116	2.23236	2.52963	3.16646	5.66041	4.45089	266 - -
	2.68618	4.42225	2.77519	2.73123	3.46354	2.40513	3.72494	3.29354	2.67741	2.69355	4.24690	2.90347	2.73739	3.18146	2.89801	2.37887	2.77519	2.98518	4.58477	3.61503	
	0.02696	4.02262	4.74496	0.61958	0.77255	0.48576	0.95510														
250	2.49976	4.34431	3.56805	3.33000	4.10800	3.17788	4.33500	3.25370	3.27593	3.11180	4.15459	3.51326	3.89436	3.66375	3.52580	2.70296	0.94063	2.93233	5.53249	4.32538	267 - -
	2.68618	4.42225	2.77519	2.73123	3.46354	2.40513	3.72494	3.29354	2.67741	2.69355	4.24690	2.90347	2.73739	3.18146	2.89801	2.37887	2.77519	2.98518	4.58477	3.61503	
	0.02696	4.02262	4.74496	0.61958	0.77255	0.48576	0.95510														
251	3.17827	4.48512	4.84624	4.31432	3.36300	4.52119	4.95686	1.24299	4.18757	1.51951	3.16393	4.55468	4.76618	4.39242	4.35667	3.88648	3.42283	1.65026	5.37964	4.23085	268 - -
	2.68618	4.42225	2.77519	2.73123	3.46354	2.40513	3.72494	3.29354	2.67741	2.69355	4.24690	2.90347	2.73739	3.18146	2.89801	2.37887	2.77519	2.98518	4.58477	3.61503	
	0.02696	4.02262	4.74496	0.61958	0.77255	0.48576	0.95510														
252	3.17169	4.95855	3.28396	3.06125	3.39905	3.58234	0.86156	3.89784	2.90062	3.37193	4.42840	3.46359	4.14428	3.43008	3.15617	3.24168	3.48110	3.63022	4.86163	3.34615	269 - -
	2.68618	4.42225	2.77519	2.73123	3.46354	2.40513	3.72494	3.29354	2.67741	2.69355	4.24690	2.90347	2.73739	3.18146	2.89801	2.37887	2.77519	2.98518	4.58477	3.61503	
	0.02696	4.02262	4.74496	0.61958	0.77255	0.48576	0.95510														
253	0.90846	4.20649	3.54679	3.33772	4.19249	3.02519	4.35858	3.36193	3.36057	3.24581	4.21363	3.44274	3.78628	3.68417	3.61296	2.48133	2.81643	2.95740	5.62499	4.39847	270 - -
	2.68618	4.42225	2.77519	2.73123	3.46354	2.40513	3.72494	3.29354	2.67741	2.69355	4.24690	2.90347	2.73739	3.18146	2.89801	2.37887	2.77519	2.98518	4.58477	3.61503	
	0.02696	4.02262	4.74496	0.61958	0.77255	0.48576	0.95510														
254	2.49276	4.55741	2.76055	2.61176	4.41589	1.46173	3.98507	3.91788	2.91355	3.53873	4.37562	2.22433	3.77668	3.20166	3.32100	2.51281	2.91525	3.41693	5.70041	4.37227	271 - -
	2.68618	4.42225	2.77519	2.73123	3.46354	2.40513	3.72494	3.29354	2.67741	2.69355	4.24690	2.90347	2.73739	3.18146	2.89801	2.37887	2.77519	2.98518	4.58477	3.61503	
	0.02696	4.02262	4.74496	0.61958	0.77255	0.48576	0.95510														
255	2.43131	4.43648	3.09706	2.72576	4.09315	3.00886	3.51998	3.52400	2.75707	3.17171	4.01011	3.11235	3.77355	3.10695	3.14098	1.52685	2.26598	3.12398	5.41396	4.13016	272 - -
	2.68618	4.42225	2.77519	2.73123	3.46354	2.40513	3.72494	3.29354	2.67741	2.69355	4.24690	2.90347	2.73739	3.18146	2.89801	2.37887	2.77519	2.98518	4.58477	3.61503	
	0.02696	4.02262	4.74496	0.61958	0.77255	0.48576	0.95510														
256	1.27835	4.25954	3.34267	2.90833	4.05297	2.72788	4.04720	3.38004	2.99184	3.11561	3.97508	3.24842	3.45571	3.30801	3.33847	2.50483	2.75369	2.77344	5.42362	4.18799	273 - -
	2.68618	4.42225	2.77519	2.73123	3.46354	2.40513	3.72494	3.29354	2.67741	2.69355	4.24690	2.90347	2.73739	3.18146	2.89801	2.37887	2.77519	2.98518	4.58477	3.61503	
	0.02696	4.02262	4.74496	0.61958	0.77255	0.48576	0.95510														
257	2.54050	4.21279	3.67115	3.09273	3.30998	3.62297	3.88063	2.23728	2.83261	1.81203	3.24626	3.43788	3.99431	3.23542	3.02030	2.89101	2.78704	2.41734	4.80605	3.48379	274 - -
	2.68618	4.42225	2.77519	2.73123	3.46354	2.40513	3.72494	3.29354	2.67741	2.69355	4.24690	2.90347	2.73739	3.18146	2.89801	2.37887	2.77519	2.98518	4.58477	3.61503	
	0.02696	4.02262	4.74496	0.61958	0.77255	0.48576	0.95510														
258	2.05323	4.20525	3.27371	3.08142	4.41507	1.26462	4.24790	3.84044	3.24678	3.53078	4.35425	3.26749	3.69967	3.51761	3.56462	2.08698	2.74521	3.25478	5.74525	4.52295	275 - -
	2.68618	4.42225	2.77519	2.73123	3.46354	2.40513	3.72494	3.29354	2.67741	2.69355	4.24690	2.90347	2.73739	3.18146	2.89801	2.37887	2.77519	2.98518	4.58477	3.61503	
	0.02696	4.02262	4.74496	0.61958	0.77255	0.48576	0.95510														
259	1.01480	4.15533	3.49931	3.24868	4.27617	2.65025	4.29284	3.54694	3.29492	3.34907	4.21698	3.36313	3.72021	3.58953	3.57558	2.43085	2.59878	3.05914	5.65267	4.45392	276 - -
	2.68618	4.42225	2.77519	2.73123	3.46354	2.40513	3.72494	3.29354	2.67741	2.69355	4.24690	2.90347	2.73739	3.18146	2.89801	2.37887	2.77519	2.98518	4.58477	3.61503	
	0.02696	4.02262	4.74496	0.61958	0.77255	0.48576	0.95510														
260	3.27233	4.70095	4.50429	4.07784	3.10671	4.28578	4.67301	2.24410	3.82122	0.80077	3.04820	4.33725	4.63041	4.13251	3.98357	3.74018	3.54518	2.35032	5.10524	3.84990	277 - -
	2.68618	4.42225	2.77519	2.73123	3.46354	2.40513	3.72494	3.29354	2.67741	2.69355	4.24690	2.90347	2.73739	3.18146	2.89801	2.37887	2.77519	2.98518	4.58477	3.61503	
	0.02696	4.02262	4.74496	0.61958	0.77255	0.48576	0.95510														
261	2.61931	4.54633	3.23926	2.65779	3.70503	3.50243	3.53697	2.99031	2.40398	2.36873	3.54747	3.02285	3.88169	2.90810	2.16001	2.73918	2.84371	2.84909	5.05691	3.37794	278 - -
	2.68618	4.42225	2.77519	2.73123	3.46354	2.40513	3.72494	3.29354	2.67741	2.69355	4.24690	2.90347	2.73739	3.18146	2.89801	2.37887	2.77519	2.98518	4.58477	3.61503	
	0.02696	4.02262	4.74496	0.61958	0.77255	0.48576	0.95510														
262	3.18120	5.00542	3.68780	3.17038	4.39834	3.63746	3.94268	3.95237	2.31116	3.44111	4.43934	3.51762	4.13451	3.15727	0.77162	3.25933	3.43807	3.67032	5.42160	4.29519	279 - -
	2.68618	4.42225	2.77519	2.73123	3.46354	2.40513	3.72494	3.29354	2.67741	2.69355	4.24690	2.90347	2.73739	3.18146	2.89801	2.37887	2.77519	2.98518	4.58477	3.61503	
	0.02696	4.02262	4.74496	0.61958	0.77255	0.48576	0.95510														
263	3.29187	4.63821	4.79138	4.22568	3.09141	4.52985	4.82525	2.04432	4.07634	0.91740	2.43919	4.51084	4.71744	4.21836	4.22245	3.87152	3.51388	2.20090	5.17483	4.10610	280 - -
	2.68618	4.42225	2.77519	2.73123	3.46354	2.40513	3.72494	3.29354	2.67741	2.69355	4.24690	2.90347	2.73739	3.18146	2.89801	2.37887	2.77519	2.98518	4.58477	3.61503	
	0.02696	4.02262	4.74496	0.61958	0.77255	0.48576	0.95510														
264	3.08846	5.22327	2.56059	0.82931	4.58940	3.37593	3.97883	4.07348	2.87880	3.66752	4.63740	3.02771	3.98839	3.19292	3.30140	3.05703	3.39464	3.74275	5.73268	4.45792	281 - -
	2.68618	4.42225	2.77519	2.73123	3.46354	2.40513	3.72494	3.29354	2.67741	2.69355	4.24690	2.90347	2.73739	3.18146	2.89801	2.37887	2.77519	2.98518	4.58477	3.61503	
	0.02696	4.02262	4.74496	0.61958	0.77255	0.48576	0.95510														
265	2.80481	4.90814	2.84095	2.58393	4.20435	3.36663	3.79318	3.78194	2.44355	3.33406	4.25921	3.05151	3.92100	1.30307	2.75219	2.70568	3.10767	3.44453	5.45529	4.10084	282 - -
	2.68618	4.42225	2.77519	2.73123	3.46354	2.40513	3.72494	3.29354	2.67741	2.69355	4.24690	2.90347	2.73739	3.18146	2.89801	2.37887	2.77519	2.98518	4.58477	3.61503	
	0.02696	4.02262	4.74496	0.61958	0.77255	0.48576	0.95510														
266	3.32709	4.74617	4.42998	4.07280	3.19879	4.17148	4.68160	2.41344	3.83907	0.71022	3.17896	4.33366	4.58250	4.17133	3.98965	3.76330	3.61534	2.48932	5.13017	3.88739	283 - -
	2.68618	4.42225	2.77519	2.73123	3.46354	2.40513	3.72494	3.29354	2.67741	2.69355	4.24690	2.90347	2.73739	3.18146	2.89801	2.37887	2.77519	2.98518	4.58477	3.61503	
	0.02696	4.02262	4.74496	0.61958	0.77255	0.48576	0.95510														
267	2.86005	4.34649	4.49739	3.99380	3.57624	4.15245	4.73709	1.16949	3.87585	2.17950	3.40747	4.22367	4.53929	4.16455	4.09964	3.52119	2.91290	1.56264	5.40821	4.20825	284 - -
	2.68618	4.42225	2.77519	2.73123	3.46354	2.40513	3.72494	3.29354	2.67741	2.69355	4.24690	2.90347	2.73739	3.18146	2.89801	2.37887	2.77519	2.98518	4.58477	3.61503	
	0.02696	4.02262	4.74496	0.61958	0.77255	0.48576	0.95510														
268	2.83146	4.78038	3.21550	2.76886	3.70463	3.57504	3.80010	3.16401	2.48315	2.12033	3.74571	3.21923	4.01048	1.66607	2.78586	2.92778	3.07637	3.00244	5.16790	3.83071	285 - -
	2.68618	4.42225	2.77519	2.73123	3.46354	2.40513	3.72494	3.29354	2.67741	2.69355	4.24690	2.90347	2.73739	3.18146	2.89801	2.37887	2.77519	2.98518	4.58477	3.61503	
	0.02696	4.02262	4.74496	0.61958	0.77255	0.48576	0.95510														
269	3.08846	5.22327	2.56059	0.82931	4.58940	3.37593	3.97883	4.07348	2.87880	3.66752	4.63740	3.02771	3.98839	3.19292	3.30140	3.05703	3.39464	3.74275	5.73268	4.45792	286 - -
	2.68618	4.42225	2.77519	2.73123	3.46354	2.40513	3.72494	3.29354	2.67741	2.69355	4.24690	2.90347	2.73739	3.18146	2.89801	2.37887	2.77519	2.98518	4.58477	3.61503	

	0.02696	4.02262	4.74496	0.61958	0.77255	0.48576	0.95510													
270	1.55085	4.23153	3.73746	3.24773	3.67114	3.40471	4.14290	2.39577	3.17264	2.60408	3.60147	3.50892	3.96628	3.48422	3.47738	2.65550	2.85275	1.97193	5.19741	3.97243	287 - -
	2.68618	4.42225	2.77519	2.73123	3.46354	2.40513	3.72494	3.29354	2.67741	2.69355	4.24690	2.90347	2.73739	3.18146	2.89801	2.37887	2.77519	2.98518	4.58477	3.61503	
	0.02696	4.02262	4.74496	0.61958	0.77255	0.48576	0.95510													
271	2.24469	4.23731	4.03625	3.52952	3.56784	3.70153	4.32220	2.00873	3.43578	2.41298	3.47829	3.77463	4.18410	3.73137	3.69946	2.89939	2.97675	1.33818	5.18732	3.97038	288 - -
	2.68618	4.42225	2.77519	2.73123	3.46354	2.40513	3.72494	3.29354	2.67741	2.69355	4.24690	2.90347	2.73739	3.18146	2.89801	2.37887	2.77519	2.98518	4.58477	3.61503	
	0.02696	4.02262	4.74496	0.61958	0.77255	0.48576	0.95510													
272	2.48231	4.22334	3.91582	3.34580	3.51006	3.68006	4.19385	2.08932	3.29983	2.41710	3.43663	3.67517	4.00527	3.60112	3.57257	2.92913	2.79284	1.37164	5.08215	3.86815	289 - -
	2.68618	4.42225	2.77519	2.73123	3.46354	2.40513	3.72494	3.29354	2.67741	2.69355	4.24690	2.90347	2.73739	3.18146	2.89801	2.37887	2.77519	2.98518	4.58477	3.61503	
	0.02696	4.02262	4.74496	0.61958	0.77255	0.48576	0.95510													
273	2.44282	4.63754	3.04415	2.52124	3.86840	3.41812	3.57171	3.26770	2.48504	2.90882	3.75181	2.61644	3.82813	2.80264	2.83043	2.64401	2.10288	2.90488	5.18654	3.64108	290 - -
	2.68618	4.42225	2.77519	2.73123	3.46354	2.40513	3.72494	3.29354	2.67741	2.69355	4.24690	2.90347	2.73739	3.18146	2.89801	2.37887	2.77519	2.98518	4.58477	3.61503	
	0.02696	4.02262	4.74496	0.61958	0.77255	0.48576	0.95510													
274	2.47768	4.26661	3.68368	3.26884	3.70147	3.39575	4.19008	2.57430	3.19918	2.60064	3.64279	3.52412	2.54415	3.53191	3.48838	2.79428	2.89587	1.42435	5.25106	4.01948	291 - -
	2.68618	4.42225	2.77519	2.73123	3.46354	2.40513	3.72494	3.29354	2.67741	2.69355	4.24690	2.90347	2.73739	3.18146	2.89801	2.37887	2.77519	2.98518	4.58477	3.61503	
	0.02696	4.02262	4.74496	0.61958	0.77255	0.48576	0.95510													
275	2.45764	4.39290	3.19605	3.01336	4.23553	3.09515	4.17653	3.72471	3.09626	3.41366	4.33007	3.29303	1.13183	3.46213	3.39743	2.43246	2.92003	3.26952	5.57580	4.30809	292 - -
	2.68618	4.42225	2.77519	2.73123	3.46354	2.40513	3.72494	3.29354	2.67741	2.69355	4.24690	2.90347	2.73739	3.18146	2.89801	2.37887	2.77519	2.98518	4.58477	3.61503	
	0.02696	4.02262	4.74496	0.61958	0.77255	0.48576	0.95510													
276	2.85553	4.88047	3.34907	2.75275	3.65398	3.58218	3.66674	3.53521	2.05026	3.09774	4.00225	3.17792	3.97926	2.84628	1.37143	2.91125	3.06835	3.25956	5.25512	3.97585	293 - -
	2.68618	4.42225	2.77519	2.73123	3.46354	2.40513	3.72494	3.29354	2.67741	2.69355	4.24690	2.90347	2.73739	3.18146	2.89801	2.37887	2.77519	2.98518	4.58477	3.61503	
	0.02696	4.02262	4.74496	0.61958	0.77255	0.48576	0.95510													
277	1.66647	4.66483	2.88952	2.35155	4.03639	3.25037	3.72273	3.43047	2.53981	3.06641	3.90576	2.99611	3.68259	2.90637	2.77073	2.63248	2.83076	3.07384	5.32864	4.02328	294 - -
	2.68618	4.42225	2.77519	2.73123	3.46354	2.40513	3.72494	3.29354	2.67741	2.69355	4.24690	2.90347	2.73739	3.18146	2.89801	2.37887	2.77519	2.98518	4.58477	3.61503	
	0.02696	4.02262	4.74496	0.61958	0.77255	0.48576	0.95510													
278	3.30023	4.69967	4.63105	4.12815	3.08165	4.41382	4.72742	2.22560	3.87478	0.81607	2.77878	4.40788	4.67955	4.14218	4.03672	3.80712	3.54677	2.33254	5.13155	3.94796	295 - -
	2.68618	4.42225	2.77519	2.73123	3.46354	2.40513	3.72494	3.29354	2.67741	2.69355	4.24690	2.90347	2.73739	3.18146	2.89801	2.37887	2.77519	2.98518	4.58477	3.61503	
	0.02696	4.02262	4.74496	0.61958	0.77255	0.48576	0.95510													
279	3.15037	4.51337	4.62848	4.19442	3.41414	4.33960	4.87364	0.98488	4.00374	1.90686	3.30757	4.42638	4.69774	4.32534	4.18959	3.77680	3.42957	1.80026	5.37820	4.11944	296 - -
	2.68618	4.42225	2.77519	2.73123	3.46354	2.40513	3.72494	3.29354	2.67741	2.69355	4.24690	2.90347	2.73739	3.18146	2.89801	2.37887	2.77519	2.98518	4.58477	3.61503	
	0.02696	4.02262	4.74496	0.61958	0.77255	0.48576	0.95510													
280	0.81728	4.27297	3.62041	3.42136	4.20706	3.09296	4.42492	3.35544	3.44162	3.22989	4.24718	3.53130	3.84663	3.76944	3.68019	2.62296	2.91086	2.98372	5.61916	4.43687	297 - -
	2.68618	4.42225	2.77519	2.73123	3.46354	2.40513	3.72494	3.29354	2.67741	2.69355	4.24690	2.90347	2.73739	3.18146	2.89801	2.37887	2.77519	2.98518	4.58477	3.61503	
	0.02696	4.02262	4.74496	0.61958	0.77255	0.48576	0.95510													
281	2.83784	5.14938	2.39402	1.21431	4.42273	3.31263	3.75755	3.63029	2.62337	3.45106	4.33168	2.83825	3.87010	2.92811	3.09199	2.80258	3.11399	3.48155	5.66276	4.25599	298 - -
	2.68618	4.42225	2.77519	2.73123	3.46354	2.40513	3.72494	3.29354	2.67741	2.69355	4.24690	2.90347	2.73739	3.18146	2.89801	2.37887	2.77519	2.98518	4.58477	3.61503	
	0.02696	4.02262	4.74496	0.61958	0.77255	0.48576	0.95510													
282	1.62693	4.21717	3.57342	3.16405	3.97107	3.14071	4.15001	2.94255	3.10794	2.95559	3.89332	3.37761	3.80913	3.43769	3.41964	2.55373	1.67968	2.72263	5.41260	4.18991	299 - -
	2.68618	4.42225	2.77519	2.73123	3.46354	2.40513	3.72494	3.29354	2.67741	2.69355	4.24690	2.90347	2.73739	3.18146	2.89801	2.37887	2.77519	2.98518	4.58477	3.61503	
	0.02696	4.02262	4.74496	0.61958	0.77255	0.48576	0.95510													
283	3.13093	4.43260	4.84360	4.33161	3.52670	4.50308	5.02657	1.19742	4.21665	1.94639	3.32808	4.55952	4.78391	4.46559	4.40848	3.88120	3.38775	1.32151	5.51011	4.31907	300 - -
	2.68618	4.42225	2.77519	2.73123	3.46354	2.40513	3.72494	3.29354	2.67741	2.69355	4.24690	2.90347	2.73739	3.18146	2.89801	2.37887	2.77519	2.98518	4.58477	3.61503	
	0.02696	4.02262	4.74496	0.61958	0.77255	0.48576	0.95510													
284	2.88028	5.39059	1.41116	2.14734	4.68403	3.25209	3.74090	4.20651	2.69216	3.71082	4.54215	2.11239	3.84100	2.78889	3.13368	2.78970	3.15992	3.78715	5.86501	4.38792	301 - -
	2.68618	4.42225	2.77519	2.73123	3.46354	2.40513	3.72494	3.29354	2.67741	2.69355	4.24690	2.90347	2.73739	3.18146	2.89801	2.37887	2.77519	2.98518	4.58477	3.61503	
	0.02696	4.02262	4.74496	0.61958	0.77255	0.48576	0.95510													
285	3.13256	4.47219	4.72508	4.18719	3.33129	4.42193	4.83840	1.80419	4.05871	1.34170	3.14647	4.43779	4.68899	4.27371	4.24055	3.77613	3.37796	1.37843	5.31020	4.15668	302 - -
	2.68618	4.42225	2.77519	2.73123	3.46354	2.40513	3.72494	3.29354	2.67741	2.69355	4.24690	2.90347	2.73739	3.18146	2.89801	2.37887	2.77519	2.98518	4.58477	3.61503	
	0.02696	4.02262	4.74496	0.61958	0.77255	0.48576	0.95510													
286	2.81816	4.27820	4.28451	3.73875	3.35802	4.01329	4.11630	1.61129	3.59908	2.07979	3.28247	3.98848	4.36700	3.87697	3.81340	3.33624	3.09383	1.39571	5.06634	3.84742	303 - -
	2.68618	4.42225	2.77519	2.73123	3.46354	2.40513	3.72494	3.29354	2.67741	2.69355	4.24690	2.90347	2.73739	3.18146	2.89801	2.37887	2.77519	2.98518	4.58477	3.61503	
	0.02696	4.02262	4.74496	0.61958	0.77255	0.48576	0.95510													
287	1.82648	4.27526	3.82842	3.20687	3.52934	3.69299	4.16482	1.96497	3.22501	2.39611	3.44247	3.62613	4.13158	3.53317	3.52654	3.01154	2.94965	1.82726	5.11234	3.89159	304 - -
	2.68618	4.42225	2.77519	2.73123	3.46354	2.40513	3.72494	3.29354	2.67741	2.69355	4.24690	2.90347	2.73739	3.18146	2.89801	2.37887	2.77519	2.98518	4.58477	3.61503	
	0.02696	4.02262	4.74496	0.61958	0.77255	0.48576	0.95510													
288	2.65544	4.24907	3.71462	3.16401	2.56599	3.66044	2.91692	2.68732	3.07371	2.39578	3.34894	3.47686	4.03715	3.35278	3.35440	2.87491	2.88973	1.82789	4.53190	3.00296	305 - -
	2.68618	4.42225	2.77519	2.73123	3.46354	2.40513	3.72494	3.29354	2.67741	2.69355	4.24690	2.90347	2.73739	3.18146	2.89801	2.37887	2.77519	2.98518	4.58477	3.61503	
	0.02696	4.02262	4.74496	0.61958	0.77255	0.48576	0.95510													
289	3.25456	4.57328	4.85608	4.29749	3.17290	4.54777	4.88716	1.60029	4.16106	1.08495	2.78370	4.55735	4.74477	4.30397	4.30145	3.89691	3.48224	2.00841	5.23783	4.15329	306 - -
	2.68618	4.42225	2.77519	2.73123	3.46354	2.40513	3.72494	3.29354	2.67741	2.69355	4.24690	2.90347	2.73739	3.18146	2.89801	2.37887	2.77519	2.98518	4.58477	3.61503	
	0.02696	4.02262	4.74496	0.61958	0.77255	0.48576	0.95510													
290	2.14769	4.67000	2.91946	2.39197	3.97858	3.36235	3.67275	3.38221	2.49730	2.95987	3.83944	2.94878	3.68296	2.84569	2.84676	2.05316	2.79571	2.94149	5.27308	3.95879	307 - -
	2.68618	4.42225	2.77519	2.73123	3.46354	2.40513	3.72494	3.29354	2.67741	2.69355	4.24690	2.90347	2.73739	3.18146	2.89801	2.37887	2.77519	2.98518	4.58477	3.61503	
	0.03776	4.02262	3.95540	0.61958	0.77255	0.48576	0.95510													
291	2.63203	4.59443	3.15542	2.86286	4.30710	1.21941	3.99595	3.79451	2.73096	3.40745	4.28526	3.23770	3.87450	3.22485	2.38775	2.74700	3.01623	3.37971	5.51251	4.29738	308 - -
	2.68618	4.42225	2.77519	2.73123	3.46354	2.40513	3.72494	3.29354	2.67741	2.69355	4.24690	2.90347	2.73739	3.18146	2.89801	2.37887	2.77519	2.98518	4.58477	3.61503	
	0.02725	4.01210	4.73445	0.61958	0.77255	0.49680	0.93771													
292	2.69943	4.18694	2.98841	2.58974	4.13495	3.46252	3.65123	3.52175	2.17646	3.11765	3.96114	3.05684	3.87869	2.81131	1.58709	2.74798	2.93479	3.20573	5.31867	4.04200	309 - -
	2.68618	4.42225	2.77519	2.73123	3.46354	2.40513	3.72494	3.29354	2.67741	2.69355	4.24690	2.90347	2.73739	3.18146	2.89801	2.37887	2.77519	2.98518	4.58477	3.61503	
	0.02725	4.01210	4.73445	0.61958	0.77255	0.49680	0.93771													
293	2.69186	4.66193	2.57981	2.76446	4.50056	0.93766	4.15944	4.02880	3.18335	3.67327	4.56516	3.18890	3.87860	3.42338	3.57978	2.79223	3.12291	3.55790	5.70063	4.49596	310 - -
	2.68618	4.42225	2.77519	2.73123	3.46354	2.40513	3.72494	3.29354	2.67741	2.69355	4.24690	2.90347	2.73739	3.18146	2.89801	2.37887	2.77519	2.98518	4.58477	3.61503	
	0.02725	4.01210	4.73445	0.61958	0.77255	0.49680	0.93771													
294	2.29314	4.76574	2.96375	2.44463	4.07066	3.16059	3.53132	3.48597	2.36647	3.08332	3.89937	2.87111	3.80034	2.74664	2.46049	2.22740	2.65330	3.15158	5.31642	3.99193	311 - -
	2.68618	4.42225	2.77519	2.73123	3.46354	2.40513	3.72494	3.29354	2.67741	2.69355	4.24690	2.90347	2.73739	3.18146	2.89801	2.37887	2.77519	2.98518	4.58477	3.61503	

	0.02725	4.01210	4.73445	0.61958	0.77255	0.49680	0.93771													
295	2.22740	4.87928	2.77385	2.26322	4.15195	3.03473	3.59853	3.40558	2.15002	3.15690	3.95556	2.86781	3.78404	2.65557	2.84587	2.47834	2.78540	3.23323	5.37104	4.01572	312 - -
	2.68618	4.42225	2.77519	2.73123	3.46354	2.40513	3.72494	3.29354	2.67741	2.69355	4.24690	2.90347	2.73739	3.18146	2.89801	2.37887	2.77519	2.98518	4.58477	3.61503	
	0.02725	4.01210	4.73445	0.61958	0.77255	0.49680	0.93771													
296	3.16824	4.99396	3.67365	3.15796	4.38416	3.62702	3.93346	3.93711	2.30475	3.42776	4.42593	3.50618	4.12428	3.14828	0.78476	3.24667	3.42563	3.65544	5.41150	4.28292	313 - -
	2.68618	4.42225	2.77519	2.73123	3.46354	2.40513	3.72494	3.29354	2.67741	2.69355	4.24690	2.90347	2.73739	3.18146	2.89801	2.37887	2.77519	2.98518	4.58477	3.61503	
	0.02725	4.01210	4.73445	0.61958	0.77255	0.49680	0.93771													
297	3.00805	5.09672	3.39132	2.77144	4.41115	3.64619	3.09454	3.86162	1.88483	3.33677	4.20847	3.18333	4.02352	2.77191	1.27164	3.01684	3.18490	3.55346	5.38025	4.16041	314 - -
	2.68618	4.42225	2.77519	2.73123	3.46354	2.40513	3.72494	3.29354	2.67741	2.69355	4.24690	2.90347	2.73739	3.18146	2.89801	2.37887	2.77519	2.98518	4.58477	3.61503	
	0.02725	4.01210	4.73445	0.61958	0.77255	0.49680	0.93771													
298	3.15832	4.48794	4.76676	4.22344	3.28951	4.45781	4.85770	1.72097	4.09929	1.24176	3.09374	4.47424	4.70464	4.29050	4.26912	3.81040	3.39875	1.54891	5.29320	4.16131	315 - -
	2.68618	4.42225	2.77519	2.73123	3.46354	2.40513	3.72494	3.29354	2.67741	2.69355	4.24690	2.90347	2.73739	3.18146	2.89801	2.37887	2.77519	2.98518	4.58477	3.61503	
	0.02725	4.01210	4.73445	0.61958	0.77255	0.49680	0.93771													
299	2.33062	4.82706	2.83201	2.08214	4.10647	3.30986	3.61530	3.52969	2.33881	3.11891	3.92599	2.90642	3.78815	2.76010	2.82132	2.34881	2.59223	3.03449	5.34670	4.00106	316 - -
	2.68618	4.42225	2.77519	2.73123	3.46354	2.40513	3.72494	3.29354	2.67741	2.69355	4.24690	2.90347	2.73739	3.18146	2.89801	2.37887	2.77519	2.98518	4.58477	3.61503	
	0.02725	4.01210	4.73445	0.61958	0.77255	0.49680	0.93771													
300	2.56744	4.56526	2.72811	1.69333	4.22056	3.32138	3.66108	3.64268	2.46360	3.23086	4.04086	2.89452	3.79311	2.80925	2.88533	2.23894	2.82153	3.27884	5.44690	4.09285	317 - -
	2.68618	4.42225	2.77519	2.73123	3.46354	2.40513	3.72494	3.29354	2.67741	2.69355	4.24690	2.90347	2.73739	3.18146	2.89801	2.37887	2.77519	2.98518	4.58477	3.61503	
	0.02725	4.01210	4.73445	0.61958	0.77255	0.49680	0.93771													
301	2.89597	4.45747	4.61216	4.06817	3.28025	4.32611	4.72271	1.63110	3.93444	1.22908	3.09722	4.32728	4.60676	4.15307	4.12047	3.66964	3.33046	1.86463	5.23068	4.09350	318 - -
	2.68618	4.42225	2.77519	2.73123	3.46354	2.40513	3.72494	3.29354	2.67741	2.69355	4.24690	2.90347	2.73739	3.18146	2.89801	2.37887	2.77519	2.98518	4.58477	3.61503	
	0.02725	4.01210	4.73445	0.61958	0.77255	0.49680	0.93771													
302	1.60692	4.08977	3.70231	3.17295	3.58586	3.44647	4.05784	2.55608	3.08915	2.43982	3.37355	3.46903	3.96252	3.40100	3.40274	2.69933	2.83874	2.07662	5.09917	3.87438	319 - -
	2.68618	4.42225	2.77519	2.73123	3.46354	2.40513	3.72494	3.29354	2.67741	2.69355	4.24690	2.90347	2.73739	3.18146	2.89801	2.37887	2.77519	2.98518	4.58477	3.61503	
	0.02725	4.01210	4.73445	0.61958	0.77255	0.49680	0.93771													
303	2.60930	4.56697	3.07003	2.36628	4.13053	3.45840	3.46971	3.53174	2.24819	3.07680	3.93445	2.99661	3.84940	2.68455	1.83925	2.67933	2.71349	3.21020	5.31568	4.01888	320 - -
	2.68618	4.42225	2.77519	2.73123	3.46354	2.40513	3.72494	3.29354	2.67741	2.69355	4.24690	2.90347	2.73739	3.18146	2.89801	2.37887	2.77519	2.98518	4.58477	3.61503	
	0.02725	4.01210	4.73445	0.61958	0.77255	0.49680	0.93771													
304	2.92117	4.36113	4.39868	3.90935	3.54452	3.94865	4.65673	1.83139	3.75891	2.13396	3.43251	4.14541	4.49280	4.08385	3.99003	3.45669	3.21006	1.03916	5.35728	4.10653	321 - -
	2.68618	4.42225	2.77519	2.73123	3.46354	2.40513	3.72494	3.29354	2.67741	2.69355	4.24690	2.90347	2.73739	3.18146	2.89801	2.37887	2.77519	2.98518	4.58477	3.61503	
	0.02725	4.01210	4.73445	0.61958	0.77255	0.49680	0.93771													
305	2.54831	4.82700	2.62904	2.07360	4.07934	3.38657	3.59920	3.46686	2.37738	3.09193	3.89761	2.88691	3.78590	2.63797	2.85623	2.60259	2.56162	2.75231	4.98250	3.97559	322 - -
	2.68618	4.42225	2.77519	2.73123	3.46354	2.40513	3.72494	3.29354	2.67741	2.69355	4.24690	2.90347	2.73739	3.18146	2.89801	2.37887	2.77519	2.98518	4.58477	3.61503	
	0.02725	4.01210	4.73445	0.61958	0.77255	0.49680	0.93771													
306	2.41045	4.63170	2.63838	2.40759	4.36308	1.37238	3.93873	3.76417	2.87192	3.42649	4.28533	2.99401	3.80327	3.15312	3.29290	2.64647	2.95609	3.33893	5.65329	4.33789	323 - -
	2.68618	4.42225	2.77519	2.73123	3.46354	2.40513	3.72494	3.29354	2.67741	2.69355	4.24690	2.90347	2.73739	3.18146	2.89801	2.37887	2.77519	2.98518	4.58477	3.61503	
	0.02725	4.01210	4.73445	0.61958	0.77255	0.49680	0.93771													
307	2.96299	4.17518	4.45175	3.88834	2.35301	4.11509	4.36229	2.19696	3.76295	1.21304	3.02174	4.10258	4.41764	3.93780	3.92514	3.37472	3.19740	2.14039	4.83985	3.58029	324 - -
	2.68618	4.42225	2.77519	2.73123	3.46354	2.40513	3.72494	3.29354	2.67741	2.69355	4.24690	2.90347	2.73739	3.18146	2.89801	2.37887	2.77519	2.98518	4.58477	3.61503	
	0.02725	4.01210	4.73445	0.61958	0.77255	0.49680	0.93771													
308	2.68693	5.09143	1.75460	2.20072	4.42333	2.50886	3.68435	3.88361	2.56211	3.22009	4.24305	2.76308	3.80512	2.79205	3.07710	2.55030	2.95399	3.49566	5.62813	4.21985	325 - -
	2.68618	4.42225	2.77519	2.73123	3.46354	2.40513	3.72494	3.29354	2.67741	2.69355	4.24690	2.90347	2.73739	3.18146	2.89801	2.37887	2.77519	2.98518	4.58477	3.61503	
	0.02725	4.01210	4.73445	0.61958	0.77255	0.49680	0.93771													
309	2.34099	4.66264	2.88918	2.40576	4.10811	2.50663	3.63715	3.52533	2.56424	3.14054	3.96564	2.97161	2.41066	2.90296	2.96147	2.50915	2.76803	3.16605	5.38635	4.06800	326 - -
	2.68618	4.42225	2.77519	2.73123	3.46354	2.40513	3.72494	3.29354	2.67741	2.69355	4.24690	2.90347	2.73739	3.18146	2.89801	2.37887	2.77519	2.98518	4.58477	3.61503	
	0.04961	4.01210	3.49661	0.61958	0.77255	0.49680	0.93771													
310	2.36102	4.80351	2.42059	2.33492	4.07002	3.18131	3.61967	3.48994	2.43313	3.05376	3.90218	2.84846	3.78638	2.70429	2.87693	2.47325	2.78270	2.69914	5.32755	3.98421	327 - -
	2.68619	4.42226	2.77520	2.73124	3.46355	2.40514	3.72495	3.29353	2.67742	2.69356	4.24691	2.90346	2.73740	3.18147	2.89802	2.37883	2.77514	2.98519	4.58478	3.61504	
	0.25865	2.49162	1.93011	0.42444	1.06171	0.48283	0.95980													
311	2.31146	4.31749	2.97015	2.73447	4.15138	1.43652	4.00422	3.50972	2.91124	3.14882	4.14732	3.10990	3.71169	3.26086	3.24337	2.50073	2.75351	3.08591	5.50170	4.22383	329 - -
	2.68618	4.42225	2.77519	2.73123	3.46354	2.40513	3.72494	3.29354	2.67741	2.69355	4.24690	2.90347	2.73739	3.18146	2.89801	2.37887	2.77519	2.98518	4.58477	3.61503	
	0.03151	3.86913	4.59148	0.61958	0.77255	0.42151	1.06728													
312	2.57364	5.04672	1.82235	2.22788	4.38170	2.62524	3.65741	3.75755	2.49520	3.38350	4.18388	2.81563	3.79590	2.79775	2.79695	2.62500	2.90808	3.44870	5.57444	4.17848	330 - -
	2.68618	4.42225	2.77519	2.73123	3.46354	2.40513	3.72494	3.29354	2.67741	2.69355	4.24690	2.90347	2.73739	3.18146	2.89801	2.37887	2.77519	2.98518	4.58477	3.61503	
	0.02725	4.01210	4.73445	0.61958	0.77255	0.49680	0.93771													
313	3.45411	4.88634	4.04680	3.80408	2.31065	3.99469	3.69188	3.44442	3.65767	2.86185	4.10530	3.89907	4.46609	3.92990	3.81200	3.57621	3.74192	3.32181	3.95916	0.76181	331 - -
	2.68618	4.42225	2.77519	2.73123	3.46354	2.40513	3.72494	3.29354	2.67741	2.69355	4.24690	2.90347	2.73739	3.18146	2.89801	2.37887	2.77519	2.98518	4.58477	3.61503	
	0.02725	4.01210	4.73445	0.61958	0.77255	0.49680	0.93771													
314	2.54539	4.41780	3.14667	2.56794	3.86557	3.46891	3.41388	3.19452	2.38983	2.70087	3.74017	3.04890	3.85316	2.63289	2.04991	2.69947	2.82728	2.82621	5.16411	3.87982	332 - -
	2.68618	4.42225	2.77519	2.73123	3.46354	2.40513	3.72494	3.29354	2.67741	2.69355	4.24690	2.90347	2.73739	3.18146	2.89801	2.37887	2.77519	2.98518	4.58477	3.61503	
	0.02725	4.01210	4.73445	0.61958	0.77255	0.49680	0.93771													
315	3.02679	4.40081	4.55957	4.00361	3.30585	4.27166	4.65450	1.70482	3.88521	1.32916	3.08723	4.26031	4.55788	4.10238	4.07758	3.60177	3.00859	1.71373	5.19973	4.05739	333 - -
	2.68583	4.42234	2.77496	2.73110	3.46362	2.40520	3.72503	3.29363	2.67748	2.69341	4.24698	2.90355	2.73748	3.18155	2.89809	2.37895	2.77526	2.98522	4.58457	3.61512	
	0.15943	2.14495	3.49661	1.33167	0.30657	0.49680	0.93771													
316	2.34970	4.55274	3.04497	2.55735	3.76448	3.42643	3.61977	2.95265	2.48601	2.79009	3.61785	3.01513	3.82602	2.85232	2.85729	2.38213	2.31373	2.85228	5.11189	3.82431	343 - -
	2.68618	4.42225	2.77519	2.73123	3.46354	2.40513	3.72494	3.29354	2.67741	2.69355	4.24690	2.90347	2.73739	3.18146	2.89801	2.37887	2.77519	2.98518	4.58477	3.61503	
	0.02786	3.99036	4.71270	0.61958	0.77255	0.51889	0.90431													
317	2.59744	4.88607	2.57081	2.25887	4.17774	3.32805	3.56625	3.60709	2.50743	3.12268	4.03001	2.79121	2.06211	2.80660	2.96702	2.60890	2.92273	3.26807	5.43205	4.07986	344 - -
	2.68618	4.42225	2.77519	2.73123	3.46354	2.40513	3.72494	3.29354	2.67741	2.69355	4.24690	2.90347	2.73739	3.18146	2.89801	2.37887	2.77519	2.98518	4.58477	3.61503	
	0.02786	3.99036	4.71270	0.61958	0.77255	0.51889	0.90431													
318	2.19635	4.14415	3.86122	3.25702	2.60098	3.68168	3.90697	2.33677	3.19440	1.95753	3.14728	3.57891	4.04962	3.45046	3.44378	2.96032	2.78593	2.18692	4.75675	3.54872	345 - -
	2.68575	4.42247	2.77523	2.73127	3.46365	2.40537	3.72522	3.29359	2.67765	2.69319	4.24720	2.90374	2.73663	3.18162	2.89828	2.37863	2.77531	2.98531	4.58507	3.61534	
	0.54962	1.44386	1.67762	1.94058	0.15504	0.51889	0.90431													
319	2.49891	4.56965	2.93284	2.37822	3.73849	3.37226	3.56998	3.17554	2.45826	2.69369	3.67018	2.89911	3.27082	2.78804	2.89062	2.53887	2.59081	2.79252	5.11408	3.75239	362 - -
	2.68618	4.42225	2.77519	2.73123	3.46354	2.40513	3.72494	3.29354	2.67741	2.69355	4.24690	2.90347	2.73739	3.18146	2.89801	2.37887	2.77519	2.98518	4.58477	3.61503	

```
         0.03399  3.79454  4.51689  0.61958  0.77255  0.57247  0.83041
    320  2.40819  4.61990  2.84649  2.45591  3.87961  3.35610  3.52647  3.15413  2.41679  2.86886  3.74937  2.95363  3.29667  2.80457  2.90010  2.27471  2.61650  2.94948  5.18841  3.87864    363 - -
         2.68618  4.42225  2.77519  2.73123  3.46354  2.40513  3.72494  3.29354  2.67741  2.69355  4.24690  2.90347  2.73739  3.18146  2.89801  2.37887  2.77519  2.98518  4.58477  3.61503
         0.03131  3.87522  4.59757  0.61958  0.77255  0.53928  0.87508
    321  2.42806  4.39996  2.91668  2.49132  3.69182  3.36262  3.55313  3.16017  2.48815  2.72978  3.68891  2.99458  2.73278  2.76948  2.92551  2.54743  2.70226  2.91032  5.13572  3.83625    364 - -
         2.68618  4.42225  2.77519  2.73123  3.46354  2.40513  3.72494  3.29354  2.67741  2.69355  4.24690  2.90347  2.73739  3.18146  2.89801  2.37887  2.77519  2.98518  4.58477  3.61503
         0.02958  3.93123  4.65357  0.61958  0.77255  0.56525  0.83984
    322  2.50204  4.83113  2.56246  2.27529  4.02429  3.29920  3.40230  3.52077  2.37028  2.95244  3.90340  2.82007  3.45261  2.70045  2.84241  2.40022  2.67851  3.05859  5.32542  3.97327    365 - -
         2.68618  4.42225  2.77519  2.73123  3.46354  2.40513  3.72494  3.29354  2.67741  2.69355  4.24690  2.90347  2.73739  3.18146  2.89801  2.37887  2.77519  2.98518  4.58477  3.61503
         0.02941  3.93704  4.65939  0.61958  0.77255  0.56218  0.84389
    323  2.42200  4.84464  2.81784  2.31044  4.10941  3.36860  3.42892  3.48607  2.33381  3.04304  3.91262  2.80058  3.33540  2.54296  2.69050  2.38688  2.72518  3.16805  5.33077  3.97922    366 - -
         2.68618  4.42225  2.77519  2.73123  3.46354  2.40513  3.72494  3.29354  2.67741  2.69355  4.24690  2.90347  2.73739  3.18146  2.89801  2.37887  2.77519  2.98518  4.58477  3.61503
         0.02927  3.94149  4.66383  0.61958  0.77255  0.56521  0.83989
    324  2.50573  4.04704  3.00849  2.34855  3.71393  3.40920  3.51212  3.22997  2.44776  2.70845  3.70753  2.90724  3.33775  2.60472  2.90409  2.53873  2.63017  2.90971  5.15230  3.84656    367 - -
         2.68618  4.42225  2.77519  2.73123  3.46354  2.40513  3.72494  3.29354  2.67741  2.69355  4.24690  2.90347  2.73739  3.18146  2.89801  2.37887  2.77519  2.98518  4.58477  3.61503
         0.02927  3.94149  4.66383  0.61958  0.77255  0.46967  0.98138
    325  2.46268  4.57999  2.93946  2.54047  3.97693  1.76648  3.61895  3.17509  2.61743  3.03736  3.89491  3.03865  3.82355  2.91472  3.00783  2.61614  2.86895  3.06054  5.30317  4.00675    368 - -
         2.68618  4.42225  2.77519  2.73123  3.46354  2.40513  3.72494  3.29354  2.67741  2.69355  4.24690  2.90347  2.73739  3.18146  2.89801  2.37887  2.77519  2.98518  4.58477  3.61503
         0.02751  4.00262  4.72496  0.61958  0.77255  0.50656  0.92273
    326  2.58692  5.07644  2.21762  1.83216  4.37458  3.32583  3.60725  3.83623  2.39971  3.23326  4.14673  2.70641  3.51166  2.68814  2.91184  2.62331  2.85239  3.29134  5.54030  4.13888    369 - -
         2.68618  4.42225  2.77519  2.73123  3.46354  2.40513  3.72494  3.29354  2.67741  2.69355  4.24690  2.90347  2.73739  3.18146  2.89801  2.37887  2.77519  2.98518  4.58477  3.61503
         0.05111  4.00262  3.45592  0.61958  0.77255  0.50656  0.92273
    327  2.37933  3.93635  3.22921  2.65494  3.61623  3.44592  3.67431  2.99074  2.63068  2.33053  3.54546  3.06672  3.05226  2.89784  2.95098  2.51391  2.68766  2.73464  5.00789  3.74562    370 - -
         2.68618  4.42225  2.77519  2.73123  3.46354  2.40513  3.72494  3.29354  2.67741  2.69355  4.24690  2.90347  2.73739  3.18146  2.89801  2.37887  2.77519  2.98518  4.58477  3.61503
         0.04469  3.97967  3.68820  0.61958  0.77255  0.52940  0.88906
    328  2.46429  4.73309  2.97443  2.35760  3.97315  3.40215  3.42612  3.34299  2.31984  2.81497  3.81867  2.95028  2.76264  2.77513  2.73127  2.46323  2.80104  3.06899  5.24386  3.92645    371 - -
         2.68622  4.42229  2.77518  2.73122  3.46358  2.40513  3.72499  3.29358  2.67740  2.69348  4.24694  2.90344  2.73738  3.18151  2.89798  2.37879  2.77524  2.98523  4.58481  3.61507
         0.04644  3.09277        *  1.34909  0.30040  0.00000        *
//
```
