# Supplementary material for: Unveiling a missing component of the atypical type IV secretion system required for natural transformation of Helicobacter pylori
Source: PLoS Pathog. 2026 Jul 14;22(7):e1014140. doi: 10.1371/journal.ppat.1014140 (PMC13395361; doi:10.1371/journal.ppat.1014140)
Supplement: S1 Raw Image — (PDF) [file ppat.1014140.s017.pdf]

Fig 2A

1X Agarose Gel stained with 1X SYBR Safe DNA Gel Stain - Invitrogen

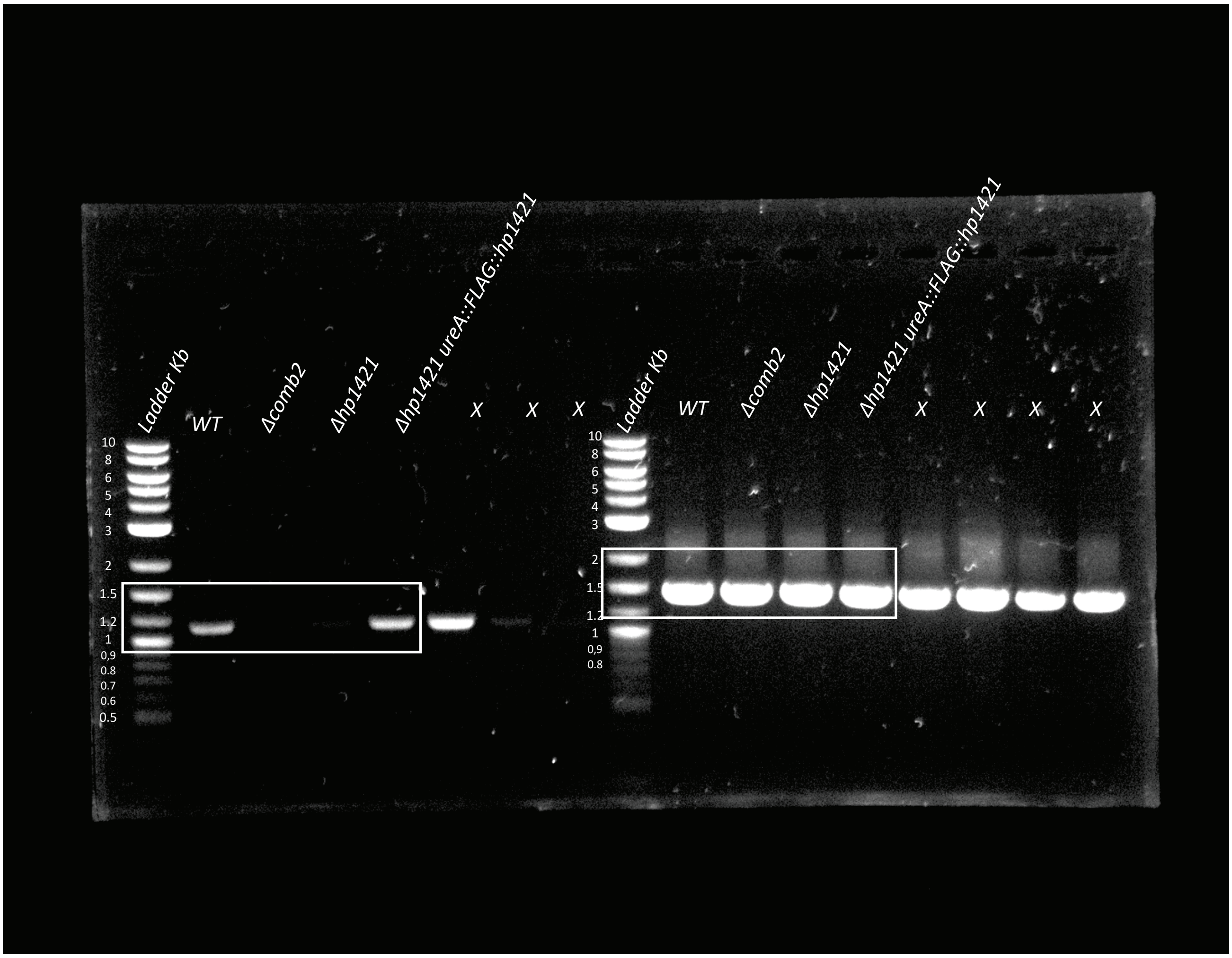

Ladder: 1 kb Plus DNA Ladder for Safe Stains- NEB

Method used to capture the image: EBOX VX5/20M, Vilber Lourmat.

Figure panel generated from the original image is marked in white.

Fig 3D

Nitrocelulose membrane incubated with the following antibodies for the detection of:

1st Mouse Anti-FLAG - 2nd Goat Anti-Mouse IR800
FLAG-HP1421 36 kDa

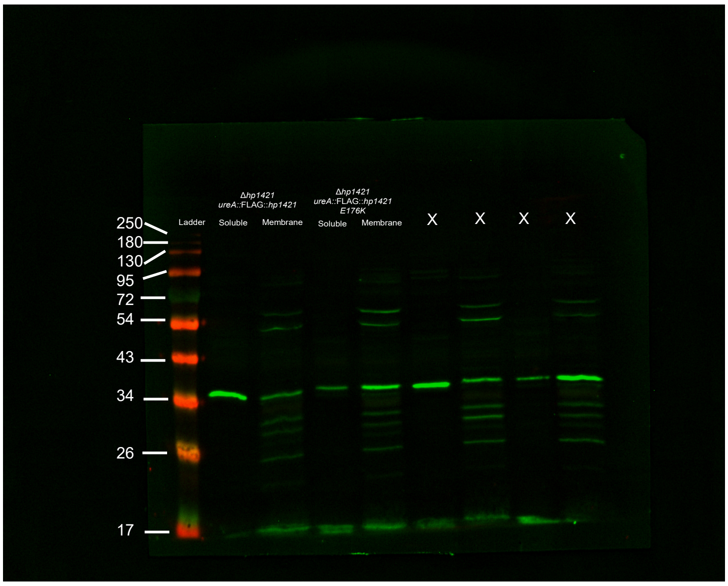

1st Rabbit Anti-MotB - 2nd Goat Anti-Rabbit IR700
MotB 28 kDa

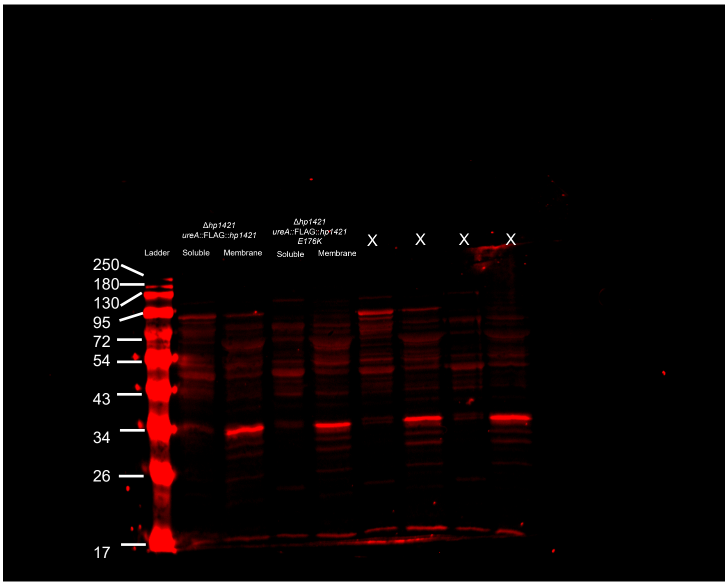

1st Rabbit Anti-NikR - 2nd Goat Anti-Rabbit IR700
NikR 17 kDa

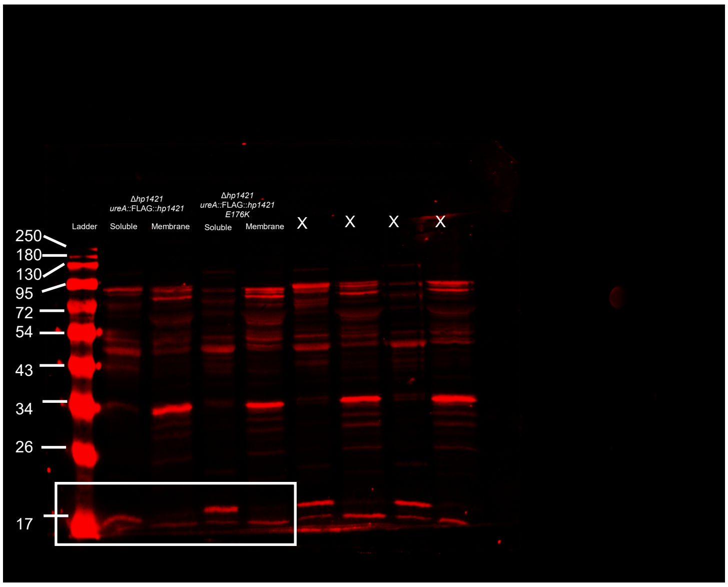

3rd revealing all antibodies merged by
iBright FL1500 Imaging System

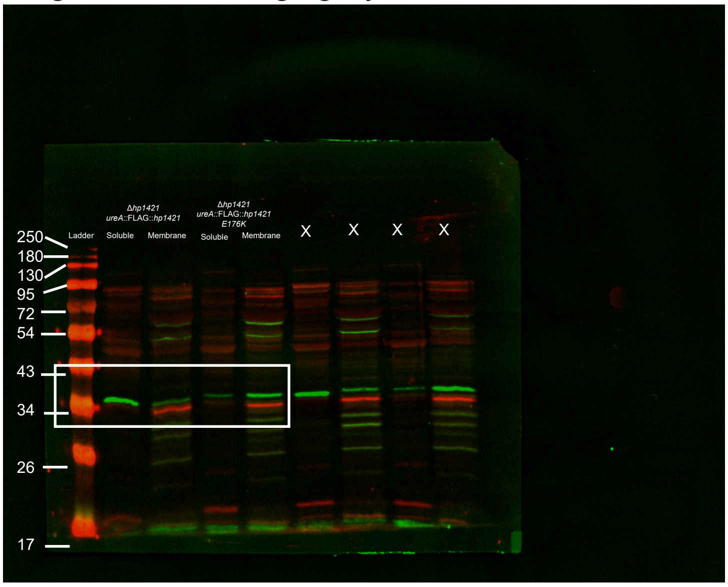

Ladder: Color Prestained Protein Standard, Broad Range (10-250 kDa) - NEB

Method used to capture the image: iBright FL1500 Imaging System - Invitrogen

Figure panel generated from the original image is marked in white.

Fig 6C

Nitrocelulose membrane incubated with the following antibodies for the detection of:

Lama FluoTag-X2 anti-ALFA-ATTO488
ComB4-ALFATag 92 kDa

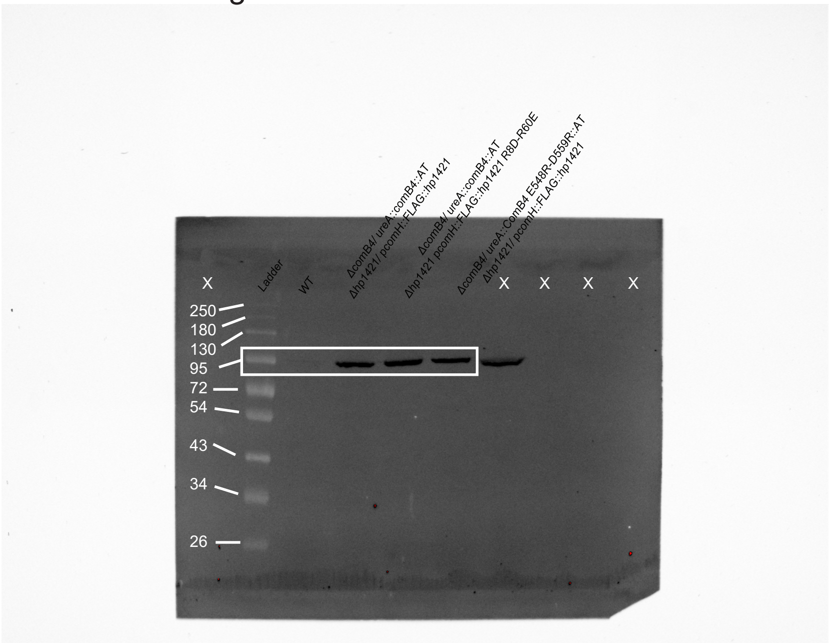

1st Mouse Anti-FLAG - 2nd Goat Anti-Mouse IR800
FLAG-HP1421 36 kDa

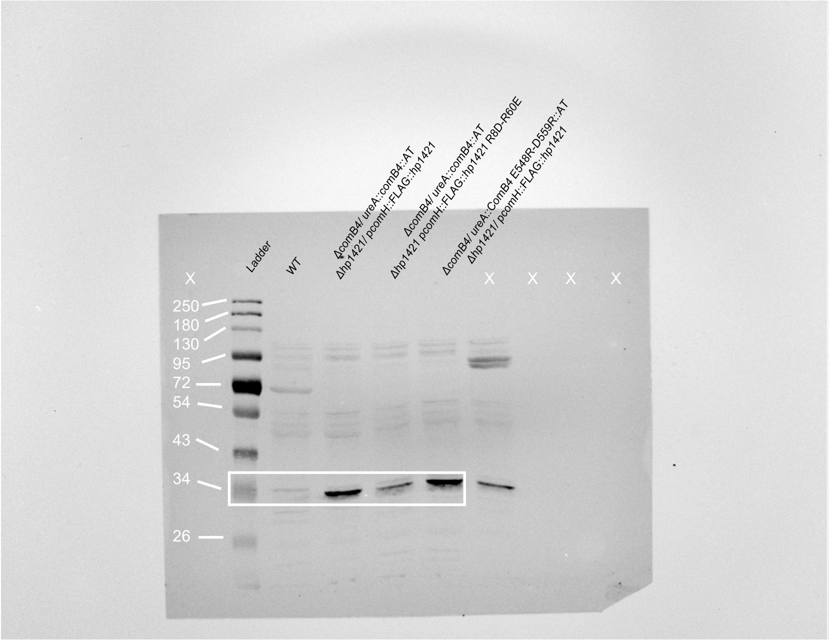

Ladder: Color Prestained Protein Standard, Broad Range (10-250 kDa) - NEB

Method used to capture the image: iBright FL1500 Imaging System - Invitrogen

Figure panel generated from the original image is marked in white.

Fig 6D

Nitrocelulose membrane incubated with the following antibodies for the detection of:

1st Mouse Anti-FLAG - 2nd Goat Anti-Mouse IR800
FLAG-HP1421 36 kDa

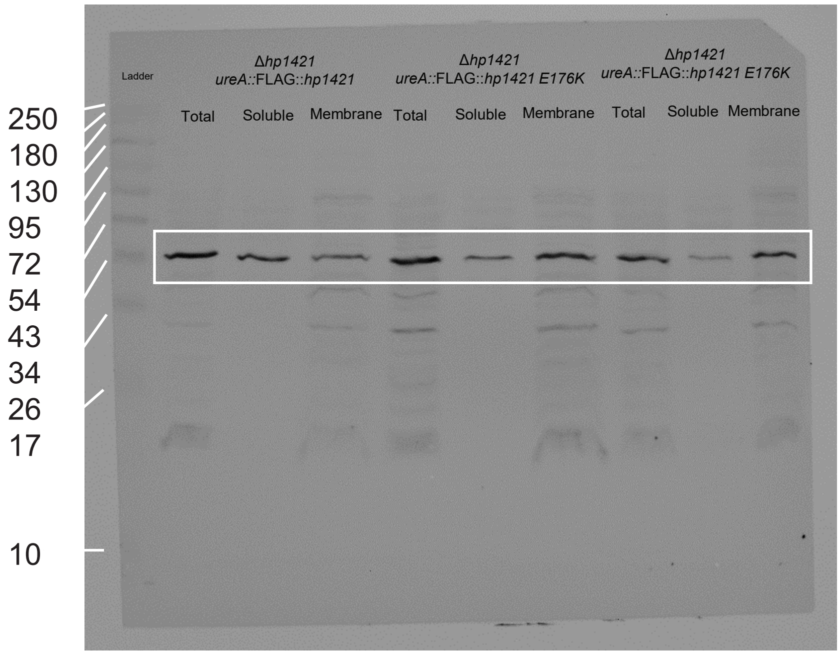

1st Rabbit Anti-NikR - 2nd Goat Anti-Rabbit IR700
NikR 17 kDa

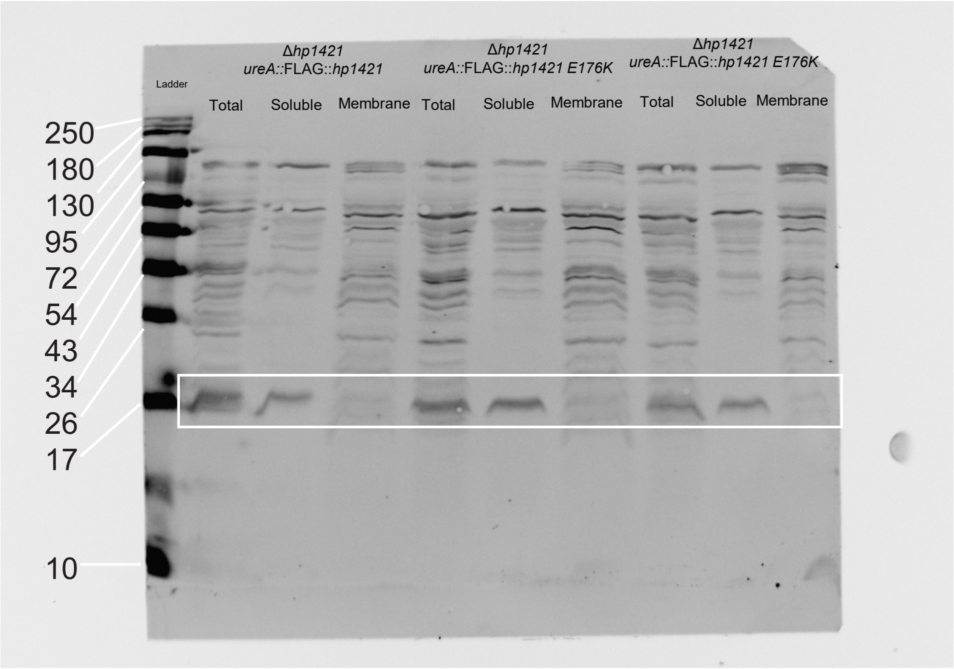

Ladder: Color Prestained Protein Standard, Broad Range (10-250 kDa) - NEB

Method used to capture the image: iBright FL1500 Imaging System - Invitrogen

Figure panel generated from the original image is marked in white.
