# Supplementary material for: Unveiling a missing component of the atypical type IV secretion system required for natural transformation of Helicobacter pylori
Source: PLoS Pathog. 2026 Jul 14;22(7):e1014140. doi: 10.1371/journal.ppat.1014140 (PMC13395361; doi:10.1371/journal.ppat.1014140)
Supplement: Attachment — Submitted filename: Response to the Reviewers.docx [file ppat.1014140.s018.docx]

**Response to the Reviewers**

We will like first to thank both reviewers for their thorough review of our manuscript and suggesting modifications and clarifications that we believe have significantly improved it.

Point by point response to Major Issues:

1. Homology search
   The manuscript states:
   “A homology search for VirB11-type ATPases pointed, in addition to the Cag-associated and well characterised HP0525 (Cagα), to the protein coded by hp1421.”
   However, no methodological details are provided regarding this homology search. It is unclear whether this was performed using BLASTP, HMM-based approaches, Foldseek, or another method. This information is essential and should be included.

The original search for homologues of VirB11 was done using BLASTP. The two more significant hits were HP0525 and HP1421. Since we knew about the role of HP0525 in the Cag system, we focused on the uncharacterised HP1421. This is now stated in the text.

In addition, given the apparent similarity of ComB11 to other VirB11 homologues (both at the sequence and structural levels), an important question arises: why was comB11 not identified earlier? Maybe a brief discussion addressing this point would strengthen the manuscript.

As now mentioned in the discussion, HP1421 had been identified as a VirB11 homologue. However, the mutant tested for transformation was a transposon insertion at position 20, leaving an ATG codon just downstream of the insertion leaving the possibility of expressing a protein that, albeit truncated, would conserve most of its sequence and still harbour its active site. This could explain its lack of effect on transformation. Furthermore, transformation with genomic DNA was not tested in that work. This is now developed in the Discussion section.

2. AlphaFold2 vs AlphaFold3 usage
   The authors predominantly rely on AlphaFold3 models, but AlphaFold2 is also used in some instances without clear justification. This inconsistency may confuse the reader.

   For example, Figure S4B (AlphaFold2) appears redundant with Figure 5 (AlphaFold3). For coherence and clarity, the authors should either:
   i) justify explicitly why both versions are used, or
   ii) perform all structural analyses using AlphaFold3 only.

AlphaFold3 is used for modelling all complexes in the study. AlphaFold2 is used in addition to AlphaFold3, only for the HP1421-ComB4 interface. For this interface, we perform both AlphaFold3 modelling (one run modelling the interaction between a HP1421 hexamer and a ComB4 C-terminal domain hexamer, and one modelling run using single copies of the full-length proteins) and AlphaFold2 modelling (one modelling run using single copies of the full-length proteins, and one modelling run using only the interacting domain of each protein). This is because recent studies have shown that AlphaFold3 can yield more false positives than AlphaFold2 for protein-protein interaction prediction, by producing highly confident predictions of interactions between random pairs (see e.g. Lambourne *et al.* Experimental assessment of AI-based interactome mapping. *Nat Commun* (2026). <https://doi.org/10.1038/s41467-026-70942-x>). Thus, the fact that both AlphaFold2 and AlphaFold3 produce highly scored and very similar predictions reinforces the confidence that we place in this interaction. We edited the text in the results section “HP1421 interaction with ComB4 is critical for NT” and methods section “AlphaFold modelling” to better explain this.

3. Structural figures and presentation
   Figure 3D lacks annotation and does not appear particularly informative in its current form. Replacing it with Figure S3B would improve clarity and relevance.

We agree that Figure 3D is mostly illustrative of an HP1421 hexameric model. We thus moved it to simply illustrate the peak in the chromatogram of Figure 3C. Figure S3B on the other hand shows the ATP binding site in the HP1421 model, which is associated to main Figure 4 and the following section of the results, so we have now fused them in Figure 4

Additionally, reorganising the figures would strengthen the narrative. For instance, moving this structural panel (Fig 3D) to Figure 4 would allow a more logical progression: first presenting the AlphaFold3 model with ATP and highlighting residue E176, followed by the functional consequences of its mutation.

The order and organization of the figures follow the logic of the results text: we first illustrate the HP1421 hexameric stoichiometry (Figure 3), then its ATPase activity (Figure 4), then its interaction with ComB4 (Figure 5). As mentioned above, by adding the original FigS3B to Figure 4 the results concerning the ATPase mutants are grouped in a single figure.

More generally, the orientation of structural models is not standardised across figures and often lacks a clear reference frame. It would greatly improve readability to adopt a consistent and biologically meaningful orientation throughout, for instance aligning all structures relative to the membrane (e.g., cytoplasmic side at the bottom and periplasmic side at the top). Maybe including a schematic indication of the membrane would further facilitate interpretation.

We thank the reviewer for this suggestion and we have changed the orientation of all structural models to make them consistent across figures. The membrane was not included in the modelling, but we now have stated the orientation in the legends for the figures.

Point by point response to Minor Issues:

Reviewer #1:

- In the section “HP1421 is required for natural transformation” (line 123), both the strong ureA promoter and the weaker comH promoter are used, and both restore wild-type levels. However, only the ureA promoter is used throughout the rest of the study. The authors should either justify this choice or simplify by removing the comH data (Fig. 1A).

We have tested both promoter since i) we wanted to make sure that the level of expression was not affecting the phenotype; ii) for some of the later experiments (looking at mutants in ComB4 and HP1421) we needed to combine two ectopic expression constructs in the same cell.

- In the section “HP1421 is a cytoplasmic ATPase”, the E176 residue is not properly introduced. It should be explicitly mentioned earlier (e.g., when discussing conserved catalytic residues). In addition, the rationale for choosing E→A and E→K substitutions should be quickly explained.

We now added to the text to mention the presence of the E176 residue within the Asp box. We also cite there the work that described the effect of mutating this residue on the enzymatic activity.

- In Figure 4, transformation frequencies are shown for both E176A and E176K mutants (panel A), whereas only E176K is analysed for tDNA foci (panel B). This inconsistency should be addressed. The authors should either: i) justify this choice in the text, or ii) present both mutants consistently. Alternatively, focusing on a single mutant throughout the manuscript may improve clarity.

We have now added to the figure the result of a new experiment showing that the E176A mutant is also devoid of DNA foci.

- Typographical issue: “comB2-3-4-6-9-9-10-11” likely contains an error (duplicate “9”). This should be corrected (or if not an error, maybe rephrasing ?).

We apologise for the typing error. It should read “comB2-3-4-6-8-9-10-11”. We have now corrected this in the text.

- Regarding the genomic organisation, the authors do not describe the genetic environment surrounding comB11. Providing this information could be informative.

Thank you for pointing out this. We have now added a brief description of the context in which *ComB11* is present in the Results section.

- Figure 3B requires further explanation, as it is not sufficiently clear in its current form.

We have now added a more detailed description including that of the controls.

- The ΔcomB2 result (Fig. 1B) is not described in the Results section.

A sentence explaining this control was now added in the Results.

Minor typographical corrections:
• Fig. S2A: “12.5%” instead of “12,5%”; missing space in “L: ladder”
• Part of Fig. 3A appears duplicated in Fig. 3C
• Fig. 4A: incorrect symbol (question mark instead of Δ)
• Line 234: “1.99×10⁻7” formatting
• Line 281: “treatments” (not “treatements”)
• Line 529: extra “r” in “solutionr”
• Line 583: missing table reference
• Line 264–265: clarify whether the experiment was performed after natural transformation

We thank the reviewer for pointing at this errors that have now been corrected in the new version of the manuscript.

Reviewer #2:

1. The text describes the apparent absence (so far) of a VirB11 component from the ComB system as “puzzling” (line 385). However, VirB11 is also missing in some other type IV secretion systems, including (according to Ref. 19) the F conjugation system, and the gonococcal GGI DNA export system. Furthermore, an early paper (Bohne et al. (1998), doi: 10.1073/pnas.95.12.7057) reported that VirB11 was specifically not important in the recipient for supporting DNA uptake during conjugation via the A. tumefaciens VirB/D4 system (while most other VirB components were required, but the mechanism was not elucidated). Would you expect that VirB11 components may also be identified in the above-mentioned systems? This could be discussed.

We have now addressed the first part of this comment in the Discussion with the following sentence including the relevant references: “While in many conjugative systems VirB11 is not present, in the Mating-pair formation T (MPF_T_) clade of T4SS, to which ComB belongs, VirB11 is usually regarded as essential [44], as shown for the *Agrobacterium* T4SS [53].”

As for the second part of the comment, the observation cited reflects a so far unexplained mechanism where the recipient can affect its own conjugation rates. We have not found a follow up in the literature. In that paper Virb6 would also not be essential, but virB6 (or a homologue) is one of the few proteins present in all T4SS. Therefore, for the sake of clarity in the conclusions, we chose not to address what seems an exceptional situation.

2. Fig. 7B: It is interesting that none of the comB genes except comB11 seems to be present in all H. pylori strains. Apart from genes potentially not annotated due to their small size (such as comB7), did you check whether strains lacking individual comB genes are at the same time devoid of (several) others? In other words, do you find strains that do not contain any comB gene at all (except comB11)? Or is only one comB gene missing in each case (or present as a pseudogene, but not annotated because of that), while all others are there? These considerations might make a difference for the conclusion (lines 427-429) that comB2 to comB10 genes are non-core genes.

We thank the reviewer for raising this point. We have now addressed it with the following sentences included in the revised version: “While *comB11* is the only ubiquitous gene in the species, most of the genes regarded as central for the function of T4SS and that are very conserved in sequence (ComB4, 6, 8-10) are present in the vast majority (95%) of the strains [44]. Hence, the ubiquity of *comB11*, while intriguing, is not that exceptional. It can be due to the presence of another function for ComB11. It may also be related with the fact that comB11 is in an operon with other genes whose inactivation, or silencing due to polar effects, is likely to be deleterious to the bacterial fitness. This may lead to lower rates of pseudogenization of the gene. Interestingly, the *comB11* gene in the 26695 reference genome is an operon with genes encoding proteins of unrelated function, notably a flagellar export protein ATP synthase (FliI), an isoleucyl-tRNA synthetase (ileS) or a putative UDP-N-acetylenolpyruvoylglucosamine reductase [45].”

3. Fig. 2A/B: An additional control showing the donor DNA in a comEC mutant would be helpful here.

Using the same assay, we had previously shown (Damke et al., 2019) that a ComEC defective strain accumulates the same amount of internalised DNA than a wild-type strain. We believe that having one positive (wild-type) and one negative (*ΔcomB2*) control should suffice to make the point that the assay had worked.

4. Lines 169-170: “...foci are detected and stabilized in H. pylori comEC mutants”. Please clarify whether the bacteria analyzed in Fig. 2C/D are (additionally) comEC mutants.

To make this point clear we have now explicitly mentioned in the description of the result that the strains used did not have additional mutations. This can also be seen in the list of strains used (S Table 1) where the complete genotypes are reported.

5. Several references are listed twice. Please revise.

6. Fig. S4A and caption: should read “pLDDT” instead of “pIDDT”.

We apologise for these mistakes and thank the reviewer for detecting them. They have now been corrected.
