# Supplementary material for: Unveiling a missing component of the atypical type IV secretion system required for natural transformation of Helicobacter pylori
Source: PLoS Pathog. 2026 Jul 14;22(7):e1014140. doi: 10.1371/journal.ppat.1014140 (PMC13395361; doi:10.1371/journal.ppat.1014140)
Supplement: Peer review history

# Peer review: Decision Letter 0

**DOI:** 10.1371/journal.ppat.1014140.r001

**Authors:** Christine Josenhans, Thomas Guillard

14 May 2026

PPATHOGENS-D-26-00844

Unveiling a missing component of the atypical type IV secretion system required for natural transformation of Helicobacter pylori

PLOS Pathogens

Dear Dr. Radicella,

We hope this message fimds you well.

Thank you for submitting your manuscript to PLOS Pathogens. After careful consideration, we feel that it has merit but does not fully meet PLOS Pathogens's publication criteria as it currently stands. Therefore, we invite you to submit a revised version of the manuscript that addresses all the points raised during the review process.

Please submit your revised manuscript by Jul 13 2026 11:59PM. If you will need more time than this to complete your revisions, please reply to this message or contact the journal office at plospathogens@plos.org. When you're ready to submit your revision, log on to https://www.editorialmanager.com/ppathogens/ and select the 'Submissions Needing Revision' folder to locate your manuscript file.

Please include the following items when submitting your revised manuscript:

* A letter that responds to each point raised by the editor and reviewer(s). You should upload this letter as a separate file labeled 'Response to Reviewers'. This file does not need to include responses to any formatting updates and technical items listed in the 'Journal Requirements' section below.

* A marked-up copy of your manuscript that highlights changes made to the original version. You should upload this as a separate file labeled 'Revised Manuscript with Track Changes'.

* An unmarked version of your revised paper without tracked changes. You should upload this as a separate file labeled 'Manuscript'.

If you would like to make changes to your financial disclosure, competing interests statement, or data availability statement, please make these updates within the submission form at the time of resubmission. Guidelines for resubmitting your figure files are available below the reviewer comments at the end of this letter.

As the corresponding author, your ORCID iD is verified in the submission system and will appear in the published article. PLOS supports the use of ORCID, and we encourage all coauthors to register for an ORCID iD and use it as well. Please encourage your coauthors to verify their ORCID iD within the submission system before final acceptance, as unverified ORCID iDs will not appear in the published article. Only the individual author can complete the verification step; PLOS staff cannot verify ORCID iDs on behalf of authors.

We look very much forward to receiving your revised manuscript and thank you very much again for submitting your fine work to PLOS Pathogens.

Kind regards,

Christine Josenhans, Ph.D.

Academic Editor

PLOS Pathogens

Thomas Guillard

Section Editor

PLOS Pathogens

Sumita Bhaduri-McIntosh

Editor-in-Chief

PLOS Pathogens

orcid.org/0000-0003-2946-9497

Michael Malim

Editor-in-Chief

PLOS Pathogens

orcid.org/0000-0002-7699-2064

**Journal Requirements:**

If the reviewer comments include a recommendation to cite specific previously published works, please review and evaluate these publications to determine whether they are relevant and should be cited. There is no requirement to cite these works unless the editor has indicated otherwise.

1) We ask that a manuscript source file is provided at Revision. Please upload your manuscript file as a .doc, .docx, .rtf or .tex. If you are providing a .tex file, please upload it under the item type u2018LaTeX Source Fileu2019 and leave your .pdf version as the item type u2018Manuscriptu2019.

2) We do not publish any copyright or trademark symbols that usually accompany proprietary names, eg ©,  ®, or TM  (e.g. next to drug or reagent names). Therefore please remove all instances of trademark/copyright symbols throughout the text, including:

- ® on pages: 19, 21, and 22

- TM on pages: 19, and 20.

3) Please upload all main figures as separate Figure files in .tif or .eps format. For more information about how to convert and format your figure files please see our guidelines:

https://journals.plos.org/plospathogens/s/figures

4) We have noticed that you have uploaded Supporting Information files, but you have not included a list of legends. Please add a full list of legends for your Supporting Information files after the references list.

5) We are unable to open the following Supporting Information file: S6 Table.hmm. Please kindly revise as necessary and re-upload.

6) In the online submission form, you indicated that  All other data can be requested to the authors. All PLOS journals now require all data underlying the findings described in their manuscript to be freely available to other researchers, either

1. In a public repository

2. Within the manuscript itself

3. Uploaded as supplementary information.

This policy applies to all data except where public deposition would breach compliance with the protocol approved by your research ethics board. If your data cannot be made publicly available for ethical or legal reasons (e.g., public availability would compromise patient privacy), please explain your reasons by return email and your exemption request will be escalated to the editor for approval. Your exemption request will be handled independently and will not hold up the peer review process, but will need to be resolved should your manuscript be accepted for publication. One of the Editorial team will then be in touch if there are any issues.

7) Please amend your detailed Financial Disclosure statement. This is published with the article. It must therefore be completed in full sentences and contain the exact wording you wish to be published.

1) State what role the funders took in the study. If the funders had no role in your study, please state: "The funders had no role in study design, data collection and analysis, decision to publish, or preparation of the manuscript."

2) If any authors received a salary from any of your funders, please state which authors and which funders..

If you did not receive any funding for this study, please simply state: u201cThe authors received no specific funding for this work.u201d

8)  Please ensure that the funders and grant numbers match between the Financial Disclosure field and the Funding Information tab in your submission form. Note that the funders must be provided in the same order in both places as well.

9) Please correct the in-text citation for the tables, which currently appear as 'Table XXXX,' and replace them with the correct table numbers.

**Reviewers' Comments:**

Reviewer's Responses to Questions

**Part I - Summary**

Please use this section to discuss strengths/weaknesses of study, novelty/significance, general execution and scholarship.

Reviewer #1: In this study, the authors identify and characterise an essential gene (hp1421) involved in natural transformation mediated by the ComB T4SS system in Helicobacter pylori, which they rename ComB11 based on its homology to VirB11 ATPases from conjugative T4SS. Using a combination of structural prediction, biochemical assays, and microscopy approaches, the authors demonstrate (i) the interaction between ComB11 and ComB4, (ii) the ATPase activity of ComB11, and (iii) its essential role in the early step of natural transformation, namely the entry of transforming DNA into the periplasm.

Overall, the manuscript is well written and easy to follow. The identification of ComB11 as a missing component of the ComB system is novel and of clear interest. Notably, the manuscript avoids overinterpretation, and the conclusions are consistently supported by experimental evidence. However, several points should be clarified or improved before publication.

Reviewer #2: Villa et al. analyzed the role of a yet uncharacterized VirB11 homologue (HP1421) for natural transformation competence of the gastric pathogen Helicobacter pylori. Unlike all other naturally competent bacteria, H. pylori is known to utilize a type IV secretion system (the ComB system) for uptake of transforming DNA into the periplasmic space. The ComB system comprises homologues of the VirB2 to VirB4, and VirB6 to VirB10 proteins from the reference Agrobacterium tumefaciens VirB/D4 type IV secretion system, all of which are essential for transformation, but a ComB analogue of VirB11, which is an essential ATPase in most type IV secretion systems, has not been described so far.

In this study, the authors show that deletion of the hp1421 gene fully abolishes H. pylori transformation competence, and that this defect of the mutant is restored upon complementation with the hp1421 gene. Deletion of hp1421 rendered the mutant strain incapable of accumulating transforming DNA in its periplasm. Next, the authors produced recombinant HP1421 and demonstrated that it has ATPase activity (depending on the presence of a Walker A motif), and that this activity is required in H. pylori for transformation. They further show that HP1421 forms hexamers, and that it interacts with the other hexameric ATPase, ComB4. Mutation of charged HP1421 or ComB4 residues at their interaction interface (as deduced from a corresponding AlphaFold model) abolished the interaction between both proteins, and resulted in loss of transformation competence as well (but retained ATPase activity and hexameric assembly). With these observations, the authors propose to rename HP1421 as ComB11, which seems reasonable. Finally, the authors determined the presence of comB11 and the other comB genes in 364 complete genome sequences of H. pylori strains, and of other Helicobacter species.

This study convincingly elucidates an important function in this unusual type IV secretion system. It will certainly be of great interest for all researchers in the type IV secretion, as well as in the Helicobacter field. The manuscript is also very clear and well-written. I have listed only a few minor comments below that should be addressed in order to clarify certain points.

**********

**Part II – Major Issues: Key Experiments Required for Acceptance**

Please use this section to detail the key new experiments or modifications of existing experiments that should be absolutely required to validate study conclusions.

Generally, there should be no more than 3 such required experiments or major modifications for a "Major Revision" recommendation. If more than 3 experiments are necessary to validate the study conclusions, then you are encouraged to recommend "Reject".

Reviewer #1: 1. Homology search

The manuscript states:

“A homology search for VirB11-type ATPases pointed, in addition to the Cag-associated and well characterised HP0525 (Cagα), to the protein coded by hp1421.”

However, no methodological details are provided regarding this homology search. It is unclear whether this was performed using BLASTP, HMM-based approaches, Foldseek, or another method. This information is essential and should be included.

In addition, given the apparent similarity of ComB11 to other VirB11 homologues (both at the sequence and structural levels), an important question arises: why was comB11 not identified earlier? Maybe a brief discussion addressing this point would strengthen the manuscript.

2. AlphaFold2 vs AlphaFold3 usage

The authors predominantly rely on AlphaFold3 models, but AlphaFold2 is also used in some instances without clear justification. This inconsistency may confuse the reader.

For example, Figure S4B (AlphaFold2) appears redundant with Figure 5 (AlphaFold3). For coherence and clarity, the authors should either:

i) justify explicitly why both versions are used, or

ii) perform all structural analyses using AlphaFold3 only.

3. Structural figures and presentation

Figure 3D lacks annotation and does not appear particularly informative in its current form. Replacing it with Figure S3B would improve clarity and relevance.

Additionally, reorganising the figures would strengthen the narrative. For instance, moving this structural panel (Fig 3D) to Figure 4 would allow a more logical progression: first presenting the AlphaFold3 model with ATP and highlighting residue E176, followed by the functional consequences of its mutation.

More generally, the orientation of structural models is not standardised across figures and often lacks a clear reference frame. It would greatly improve readability to adopt a consistent and biologically meaningful orientation throughout, for instance aligning all structures relative to the membrane (e.g., cytoplasmic side at the bottom and periplasmic side at the top). Maybe including a schematic indication of the membrane would further facilitate interpretation.

Reviewer #2: None.

**********

**Part III – Minor Issues: Editorial and Data Presentation Modifications**

Please use this section for editorial suggestions as well as relatively minor modifications of existing data that would enhance clarity.

Reviewer #1: - In the section “HP1421 is required for natural transformation” (line 123), both the strong ureA promoter and the weaker comH promoter are used, and both restore wild-type levels. However, only the ureA promoter is used throughout the rest of the study. The authors should either justify this choice or simplify by removing the comH data (Fig. 1A).

- In the section “HP1421 is a cytoplasmic ATPase”, the E176 residue is not properly introduced. It should be explicitly mentioned earlier (e.g., when discussing conserved catalytic residues). In addition, the rationale for choosing E→A and E→K substitutions should be quickly explained.

- In Figure 4, transformation frequencies are shown for both E176A and E176K mutants (panel A), whereas only E176K is analysed for tDNA foci (panel B). This inconsistency should be addressed. The authors should either: i) justify this choice in the text, or ii) present both mutants consistently. Alternatively, focusing on a single mutant throughout the manuscript may improve clarity.

- Typographical issue: “comB2-3-4-6-9-9-10-11” likely contains an error (duplicate “9”). This should be corrected (or if not an error, maybe rephrasing ?).

- Regarding the genomic organisation, the authors do not describe the genetic environment surrounding comB11. Providing this information could be informative.

- Figure 3B requires further explanation, as it is not sufficiently clear in its current form.

- The ΔcomB2 result (Fig. 1B) is not described in the Results section.

Minor typographical corrections:

• Fig. S2A: “12.5%” instead of “12,5%”; missing space in “L: ladder”

• Part of Fig. 3A appears duplicated in Fig. 3C

• Fig. 4A: incorrect symbol (question mark instead of Δ)

• Line 234: “1.99×10⁻7” formatting

• Line 281: “treatments” (not “treatements”)

• Line 529: extra “r” in “solutionr”

• Line 583: missing table reference

• Line 264–265: clarify whether the experiment was performed after natural transformation

Reviewer #2: Minor points:

1. The text describes the apparent absence (so far) of a VirB11 component from the ComB system as “puzzling” (line 385). However, VirB11 is also missing in some other type IV secretion systems, including (according to Ref. 19) the F conjugation system, and the gonococcal GGI DNA export system. Furthermore, an early paper (Bohne et al. (1998), doi: 10.1073/pnas.95.12.7057) reported that VirB11 was specifically not important in the recipient for supporting DNA uptake during conjugation via the A. tumefaciens VirB/D4 system (while most other VirB components were required, but the mechanism was not elucidated). Would you expect that VirB11 components may also be identified in the above-mentioned systems? This could be discussed.

2. Fig. 7B: It is interesting that none of the comB genes except comB11 seems to be present in all H. pylori strains. Apart from genes potentially not annotated due to their small size (such as comB7), did you check whether strains lacking individual comB genes are at the same time devoid of (several) others? In other words, do you find strains that do not contain any comB gene at all (except comB11)? Or is only one comB gene missing in each case (or present as a pseudogene, but not annotated because of that), while all others are there? These considerations might make a difference for the conclusion (lines 427-429) that comB2 to comB10 genes are non-core genes.

3. Fig. 2A/B: An additional control showing the donor DNA in a comEC mutant would be helpful here.

4. Lines 169-170: “...foci are detected and stabilized in H. pylori comEC mutants”. Please clarify whether the bacteria analyzed in Fig. 2C/D are (additionally) comEC mutants.

5. Several references are listed twice. Please revise.

6. Fig. S4A and caption: should read “pLDDT” instead of “pIDDT”.

**********

PLOS authors have the option to publish the peer review history of their article (what does this mean?). If published, this will include your full peer review and any attached files.

If you choose “no”, your identity will remain anonymous but your review may still be made public.

**Do you want your identity to be public for this peer review?**  For information about this choice, including consent withdrawal, please see our Privacy Policy.

Reviewer #1: **Yes:**  Kévin MACÉ

Reviewer #2: **Yes:**  Wolfgang Fischer

**Figure resubmission:**

While revising your submission, we strongly recommend that you use PLOS’s NAAS tool (https://ngplosjournals.pagemajik.ai/artanalysis) to test your figure files. NAAS can convert your figure files to the TIFF file type and meet basic requirements (such as print size, resolution), or provide you with a report on issues that do not meet our requirements and that NAAS cannot fix.

After uploading your figures to PLOS’s NAAS tool - https://ngplosjournals.pagemajik.ai/artanalysis, NAAS will process the files provided and display the results in the "Uploaded Files" section of the page as the processing is complete. If the uploaded figures meet our requirements (or NAAS is able to fix the files to meet our requirements), the figure will be marked as "fixed" above. If NAAS is unable to fix the files, a red "failed" label will appear above. When NAAS has confirmed that the figure files meet our requirements, please download the file via the download option, and include these NAAS processed figure files when submitting your revised manuscript.

**Reproducibility:**

To enhance the reproducibility of your results, we recommend that authors of applicable studies deposit laboratory protocols in protocols.io, where a protocol can be assigned its own identifier (DOI) such that it can be cited independently in the future. Additionally, PLOS ONE offers an option to publish peer-reviewed clinical study protocols. Read more information on sharing protocols at https://plos.org/protocols?utm_medium=editorial-email&utm_source=authorletters&utm_campaign=protocols

# Author comment: Author response to Decision Letter 1

**DOI:** 10.1371/journal.ppat.1014140.r002

19 Jun 2026

Attachment Submitted filename: Response to the Reviewers.docx

# Editor report: Decision Letter 1

**DOI:** 10.1371/journal.ppat.1014140.r003

**Authors:** Christine Josenhans, Thomas Guillard

25 Jun 2026

Dear Dr. Radicella,

We are pleased to inform you that your manuscript 'Unveiling a missing component of the atypical type IV secretion system required for natural transformation of Helicobacter pylori' has been provisionally accepted for publication in PLOS Pathogens.

Before your manuscript can be formally accepted you will need to complete some formatting changes, which you will receive in a follow up email. A member of our team will be in touch with a set of requests.

Please note that your manuscript will not be scheduled for publication until you have made the required changes, so a swift response is appreciated.

IMPORTANT: The editorial review process is now complete. PLOS will only permit corrections to spelling, formatting or significant scientific errors from this point onwards. Requests for major changes, or any which affect the scientific understanding of your work, will cause delays to the publication date of your manuscript.

Should you, your institution's press office or the journal office choose to press release your paper, you will automatically be opted out of early publication. We ask that you notify us now if you or your institution is planning to press release the article. All press must be co-ordinated with PLOS.

Thank you again for supporting Open Access publishing; we are looking forward to publishing your work in PLOS Pathogens.

Best regards,

Christine Josenhans, Ph.D.

Academic Editor

PLOS Pathogens

Thomas Guillard

Section Editor

PLOS Pathogens

Sumita Bhaduri-McIntosh

Editor-in-Chief

PLOS Pathogens

orcid.org/0000-0003-2946-9497

Michael Malim

Editor-in-Chief

PLOS Pathogens

orcid.org/0000-0002-7699-2064

***********************************************************

The authors have responded with very detailed comments and correction to all reviewers' requests.

We are happy that you have considered Plos Pathogens for the submission of your work.

Reviewer Comments (if any, and for reference):

# Editor report: Acceptance letter

**DOI:** 10.1371/journal.ppat.1014140.r004

**Authors:** Christine Josenhans, Thomas Guillard

Dear Dr. Radicella,

We are delighted to inform you that your manuscript, "Unveiling a missing component of the atypical type IV secretion system required for natural transformation of Helicobacter pylori," has been formally accepted for publication in PLOS Pathogens.

We have now passed your article onto the PLOS Production Department who will complete the rest of the pre-publication process. All authors will receive a confirmation email upon publication.

The corresponding author will soon be receiving a typeset proof for review, to ensure errors have not been introduced during production. Please review the PDF proof of your manuscript carefully, as this is the last chance to correct any scientific or type-setting errors. Please note that major changes, or those which affect the scientific understanding of the work, will likely cause delays to the publication date of your manuscript. Note: Proofs for Front Matter articles (Pearls, Reviews, Opinions, etc...) are generated on a different schedule and may not be made available as quickly.

Soon after your final files are uploaded, the early version of your manuscript, if you opted to have an early version of your article, will be published online. The date of the early version will be your article's publication date. The final article will be published to the same URL, and all versions of the paper will be accessible to readers.

For Research Articles, you will receive an invoice from PLOS for your publication fee after your manuscript has reached the completed accept phase. If you receive an email requesting payment before acceptance or for any other service, this may be a phishing scheme. Learn how to identify phishing emails and protect your accounts at https://explore.plos.org/phishing.

Thank you again for supporting open-access publishing; we are looking forward to publishing your work in PLOS Pathogens.

Best regards,

Sumita Bhaduri-McIntosh

Editor-in-Chief

PLOS Pathogens

orcid.org/0000-0003-2946-9497

Michael Malim

Editor-in-Chief

PLOS Pathogens

orcid.org/0000-0002-7699-2064
